# Supplementary material for: Asymmetric α-allylic allenylation of β-ketocarbonyls and aldehydes by synergistic Pd/chiral primary amine catalysis
Source: Nat Commun. 2023 May 22;14:2911. doi: 10.1038/s41467-023-38488-4 (PMC10203310; doi:10.1038/s41467-023-38488-4)
Supplement: Supplementary file 1 — Supplementary information [file 41467_2023_38488_MOESM1_ESM.pdf]

Asymmetric α-Allylic Allenylation of β-Ketocarbonyls and Aldehydes by Synergistic Pd/Chiral Primary Amine Catalysis

Chang You[1], Mingying Shi[2], Xueling Mi[2]*, and Sanzhong Luo[1]*

[1] Center of Basic Molecular Science, Department of Chemistry, Tsinghua University Beijing 100084

[2] College of Chemistry, Beijing Normal University Beijing 100875

*Corresponding Author(s): luosz@tsinghua.edu.cn; xlmi@bnu.edu.cn

Supplementary Information

Table of Contents

1. Supplementary Methods....................S3

1.1 General information and materials....................S3

1.2 General experiment procedure....................S3

2. Supplementary Discussion....................S4

2.1 X-ray crystallographic structure of 5b....................S4

2.2 Stereodivergent synthesis of all four isomers....................S5

2.3 Scale-up experiment of 3l....................S6

2.4 Synthetic transformations of 3a....................S6

3. Supplementary Notes....................S7

4. Supplementary Figures....................S20

5.1 NMR spectra....................S20

5.2 HPLC spectra....................S80

5. Supplementary References....................S124

1. Supplementary Methods

1.1 General information and materials

General information: All commercial reagents were used without further purification unless otherwise noted. Nuclear magnetic resonance (NMR) was recorded on Bruker AV-400 and AV-500 spectrometers. Proton and carbon magnetic resonance spectra (^{1}H NMR, ^{13}C NMR and ^{19}F NMR) were measured on a NMR instrument (400 MHz for ^{1}HNMR, 101 MHz for ^{13}C NMR, and 376 MHz for ^{19}FNMR,) with solvent resonance as the internal standard (^{1}H NMR: $CDCl_3$ at 7.26 ppm; ^{13}C NMR: $CDCl_3$ at 77.16 ppm). ^{1}H NMR data were reported as follows: chemical shift, multiplicity (s = singlet, d = doublet, t = triplet, m = multiplet, td = triplet of doublet, dt = doublet of triplet, dd = doublet of doublet), coupling constants (Hz), and integration. The enantiomeric excesses were determined by HPLC analysis on Chiral Daicel Chiralpak AD-H, IC-H, IA-H, AS-H, OJ-H, OD-H columns. Optical rotation was measured on a commercial polarimeter and reported as follows: $[\alpha]_D^{25}$ (c = g/100 mL, solvent). Infrared Spectroscopy was conducted on Thermo Fisher Nicolet 6700. High resolution mass spectra were obtained using electrospray ionization (ESI), Atmospheric Pressure Chemical Ionization (APCI) and Electron Impact (EI) mass spectrometer. Silica gel (300 - 400 mesh) was used for column chromatography.

Materials: The corresponding *β*-ketocarbonyl substrates were prepared according to reported procedures.[1] The *α*-branched aldehydes were prepared following literature precedent.[2]

1.2 General experiment procedure

Enynes **1a**-**1j**, **1l**-**1q**, **1t**-**1dd** are known compounds, which were prepared according to previously reported procedures.[3-4]

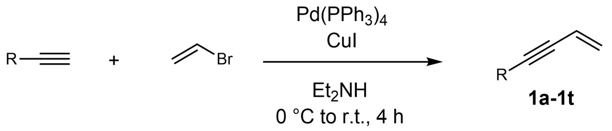

General procedure: To a three-necked flask with $Pd(PPh_3)_4$ (0.5 mmol) and CuI (1 mmol) was added Et_2NH (5.0 mL) under argon. Then alkyne (10 mmol) and bromoethene (1M in THF, 13 mL) was added to above solution dropwise under ice bath. The resulting solution was stirred at room temperature for 4 h. After this time, it was quenched by HCl aqueous solution (1.0 M, 15 mL), extracted by DCM (50 mL x 3), concentrated and purified by flash column chromatography to provide the pure product.

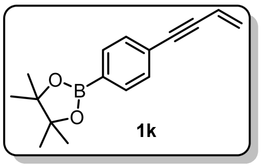

1k. pale yellow soild, 67% yield. ^{1}H NMR (400 MHz, $CDCl_3$) δ 7.79 - 7.70 (m, 2H), 7.47 - 7.39 (m, 2H), 6.02 (dd, *J* = 17.6, 11.2 Hz, 1H), 5.75 (dd, *J* = 17.6, 2.1 Hz, 1H), 5.56 (dd, *J* = 11.1, 2.1 Hz, 1H), 1.34 (s, 12H). ^{13}C NMR (101 MHz, $CDCl_3$) δ 134.6, 130.7, 127.2, 125.8, 117.1, 90.1, 89.4, 84.0, 24.9. HRMS (ESI) calcd for $C_{16}H_{20}BO_2^+$ (M+H$^+$): 255.1551, found: 255.1553.

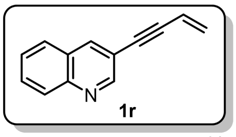

1r. pale brown oil, 54% yield. ^{1}H NMR (400 MHz, $CDCl_3$) δ 8.91 (d, *J* = 2.1 Hz, 1H), 8.20 (d, *J* = 1.9 Hz, 1H), 8.08 (dd, *J* = 8.5, 1.2 Hz, 1H), 7.75 (dd, *J* = 8.2, 1.5 Hz, 1H), 7.70 (ddd, *J* = 8.4, 6.9, 1.5 Hz, 1H), 7.54 (ddd, *J* = 8.1, 6.9, 1.2 Hz, 1H), 6.07 (dd, *J* = 17.5, 11.2 Hz, 1H), 5.83 (dd, *J* = 17.6, 2.0 Hz, 1H), 5.64 (dd, *J* = 11.2, 2.0 Hz, 1H). ^{13}C NMR (101 MHz, $CDCl_3$) δ 152.1, 146.8, 138.3, 130.1, 129.4, 128.1, 127.6, 127.3, 127.2, 117.3, 116.8, 91.3, 87.2.HRMS (ESI) calcd for $C_{13}H_{10}N^+$ (M+H$^+$): 180.0808, found: 180.0808.

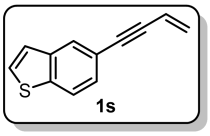

1s. pale yellow oil, 73% yield. ^{1}H NMR (400 MHz, $CDCl_3$) δ 7.70 (d, *J* = 1.9 Hz, 1H), 7.61 (d, *J* = 2.2 Hz, 1H), 7.44 (dt, *J* = 8.5, 0.9 Hz, 1H), 7.38 (dd, *J* = 8.6, 1.7 Hz, 1H), 6.72 (dd, *J* = 2.3, 0.9 Hz, 1H), 6.03 (dd, *J* = 17.5, 11.2 Hz, 1H), 5.73 (dd, *J* = 17.5, 2.1 Hz, 1H),

5.53 (dd, J = 11.2, 2.1 Hz, 1H). ^{13}C NMR (101 MHz, $CDCl_3$) δ 154.6, 145.9, 128.0, 127.6, 126.5, 124.7, 117.7, 117.4, 111.6, 106.5, 90.4, 86.8. HRMS (ESI) calcd for $C_{12}H_9S^+$ (M+H$^+$): 185.0419, found: 185.0420.

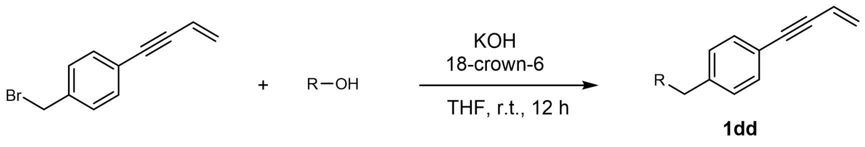

To a solution of alcohol (4.5 mmol), 18-crown-6 (0.3 mmol) in dry THF (20 mL) was added the KOH (18 mmol). The reaction was stirred at room temperature for 3 h. Then 1-(bromomethyl)-4-(but-3-en-1-yn-1-yl)benzene[3] in THF (5 mL) was added to above solution dropwise. The resulting mixture continued to stir at room temperature for 12 h. After this time, the reaction was quenched with water (50 mL), extracted by EtOAc (40 mL × 3), concentrated and purified by flash column chromatography toprovide the pure product.

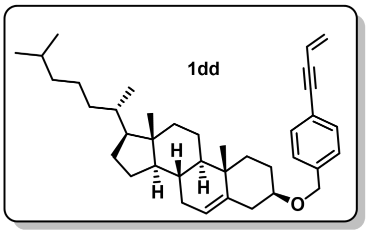

1dd. pale white soild, 57% yield. ^{1}H NMR (400 MHz, $CDCl_3$) δ 7.41 (d, J = 8.2 Hz, 2H), 7.30 (d, J = 7.9 Hz, 2H), 6.02 (ddd, J = 17.5, 11.2, 2.1 Hz, 1H), 5.73 (ddd, J = 17.5, 4.1, 2.1 Hz, 1H), 5.54 (ddd, J = 11.1, 4.6, 2.1 Hz, 1H), 5.38 - 5.30 (m, 1H), 4.55 (s, 2H), 3.26 (tt, J = 11.2, 4.5 Hz, 1H), 2.41 (ddd, J = 13.2, 4.9, 2.3 Hz, 1H), 2.34 – 2.19 (m, 1H), 2.05 - 1.75 (m, 5H), 1.63 - 0.98 (m, 24H), 0.91 (d, J = 6.5 Hz, 3H), 0.86 (dd, J = 6.6, 1.9 Hz, 6H), 0.67 (s, 3H). ^{13}C NMR (101 MHz, $CDCl_3$) δ 140.9, 139.5, 131.6, 127.4, 126.8, 121.7, 117.2, 90.0, 88.0, 78.8, 69.6, 56.8, 56.2, 50.2, 42.3, 39.8, 39.5, 39.2, 37.2, 36.9, 36.2, 35.8, 32.0, 31.9, 28.5, 28.3, 28.0, 24.3, 23.8, 22.8, 22.6, 21.1, 19.4, 18.7, 11.9. HRMS (ESI) calcd for $C_{38}H_{55}O^+$ (M+H$^+$): 527.4247, found: 527.4247.

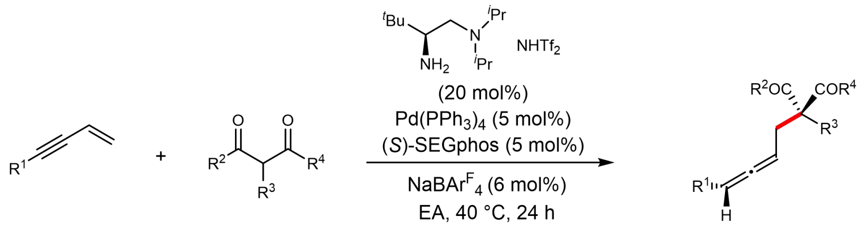

General procedure: In glove box, to a flame-dried Schlenk tube equipped with a magnetic stir bar was added *tert*-butyl 2-methyl-3-oxobutanoate (**2a**, 0.30 mmol), but-3-en-1-yn-1-ylbenzene (**1a**, 0.10 mmol), $Pd(PPh_3)_4$ (5 mol%), (*S*)-SEGphos (5 mol%), $NaBAr^F_4$ (6 mol%) and primary amine (*S*)-**A1** (20 mol%), the mixture was diluted with 0.3 mL of anhydrous EA, then the mixture was moved out from glove box and stirred at 40 °C for 24 h, solvent was removed and residue was purified by silica gel chromatography (10% EtOAc in Petroleum ether) to give **3a** as a colorless oil. The enantiometric excess was determined by HPLC (AD-H*2).

2. Supplementary Discussion

2.1 X-ray crystallographic structure of 5b

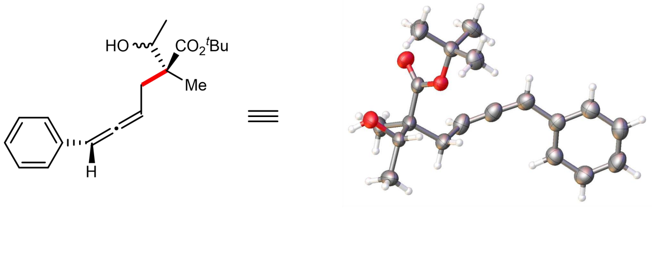

Identification code	ALLENE5 (CCDC 2222345)
Empirical formula	$C_{19}H_{26}O_3$
Formula weight	302.40
Temperature/K	169.99(10)

Crystal system	monoclinic
Space group	$P2_1$
a/Å	8.9972(2)
b/Å	8.7603(2)
c/Å	11.1797(2)
α/°	90
β/°	97.733(2)
γ/°	90
Volume/Å^3	873.15(3)
Z	2
ρ_{calc}g/cm^3	1.150
μ/mm^{-1}	0.604
F(000)	328.0
Crystal size/mm^3	0.15 × 0.13 × 0.12
Radiation	Cu Kα (λ = 1.54184)
2Θ range for data collection/°	7.98 to 133.94
Index ranges	$-10 \le h \le 10$, $-10 \le k \le 10$, $-13 \le l \le 13$
Reflections collected	15845
Independent reflections	3026 [R_{int} = 0.0384, R_{sigma} = 0.0204]
Data/restraints/parameters	3026/128/245
Goodness-of-fit on F^2	1.042
Final R indexes [I>=2σ (I)]	R_1 = 0.0345, wR_2 = 0.0862
Final R indexes [all data]	R_1 = 0.0364, wR_2 = 0.0893
Largest diff. peak/hole / e Å^{-3}	0.11/-0.11
Flack parameter	0.07(9)

2.2 Stereodivergent synthesis of all four isomers

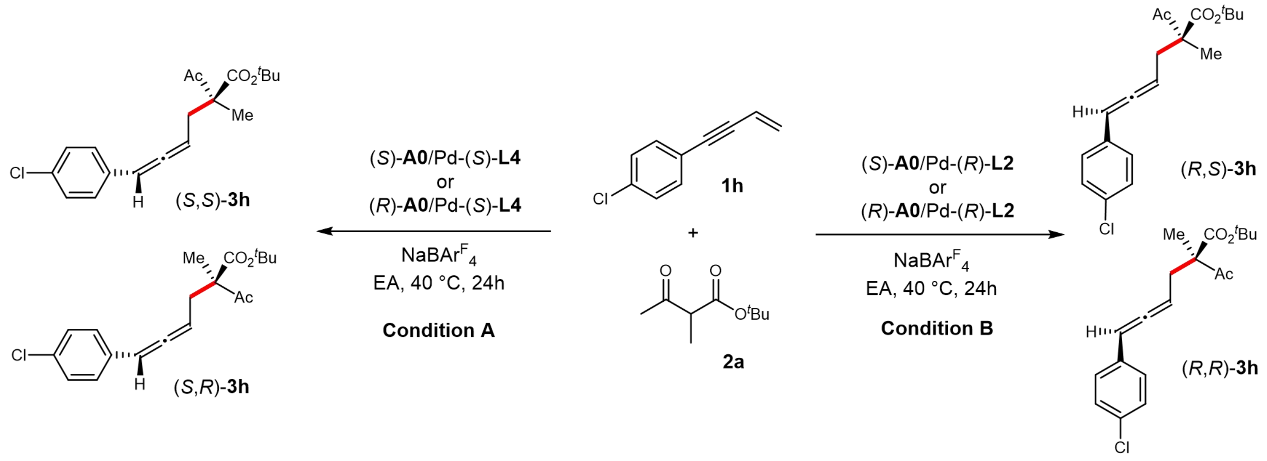

General procedure A: In glove box, to a flame-dried Schlenk tube equipped with a magnetic stir bar was added *tert*-butyl 2-methyl-3-oxobutanoate (**2a**, 0.30 mmol), enyne (**1h**, 0.10 mmol), $Pd(PPh_3)_4$ (5 mol%), (*S*)-SEGphos (5 mol%), $NaBAr^F_4$ and primary amine (*S*)-**A0 or** (*R*)-**A0** (20 mol%), the mixture was diluted with 0.3 mL of anhydrous EA, then the mixture was moved out from glove box and stirred at 40 °C for 24 h, solvent was removed and residue was purified by silica gel chromatography (10% EtOAc in Petroleum ether) to give **3h** as a colorless oil. The enantiometric excess was determined by HPLC (AD-H*2).

General procedure B: In glove box, to a flame-dried Schlenk tube equipped with a magnetic stir bar was added *tert*-butyl 2-methyl-3-oxobutanoate (**2a**, 0.30 mmol), enyne (**1h**, 0.10 mmol), $Pd(PPh_3)_4$ (5 mol%), (*R*)-BINAP (5 mol%), $NaBAr^F_4$ and primary amine (*S*)-**A0 or** (*R*)-**A0** (20 mol%), the mixture was diluted with 0.3 mL of anhydrous EA, then the mixture was moved out from glove box and stirred at 40 °C for 24 h, solvent was removed and residue was purified by silica gel chromatography (10% EtOAc in Petroleum ether) to give **3h** as a colorless oil. The enantiometric excess was determined by HPLC (AD-H*2).

2.3 Scale-up experiment of 3l

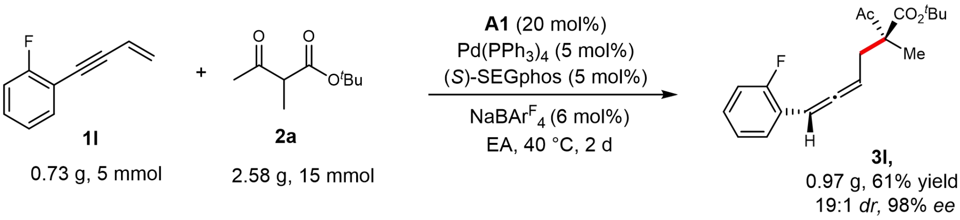

In glove box, Pd(PPh$_3$)$_4$ (289 mg, 0.25 mmol, 5 mol%) and (*S*)-SEGphos (155 mg, 0.25 mmol, 5 mol%), NaBAr^F$_4$ (318.6 mg, 0.3 mmol, 6 mol%), primary amine (*S*)-**A1** (350 mg, 1 mmol, 20 mol%) were dissolved in dry EtOAc (15 mL). To the solution, substrate entry 1a (0.73 g, 5 mmol), *tert*-butyl 2-methyl-3-oxobutanoate **2a** (2.58 g, 15 mmol) were added sequentially. The mixture was moved out from glove box and stirred at 40 °C under atmospheric pressure for 2 days. Then EtOAc (100 mL) was added to dilute the mixture, which was quenched with saturated NH_4Cl solution (100 mL). Then the mixture was extracted with EtOAc (100 mL x 3). The combined organic layer was washed with brine (200 mL x 2), dried over anhydrous Na_2SO_4 , filtered, and concentrated under vacuum. The residue was subjected to flash column chromatography for purification using petroleum ether/EtOAc (10:1) as eluent to give (*S*,*S*)-**3l** (0.97 g, 61% yield, 19:1 *dr*, 98% *ee*).

2.4 Synthetic transformations of 3a

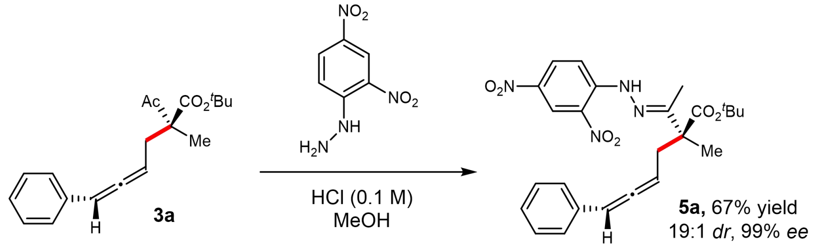

A mixture of the hydrazine (19.8 mg, 0.1 mmol) and **3a** (30 mg, 0.1 mmol) in methanol with the addition of conc. HCl was tirred under room temperature for 2 h. It was filtered off and concentrated in vacuo. The residue was purified by column chromatography (EtOAc:hexane = 1:10) to obtain a bright-yellow solid. Yield of **5a** 32.16 mg (67%). ^{1}H NMR (400 MHz, $CDCl_3$) δ 10.98 (s, 1H), 9.12 (d, *J* = 2.6 Hz, 1H), 8.28 (dd, *J* = 9.6, 2.6 Hz, 1H), 7.93 (d, *J* = 9.6 Hz, 1H), 7.18 - 7.14 (m, 3H), 7.14 - 7.08 (m, 1H), 6.12 (dt, *J* = 6.4, 2.4 Hz, 1H), 5.49 (q, *J* = 7.6 Hz, 1H), 2.87 - 2.68 (m, 2H), 2.01 (s, 3H), 1.54 (s, 3H), 1.47 (s, 9H). ^{13}C NMR (101 MHz, $CDCl_3$) δ 206.7, 172.7, 156.4, 145.2, 138.1, 134.1, 129.9, 129.5, 128.5, 126.9, 126.7, 123.3, 116.7, 94.5, 89.6, 82.0, 55.3, 35.4, 27.9, 20.4, 14.2. HRMS (ESI) calcd for $C_{25}H_{28}N_4NaO_6^+$ (M+Na$^+$): 503.1901, found: 503.1905.

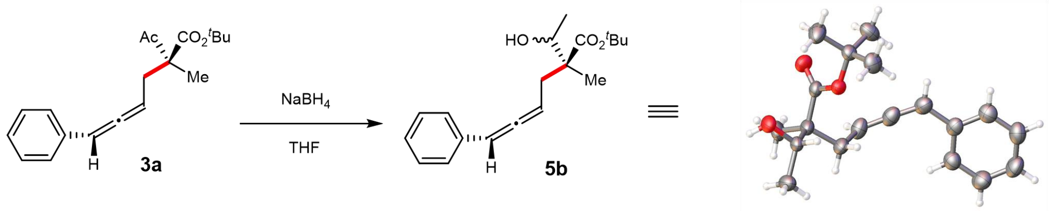

To a stirred solution of **3a** (120 mg, 0.4 mmol) in THF (2 mL) was added $NaBH_4$ (60.5 mg, 1.6 mmol) at room temperature, and the resulting mixture was stirred for 2 h. The reaction mixture was poured into 3 M HCl. Then, saturated aqueous $NaHCO_3$ solution was added to neutralize the mixture, and the resulting mixture was concentrated in vacuo. The residue was extracted with EtOAc twice. The combined organic phases were washed with brine, dried over $MgSO_4$, filtered, and concentrated in vacuo. The residue was purified by column chromatography (EtOAc:hexane = 1:10). Yield **5b** (118.4 mg, 98%) as a white solid and a mixture of diastereomers. ^{1}H NMR (400 MHz, $CDCl_3$) δ 7.36 - 7.26 (m, 4H), 7.19 (pd, *J* = 5.8, 2.4 Hz, 1H), 6.12 (dt, *J* = 6.5, 2.4 Hz, 1H), 5.57 - 5.40 (m, 1H), 4.02 - 3.78 (m, 1H), 2.89 - 2.26 (m, 3R (400 MHzH), 1.49 (d, *J* = 4.5 Hz, 9H), 1.20 (d, *J* = 10.4 Hz, 3H), 1.17 (d, *J* = 2.7 Hz, 3H). ^{13}C NMR (101 MHz, $CDCl_3$) δ 206.4, 175.6, 134.5, 128.6, 126.9, 126.7, 94.2, 90.2, 81.4, 71.6, 51.0, 35.2, 28.1, 18.4, 17.7. HRMS (ESI) calcd for $C_{19}H_{26}NaO_3^+$ (M+Na$^+$): 325.1774, found: 325.1779.

3. Supplementary Notes

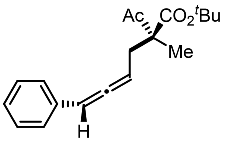

3a

3a. colorless oil, 74% yield, 20:1 *dr*, >99% *ee*. $[\alpha]_D^{20}$ = -18.6 (*c* = 3.5, $CHCl_3$). HPLC analysis: Daicel Chiralpak IC-H, 5% iso-propanol/hexane, flow rate = 1.0 mL/min, λ = 254 nm, retention time: 52.8 min (major), 69.8 min (minor). ^1H NMR (400 MHz, $CDCl_3$) δ 7.35 - 7.19 (m, 5H), 6.15 (dt, *J* = 6.4, 2.4 Hz, 1H), 5.43 (td, *J* = 7.9, 6.3 Hz, 1H), 2.66 (qdd, *J* = 14.3, 7.9, 2.5 Hz, 2H), 2.18 (s, 3H), 1.49 (s, 9H), 1.41 (s, 3H). ^{13}C NMR (101 MHz, $CDCl_3$) δ 206.8, 205.3, 171.5, 134.3, 128.7, 127.1, 126.9, 94.7, 89.7, 82.2, 60.3, 35.0, 28.0, 26.3, 19.0. IR (thin film, cm^{-1}) 2980, 2928, 1711, 1456, 1369, 1253, 1151, 1107, 845, 714. HRMS (ESI) calcd for $C_{19}H_{24}NaO_3^+$ ($M+Na^+$): 323.1683, found: 323.1683.

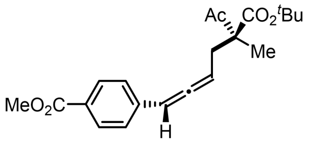

3b

3b. colorless oil, 61% yield, >20:1 *dr*, >99% *ee*. $[\alpha]_D^{20}$ = -4.2 (*c* = 3.9, $CHCl_3$). HPLC analysis: Daicel Chiralpak AD-H*2, 5% iso-propanol/hexane, flow rate = 1.0 mL/min, λ = 254 nm, retention time: 17.8 min (major), 21.0 min (minor). ^1H NMR (400 MHz, $CDCl_3$) δ 8.01 - 7.95 (m, 2H), 7.36 - 7.30 (m, 2H), 6.18 (dt, *J* = 6.5, 2.4 Hz, 1H), 5.50 (td, *J* = 7.9, 6.3 Hz, 1H), 3.92 (s, 3H), 2.67 (qdd, *J* = 14.3, 8.0, 2.4 Hz, 2H), 2.18 (s, 3H), 1.49 (s, 9H), 1.41 (s, 3H). ^{13}C NMR (101 MHz, $CDCl_3$) δ 207.8, 205.1, 171.4, 167.0, 139.4, 130.1, 128.6, 126.8, 94.3, 90.2, 82.3, 60.2, 52.2, 34.7, 28.0, 26.3, 19.0. IR (thin film, cm^{-1}) 2980, 2952, 2929, 1715, 1608, 1457, 1436, 1369, 1279, 1152, 1111, 845, 757. HRMS (ESI) calcd for $C21H_{26}NaO_5^+$ ($M+Na^+$): 381.1672, found: 381.1673.

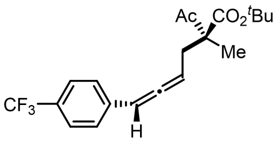

3c

3c. colorless oil, 59% yield, 18:1 *dr*, >99% *ee*. $[\alpha]_D^{20}$ = -18.6 (*c* = 3.8, $CHCl_3$). HPLC analysis: Daicel Chiralpak AD-H*2, 5% iso-propanol/hexane, flow rate = 1.0 mL/min, λ = 254 nm, retention time: 9.2 min (major), 10.8 min (minor). ^1H NMR (400 MHz, $CDCl_3$) δ 7.57 (d, *J* = 8.1 Hz, 2H), 7.37 (d, *J* = 8.1 Hz, 2H), 6.18 (dt, *J* = 6.5, 2.4 Hz, 1H), 5.51 (q, *J* = 7.5 Hz, 1H), 2.67 (qdd, *J* = 14.3, 8.0, 2.4 Hz, 2H), 2.18 (s, 3H), 1.49 (s, 9H), 1.41 (s, 3H). ^{13}C NMR (101 MHz, $CDCl_3$) δ 207.6, 205.1, 171.4, 138.3, 129.2, 128.8, 127.0, 125.7 (q, J=3.5), 93.9, 90.4, 82.3, 60.2, 34.7, 28.0, 26.3, 19.0. ^{19}F NMR (376 MHz, $CDCl_3$) δ -62.4. IR (thin film, cm^{-1}) 2980, 2952, 2929, 1715, 1608, 1457, 1436, 1369, 1279, 1152, 1111, 845, 757. HRMS (ESI) calcd for $C_{20}H_{23}NaO_3^+$ ($M+Na^+$): 391.1490, found: 391.1492.

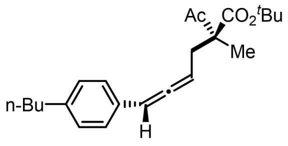

3d

3d. colorless oil, 68% yield, >20:1 *dr*, >99% *ee*. $[\alpha]_D^{20}$ = -12.4 (*c* = 4.2, $CHCl_3$). HPLC analysis: Daicel Chiralpak AD-H*2, 5% iso-propanol/hexane, flow rate = 1.0 mL/min, λ = 254 nm, retention time: 8.3 min (major), 10.3 min (minor). ^1H NMR (400 MHz, $CDCl_3$) δ 7.19 (d, *J* = 8.2 Hz, 2H), 7.13 (d, *J* = 8.2 Hz, 2H), 6.13 (dt, *J* = 6.4, 2.4 Hz, 1H), 5.41 (td, *J* = 7.9, 6.4 Hz, 1H), 2.68 (ddd, *J* = 14.2, 7.6, 2.7 Hz, 1H), 2.66 - 2.56 (m, 3H), 2.18 (s, 3H), 1.67 - 1.54 (m, 2H), 1.49 (s, 9H), 1.41 (s, 3H), 1.40 - 1.28 (m, 2H), 0.94 (t, *J* = 7.3 Hz, 3H). ^{13}C NMR (101 MHz, $CDCl_3$) δ 206.7, 205.3, 171.6, 142.0, 131.5, 128.8, 126.8, 94.5, 89.5, 82.1, 60.3, 35.5, 35.1, 33.8, 28.0, 26.3, 22.5, 19.0, 14.1. IR (thin film, cm^{-1}) 2978, 2929, 1713, 1457, 1370, 1325, 1254, 1165, 1128, 1067, 1018, 845, 757. HRMS (ESI) calcd for $C_{23}H_{32}NaO_3^+$ ($M+Na^+$): 379.2244, found: 379.2242.

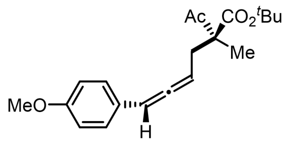

3e

3e. colorless oil, 51% yield, 14:1 *dr*, 95% *ee*. $[\alpha]_D^{20}$ = -13.3 (*c* = 2.9, $CHCl_3$). HPLC analysis: Daicel Chiralpak AD-H*2, 5% isopropanol/hexane, flow rate = 1.0 mL/min, λ = 254 nm, retention time: 12.2 min (major), 15.7 min (minor). ^1H NMR (400 MHz, $CDCl_3$) δ 7.23 - 7.18 (m, 2H), 6.90 - 6.84 (m, 2H), 6.11 (dt, *J* = 6.4, 2.5 Hz, 1H), 5.40 (td, *J* = 8.0, 6.5 Hz, 1H), 3.82 (s, 3H), 2.64 (qdd, *J* = 14.2, 7.9, 2.4 Hz, 2H), 2.18 (s, 3H), 1.49 (s, 9H), 1.40 (s, 3H). ^{13}C NMR (101 MHz, $CDCl_3$) δ 206.3, 205.3, 171.6, 158.9, 128.0, 126.6, 114.3, 94.2, 89.6, 82.1, 60.4, 55.5, 35.2, 28.0, 26.3, 19.0. IR (thin film, cm^{-1}): 2978, 2929, 1711, 1603, 1542, 1512, 1458, 1423, 1369, 1153, 1111, 1030, 845, 772. HRMS (ESI) calcd for $C_{20}H_{26}NaO_4^+$ (M+Na$^+$): 353.1723, found: 353.1723.

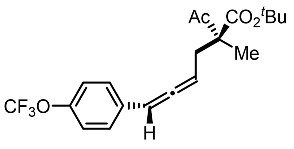

3f

3f. colorless oil, 65% yield, >20:1 *dr*, >99% *ee*. $[\alpha]_D^{20}$ = -15.9 (*c* = 1.9, $CHCl_3$). HPLC analysis: Daicel Chiralpak AD-H*2, 5% isopropanol/hexane, flow rate = 1.0 mL/min, λ = 254 nm, retention time: 8.6 min (major), 10.9 min (minor). ^1H NMR (400 MHz, $CDCl_3$) δ 7.28 - 7.25 (m, 2H), 7.14 (dt, *J* = 7.8, 1.1 Hz, 2H), 6.11 (dt, *J* = 6.4, 2.4 Hz, 1H), 5.44 (td, *J* = 7.9, 6.4 Hz, 1H), 2.63 (qdd, *J* = 14.2, 7.9, 2.4 Hz, 2H), 2.16 (s, 3H), 1.47 (s, 9H), 1.38 (s, 3H). ^{13}C NMR (101 MHz, $CDCl_3$) δ 207.0, 205.1, 171.4, 148.2, 133.2, 128.1, 121.3, 120.6 (q, *J* = 258.6 Hz), 93.6, 90.2, 82.3, 60.2, 34.9, 28.0, 26.3, 19.0. ^{19}F NMR (376 MHz, $CDCl_3$) δ -57.9. IR (thin film, cm^{-1}) 2980,2928, 1713, 1508, 1458, 1370, 1256, 1219, 1163, 1019, 924, 845, 757. HRMS (ESI) calcd for $C_{20}H_{23}F_3NaO_4^+$ (M+Na$^+$): 407.1441, found: 407.1439.

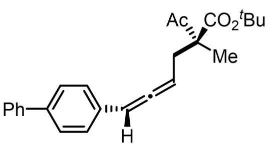

3g

3g. colorless oil, 71% yield, 17:1 *dr*, >99% *ee*. $[\alpha]_D^{20}$ = -12.5 (*c* = 6.9, $CHCl_3$). HPLC analysis: Daicel Chiralpak AD-H*2, 5% isopropanol/hexane, flow rate = 1.0 mL/min, λ = 254 nm, retention time: 12.6 min (major), 15.3 min (minor). ^1H NMR (400 MHz, $CDCl_3$) δ 7.63 - 7.55 (m, 11H), 7.49 - 7.43 (m, 6H), 7.39 - 7.33 (m, 8H), 6.20 (dt, *J* = 6.4, 2.4 Hz, 3H), 5.47 (td, *J* = 7.9, 6.4 Hz, 3H), 2.68 (qdd, *J* = 14.2, 7.9, 2.4 Hz, 6H), 2.20 (s, 8H), 1.50 (s, 25H), 1.43 (s, 8H). ^{13}C NMR (101 MHz, $CDCl_3$) δ 207.1, 205.3, 171.5, 140.9, 139.9, 133.4, 128.9, 127.5, 127.4, 127.3, 127.0, 94.4, 89.8, 82.2, 60.3, 35.0, 28.0, 26.3, 19.0. IR (thin film, cm^{-1}): 2978, 2928, 1710, 1605, 1457, 1369, 1254, 1217, 1152, 1008, 843, 752, 698, 668. HRMS (ESI) calcd for $C_{25}H_{28}NaO_3^+$ (M+Na$^+$): 399.1931, found: 399.1937.

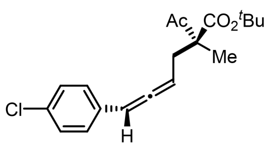

3h

3h. colorless oil, 72% yield, >20:1 *dr*, >99% *ee*. $[\alpha]_D^{20}$ = -9.3 (*c* = 4.0, $CHCl_3$). HPLC analysis: Daicel Chiralpak AD-H*2, 5% isopropanol/hexane, flow rate = 1.0 mL/min, λ = 254 nm, retention time: 9.9 min (major), 12.5 min (minor). ^1H NMR (400 MHz, $CDCl_3$) δ 7.30 - 7.26 (m, 2H), 7.23 - 7.16 (m, 2H), 6.10 (dt, *J* = 6.4, 2.4 Hz, 1H), 5.44 (td, *J* = 8.0, 6.4 Hz, 1H), 2.65 (qdd, *J* = 14.2, 7.9, 2.4 Hz, 2H), 2.17 (s, 3H), 1.49 (s, 9H), 1.40 (s, 3H). ^{13}C NMR (101 MHz, $CDCl_3$): δ 206.9, 205.1, 171.5, 132.9, 132.7, 128.9, 128.1, 93.9, 90.2, 82.3, 60.3, 34.9, 28.0, 26.3, 19.0. IR (thin film, cm^{-1}): 2982, 2928, 1711, 1593, 1491, 1457, 1369, 1254, 1151, 1092, 1015, 844, 758. HRMS (ESI) calcd for $C_{19}H_{23}ClNaO_3^+$ (M+Na$^+$): 357.1228, found: 357.1228.

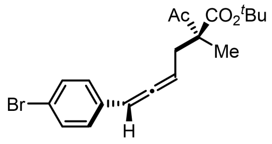

3i

3i. colorless oil, 63% yield, 13:1 *dr*, >99% *ee*. $[\alpha]_D^{20}$ = -9.4 (*c* = 4.3, $CHCl_3$). HPLC analysis: Daicel Chiralpak AD-H*2, 5% iso-propanol/hexane, flow rate = 1.0 mL/min, λ = 254 nm, retention time: 10.3 min (major), 13.0 min (minor). ^1H NMR (400 MHz, $CDCl_3$) δ 7.46 - 7.39 (m, 2H), 7.17 - 7.10 (m, 2H), 6.09 (dt, *J* = 6.4, 2.4 Hz, 1H), 5.43 (td, *J* = 7.9, 6.3 Hz, 1H), 2.64 (qdd, *J* = 14.3, 8.0, 2.4 Hz, 2H), 2.17 (s, 3H), 1.49 (s, 9H), 1.39 (s, 3H). ^{13}C NMR (101 MHz, $CDCl_3$) δ 206.9, 205.1, 171.4, 133.4, 131.8, 128.4, 120.7, 93.9, 90.2, 82.2, 60.2, 34.8, 28.0, 26.3, 19.0. IR (thin film, cm^{-1}): 2979, 2936, 1711, 1588,1457, 1369, 1255, 1217, 1151, 1070, 1012, 845, 755, 668. HRMS (ESI) calcd for $C_{19}H_{23}BrNaO_3^+$ (M+Na$^+$): 401.0723, found: 401.0721.

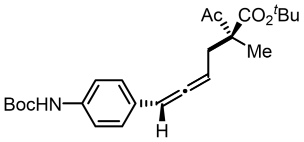

3j

3j. colorless oil, 61% yield, >20:1 *dr*, >99% *ee*. $[\alpha]_D^{20}$ = 36 (*c* = 4.3, $CHCl_3$). HPLC analysis: Daicel Chiralpak AD-H*2, 5% iso-propanol/hexane, flow rate = 1.0 mL/min, λ = 254 nm, retention time: 22.3 min (major), 32.4 min (minor). ^1H NMR (400 MHz, $CDCl_3$) δ 7.25 - 7.18 (m, 3H), 6.94 (dt, *J* = 7.2, 1.5 Hz, 1H), 6.52 (s, 1H), 6.09 (dt, *J* = 6.4, 2.4 Hz, 1H), 5.42 (dd, *J* = 8.0, 6.4 Hz, 1H), 2.69 - 2.56 (m, 2H), 2.15 (s, 3H), 1.52 (s, 9H), 1.47 (s, 9H), 1.39 (s, 3H). ^{13}C NMR (101 MHz, $CDCl_3$) δ 206.9, 205.4, 171.5, 152.8, 135.2, 129.3, 121.7, 94.6, 89.8, 82.2, 80.6, 60.4, 35.0, 28.5, 28.0, 26.3, 18.9. IR (thin film, cm^{-1}) 3359, 2976, 2927, 1704, 1606,1538, 1490, 1456, 1368, 1233, 1153, 844, 754, 667. HRMS (ESI) calcd for $C_{24}H_{33}NNaO_5^+$ (M+Na$^+$): 438.2251, found: 438.2253.

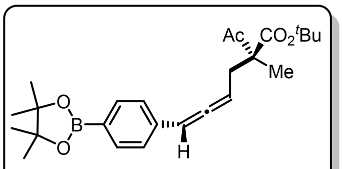

3k

3k. colorless oil, 81% yield, 19:1 *dr*, 99% *ee*. $[\alpha]_D^{20}$ = 14 (*c* = 4.5, $CHCl_3$). HPLC analysis: Daicel Chiralpak OJ-H*2, 1% iso-propanol/hexane, flow rate = 0.3 mL/min, λ = 254 nm, retention time: 32.7 min (major), 33.9 min (minor). ^1H NMR (400 MHz, $CDCl_3$) δ 7.76 - 7.71 (m, 2H), 7.27 - 7.23 (m, 2H), 6.13 (dt, *J* = 6.4, 2.4 Hz, 1H), 5.42 (td, *J* = 7.9, 6.3 Hz, 1H), 2.63 (qdd, *J* = 14.2, 7.9, 2.5 Hz, 2H), 2.15 (s, 3H), 1.47 (s, 8H), 1.38 (s, 3H), 1.34 (s, 12H). ^{13}C NMR (101 MHz, $CDCl_3$) δ 207.3, 205.2, 171.5, 137.3, 135.2, 126.2, 94.9, 89.8, 83.9, 82.2, 60.3, 34.9, 28.0, 26.3, 25.0, 19.0. IR (thin film, cm^{-1}) 2978, 2928, 1709, 1608, 1559, 1515, 1457, 1395, 1358, 1321, 1272, 1217, 1143, 1088, 1020, 963, 858, 755, 658, 578, 520. HRMS (ESI) calcd for $C_{25}H_{35}BNaO_5^+$ (M+Na$^+$): 449.2470, found: 449.2468.

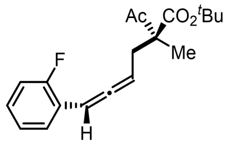

3l

3l. colorless oil, 84% yield, >20:1 *dr*, >99% *ee*. $[\alpha]_D^{20}$ = 40 (*c* = 2.2, $CHCl_3$). HPLC analysis: Daicel Chiralpak IC-H*2, 3% iso-propanol/hexane, flow rate = 0.5 mL/min, λ = 254 nm, retention time: 146.9 min (major), 153.6 min (minor). (The enantioselectivity is determined by its hydrazone derivative).^1H NMR (400 MHz, $CDCl_3$) δ 7.34 (td, *J* = 7.7, 1.9 Hz, 1H), 7.16 (tdd, *J* = 7.3, 5.1, 1.8 Hz, 1H), 7.08 (td, *J* = 7.5, 1.4 Hz, 1H), 7.01 (ddd, *J* = 10.6, 8.1, 1.3 Hz, 1H), 6.36 (dt, *J* = 6.5, 2.5 Hz, 1H), 5.43 (td, *J* = 8.0, 6.5 Hz, 1H), 2.64 (qdd, *J* = 14.3, 8.0, 2.5 Hz, 2H), 2.16 (s, 3H), 1.47 (s, 9H), 1.38 (s, 3H). ^{13}C NMR (101 MHz, $CDCl_3$) δ 207.4, 205.2, 171.5, 159.8 (d, *J* = 249.1 Hz), 128.4 (d, *J* = 11.1 Hz), 128.3, 124.3 (d, *J* = 3.6 Hz), 122.0 (d, *J* = 12.1 Hz), 115.7 (d, *J* = 21.4 Hz), 89.7, 87.1 (d, *J* = 6.7 Hz), 82.2, 60.3, 34.8, 28.0, 26.3, 19.0. ^{19}F NMR (376 MHz, $CDCl_3$) δ -119.5 (ddd, *J* = 10.3, 7.5, 4.9 Hz). IR (thin film, cm^{-1}) 3005, 2979, 2938, 2842, 1711, 1596, 1478, 1458, 1369, 1247, 1205, 1150, 1067, 942,821,683. HRMS (ESI) calcd for $C_{19}H_{23}FNaO_3^+$ (M+Na$^+$): 341.1523, found: 341.1527.

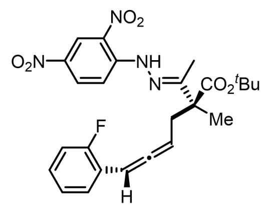

(rac)-3l-derivation

(rac)-3l-derivation. ^{1}H NMR (400 MHz, $CDCl_3$) δ 11.02 (d, *J* = 3.0 Hz, 1H), 9.14 (dd, *J* = 9.4, 2.6 Hz, 1H), 8.34 - 8.25 (m, 1H), 7.95 (d, *J* = 9.5 Hz, 1H), 7.25 (td, *J* = 7.7, 1.8 Hz, 1H), 7.11 (qdd, *J* = 7.3, 5.1, 1.7 Hz, 1H), 7.04 - 6.87 (m, 2H), 6.34 (ddd, *J* = 8.5, 5.6, 3.1 Hz, 1H), 5.53 (q, *J* = 7.4 Hz, 1H), 2.79 (dqdd, *J* = 29.6, 14.7, 7.6, 2.6 Hz, 2H), 2.05 (d, *J* = 13.3 Hz, 3H), 1.55 (d, *J* = 2.6 Hz, 3H), 1.50 (d, *J* = 3.0 Hz, 9H). ^{13}C NMR (101 MHz, $CDCl_3$) δ 207.5, 172.8, 160.9, 156.3 (d, *J* = 18.7 Hz), 145.3 (d, *J* = 4.0 Hz), 138.2, 130.1 (d, *J* = 3.7 Hz), 128.4 (d, *J* = 8.1 Hz), 128.2 (d, *J* = 3.3 Hz), 124.1, 123.5, 116.8 (d, *J* = 4.6 Hz), 115.8 (d, *J* = 9.3 Hz), 115.5 (d, *J* = 9.3 Hz), 89.9 (d, *J* = 33.7 Hz), 87.3 (d, *J* = 6.3 Hz), 82.2, 55.4 (d, *J* = 4.7 Hz), 35.4 (d, *J* = 11.0 Hz), 28.1 (d, *J* = 2.4 Hz), 21.1, 20.6, 14.4 (d, *J* = 12.7 Hz). ^{19}F NMR (376 MHz, $CDCl_3$) δ -119.0 (ddd, *J* = 10.6, 7.6, 5.2 Hz), -119.2 (ddd, J = 10.7, 7.3, 5.1 Hz).

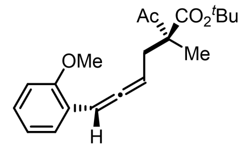

3m

3m. colorless oil, 57% yield, 15:1 *dr*, >99% *ee*. $[\alpha]_D^{20}$ = -17.5 (*c* = 3.2, $CHCl_3$). HPLC analysis: Daicel Chiralpak OJ-H, 5% iso-propanol/hexane, flow rate = 1.0 mL/min, λ = 254 nm, retention time: 8.6 min (major), 16.8 min (minor). ^{1}H NMR (400 MHz, $CDCl_3$) δ 7.32 (dd, *J* = 7.6, 1.7 Hz, 1H), 7.17 (td, *J* = 7.7, 1.8 Hz, 1H), 6.91 (td, *J* = 7.5, 1.1 Hz, 1H), 6.85 (dd, *J* = 8.3, 1.1 Hz, 1H), 6.53 (dt, *J* = 6.5, 2.5 Hz, 1H), 5.36 (td, *J* = 7.9, 6.5 Hz, 1H), 3.83 (s, 3H), 2.63 (qdd, *J* = 14.2, 7.9, 2.5 Hz, 2H), 2.15 (s, 3H), 1.47 (s, 9H), 1.38 (s, 3H). ^{13}C NMR (101 MHz, $CDCl_3$) δ 207.3, 205.4, 171.6, 156.1, 128.2, 128.0, 122.8, 120.9, 111.0, 88.8, 88.6, 82.1, 60.4, 55.7, 35.1, 28.0, 26.3, 19.0. IR (thin film, cm^{-1}) 2980, 2929, 1711, 1600, 1491, 1459, 1369, 1248, 1153, 1028, 846, 755, 667. HRMS (ESI) calcd for $C_{20}H_{26}NaO_4^+$ (M+Na$^+$): 353.1723, found: 353.1725.

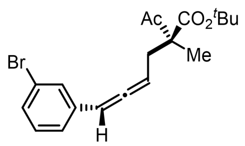

3n

3n. colorless oil, 65% yield, >20:1 *dr*. >99% *ee*. $[\alpha]_D^{20}$ = 28 (*c* = 3.6, $CHCl_3$). HPLC analysis: Daicel Chiralpak AD-H*2, 3% iso-propanol/hexane, flow rate = 0.5 mL/min, λ = 254 nm, retention time: 20.3 min (major), 22.2 min (minor). ^{1}H NMR (400 MHz, $CDCl_3$) δ 7.40 (q, *J* = 1.3 Hz, 1H), 7.31 (tq, *J* = 4.6, 1.9 Hz, 1H), 7.20 – 7.12 (m, 2H), 6.06 (dt, *J* = 6.4, 2.4 Hz, 1H), 5.46 (td, *J* = 7.9, 6.3 Hz, 1H), 2.71 – 2.56 (m, 2H), 2.17 (s, 3H), 1.47 (s, 9H), 1.39 (s, 3H). ^{13}C NMR (101 MHz, $CDCl_3$) δ 207.0, 205.1, 171.4, 136.7, 130.2, 130.0, 129.7, 125.5, 122.9, 93.7, 90.4, 82.3, 60.2, 34.8, 28.0, 26.3, 19.0. IR (thin film, cm^{-1}) 2955, 2925, 1709, 1568, 1457, 1369, 1251, 1218, 1150, 845, 756, 668. HRMS (ESI) calcd for $C_{19}H_{23}BrNaO_3^+$ (M+Na$^+$): 401.0723, found: 401.0722.

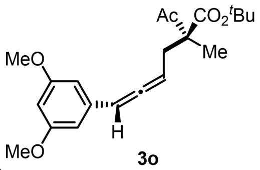

3o

3o. colorless oil, 67% yield, 17:1 *dr*, >99% *ee*. $[\alpha]_D^{20}$ = 61.6 (*c* = 3.5, $CHCl_3$). HPLC analysis: Daicel Chiralpak AD-H*2, 5% iso-propanol/hexane, flow rate = 1.0 mL/min, λ = 254 nm, retention time: 11.9 min (major), 13.8 min (minor). ^{1}H NMR (400 MHz, $CDCl_3$) δ 6.45 (d, *J* = 2.3 Hz, 2H), 6.34 (t, *J* = 2.3 Hz, 1H), 6.08 (dt, *J* = 6.4, 2.4 Hz, 1H), 5.43 (dt, *J* = 8.5, 7.2 Hz, 1H), 3.81 (s, 6H), 2.65 (qdd, *J* = 14.2, 8.0, 2.4 Hz, 2H), 2.17 (s, 3H), 1.48 (s, 9H), 1.41 (s, 3H). ^{13}C NMR (101 MHz, $CDCl_3$) δ 206.9, 205.1, 171.5, 161.1, 136.4, 104.8, 99.8, 94.9, 89.8, 82.2, 60.3, 55.5, 34.9, 28.0, 26.3, 18.9. IR (thin film, cm^{-1}) 2977, 2934, 1708, 1594, 1457, 1428, 1368, 1292, 1204, 1151, 1063, 928, 843, 752, 667, 540. HRMS (ESI) calcd for $C_{21}H_{28}NaO_5^+$ (M+Na$^+$): 383.1829, found: 383.1826.

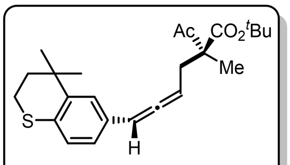

3p

3p. colorless oil, 45% yield, 18:1 *dr*, >99% *ee*. $[\alpha]_D^{20}$ = 109 (*c* = 2.8, $CHCl_3$). HPLC analysis: Daicel Chiralpak AD-H*2, 5% iso-propanol/hexane, flow rate = 1.0 mL/min, λ = 254 nm, retention time: 9.6 min (major), 11.3 min (minor). ^{1}H NMR (400 MHz, $CDCl_3$) δ 7.24 (d, *J* = 1.9 Hz, 1H), 7.02 (d, *J* = 8.1 Hz, 1H), 6.93 (dd, *J* = 8.1, 1.9 Hz, 1H), 6.06 (dt, *J* = 6.5, 2.4 Hz, 1H), 5.38 (dt, *J* = 8.6, 6.9 Hz, 1H), 3.05 - 2.99 (m, 2H), 2.72 - 2.53 (m, 2H), 2.16 (s, 3H), 1.97 - 1.92 (m, 2H), 1.47 (s, 9H), 1.38 (s, 3H), 1.33 (s, 6H). ^{13}C NMR (101 MHz, $CDCl_3$) δ 206.5, 205.2, 171.5, 142.4, 130.0, 127.0, 125.0, 124.7, 124.7, 94.7, 89.7, 82.2, 60.3, 37.8, 35.2, 33.2, 30.2, 28.0, 26.3, 23.3, 19.0. IR (thin film, cm^{-1}) 2966, 2926, 1709, 1457, 1369, 1215, 1150, 749, 667. HRMS (ESI) calcd for $C_{24}H_{32}NaO_3S^+$ (M+Na$^+$): 423.1964, found: 423.1962.

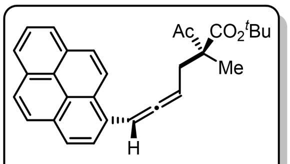

3q

3q. colorless oil, 76% yield, 9:1 *dr*, 96% *ee*. $[\alpha]_D^{20}$ = 19 (*c* = 3.2, $CHCl_3$). HPLC analysis: Daicel Chiralpak AS-H*2, 3% iso-propanol/hexane, flow rate = 0.5 mL/min, λ = 254 nm, retention time: 38.8 min (minor), 43.1 min (major). ^{1}H NMR (400 MHz, $CDCl_3$) δ 8.35 (d, *J* = 9.3 Hz, 1H), 8.16 (q, *J* = 1.2 Hz, 1H), 8.15 (d, *J* = 1.5 Hz, 1H), 8.10 (d, *J* = 3.9 Hz, 1H), 8.09 - 8.04 (m, 2H), 8.02 (s, 2H), 7.98 (t, *J* = 7.6 Hz, 1H), 7.15 (dt, *J* = 6.5, 2.4 Hz, 1H), 5.59 (td, *J* = 8.0, 6.4 Hz, 1H), 2.76 (qdd, *J* = 14.3, 7.9, 2.5 Hz, 2H), 2.17 (s, 3H), 1.49 (s, 9H), 1.45 (s, 3H). ^{13}C NMR (101 MHz, $CDCl_3$) δ 208.9, 205.3, 171.6, 131.6, 131.0, 130.5, 127.9, 127.8, 127.7, 127.6, 127.2, 126.1, 125.6, 125.4, 125.3, 125.2, 125.2, 125.1, 122.8, 91.8, 89.4, 82.2, 60.4, 35.1, 28.0, 26.4, 19.1. IR (thin film, cm^{-1}) 2978, 1709, 1458, 1370, 1278, 1217, 1147, 1030, 748, 667, 638, 542. HRMS (ESI) calcd for $C_{29}H_{28}NaO_3^+$ (M+Na$^+$): 447.1931, found: 447.1953.

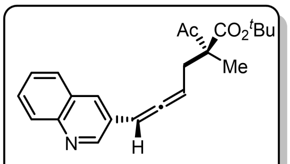

3r

3r. colorless oil, 57% yield, 9:1 *dr*, 99% *ee*. $[\alpha]_D^{20}$ = 141.1 (*c* = 0.9, $CHCl_3$). HPLC analysis: Daicel Chiralpak AD-H*2, 5% iso-propanol/hexane, flow rate = 1.0 mL/min, λ = 254 nm, retention time: 27.2 min (major), 38.1 min (minor). ^{1}H NMR (400 MHz, $CDCl_3$) δ 8.86 (d, *J* = 2.1 Hz, 1H), 8.07 (d, *J* = 8.4 Hz, 1H), 7.94 (d, *J* = 2.1 Hz, 1H), 7.78 (dd, *J* = 8.1, 1.3 Hz, 1H), 7.66 (ddd, *J* = 8.4, 6.9, 1.5 Hz, 1H), 7.53 (ddd, *J* = 8.0, 6.8, 1.2 Hz, 1H), 6.30 (dt, *J* = 6.5, 2.5 Hz, 1H), 5.57 (td, *J* = 7.8, 6.2 Hz, 1H), 2.69 (qdd, *J* = 14.3, 8.0, 2.5 Hz, 2H), 2.18 (s, 3H), 1.48 (s, 9H), 1.43 (s, 3H). ^{13}C NMR (101 MHz, $CDCl_3$) δ 207.5, 205.1, 171.4, 149.9, 132.5, 129.4, 129.3, 129.0, 128.3, 128.2, 127.7, 127.19, 92.0, 90.8, 82.3, 60.2, 34.8, 27.9, 26.3, 19.1. IR (thin film, cm^{-1}) 2926, 1710, 1458, 1369, 1219, 1152, 845, 771, 667. HRMS (ESI) calcd for $C_{22}H_{25}NNaO_3^+$ (M+Na$^+$): 374.1727, found: 374.1724.

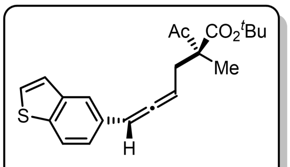

3s

3s. colorless oil, 61% yield, >20:1 *dr*, 98% *ee*. $[\alpha]_D^{20}$ = 23.8 (*c* = 3.9, $CHCl_3$). HPLC analysis: Daicel Chiralpak AS-H, 5% iso-propanol/hexane, flow rate = 1.0 mL/min, λ = 254 nm, retention time: 5.1 min (minor), 5.6 min (major). ^{1}H NMR (400 MHz, $CDCl_3$) δ 7.62 (d, *J* = 2.2 Hz, 1H), 7.48 (d, *J* = 1.8 Hz, 1H), 7.45 (dt, *J* = 8.5, 0.8 Hz, 1H), 7.25 (dd, *J* = 8.6, 1.8 Hz, 1H), 6.75 (dd, *J* = 2.2, 0.9 Hz, 1H), 6.25 (dt, *J* = 6.4, 2.4 Hz, 1H), 5.45 (td, *J* = 7.9, 6.3 Hz, 1H), 2.74 - 2.57 (m, 2H), 2.19 (s, 3H), 1.50 (s, 9H), 1.43 (s, 3H). ^{13}C NMR (101 MHz, $CDCl_3$) δ 206.5, 205.3, 171.6, 154.4, 145.5, 129.1, 128.0, 123.5, 119.4, 111.6, 106.7, 94.8, 89.7, 82.2, 60.3, 35.2, 28.0, 26.3, 19.0. IR (thin film, cm^{-1}) 2927, 1708, 1458, 1369, 1254, 1219, 1151, 1031, 844, 771, 667. HRMS (ESI) calcd for $C_{21}H_{24}NaO_3S^+$ (M+Na$^+$): 379.1338, found: 379.1347.

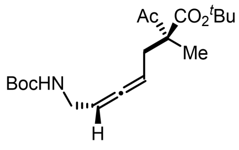

3t

3t. colorless oil, 57% yield, >20:1 *dr*, 99% *ee*. $[\alpha]_D^{20}$ = 54.3 (*c* = 1.4, $CHCl_3$). HPLC analysis: Daicel Chiralpak OJ-H*2, 5% iso-propanol/hexane, flow rate = 1.0 mL/min, λ = 254 nm, retention time: 25.4 min (major), 36.7 min (minor). ^{1}H NMR (400 MHz, $CDCl_3$) δ 5.16 (t, *J* = 7.9 Hz, 1H), 5.11 (dtt, *J* = 9.4, 6.3, 3.1 Hz, 1H), 4.71 (s, 1H), 3.69 (q, *J* = 5.3 Hz, 2H), 2.58 - 2.41 (m, 2H), 2.16 (s, 3H), 1.46 (s, 9H), 1.45 (s, 9H), 1.33 (s, 3H). ^{13}C NMR (101 MHz, $CDCl_3$) δ 205.4, 205.1, 171.5, 155.8, 89.4, 88.7, 88.5, 82.2, 60.3, 39.2, 34.7, 28.5, 28.0, 26.4, 18.9. IR (thin film, cm^{-1}) 3391, 2978, 2927, 1708, 1507, 1457, 1368, 1249, 1219, 1162, 1115, 845, 772, 667. HRMS (ESI) calcd for $C_{19}H_{31}NaO_5^+$ (M+Na$^+$): 376.2094, found: 376.2093.

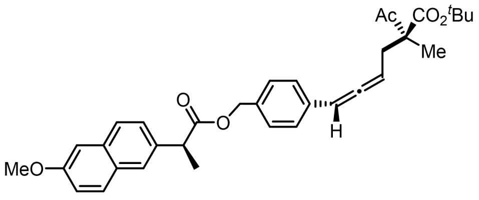

3aa

3aa. White solid, 47% yield, 10:1 *dr*. $[\alpha]_D^{20}$ = 17.8 (*c* = 2.7, $CHCl_3$). ^{1}H NMR (400 MHz, $CDCl_3$) δ 7.71 - 7.62 (m, 3H), 7.39 (dd, *J* = 8.5, 1.9 Hz, 1H), 7.23 - 7.07 (m, 6H), 6.09 (dt, *J* = 6.4, 2.5 Hz, 1H), 5.40 (q, *J* = 7.6 Hz, 1H), 5.15 – 4.99 (m, 2H), 3.92 (s, 4H), 2.70 - 2.53 (m, 2H), 2.16 (d, *J* = 8.7 Hz, 3H), 1.58 (d, *J* = 7.1 Hz, 3H), 1.55 (s, 4H), 1.47 (s, 5H), 1.46 (s, 1H), 1.37 (s, 2H). ^{13}C NMR (101 MHz, $CDCl_3$) δ 207.0, 205.2, 174.6, 171.5, 157.8, 135.7, 134.8, 133.7, 129.6, 129.3, 129.1, 128.5, 127.3, 127.0, 126.3, 126.4, 119.1, 105.7, 94.4, 90.0, 82.2, 66.4, 60.2, 55.4, 45.6, 34.8, 28.0, 26.4, 19.2, 18.7. IR (thin film, cm^{-1}) 2925, 1733, 1716, 1607, 1504, 1457, 1376, 1267, 1217, 1157, 856, 776, 668. HRMS (ESI) calcd for $C_{34}H_{38}NaO_6^+$ (M+Na$^+$): 565.2561, found: 565.2567.

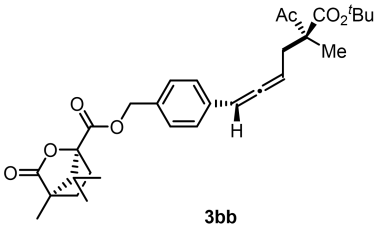

3bb

3bb. White solid, 57% yield, >20:1 *dr*. ^{1}H NMR (400 MHz, $CDCl_3$) δ 7.31 (d, *J* = 8.3 Hz, 2H), 7.25 (d, *J* = 6.3 Hz, 2H), 6.12 (dt, *J* = 6.4, 2.4 Hz, 1H), 5.43 (q, *J* = 7.5 Hz, 1H), 5.23 (s, 2H), 2.63 (qdd, *J* = 14.2, 7.9, 2.4 Hz, 2H), 2.43 (ddd, *J* = 13.4, 10.7, 4.2 Hz, 1H), 2.16 (s, 3H), 2.04 (ddd, *J* = 13.7, 9.4, 4.6 Hz, 1H), 1.91 (ddd, *J* = 13.1, 10.8, 4.5 Hz, 1H), 1.68 (ddd, *J* = 13.4, 9.4, 4.3 Hz, 1H), 1.47 (s, 9H), 1.38 (s, 3H), 1.10 (s, 3H), 1.01 (s, 3H), 0.90 (s, 3H). ^{13}C NMR (101 MHz, $CDCl_3$) δ 207.1, 205.2, 202.9, 197.0, 171.5, 134.9, 133.9, 129.0, 127.1, 94.3, 90.0, 82.3, 67.1, 60.3, 54.9, 54.4, 34.9, 30.8, 29.1, 28.0, 26.3, 19.0, 16.9, 16.9, 9.8. IR (thin film, cm^{-1}) 2980, 2952, 2929, 1733, 1715, 1607, 1504, 1453, 1372, 1217, 1157, 855, 763, 665. HRMS (ESI) calcd for $C_{30}H_{39}O_7^+$ (M+H$^+$): 511.2690, found: 511.2697.

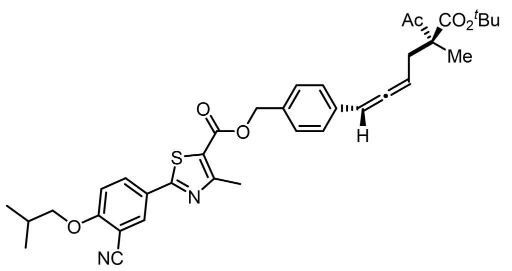

3cc

3cc. White solid, 42% yield, 4:1 *dr,* >99% *ee.* $[\alpha]_D^{20}$ = -17.2 (*c* = 13.1, $CHCl_3$). HPLC analysis: Daicel Chiralpak AD-H*2, 5% iso-propanol/hexane, flow rate = 1.0 mL/min, λ = 254 nm, retention time: 25.4 min (major), 36.7 min (minor). ^1H NMR (400 MHz, $CDCl_3$) δ 8.17 (d, *J* = 2.3 Hz, 1H), 8.08 (dd, *J* = 8.9, 2.3 Hz, 1H), 7.37 (d, *J* = 8.2 Hz, 2H), 7.32 - 7.24 (m, 2H), 7.00 (d, *J* = 8.9 Hz, 1H), 6.14 (dt, *J* = 6.5, 2.4 Hz, 1H), 5.48 – 5.38 (m, 1H), 5.30 (s, 2H), 3.90 (d, *J* = 6.5 Hz, 2H), 2.76 (s, 3H), 2.64 (qdd, *J* = 14.2, 7.9, 2.4 Hz, 2H), 2.26 - 2.18 (m, 1H), 2.16 (s, 3H), 1.47 (s, 9H), 1.39 (s, 3H), 1.09 (d, *J* = 6.7 Hz, 6H). ^{13}C NMR (101 MHz, $CDCl_3$) δ 207.1, 205.1, 171.5, 167.5, 162.6, 161.9, 161.7, 134.8, 134.3, 132.7, 132.2, 128.8, 127.2, 127.1, 126.1, 121.7, 115.5, 112.7, 103.1, 94.3, 90.1, 82.2, 75.8, 66.9, 60.2, 34.8, 28.3, 28.0, 26.4, 19.2, 19.0, 17.7. IR (thin film, cm^{-1}) 2976, 2929, 2250, 1731, 1715, 1607, 1580, 1502, 1451, 1371, 1215, 1157, 855, 763, 665. HRMS (ESI) calcd for $C_{36}H_{40}N_2NaO_6S^+$ ($M+Na^+$): 651.2499, found: 651.2478.

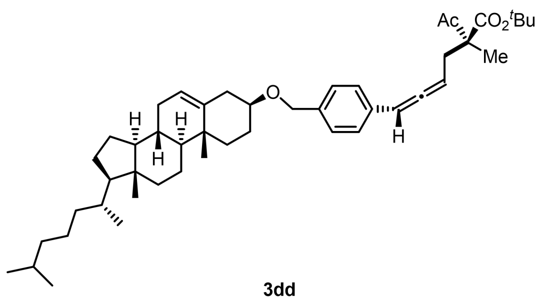

3dd

3dd. White solid, 53% yield, >20:1 *dr.* ^1H NMR (400 MHz, $CDCl_3$) δ 7.30 – 7.26 (m, 2H), 7.22 (dd, *J* = 8.2, 1.8 Hz, 2H), 6.12 (dt, *J* = 6.4, 2.4 Hz, 1H), 5.40 (q, *J* = 7.4, 6.6 Hz, 1H), 5.33 (dd, *J* = 5.2, 2.6 Hz, 1H), 4.53 (s, 2H), 3.30 - 3.19 (m, 1H), 2.63 (dtdd, *J* = 16.7, 14.2, 7.8, 2.5 Hz, 2H), 2.40 (ddd, *J* = 13.3, 4.7, 2.2 Hz, 1H), 2.31 - 2.24 (m, 1H), 2.17 (d, *J* = 8.6 Hz, 3H), 2.04 - 1.77 (m, 6H), 1.46 (s, 18H), 1.38 (s, 3H), 1.20 - 1.03 (m, 8H), 1.01 (s, 5H), 0.91 (d, *J* = 6.5 Hz, 4H), 0.86 (dd, *J* = 6.6, 1.8 Hz, 6H), 0.67 (s, 3H). ^{13}C NMR (101 MHz, $CDCl_3$) δ 206.9, 205.2, 171.6, 141.1, 138.1, 133.5, 128.0, 126.9, 121.7, 94.6, 89.8, 82.2, 78.6, 69.8, 60.2, 56.9, 56.3, 50.4, 42.5, 40.0, 39.7, 39.3, 37.4, 37.1, 36.4, 35.9, 34.8, 32.1, 32.1, 28.6, 28.4, 28.2, 28.0, 26.4, 24.4, 24.0, 23.0, 22.7, 21.2, 19.5, 19.2, 12.0. IR (thin film, cm^{-1}) 3054, 2982, 2953, 2927, 1731, 1603, 1504, 1453, 1376, 1263, 1157, 855, 763, 668. HRMS (ESI) calcd for $C_{47}H_{70}O_4^+$ ($M+Na^+$): 721.5166, found: 721.5183.

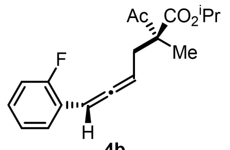

4b

4b. colorless oil, 81% yield, >20:1 *dr*, 99% *ee*. $[\alpha]_D^{20}$ = 15.2 (*c* = 5.1, $CHCl_3$). HPLC analysis: Daicel Chiralpak OJ-H*2, 1% iso-propanol/hexane, flow rate = 0.3 mL/min, λ = 254 nm, retention time: 94.0 min (major), 106.1 min (minor). ^1H NMR (400 MHz, $CDCl_3$) δ 7.33 (td, *J* = 7.7, 1.9 Hz, 1H), 7.16 (tdd, *J* = 7.3, 5.1, 1.9 Hz, 1H), 7.08 (td, *J* = 7.5, 1.4 Hz, 1H), 7.00 (ddd, *J* = 10.5, 8.1, 1.3 Hz, 1H), 6.36 (dt, *J* = 6.5, 2.4 Hz, 1H), 5.44 (q, *J* = 7.5 Hz, 1H), 5.07 (hept, *J* = 6.3 Hz, 1H), 2.76 - 2.56 (m, 2H), 2.16 (s, 3H), 1.42 (s, 3H), 1.25 (dd, *J* = 6.3, 3.5 Hz, 6H). ^{13}C NMR (101 MHz, $CDCl_3$) δ 207.4, 205.0, 171.9, 159.8 (d, *J* = 249.1 Hz), 128.5 (d, *J* = 8.6 Hz), 128.4 (d, *J* = 4.0 Hz), 124.3 (d, *J* = 3.5 Hz), 121.9 (d, *J* = 11.9 Hz), 115.7 (d, *J* = 21.4 Hz), 89.7, 87.2 (d, *J* = 6.6 Hz), 69.3, 59.8, 34.7, 26.3, 21.7 (d, *J* = 6.5 Hz), 19.0. ^{19}F NMR (376 MHz, $CDCl_3$) δ -119.4 (ddd, *J* = 10.4, 7.6, 5.1 Hz). IR (thin film, cm^{-1}) 2982, 2934, 1709, 1613, 1491, 1456, 1376, 1220, 1097, 937, 771. HRMS (ESI) calcd for $C_{18}H_{21}FNaO_3^+$ ($M+Na^+$): 327.1367, found: 327.1368.

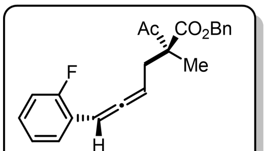

4c

4c. colorless oil, 78% yield, >20:1 *dr*, >99% *ee*. $[\alpha]_D^{20}$ = 7.2 (*c* = 5.7, $CHCl_3$). HPLC analysis: Daicel Chiralpak IC-H*2, 3% iso-propanol/hexane, flow rate = 0.5 mL/min, λ = 254 nm, retention time: 48.2 min (major), 56.5 min (minor). ^{1}H NMR (400 MHz, $CDCl_3$) δ 7.37 - 7.27 (m, 6H), 7.15 (tdd, *J* = 7.3, 5.1, 1.9 Hz, 1H), 7.06 (td, *J* = 7.5, 1.3 Hz, 1H), 7.00 (ddd, *J* = 10.5, 8.1, 1.3 Hz, 1H), 6.32 (dt, *J* = 6.6, 2.5 Hz, 1H), 5.42 (q, *J* = 7.4 Hz, 1H), 5.16 (s, 2H), 2.68 (dddd, *J* = 52.7, 14.3, 7.9, 2.5 Hz, 2H), 2.08 (s, 3H), 1.45 (s, 3H). ^{13}C NMR (101 MHz, $CDCl_3$) δ 207.4, 204.8, 172.2, 159.8 (d, *J* = 249.6 Hz), 135.3, 128.9, 128.7 (d, *J* = 12.5 Hz), 128.6, 128.5, 128.4 (d, *J* = 3.5 Hz), 124.1 (d, *J* = 3.4 Hz), 121.9 (d, *J* = 12.1 Hz), 115.7 (d, *J* = 21.4 Hz), 89.5, 87.2 (d, *J* = 6.7 Hz), 67.4, 59.9, 34.8, 26.3, 19.1. ^{19}F NMR (376 MHz, $CDCl_3$) δ -119.3 (ddd, *J* = 10.3, 7.6, 5.1 Hz). IR (thin film, cm^{-1}) 3030, 2926, 1712, 1490, 1456, 1378, 1217, 1100, 750, 697, 667. HRMS (ESI) calcd for $C_{22}H_{21}FNaO_3^+$ (M+Na$^+$): 375.1367, found: 375.1370.

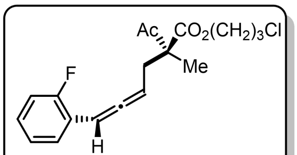

4d

4d. colorless oil, 94% yield, 10:1 *dr*, >99% *ee*. $[\alpha]_D^{20}$ = -6.4 (*c* = 7.0, $CHCl_3$). HPLC analysis: Daicel Chiralpak OJ-H, 5% iso-propanol/hexane, flow rate = 1.0 mL/min, λ = 254 nm, retention time: 18.7 min (major), 24.3 min (minor). ^{1}H NMR (400 MHz, $CDCl_3$) δ 7.32 (td, *J* = 7.7, 1.9 Hz, 1H), 7.17 (tdd, *J* = 7.3, 5.1, 1.9 Hz, 1H), 7.08 (td, *J* = 7.5, 1.3 Hz, 1H), 7.01 (ddd, *J* = 10.5, 8.2, 1.4 Hz, 1H), 6.37 (dt, *J* = 6.5, 2.5 Hz, 1H), 5.45 (q, *J* = 7.4 Hz, 1H), 4.29 (t, *J* = 6.1 Hz, 2H), 3.57 (t, *J* = 6.3 Hz, 2H), 2.68 (dddd, *J* = 46.9, 14.3, 7.8, 2.5 Hz, 2H), 2.17 (s, 3H), 2.09 (p, *J* = 6.2 Hz, 2H), 1.45 (s, 3H). ^{13}C NMR (101 MHz, $CDCl_3$) δ 207.4, 204.8, 172.2, 159.8 (d, *J* = 249.4 Hz), 128.6 (d, *J* = 8.0 Hz), 128.4 (d, *J* = 3.4 Hz), 124.3 (d, *J* = 3.6 Hz), 121.8 (d, *J* = 12.0 Hz), 115.8 (d, *J* = 21.4 Hz), 89.5, 87.4 (d, *J* = 6.6 Hz), 62.3, 59.9, 41.1, 34.8, 31.4, 26.3, 19.1. ^{19}F NMR (376 MHz, $CDCl_3$) δ -119.2 (ddd, *J* = 10.7, 7.8, 5.3 Hz). IR (thin film, cm^{-1}) 2962, 2926, 1714, 1613, 1490, 1456, 1358,1220,1103, 772, 656. HRMS (ESI) calcd for $C_{18}H_{20}ClFNaO_3^+$ (M+Na$^+$): 361.0977, found: 361.0983.

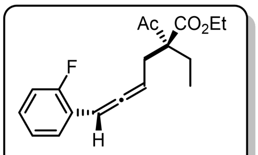

4e

4e. colorless oil, 64% yield, 11:1 *dr*, >99% *ee*. $[\alpha]_D^{20}$ = 78 (*c* = 1.3, $CHCl_3$). HPLC analysis: Daicel Chiralpak OJ-H*2, 5% iso-propanol/hexane, flow rate = 1.0 mL/min, λ = 254 nm, retention time: 25.5 min (major), 31.8 min (minor). ^{1}H NMR (400 MHz, $CDCl_3$) δ 7.31 (td, *J* = 7.7, 1.9 Hz, 1H), 7.16 (tdd, *J* = 7.3, 5.0, 1.8 Hz, 1H), 7.08 (td, *J* = 7.5, 1.4 Hz, 1H), 7.00 (ddd, *J* = 9.7, 8.1, 1.3 Hz, 1H), 6.35 (dt, *J* = 6.5, 2.5 Hz, 1H), 5.36 (q, *J* = 7.5 Hz, 1H), 4.19 (q, 2H), 2.76 - 2.63 (m, 2H), 2.14 (s, 3H), 2.02 (ddq, *J* = 29.5, 14.8, 7.4 Hz, 2H), 1.26 (t, *J* = 7.1 Hz, 3H), 0.80 (t, *J* = 7.6 Hz, 3H). ^{13}C NMR (101 MHz, $CDCl_3$) δ 207.2, 204.7, 171.9, 159.7 (d, *J* = 249.1 Hz), 128.5 (d, *J* = 8.3 Hz), 128.3 (d, *J* = 3.4 Hz), 124.3 (d, *J* = 2.9 Hz), 121.9 (d, *J* = 12.2 Hz), 115.7 (d, *J* = 21.5 Hz), 89.4, 87.2 (d, *J* = 6.6 Hz), 64.0, 61.6, 30.7, 26.9, 24.5, 14.2, 8.3. ^{19}F NMR (376 MHz, $CDCl_3$) δ -119.5 (ddd, *J* = 10.3, 7.5, 4.9 Hz). IR (thin film, cm^{-1}) 2966, 2927, 1711, 1489, 1457, 1219,1100, 1033, 771, 669. HRMS (ESI) calcd for $C_{18}H_{21}FNaO_3^+$ (M+Na$^+$): 327.1367, found: 327.1368.

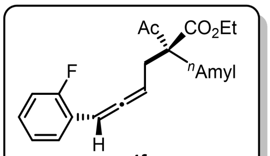

4f

4f. colorless oil, 47% yield, >12:1 *dr*, >99% *ee*. $[\alpha]_D^{20}$ = 158 (*c* = 2.7, $CHCl_3$). HPLC analysis: Daicel Chiralpak OJ-H*3, 3% iso-propanol/hexane, flow rate = 0.5 mL/min, λ = 254 nm, retention time: 25.6 min (major), 32.5 min (minor). ^{1}H NMR (400 MHz, $CDCl_3$) δ 7.31 (td, *J* = 7.7, 1.9 Hz, 1H), 7.16 (tdd, *J* = 7.4, 5.1, 1.9 Hz, 1H), 7.07 (td, *J* = 7.5, 1.3 Hz, 1H), 7.00 (ddd, *J* = 9.7, 8.1, 1.3 Hz, 1H), 6.35 (dt, *J* = 6.6, 2.4 Hz, 1H), 5.36 (q, *J* = 7.5 Hz, 1H), 4.20 (q, *J* = 7.1 Hz, 2H), 2.77 - 2.61 (m, 2H), 2.13 (s, 3H), 2.04 - 1.82 (m, 2H), 1.30 - 1.20 (m, 7H), 1.08 (ddq, *J* = 17.9, 12.8, 5.8 Hz, 2H), 0.86 - 0.79 (t, 3H). ^{13}C NMR (101 MHz, $CDCl_3$) δ 207.3, 204.7, 172.0,159.7 (d, *J* = 249.2 Hz), 128.3 (d, *J* = 8.1 Hz), 128.2 (d, *J* = 3.4 Hz), 124.1 (d, *J* = 3.6 Hz), 121.7, 115.6 (d, *J* = 21.4 Hz), 89.3, 87.1 (d, *J* = 6.6 Hz), 62.4 (d, *J* = 207.2 Hz), 60.6 (d, *J* = 128.2 Hz), 31.9 (d, *J* = 62.2 Hz), 31.4 (d, *J* = 24.5 Hz), 28.6 (d, *J* = 52.0 Hz), 27.0 (d, *J* = 41.0 Hz), 23.5, 22.5, 14.2 (d, *J* = 3.2 Hz), 14.1 (d, *J* = 2.1 Hz). ^{19}F NMR (376 MHz, $CDCl_3$) δ -119.5 (ddd, *J* = 10.8, 7.3,

5.2 Hz). IR (thin film, cm^{-1}) 2963, 2927, 1713, 1490, 1457, 1219, 1100, 756, 693, 663. HRMS (ESI) calcd for $C_{21}H_{27}FNaO_3^+$ (M+Na$^+$) 369.1836, found 369.1847.

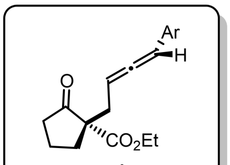

4g

4g. colorless oil, 23% yield, 14:1 *dr*, 93% *ee*. $[\alpha]_D^{20}$ = -9.8 (*c* = 0.2, $CHCl_3$). HPLC analysis: Daicel Chiralpak OJ-H*2, 3% iso-propanol/hexane, flow rate = 0.5 mL/min, λ = 254 nm, retention time: 98.8 min (minor), 109.6 min (major). ^{1}H NMR (400 MHz, $CDCl_3$) δ 7.34 (td, *J* = 7.6, 1.8 Hz, 1H), 7.16 (tdd, *J* = 7.3, 5.0, 1.8 Hz, 1H), 7.08 (td, *J* = 7.5, 1.3 Hz, 1H), 7.01 (ddd, *J* = 9.7, 8.1, 1.3 Hz, 1H), 6.37 (dt, *J* = 6.5, 2.7 Hz, 1H), 5.51 (p, *J* = 7.2 Hz, 1H), 4.14 (qdd, *J* = 7.1, 4.5, 2.8 Hz, 2H), 2.78 (ddt, *J* = 14.5, 7.5, 2.7 Hz, 1H), 2.60 - 2.50 (m, 1H), 2.49 - 2.38 (m, 2H), 2.28 (dt, *J* = 18.8, 8.2 Hz, 1H), 2.14 - 2.01 (m, 2H), 1.99 - 1.89 (m, 1H), 1.24 (t, *J* = 7.1 Hz, 3H). ^{13}C NMR (101 MHz, $CDCl_3$) δ 214.4, 207.5, 170.7, 159.8 (d, *J* = 249.3 Hz), 128.5, 128.4, 124.3 (d, *J* = 3.4 Hz), 121.9 (d, *J* = 11.9 Hz), 115.7 (d, *J* = 21.3 Hz), 90.2, 87.5, 61.7, 60.2, 38.2, 33.1, 32.8, 19.7, 14.2. ^{19}F NMR (376 MHz, $CDCl_3$) δ -119.3 (ddd, *J* = 10.6, 7.8, 5.2 Hz). IR (thin film, cm^{-1}) 2954, 2926, 1718, 1613, 1490, 1456, 1368, 1220, 1157, 1115, 1097, 1030, 771, 520. HRMS (ESI) calcd for $C_{18}H_{19}FNaO_3^+$ (M+Na$^+$): 325.1210, found: 325.1211.

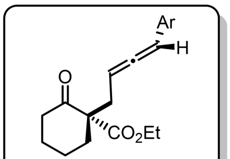

4h

4h. colorless oil, 37% yield, 14:1 *dr*, >99% *ee*. $[\alpha]_D^{20}$ = -14.5 (*c* = 3.8, $CHCl_3$). HPLC analysis: Daicel Chiralpak OJ-H, 5% iso-propanol/hexane, flow rate = 1.0 mL/min, λ = 254 nm, retention time: 8.0 min (minor), 10.5 min (major). ^{1}H NMR (400 MHz, $CDCl_3$) δ 7.34 (td, *J* = 7.7, 1.9 Hz, 1H), 7.15 (tdd, *J* = 7.3, 5.1, 1.9 Hz, 1H), 7.07 (td, *J* = 7.5, 1.3 Hz, 1H), 7.00 (ddd, *J* = 10.5, 8.1, 1.3 Hz, 1H), 6.33 (dt, *J* = 6.6, 2.4 Hz, 1H), 5.52 (q, *J* = 7.5 Hz, 1H), 4.19 (qd, *J* = 7.1, 2.5 Hz, 2H), 2.67 (ddd, *J* = 14.1, 7.6, 2.8 Hz, 1H), 2.60 - 2.44 (m, 4H), 2.02 (ddd, *J* = 12.2, 5.5, 3.1 Hz, 1H), 1.82 - 1.60 (m, 4H), 1.25 (t, *J* = 7.1 Hz, 3H). ^{13}C NMR (101 MHz, $CDCl_3$) δ 207.6, 207.5, 171.5, 159.7 (d, *J* = 249.2), 128.4 (d, *J* = 3.4), 128.3 (d, *J* = 8.3), 124.2 (d, *J* = 3.5), 122.1 (d, *J* = 12.3), 115.7 (d, *J* = 21.1), 90.1, 86.9 (d, *J* = 6.6), 61.6, 61.0, 41.1, 35.7, 34.6, 27.5, 22.5, 14.3. ^{19}F NMR (376 MHz, $CDCl_3$) δ -119.6 (ddd, *J* = 10.7, 8.0, 5.3 Hz). IR (thin film, cm^{-1}) 2955, 2927, 2867, 1712, 1489, 1456, 1373, 1219, 1094, 1024, 771. HRMS (ESI) calcd for $C_{19}H_{21}FNaO_3^+$ (M+Na$^+$): 339.1367, found: 339,1367.

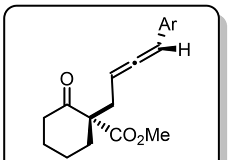

4i

4i. colorless oil, 47% yield, >20:1 *dr*, >99% *ee*. $[\alpha]_D^{20}$ = -12.8 (*c* = 2.3, $CHCl_3$). HPLC analysis: Daicel Chiralpak OJ-H, 5% iso-propanol/hexane, flow rate = 1.0 mL/min, λ = 254 nm, retention time: 11.2 min (minor), 13.8 min (major). ^{1}H NMR (400 MHz, $CDCl_3$) δ 7.3 (td, *J* = 7.7, 1.9 Hz, 1H), 7.2 (tdd, *J* = 7.3, 5.1, 1.9 Hz, 1H), 7.1 (td, *J* = 7.5, 1.4 Hz, 1H), 7.0 (ddd, *J* = 10.4, 8.1, 1.3 Hz, 1H), 6.3 (dt, *J* = 6.5, 2.4 Hz, 1H), 5.5 (q, *J* = 7.5 Hz, 1H), 3.7 (s, 3H), 2.7 (ddd, *J* = 14.1, 7.5, 2.8 Hz, 1H), 2.6 - 2.4 (m, 4H), 2.2 - 1.9 (m, 1H), 1.8 - 1.6 (m, 4H). ^{13}C NMR (101 MHz, $CDCl_3$) δ 207.4, 207.3, 172.1, 161.0, 158.5, 129.4 - 127.8 (m), 124.2 (d, *J* = 3.5 Hz), 122.1 (d, *J* = 12.1 Hz), 115.7 (d, *J* = 21.5 Hz), 90.1, 86.9 (d, *J* = 6.5 Hz), 61.1, 52.6, 41.1, 35.8, 34.6, 27.5, 22.5. ^{19}F NMR (376 MHz, $CDCl_3$) δ -119.5 (ddd, *J* = 10.4, 7.7, 5.2 Hz). IR (thin film, cm^{-1}) 2951, 2930, 2867, 1712, 1489, 1455, 1218, 1094, 770, 758, 668. HRMS (ESI) calcd for $C_{18}H_{19}FNaO_3^+$ (M+Na$^+$): 325.1210, found: 325.1210.

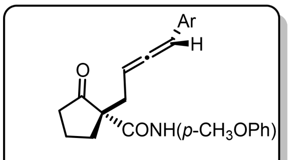

4j

4j. colorless oil, 67% yield, 5:1 *dr*, 92% *ee*. $[\alpha]_D^{20}$ = 19.4 (*c* = 1.9, $CHCl_3$). HPLC analysis: Daicel Chiralpak OD-H, 5% iso-propanol/hexane, flow rate = 1.0 mL/min, λ = 254 nm, retention time: 21.7 min (major), 25.3 min (minor). ^{1}H NMR (400 MHz, $CDCl_3$) δ 8.50 (s, 1H), 7.44 (dd, *J* = 9.0, 5.5 Hz, 2H), 7.31 (td, *J* = 7.9, 1.9 Hz, 1H), 7.18 - 7.08 (m, 1H), 7.05 - 6.93 (m, 2H), 6.90 - 6.81 (m, 2H), 6.36 (dt, *J* = 6.4, 2.3 Hz, 1H), 5.48 (q, *J* = 7.2 Hz, 1H), 3.80 (s, 3H), 2.80 (dt, *J* = 13.9, 7.1 Hz, 1H), 2.67 (ddd, *J* = 14.1, 7.2, 2.8 Hz,

1H), 2.61 - 2.51 (m, 1H), 2.44 (t, *J* = 7.7 Hz, 2H), 2.15 – 2.05 (m, 1H), 1.94 (p, *J* = 7.3 Hz, 2H). ^{13}C NMR (101 MHz, $CDCl_3$) δ 207.6, 207.6, 166.4, 156.6, 131.0, 128.5 (d, *J* = 15.0 Hz), 124.3 (d, *J* = 3.7 Hz), 121.7, 121.6, 115.8, 115.6, 114.3, 114.3, 89.2, 87.7 (d, *J* = 6.6 Hz), 61.0, 55.7, 39.2, 37.3, 31.4, 18.9. ^{19}F NMR (376 MHz, $CDCl_3$) δ -119.2 (ddd, *J* = 10.6, 7.6, 5.2 Hz). IR (thin film, cm^{-1}) 3341. 2959. 2926, 1724, 1683, 1512, 1457, 1217, 1035, 771, 668. HRMS (ESI) calcd for $C_{23}H_{22}FNaO_3^+$ (M+Na$^+$): 402.1476, found: 402.1479.

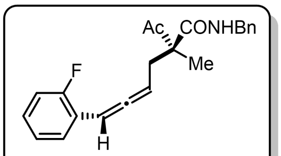

4k

4k. colorless oil, 53% yield, 20:1 *dr*, >99% *ee*. $[\alpha]_D^{20}$ = 33.2 (*c* = 3.6, $CHCl_3$). HPLC analysis: Daicel Chiralpak OD-H, 5% iso-propanol/hexane, flow rate = 1.0 mL/min, λ = 254 nm, retention time: 23.5 min (minor), 27.5 min (major). ^{1}H NMR (400 MHz, $CDCl_3$) δ 7.36 - 7.21 (m, 6H), 7.16 (tdd, *J* = 7.3, 5.1, 1.8 Hz, 1H), 7.10 - 7.03 (m, 1H), 7.00 (ddd, *J* = 10.6, 8.2, 1.3 Hz, 1H), 6.45 (t, *J* = 5.8 Hz, 1H), 6.31 (dt, *J* = 6.5, 2.5 Hz, 1H), 5.44 (q, *J* = 7.3 Hz, 1H), 4.50 - 4.35 (m, 2H), 2.78 - 2.66 (m, 2H), 2.21 (s, 3H), 1.49 (s, 3H). ^{13}C NMR (101 MHz, $CDCl_3$) δ 208.8, 207.3, 170.6, 159.8 (d, *J* = 249.5 Hz), 138.0, 128.9, 128.6 (d, *J* = 8.1 Hz), 128.4 (d, *J* = 3.5 Hz), 127.8, 127.8, 124.3 (d, *J* = 3.5 Hz), 121.8 (d, *J* = 11.7 Hz), 115.7 (d, *J* = 21.4 Hz), 89.8, 87.6 (d, *J* = 6.3 Hz), 59.7, 44.1, 35.9, 26.8, 19.8. ^{19}F NMR (376 MHz, $CDCl_3$) δ -119.0 (ddd, *J* = 10.6, 7.8, 5.3 Hz). IR (thin film, cm^{-1}) 3351, 2926, 1711, 1648, 1523, 1489, 1455, 1357, 1219, 1095, 770, 757, 699, 667. HRMS (ESI) calcd for $C_{22}H_{22}FNaO_3^+$ (M+Na$^+$): 374.1527, found: 374.1531.

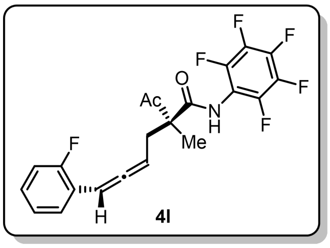

4l

4l. colorless oil, 37% yield, 11:1 *dr*, 95% *ee*. $[\alpha]_D^{20}$ = 51.3 (*c* = 1.7, $CHCl_3$). HPLC analysis: Daicel Chiralpak AD-H*2, 5% iso-propanol/hexane, flow rate = 0.5 mL/min, λ = 254 nm, retention time: 35.9 min (major), 37.4 min (minor). ^{1}H NMR (400 MHz, $CDCl_3$) δ 8.53 (s, 1H), 7.29 (td, *J* = 7.7, 1.8 Hz, 1H), 7.18 (tdd, *J* = 7.4, 5.1, 1.8 Hz, 1H), 7.06 (td, *J* = 7.5, 1.3 Hz, 1H), 7.04 - 6.95 (m, 1H), 6.40 (dt, *J* = 6.6, 2.7 Hz, 1H), 5.51 (q, *J* = 7.1 Hz, 1H), 2.83 (dddd, *J* = 63.2, 14.8, 7.3, 2.7 Hz, 2H), 2.33 (s, 3H), 1.61 (s, 3H). ^{13}C NMR (101 MHz, $CDCl_3$) δ 210.2, 207.3, 170.2, 161.1, 158.7, 128.8 (d, *J* = 8.2), 128.5 (d, *J* = 3.1), 124.3 (d, *J* = 3.5), 121.5 (d, *J* = 12.1), 115.8 (d, *J* = 21.3), 111.9, 89.9 - 88.9 (m), 88.5 (d, *J* = 6.1), 59.3, 36.7, 27.0, 21.5. ^{19}F NMR (376 MHz, $CDCl_3$) δ -118.7 (ddd, *J* = 10.9, 7.7, 5.1 Hz), -144.3- -146.4 (m), -156.4 (t, J = 21.4 Hz), -161.2- -162.6 (m). IR (thin film, cm^{-1}) 3294, 2926, 1696, 1653, 1521, 1490, 1457, 1360, 1220, 1094, 993, 771, 630. HRMS (ESI) calcd for $C_{21}H_{15}F_6NaO_3^+$ (M+Na$^+$): 450.0899, found: 450.0903.

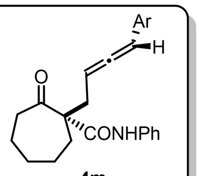

4m

4m. colorless oil, 81% yield, 12:1 *dr*, 87% *ee*. $[\alpha]_D^{20}$ = 13.1 (*c* = 6.3, $CHCl_3$). HPLC analysis: Daicel Chiralpak IA-H*2, 5% iso-propanol/hexane, flow rate = 0.5 mL/min, λ = 254 nm, retention time: 48.0 min (minor), 68.1 min (major). ^{1}H NMR (400 MHz, $CDCl_3$) δ 9.38 (s, 1H), 7.63 - 7.47 (m, 2H), 7.33 (td, *J* = 8.7, 8.1, 2.0 Hz, 2H), 7.28 - 7.22 (m, 1H), 7.16 - 7.06 (m, 2H), 7.01 - 6.82 (m, 2H), 6.33 (ddt, *J* = 6.3, 4.7, 2.4 Hz, 1H), 5.49 - 5.38 (m, 1H), 2.81 (tdd, *J* = 10.7, 6.1, 2.6 Hz, 1H), 2.69 (ddd, *J* = 8.0, 3.9, 2.4 Hz, 2H), 2.62 (ddd, *J* = 11.6, 8.7, 2.3 Hz, 1H), 1.95 - 1.81 (m, 2H), 1.76 (dtd, *J* = 14.8, 9.0, 7.5, 4.7 Hz, 2H), 1.69 - 1.53 (m, 3H), 1.51 - 1.37 (m, 1H). ^{13}C NMR (101 MHz, $CDCl_3$) δ 216.6, 207.5, 168.4, 159.7 (d, *J* = 248.7 Hz), 138.0, 129.1 (d, *J* = 3.4 Hz), 128.4, 128.5 (d, *J* = 3.8 Hz), 124.5 (d, *J* = 2.7 Hz), 124.2 (d, *J* = 2.9 Hz), 121.6 (d, *J* = 12.0 Hz), 120.3 (d, *J* = 5.4 Hz), 115.6 (d, *J* = 21.3 Hz), 89.3 (d, *J* = 8.8 Hz), 87.5 (d, *J* = 6.4 Hz), 63.5, 42.9, 38.7, 32.3, 30.2, 26.5, 25.4. ^{19}F NMR (376 MHz, $CDCl_3$) δ -119.4 (ddd, *J* = 10.6, 7.8, 5.3 Hz). IR (thin film, cm^{-1}) 3323, 2926, 1688, 1597, 1534, 1490, 1442, 1313, 1219, 771, 758, 692. HRMS (ESI) calcd for $C_{24}H_{24}FNaO_2^+$ (M+Na$^+$): 400.1683, found: 400.1684.

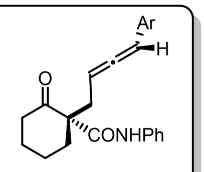

4n

4n. colorless oil, 81% yield, 12:1 *dr*, 99% *ee*. $[\alpha]_D^{20}$ = -13 (*c* = 6.6, $CHCl_3$). HPLC analysis: Daicel Chiralpak IA-H*2, 5% iso-propanol/hexane, flow rate = 1.0 mL/min, λ = 254 nm, retention time: 23.4 min (minor), 30.4 min (major). ^{1}H NMR (400 MHz, $CDCl_3$) δ 8.47 (d, *J* = 10.0 Hz, 1H), 7.57 - 7.46 (m, 2H), 7.36 - 7.20 (m, 3H), 7.16 - 7.07 (m, 2H), 7.01 – 6.85 (m, 2H), 6.33 (ddt, *J* = 13.7, 6.5, 2.5 Hz, 1H), 5.52 - 5.40 (m, 1H), 2.89 - 2.68 (m, 2H), 2.65 - 2.34 (m, 3H), 2.00 - 1.76 (m, 5H). ^{13}C NMR (101 MHz, $CDCl_3$) δ 213.1, 207.3, 168.1, 159.7 (d, *J* = 249.4 Hz), 137.7, 129.1, 128.5 (d, *J* = 2.9 Hz),128.4, 124.7, 124.2 (d, *J* = 3.5 Hz), 121.7 (d, *J* = 12.1 Hz), 120.3, 115.6 (d, *J* = 21.5 Hz), 89.4, 87.5 (d, *J* = 6.7 Hz), 60.7, 41.0, 36.4, 34.5, 26.3, 21.7. ^{19}F NMR (376 MHz, $CDCl_3$) δ -119.4 (ddd, *J* = 10.4, 7.7, 5.2 Hz). IR (thin film, cm^{-1}) 3330, 3019, 2944, 2870, 1684, 1598, 1526, 1489, 1440, 1312, 1216, 775, 746, 691, 667, 506. HRMS (ESI) calcd for $C_{23}H_{22}FNaO_2^+$ (M+Na$^+$): 386.1527, found: 386.1527.

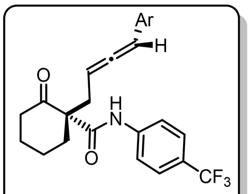

4o

4o. colorless oil, 75% yield, 10:1 *dr*, 96% *ee*. $[\alpha]_D^{20}$ = -1.89 (*c* = 7.3, $CHCl_3$). HPLC analysis: Daicel Chiralpak IA-H*2, 5% iso-propanol/hexane, flow rate = 1.0 mL/min, λ = 254 nm, retention time: 29.4 min (minor), 32.9 min (major). ^{1}H NMR (400 MHz, $CDCl_3$) δ 9.09 (s, 1H), 7.66 (d, *J* = 8.5 Hz, 2H), 7.57 (d, *J* = 8.5 Hz, 2H), 7.22 (td, *J* = 7.7, 1.8 Hz, 1H), 7.18 - 7.03 (m, 1H), 7.00 - 6.78 (m, 2H), 6.31 (dt, *J* = 6.5, 2.4 Hz, 1H), 5.43 (q, *J* = 7.6 Hz, 1H), 2.91 - 2.71 (m, 2H), 2.64 (ddd, *J* = 15.7, 7.6, 4.7 Hz, 1H), 2.55 - 2.32 (m, 2H), 2.07 (dt, *J* = 14.4, 5.2 Hz, 1H), 1.88 (dtt, *J* = 11.0, 7.5, 4.0 Hz, 4H). ^{13}C NMR (101 MHz, $CDCl_3$) δ 213.6, 207.3, 169.0, 159.7 (d, *J* = 249.5 Hz), 140.8, 128.6 (d, *J* = 8.1 Hz), 128.4 (d, *J* = 3.5 Hz), 126.3 (q, *J* = 3.5 Hz), 125.6, 124.2 (d, *J* = 3.6 Hz), 122.9, 121.5 (d, *J* = 12.1 Hz), 119.9, 115.7 (d, *J* = 21.4 Hz), 89.0, 87.8 (d, *J* = 6.4 Hz), 60.4, 40.8, 36.4, 34.8, 26.3, 21.4. ^{19}F NMR (376 MHz, $CDCl_3$) δ -62.1, -119.1 (ddd, *J* = 10.5, 7.7, 5.3 Hz). IR (thin film, cm^{-1}) 3330, 3023, 2928, 2870, 1685, 1603, 1525, 1490, 1322, 1217, 1165, 1114, 1067, 1017, 842, 753, 668, 594. HRMS (ESI) calcd for $C_{24}H_{21}F_4NaO_3^+$ (M+Na$^+$): 454.1401, found: 454.1402.

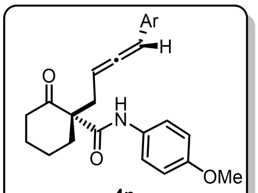

4p

4p. colorless oil, 83% yield, 13:1 *dr*, >99% *ee*. $[\alpha]_D^{20}$ = -14.38 (*c* = 5, $CHCl_3$). HPLC analysis: Daicel Chiralpak OD-H, 5% iso-propanol/hexane, flow rate =1.0 mL/min, λ = 254 nm, retention time: 23.8 min (major), 43.2 min (minor). ^{1}H NMR (400 MHz, $CDCl_3$) δ 8.30 (s, 1H), 7.45 - 7.36 (m, 2H), 7.31 - 7.23 (m, 1H), 7.12 (tdd, *J* = 7.2, 6.2, 1.8 Hz, 1H), 7.01 - 6.92 (m, 2H), 6.88 - 6.82 (m, 2H), 6.32 (dt, *J* = 6.5, 2.3 Hz, 1H), 5.46 (q, *J* = 7.7 Hz, 1H), 3.79 (s, 3H), 2.76 (dddd, *J* = 47.6, 14.3, 7.8, 2.4 Hz, 2H), 2.65 - 2.54 (m, 1H), 2.54 - 2.37 (m, 2H), 1.99 - 1.74 (m, 5H). ^{13}C NMR (101 MHz, $CDCl_3$) δ 213.1, 207.3, 167.9, 159.7 (d, *J* = 249.3 Hz), 156.7, 130.8, 128.5, 128.4 (d, *J* = 4.3 Hz), 124.2 (d, *J* = 3.5 Hz), 122.0, 121.7 (d, *J* = 12.0 Hz), 115.6 (d, *J* = 21.5 Hz), 114.3, 89.5, 87.4 (d, *J* = 6.6 Hz), 60.6, 55.6, 41.0, 36.4, 34.5, 26.3, 21.7. ^{19}F NMR (376 MHz, $CDCl_3$) δ -119.4 (ddd, *J* = 10.4, 7.8, 5.3 Hz). IR (thin film, cm^{-1}) 3338, 2928, 1671, 1599, 1510, 1489, 1455, 1411, 1299, 1220, 1178, 1033, 826, 749, 667, 521. HRMS (ESI) calcd for $C_{24}H_{24}FNaO_3^+$ (M+Na$^+$): 416.1632, found: 416.1633.

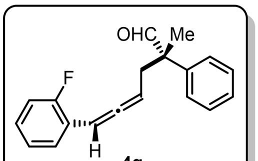

4q

4q. colorless oil, 61% yield, 9:1 *dr*, 97% *ee*. $[\alpha]_D^{20}$ = -12.5 (*c* = 3.2, $CHCl_3$). HPLC analysis: Daicel Chiralpak OD-H*2, 5% iso-propanol/hexane, flow rate =1.0 mL/min, λ = 254 nm, retention time: 48.8 min (minor), 61.3 min (major). ^{1}H NMR (400 MHz, $CDCl_3$) δ 9.56 (d, *J* = 3.6 Hz, 1H), 7.39 (ddd, *J* = 7.8, 6.7, 1.9 Hz, 2H), 7.34 - 7.26 (m, 3H), 7.12 (dddd, *J* = 10.8, 9.2, 5.9, 2.1 Hz, 1H), 7.05 - 6.89 (m, 3H), 6.31 - 6.23 (m, 1H), 5.34 (q, *J* = 7.5 Hz, 1H), 2.84 - 2.65 (m, 2H), 1.57 (s, 3H). ^{13}C NMR (101 MHz, $CDCl_3$) δ 201.7,

158.4, 139.0, 138.4, 129.2, 128.3, 128.3, 128.2, 127.6, 127.4, 124.2 (d, *J* = 3.2 Hz), 115.6 (d, *J* = 21.2 Hz), 89.9, 86.9 (d, *J* = 6.9 Hz), 54.2, 36.4, 18.8. ^{19}F NMR (376 MHz, $CDCl_3$) δ -119.8 (dt, *J* = 11.9, 6.0 Hz). IR (thin film, cm^{-1}) 2926, 2874, 2853, 1718, 1491, 1457, 1219, 1076, 1026, 771, 700, 669. HRMS (ESI) calcd for $C_{19}H_{17}FNaO^+$ (M+Na^+): 303.1156, found: 303.1160.

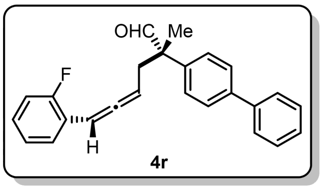

4r

4r. colorless oil, 71% yield, 6:1 *dr*, 98% *ee*. $[\alpha]_D^{20}$ = -2.9 (*c* = 3.5, $CHCl_3$). HPLC analysis: Daicel Chiralpak OD-H*2, 5% iso-propanol/hexane, flow rate =1.0 mL/min, λ = 254 nm, retention time: 11.7 min (minor), 13.7 min (major). ^{1}H NMR (400 MHz, $CDCl_3$) δ 9.59 (s, 1H), 7.64 - 7.53 (m, 4H), 7.48 - 7.41 (m, 2H), 7.39 - 7.32 (m, 3H), 7.15 - 7.02 (m, 1H), 7.01 - 6.92 (m, 2H), 6.88 (td, *J* = 7.5, 1.2 Hz, 1H), 6.30 (dt, *J* = 6.4, 2.4 Hz, 1H), 5.38 (q, *J* = 7.4 Hz, 1H), 2.79 (dddd, *J* = 44.9, 14.3, 7.8, 2.4 Hz, 2H), 1.61 (s, 3H). ^{13}C NMR (101 MHz, $CDCl_3$) δ 207.3, 201.4, 159.5 (d, *J* = 249.1 Hz), 140.4 (d, *J* = 8.9 Hz), 137.9 (d, *J* = 17.9 Hz), 128.9, 128.8, 128.2 (d, *J* = 7.5 Hz), 128.1 (d, *J* = 2.6 Hz), 127.8, 127.7, 127.7, 127.6, 127.5, 127.1, 124.0 (d, *J* = 3.7 Hz), 121.7 (d, *J* = 12.1 Hz), 115.4 (d, *J* = 21.4 Hz), 89.7, 86.8 (d, *J* = 6.8 Hz), 53.9, 36.2, 18.7. ^{19}F NMR (376 MHz, $CDCl_3$) δ -119.8 (ddd, *J* = 10.5, 7.5, 5.3 Hz). IR (thin film, cm^{-1}) 2959, 2925, 2870, 2853, 2807, 1720, 1682, 1604, 1487, 1404, 1268, 1220, 1076, 1007, 841, 770, 698. HRMS (ESI) calcd for $C_{25}H_{21}FNaO^+$ (M+Na^+): 379.1469, found: 379.1474.

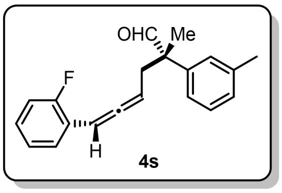

4s

4s. colorless oil, 55 yield, 4:1 *dr*, 96% *ee*. $[\alpha]_D^{20}$ = 23.3 (*c* = 1.5 $CHCl_3$). HPLC analysis: Daicel Chiralpak OD-H*2, 2% iso-propanol/hexane, flow rate =0.3 mL/min, λ = 254 nm, retention time: 50.7 min (minor), 64.9 min (major). ^{1}H NMR (400 MHz, $CDCl_3$) δ 9.56 (s, 1H), 7.32 (d, *J* = 7.6 Hz, 1H), 7.18 - 7.11 (m, 3H), 7.10 (s, 1H), 7.06 - 6.89 (m, 3H), 6.30 (ddt, *J* = 10.7, 6.4, 2.6 Hz, 1H), 5.37 (q, *J* = 7.3 Hz, 1H), 2.91 - 2.66 (m, 2H), 2.36 (s, 3H), 1.58 (s, 3H).^{13}C NMR (101 MHz,$CDCl_3$) δ 207.4, 201.7, 138.9, 138.7 (d, *J* = 4.7 Hz), 128.9 (d, *J* = 4.0 Hz), 128.3 (d, *J* = 3.1 Hz), 128.1 (d, *J* = 5.4 Hz), 128.0 (d, *J* = 3.6 Hz), 125.4, 124.3 (d, *J* = 6.8 Hz), 124.0 (d, *J* = 3.4 Hz), 121.9, 115.6 (d, *J* = 8.8 Hz), 115.4 (d, *J* = 8.8 Hz), 90.0, 86.8, 54.1, 36.3, 21.7, 18.8. ^{19}F NMR (376 MHz, $CDCl_3$) δ -119.9 (ddd, J = 10.5, 7.3, 5.4 Hz). IR (thin film, cm^{-1}) 2980, 2929, 2854, 2707, 1715, 1607, 1504, 1453, 1217, 1157, 855, 763, 663. HRMS (ESI) calcd for $C_{20}H_{19}FNaO^+$ (M+Na^+): 317.1312, found: 317.1324.

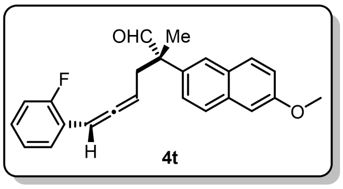

4t

4t. colorless oil, 64% yield, 4:1 *dr*, 97% *ee*. $[\alpha]_D^{20}$ = 14.1 (*c* = 4.2, $CHCl_3$). HPLC analysis: Daicel Chiralpak AS-H, 5% iso-propanol/hexane, flow rate = 1.0 mL/min, λ = 254 nm, retention time: 7.8 min (minor), 8.0 min (major). ^{1}H NMR (400 MHz, $CDCl_3$) δ 9.59 (s, 1H), 7.75 - 7.66 (m, 3H), 7.33 (ddd, *J* = 8.6, 6.2, 2.0 Hz, 1H), 7.17 (dt, *J* = 9.9, 2.5 Hz, 1H), 7.12 (t, *J* = 2.6 Hz, 1H), 7.08 - 6.98 (m, 1H), 6.97 - 6.84 (m, 1H), 6.74 (td, *J* = 7.8, 1.8 Hz, 1H), 6.63 (td, *J* = 7.5, 1.2 Hz, 1H), 6.26 (ddt, *J* = 9.9, 6.5, 2.5 Hz, 1H), 5.36 (q, *J* = 7.4 Hz, 1H), 3.93 (s, 3H), 2.95 - 2.69 (m, 2H), 1.66 (s, 3H). ^{13}C NMR (101 MHz, $CDCl_3$) δ 207.4, 201.7, 158.2, 133.7 (d, *J* = 3.8 Hz), 129.6, 129.0, 128.1 (d, *J* = 3.1 Hz), 128.0, 127.7, 126.3, 125.5, 124.0 (d, *J* = 3.5 Hz), 123.8 (d, *J* = 3.8 Hz), 121.7 (d, *J* = 11.8 Hz), 119.3, 115.3 (d, *J* = 21.3 Hz), 105.6, 105.5, 89.8, 86.7 (d, *J* = 6.9 Hz), 55.5, 54.2, 36.2, 18.8. ^{19}F NMR (376 MHz, $CDCl_3$) δ -120.0 (ddd, *J* = 10.5, 7.7, 5.3 Hz). IR (thin film, cm^{-1}) 2976, 2927, 2854, 1707, 1449, 1368, 1217, 1148, 844, 667, 617. HRMS (ESI) calcd for $C_{24}H_{21}FNaO2S^+$ (M+Na^+): 383.1418, found: 383.1421.

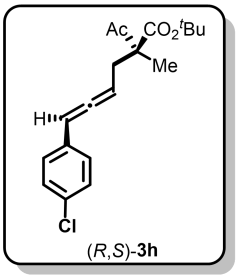

(*R*,*S*)-**3h**

(***R,S***)**-3h**. colorless oil, 57% yield, 3:1 *dr*, 86% *ee*. $[\alpha]_D^{20}$ = 23.1 (*c* = 2.2, $CHCl_3$). HPLC analysis: Daicel Chiralpak AD-H*2, 5% iso-propanol/hexane, flow rate = 1.0 mL/min, λ = 254 nm, retention time: 10.6 min (minor) 11.6 min (major). ^{1}H NMR (400 MHz, $CDCl_3$) δ 7.30 - 7.26 (m, 2H), 7.23 - 7.16 (m, 2H), 6.10 (dt, *J* = 6.4, 2.4 Hz, 1H), 5.44 (td, *J* = 8.0, 6.4 Hz, 1H), 2.65 (qdd, *J* = 14.2, 7.9, 2.4 Hz, 2H), 2.17 (s, 3H), 1.49 (s, 9H), 1.40 (s, 3H).

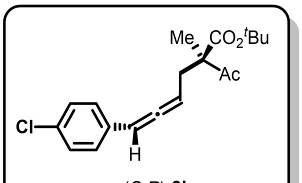

(*S*,*R*)-**3h**

(***S,R***)**-3h**. colorless oil, 64% yield, 4:1 *dr*, 92% *ee*. $[\alpha]_D^{20}$ = -17.1 (*c* = 1.4, $CHCl_3$). HPLC analysis: Daicel Chiralpak AD-H*2, 5% iso-propanol/hexane, flow rate = 1.0 mL/min, λ = 254 nm, retention time: 10.6 min (major) 11.6 min (minor). ^{1}H NMR (400 MHz, $CDCl_3$) δ 7.30 – 7.26 (m, 2H), 7.23 – 7.16 (m, 2H), 6.10 (dt, *J* = 6.4, 2.4 Hz, 1H), 5.44 (td, *J* = 8.0, 6.4 Hz, 1H), 2.65 (qdd, *J* = 14.2, 7.9, 2.4 Hz, 2H), 2.17 (s, 3H), 1.49 (s, 9H), 1.40 (s, 3H).

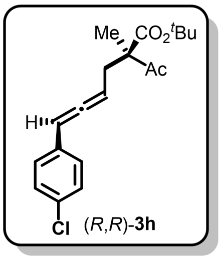

(*R*,*R*)-**3h**

(***R,R***)**-3h**. colorless oil, 67% yield, 10:1 *dr*, 97% *ee*. $[\alpha]_D^{20}$ = -29.3 (*c* = 0.9, $CHCl_3$). HPLC analysis: Daicel Chiralpak AD-H*2, 5% iso-propanol/hexane, flow rate = 1.0 mL/min, λ = 254 nm, retention time: 9.9 min (minor) 12.5 min (major). ^{1}H NMR (400 MHz, $CDCl_3$) δ 7.30 – 7.26 (m, 2H), 7.23 – 7.16 (m, 2H), 6.10 (dt, *J* = 6.4, 2.4 Hz, 1H), 5.44 (td, *J* = 8.0, 6.4 Hz, 1H), 2.65 (qdd, *J* = 14.2, 7.9, 2.4 Hz, 2H), 2.17 (s, 3H), 1.49 (s, 9H), 1.40 (s, 3H).

4. Supplementary Figures

4.1 NMR spectra

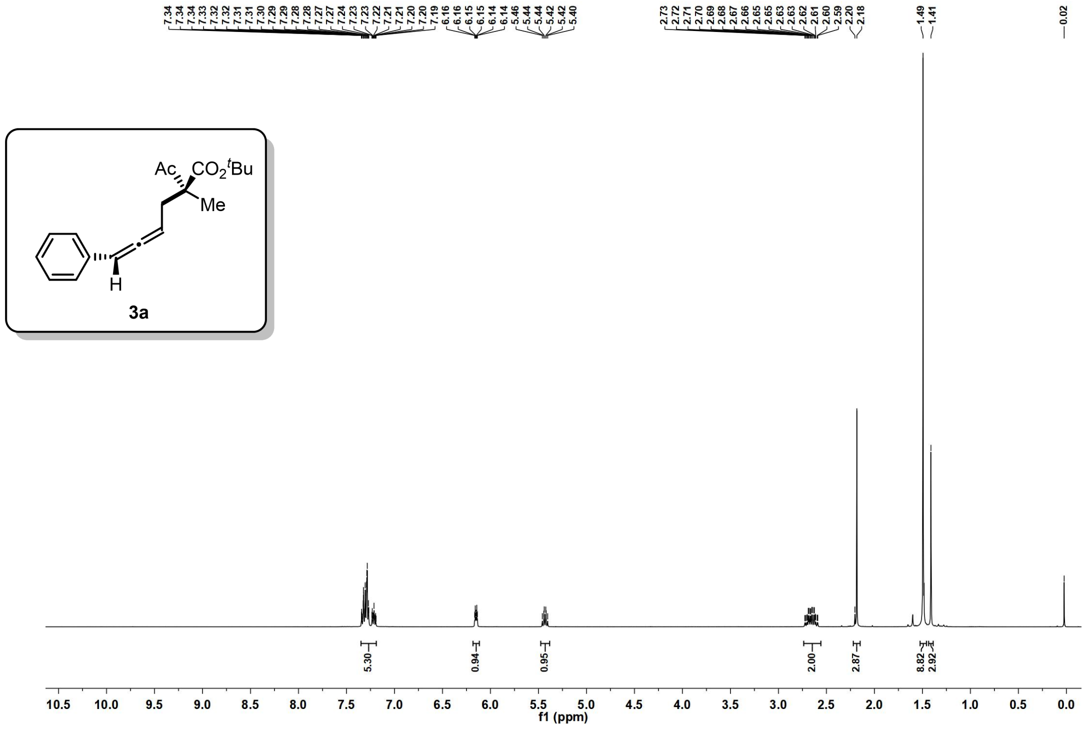

Supplementary Fig. 1. ^{1}H NMR spectra of compound **3a**. ^{1}H NMR (400 MHz, 298K) in $CDCl_3$

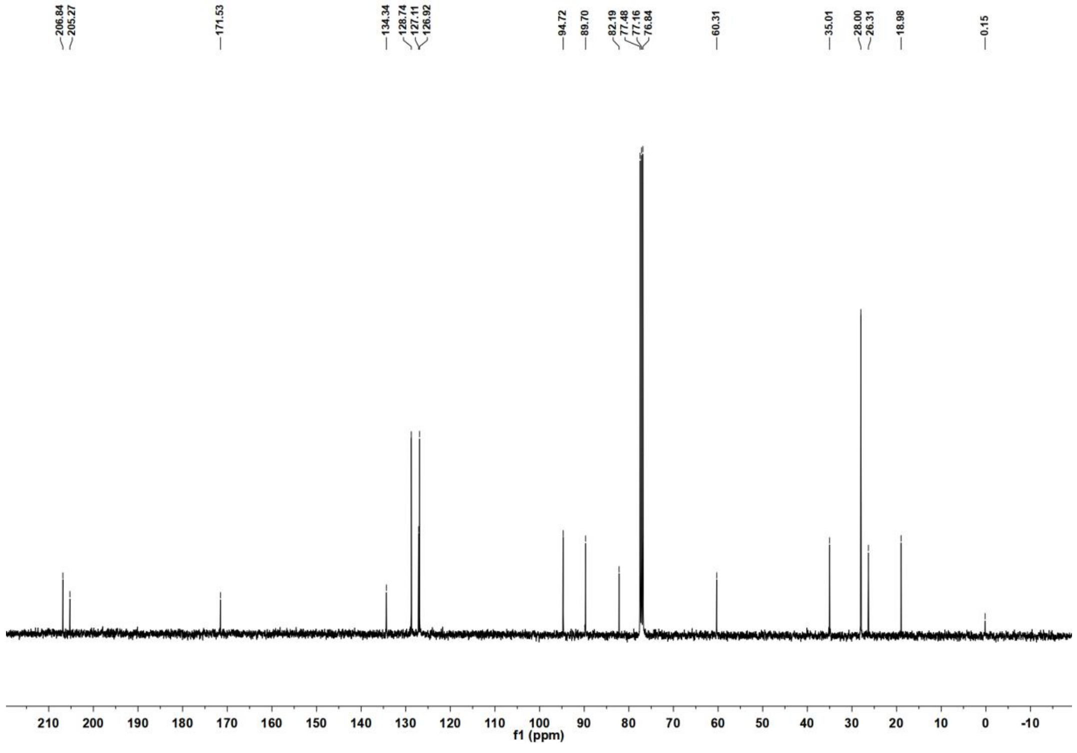

Supplementary Fig. 2. ^{13}C NMR spectra of compound **3a**. ^{13}C NMR (101 MHz, 298K) in $CDCl_3$

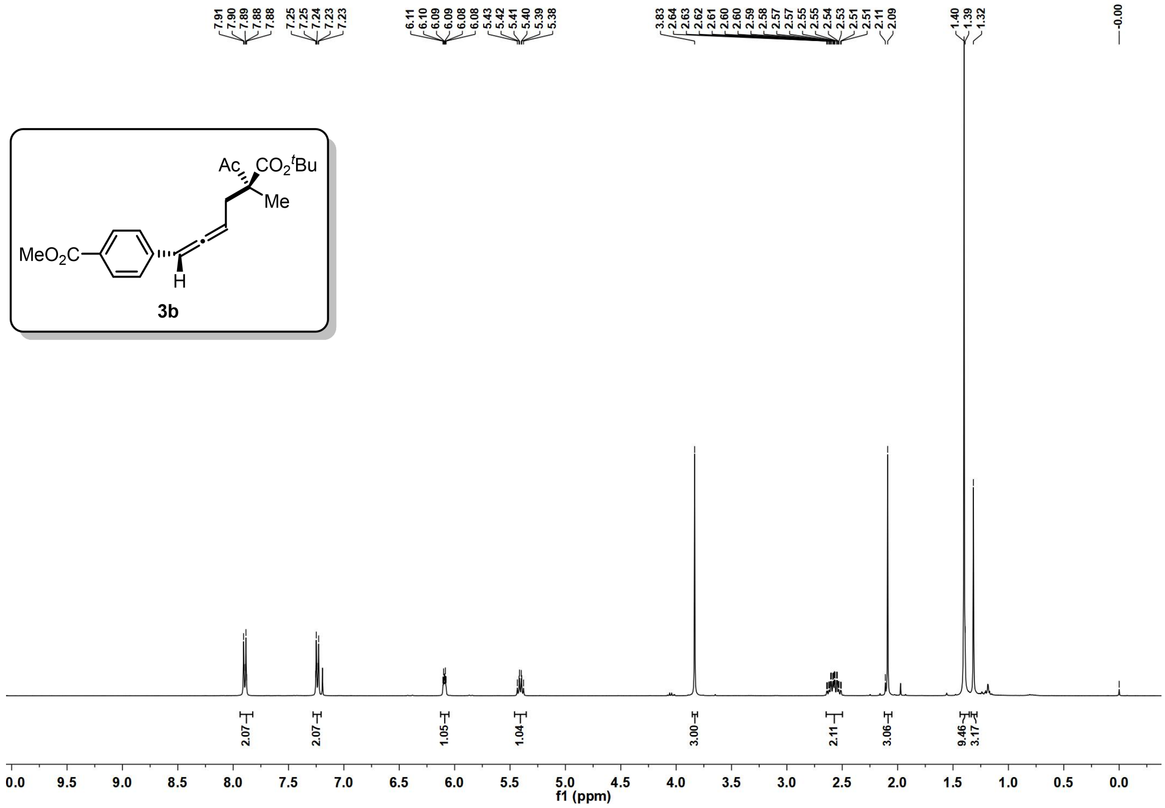

Supplementary Fig. 3. ^{1}H NMR spectra of compound **3b**. ^{1}H NMR (400 MHz, 298K) in $CDCl_3$

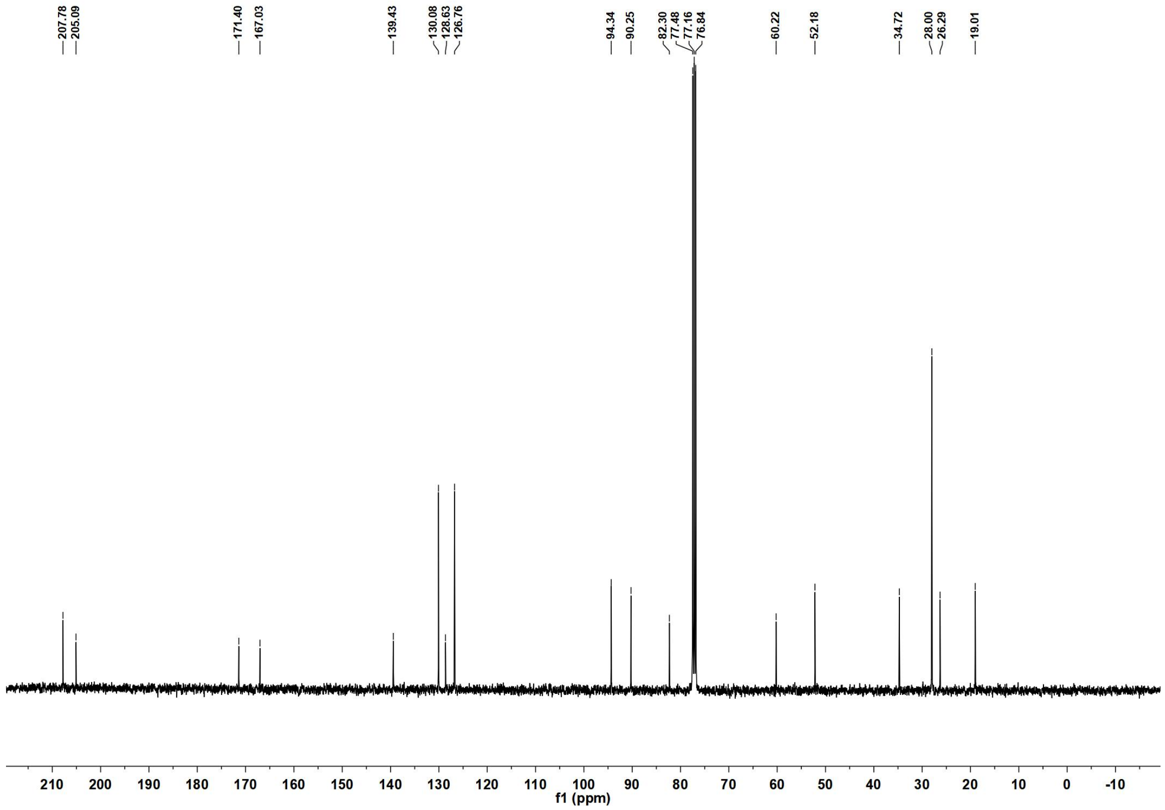

Supplementary Fig. 4. ^{13}C NMR spectra of compound **3b**. ^{13}C NMR (101 MHz, 298K) in $CDCl_3$

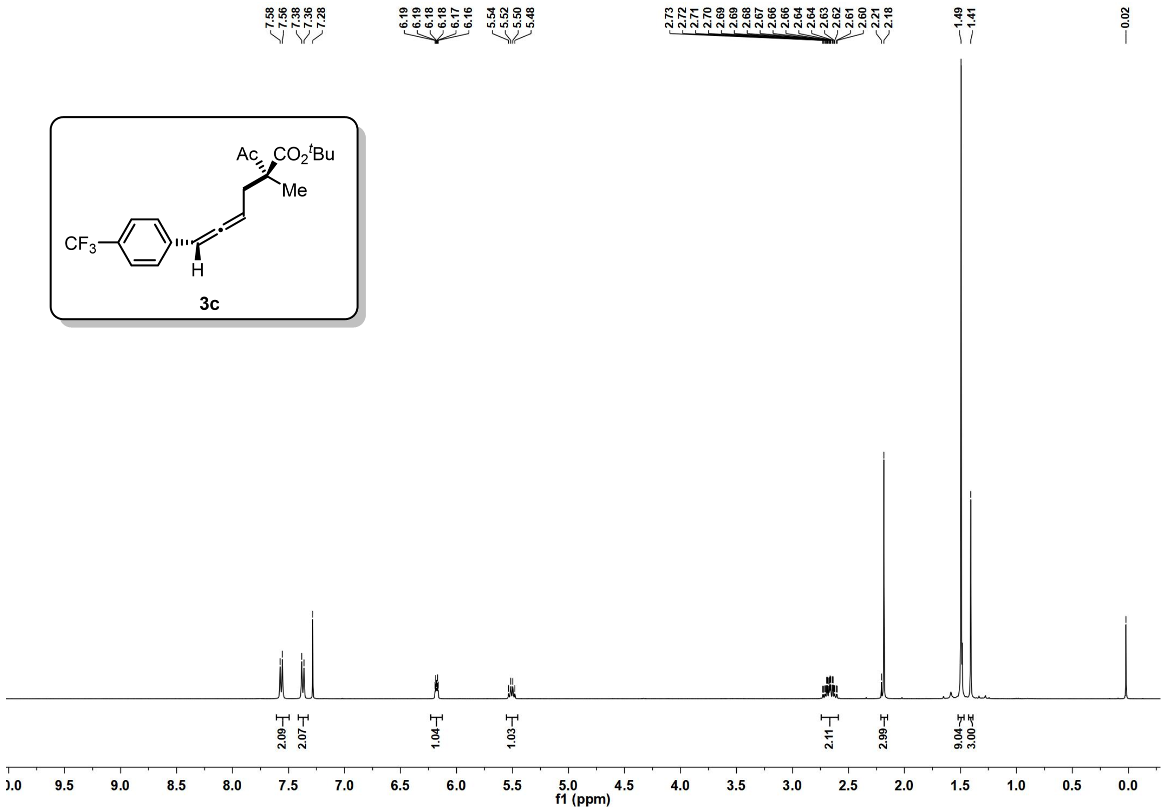

Supplementary Fig. 5. ^{1}H NMR spectra of compound **3c**. ^{1}H NMR (400 MHz, 298K) in $CDCl_3$

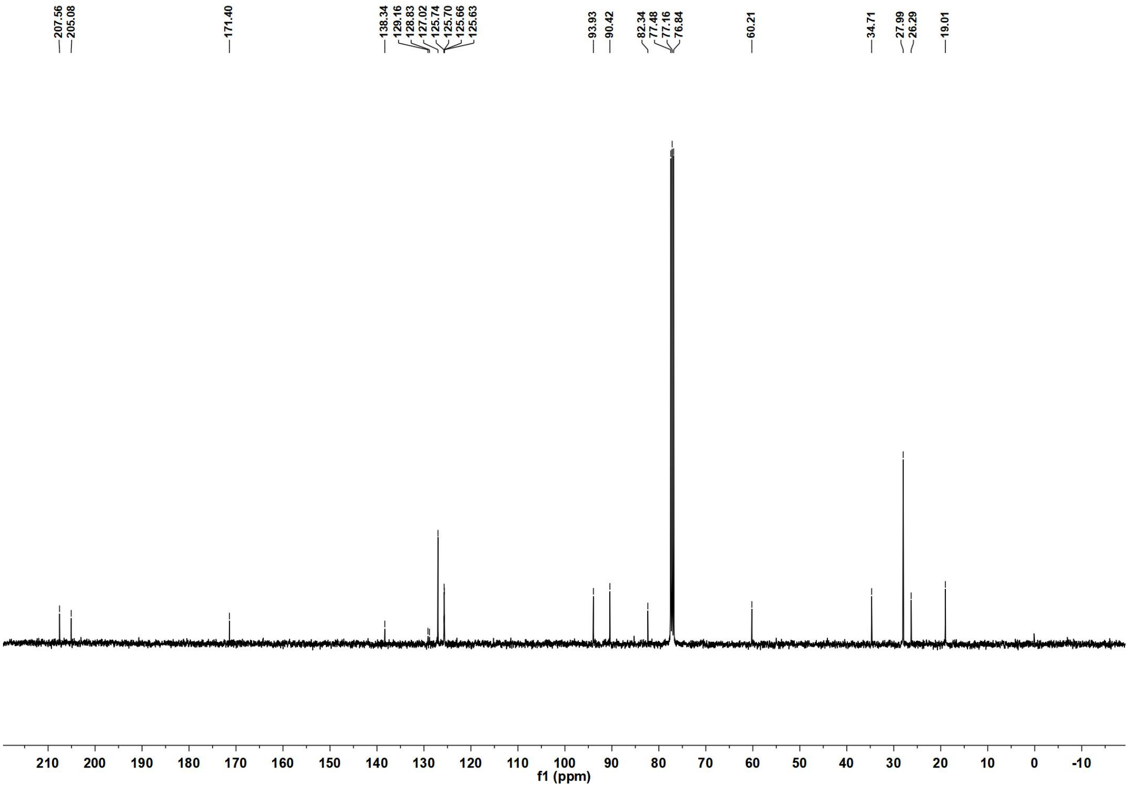

Supplementary Fig. 6. ^{13}C NMR spectra of compound **3c**. ^{13}C NMR (101 MHz, 298K) in $CDCl_3$

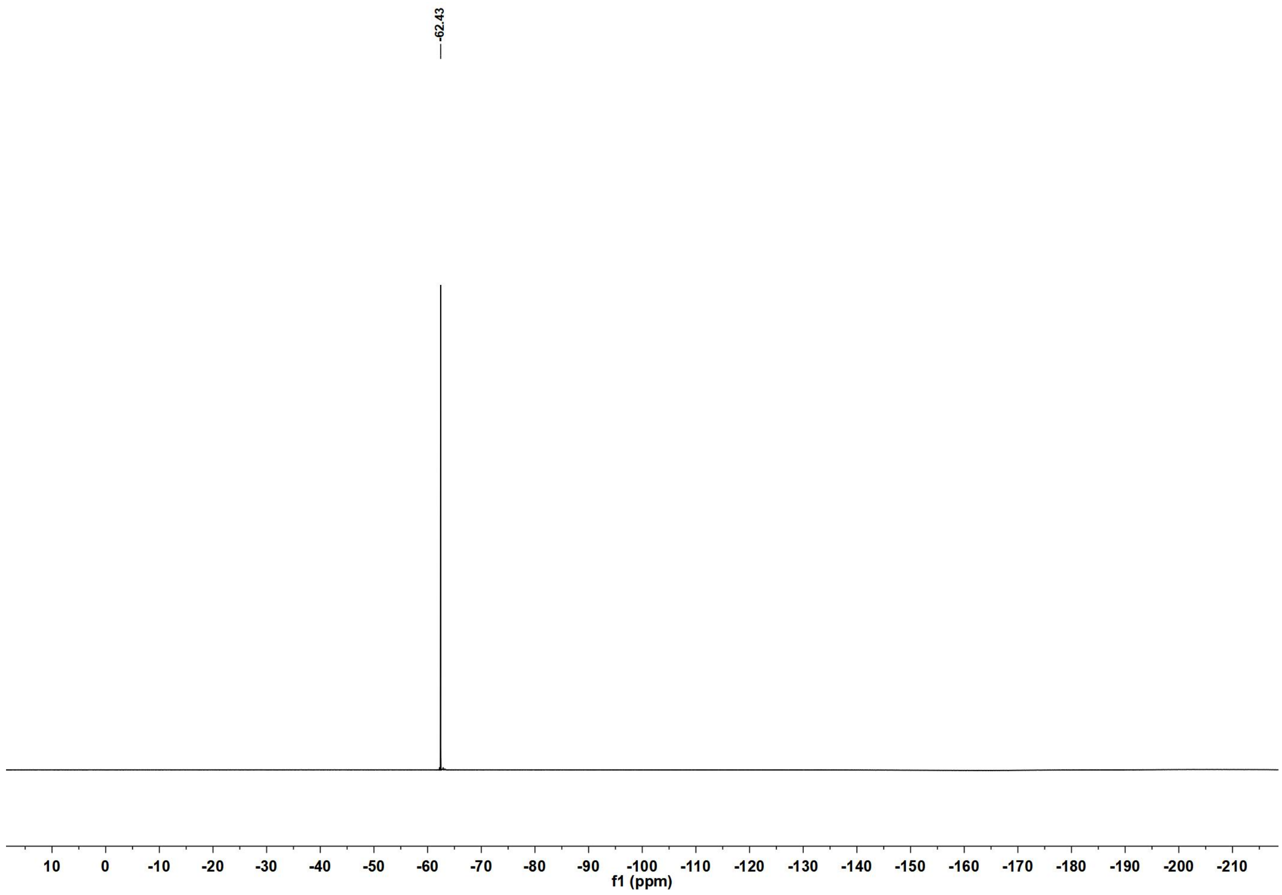

Supplementary Fig. 7. ^{19}F NMR spectra of compound **3c**. ^{19}F NMR (376 MHz, 298K) in $CDCl_3$

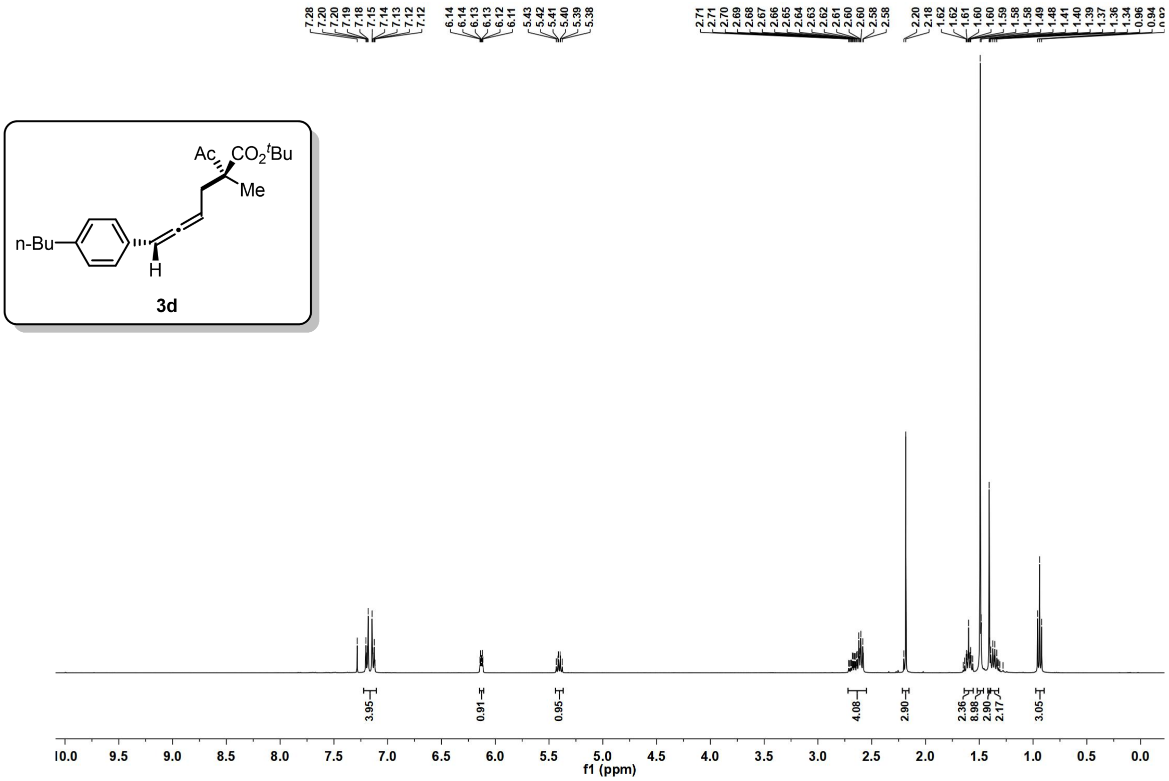

Supplementary Fig. 8. ^{1}H NMR spectra of compound **3d**. ^{1}H NMR (400 MHz, 298K) in $CDCl_3$

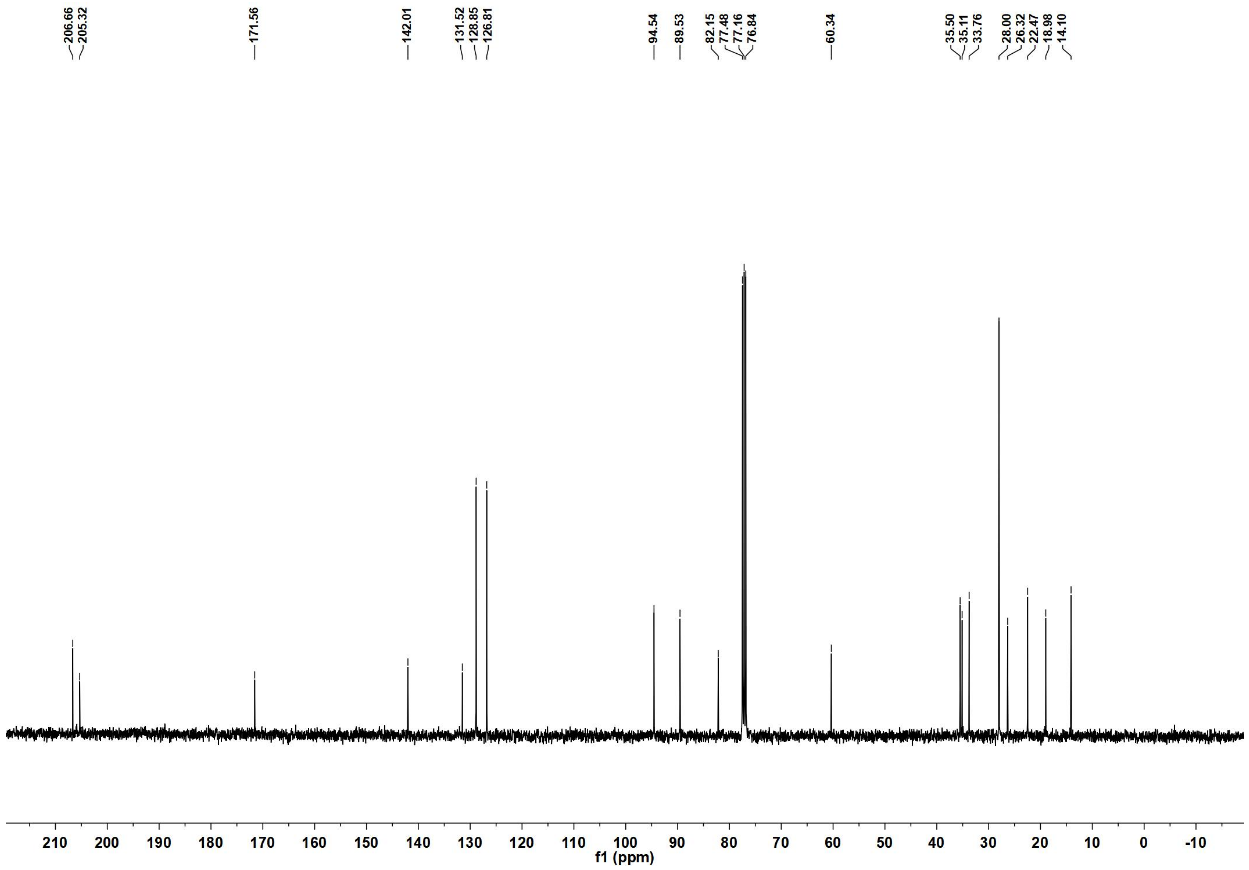

Supplementary Fig. 9. ^{13}C NMR spectra of compound **3d**. ^{13}C NMR (101 MHz, 298K) in $CDCl_3$

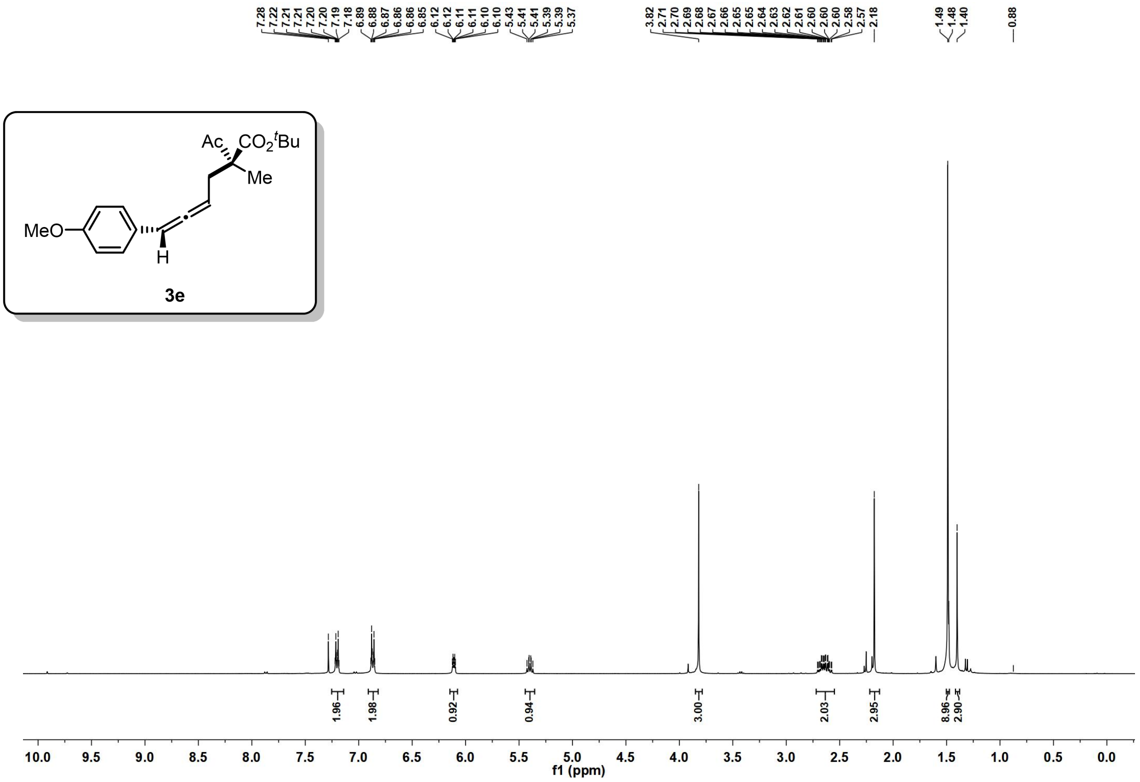

Supplementary Fig. 10. ^{1}H NMR spectra of compound **3e**. ^{1}H NMR (400 MHz, 298K) in $CDCl_3$

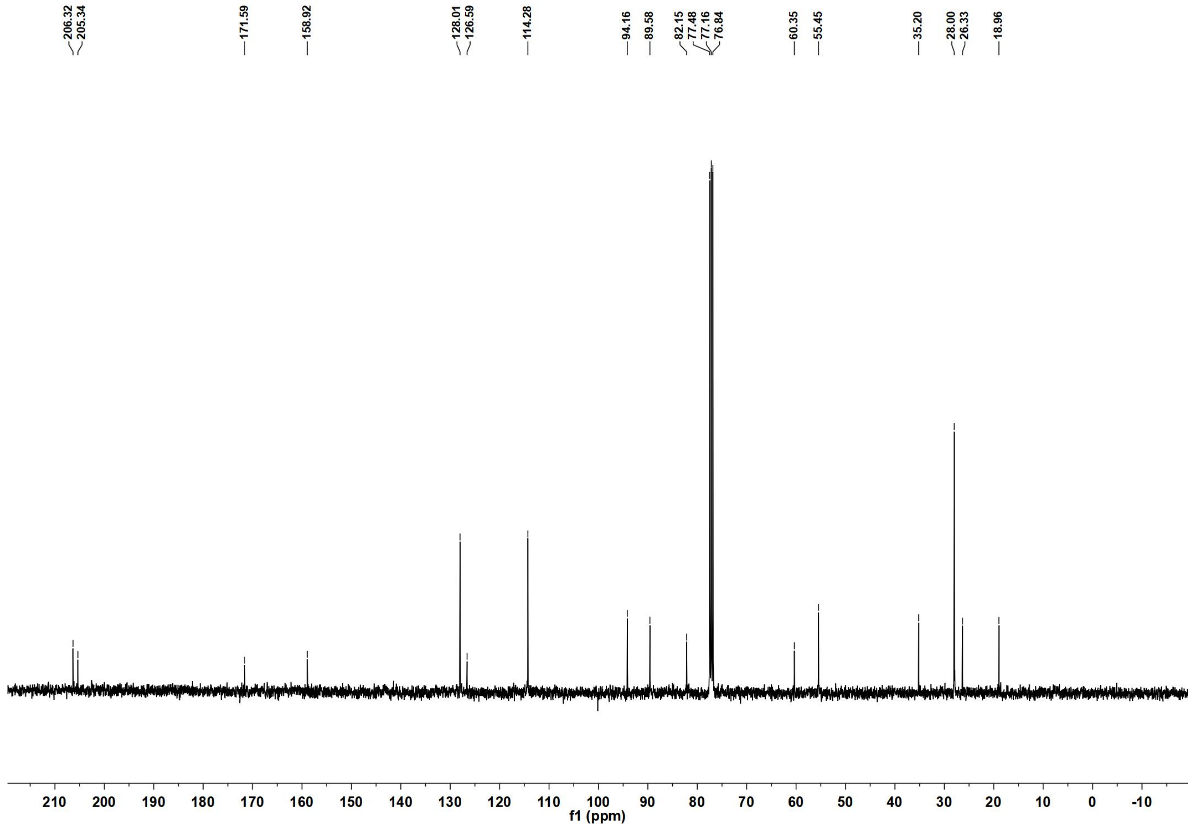

Supplementary Fig. 11. ^{13}C NMR spectra of compound **3e**. ^{13}C NMR (101 MHz, 298K) in $CDCl_3$

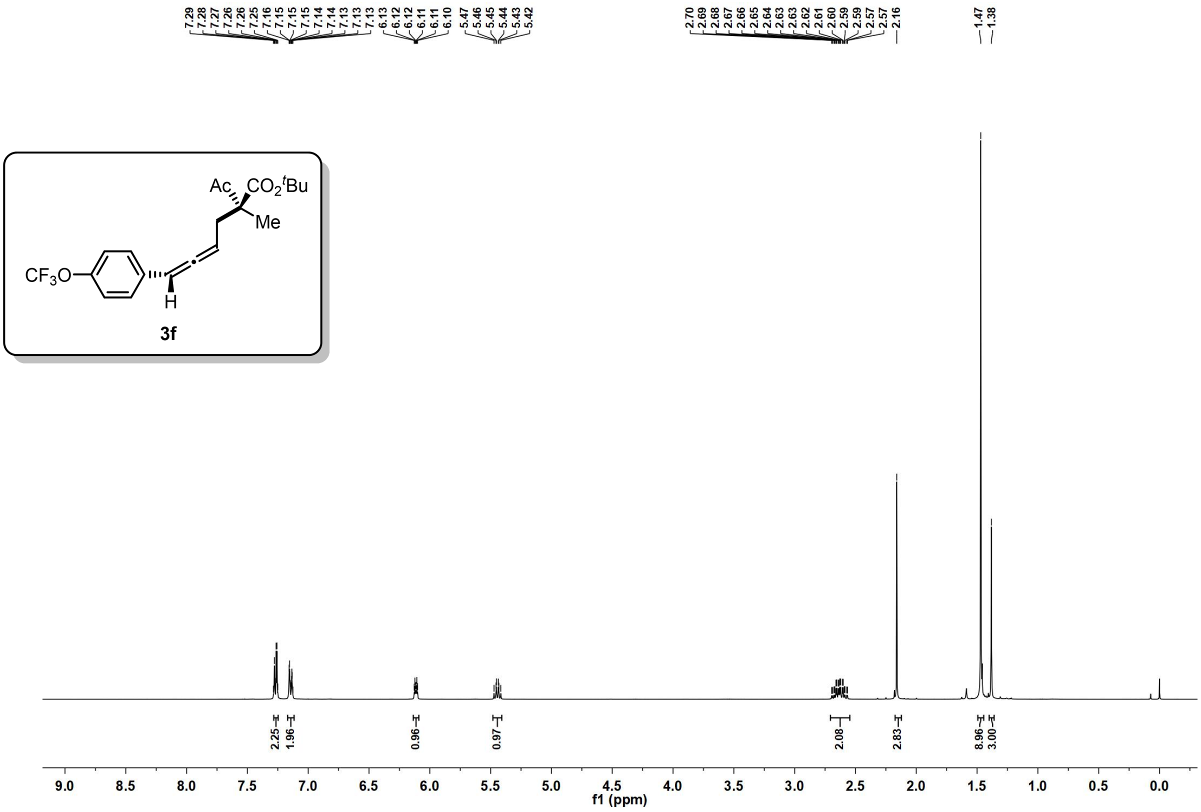

Supplementary Fig. 12. ^{1}H NMR spectra of compound **3f**. ^{1}H NMR (400 MHz, 298K) in $CDCl_3$

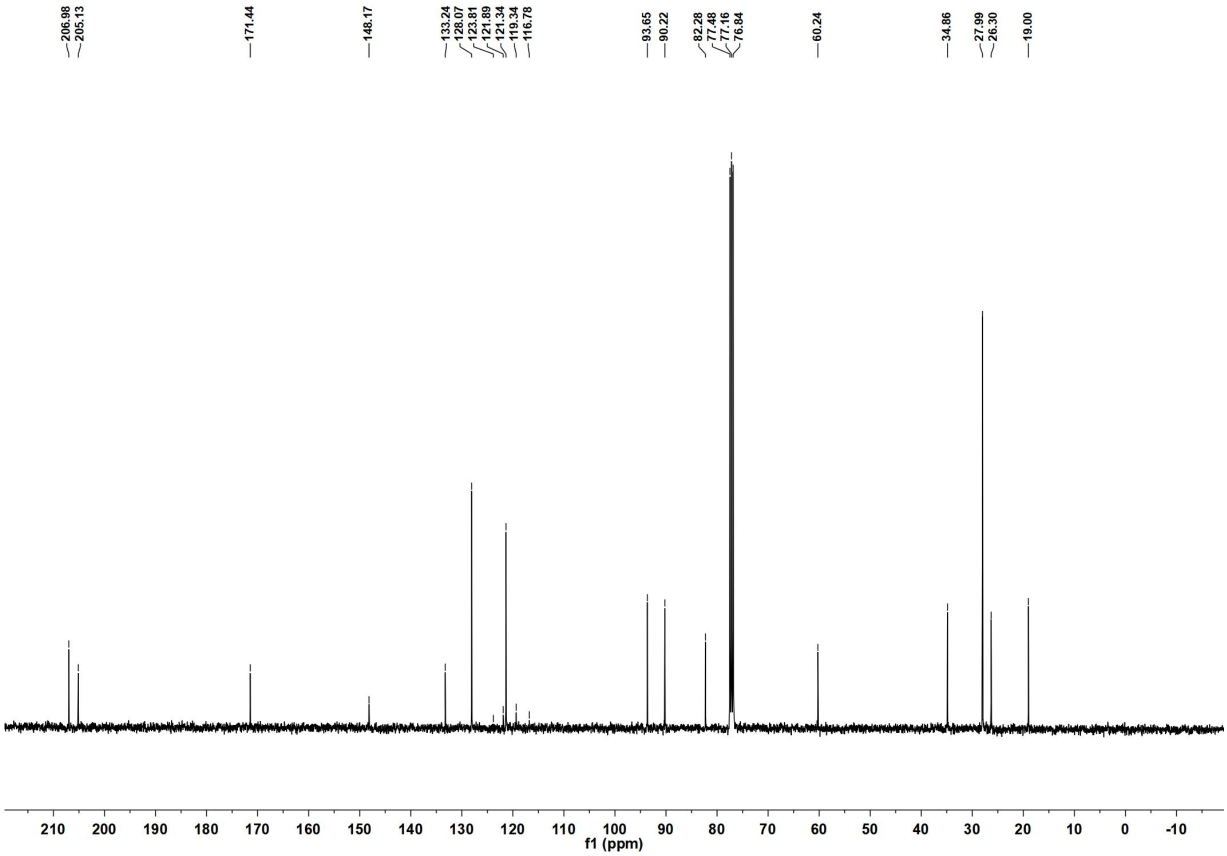

Supplementary Fig. 13. ^{13}C NMR spectra of compound **3f**. ^{13}C NMR (101 MHz, 298K) in $CDCl_3$

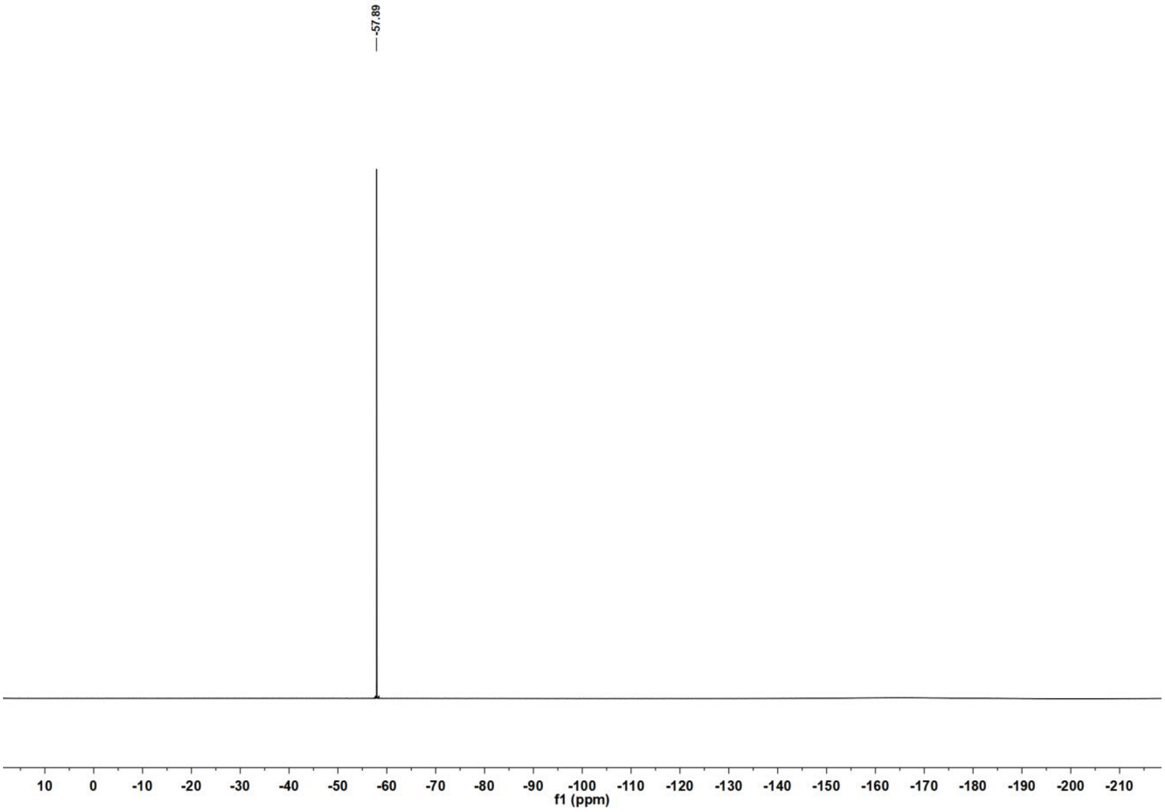

Supplementary Fig. 14. ^{19}F NMR spectra of compound **3f**. ^{19}F NMR (376 MHz, 298K) in $CDCl_3$

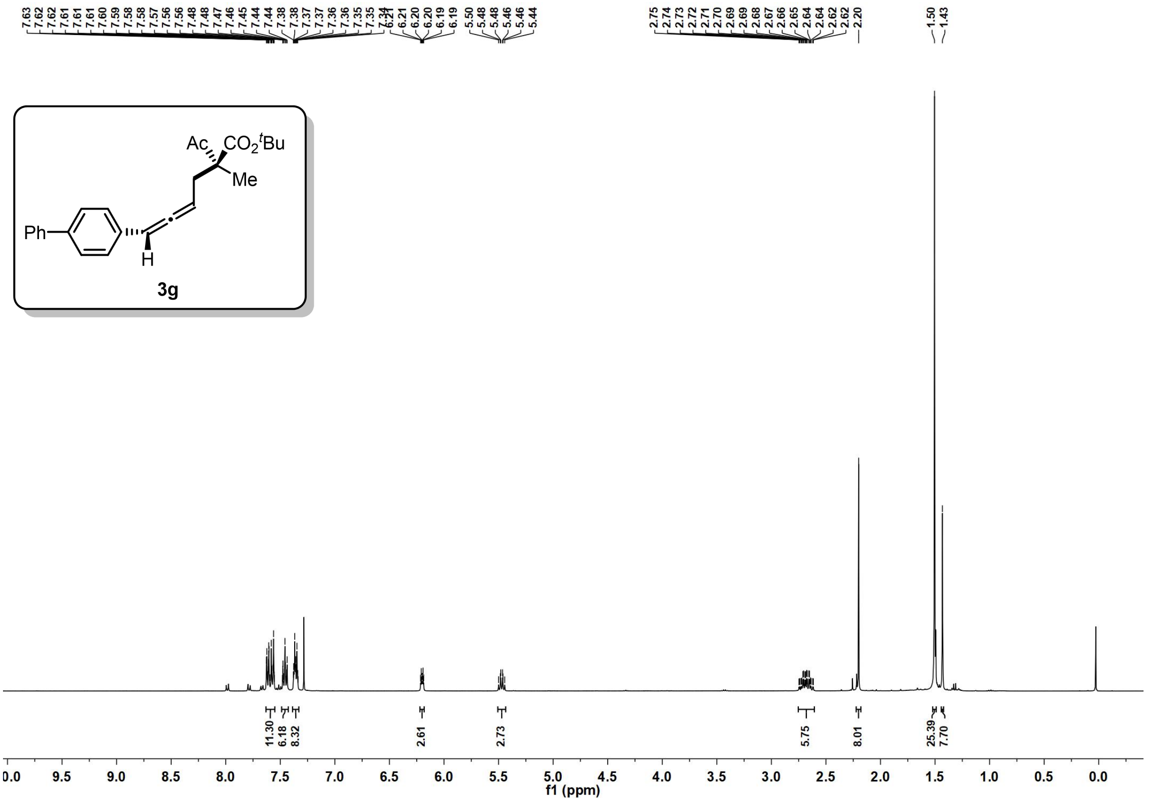

Supplementary Fig. 15. ^{1}H NMR spectra of compound **3g**. ^{1}H NMR (400 MHz, 298K) in $CDCl_3$

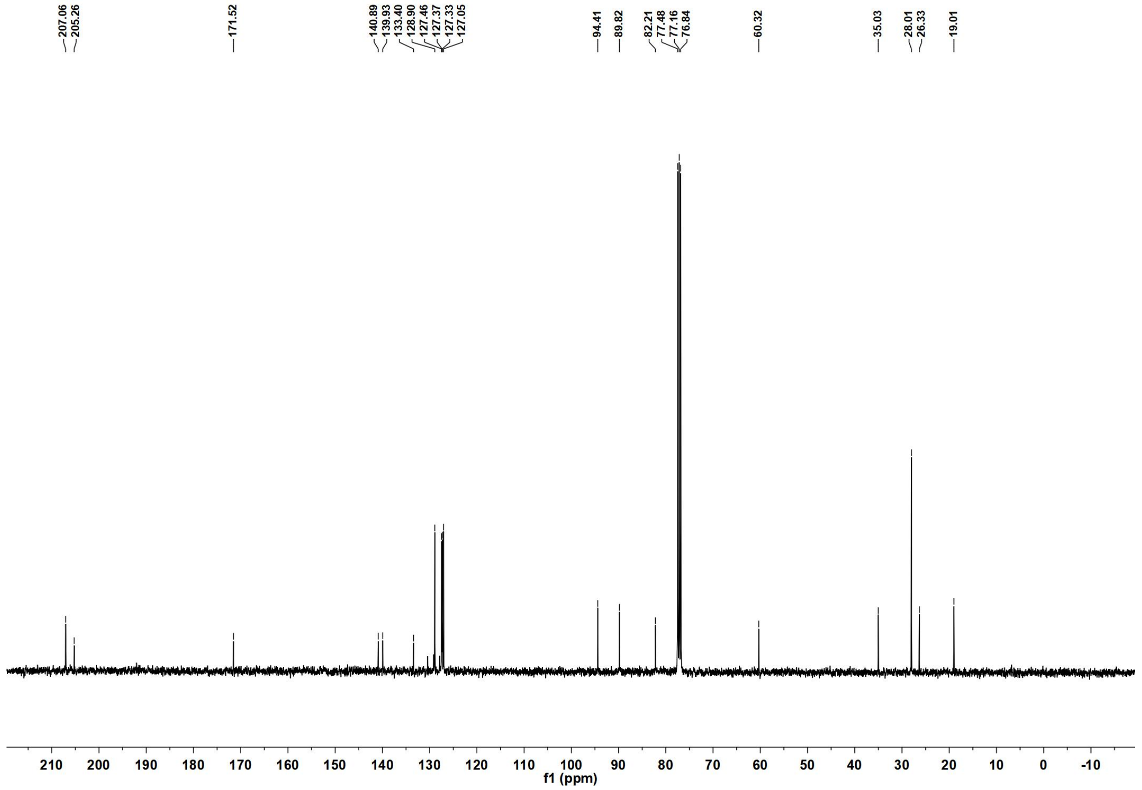

Supplementary Fig. 16. ^{13}C NMR spectra of compound **3g**. ^{13}C NMR (101 MHz, 298K) in $CDCl_3$

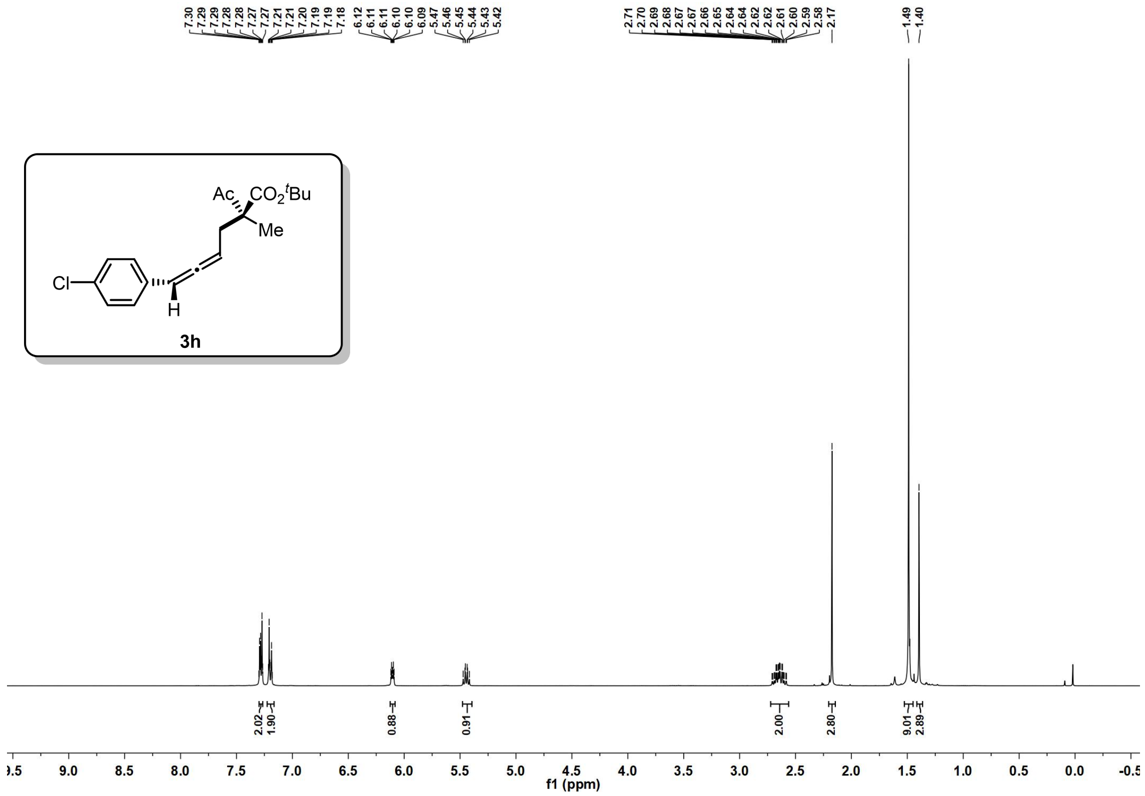

Supplementary Fig. 17. ^{1}H NMR spectra of compound **3h**. ^{1}H NMR (400 MHz, 298K) in $CDCl_3$

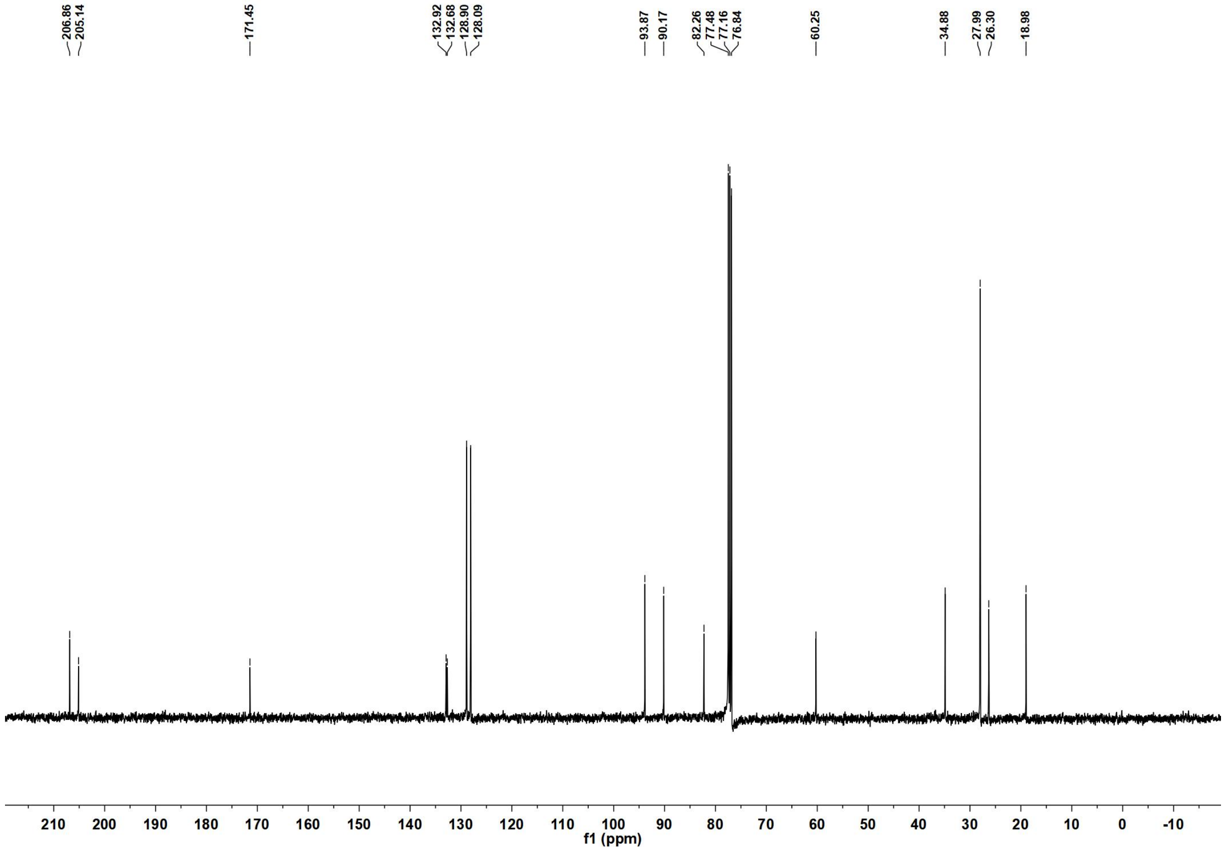

Supplementary Fig. 18. ^{13}C NMR spectra of compound **3h**. ^{13}C NMR (101 MHz, 298K) in $CDCl_3$

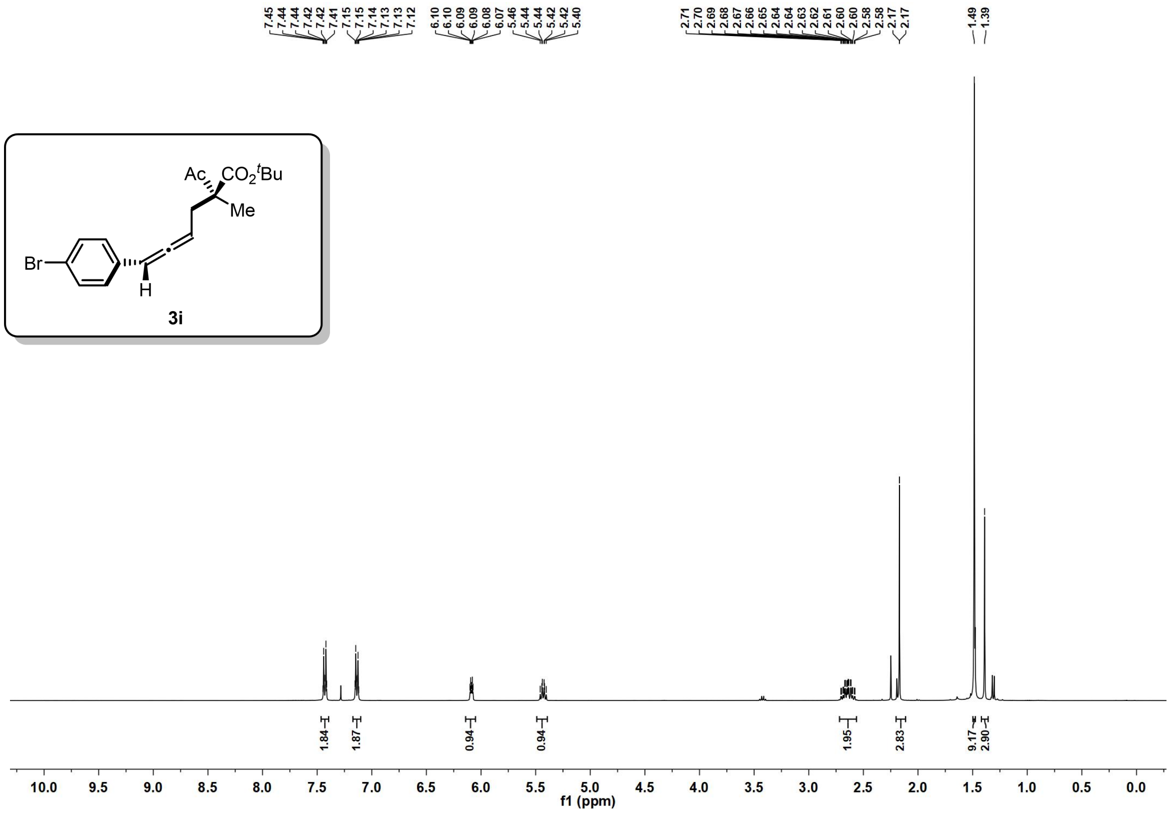

Supplementary Fig. 19. ^{1}H NMR spectra of compound **3i**. ^{1}H NMR (400 MHz, 298K) in $CDCl_3$

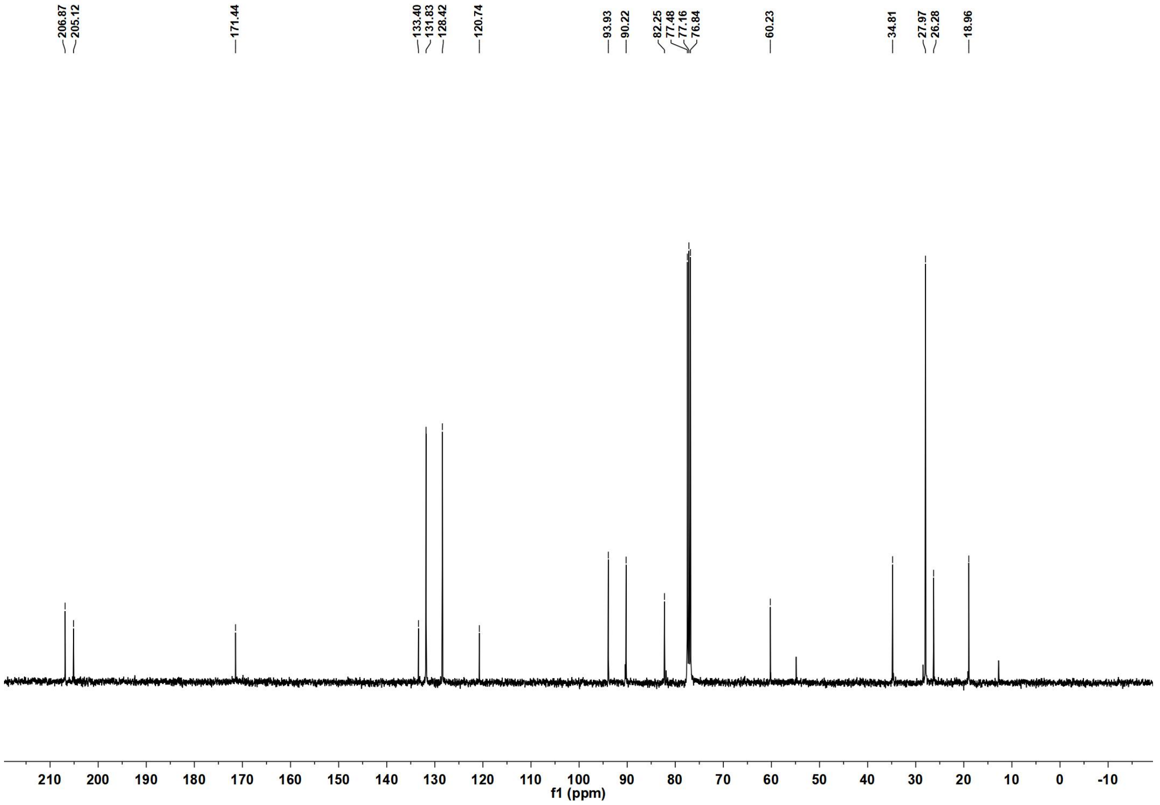

Supplementary Fig. 20. ^{13}C NMR spectra of compound **3i**. ^{13}C NMR (101 MHz, 298K) in $CDCl_3$

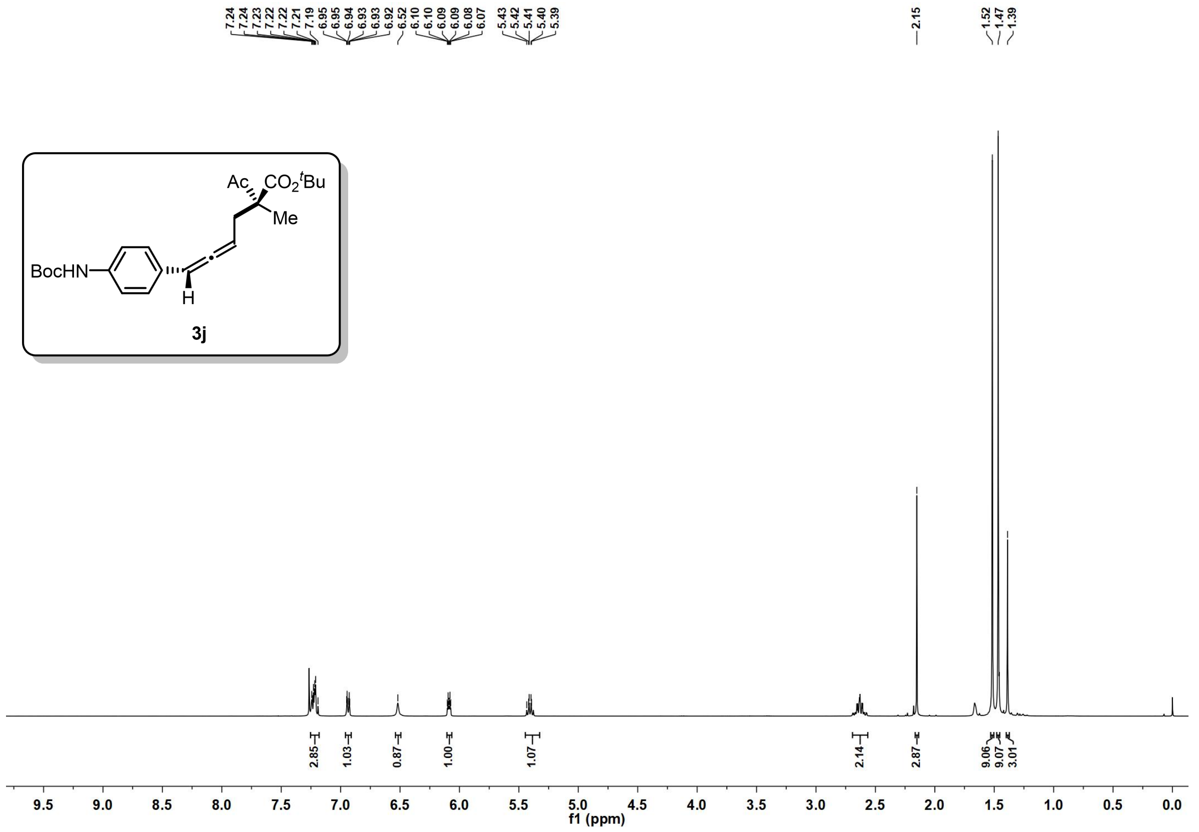

Supplementary Fig. 21. ^{1}H NMR spectra of compound **3j**. ^{1}H NMR (400 MHz, 298K) in $CDCl_3$

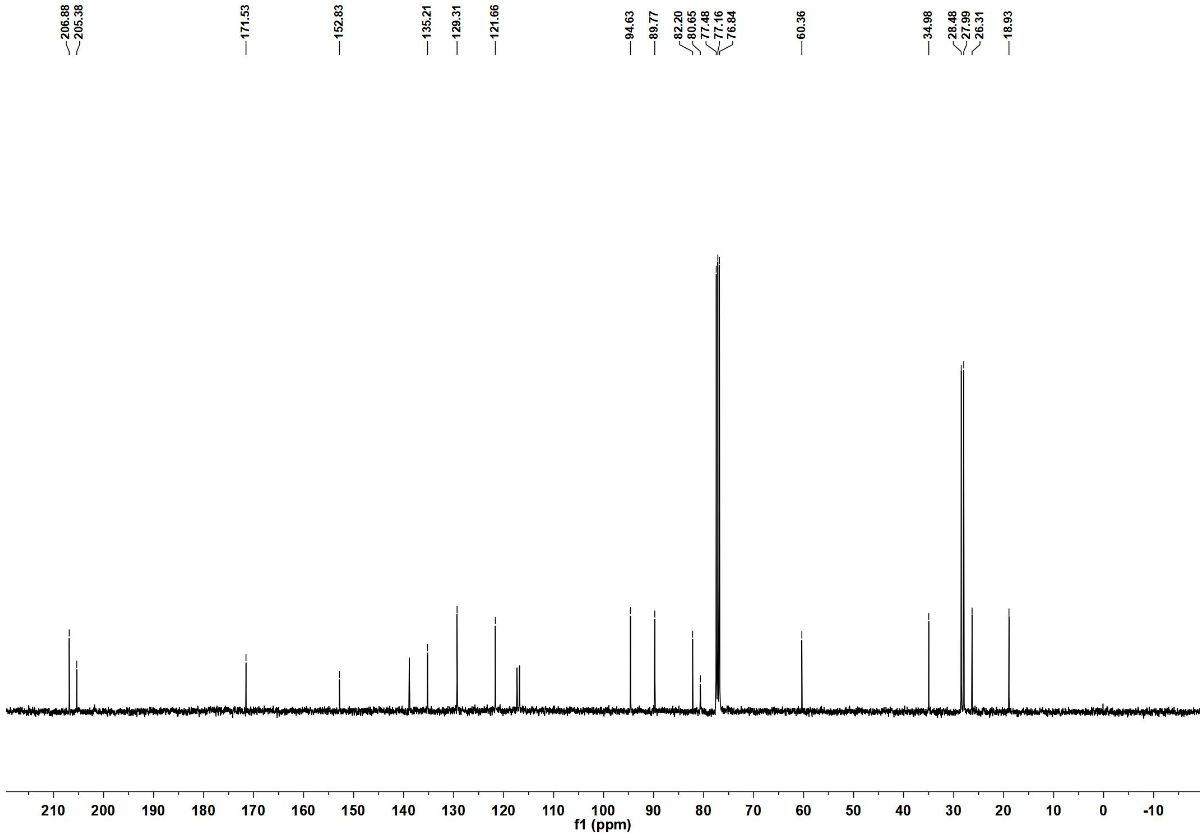

Supplementary Fig. 22. ^{13}C NMR spectra of compound **3j**. ^{13}C NMR (101 MHz, 298K) in $CDCl_3$

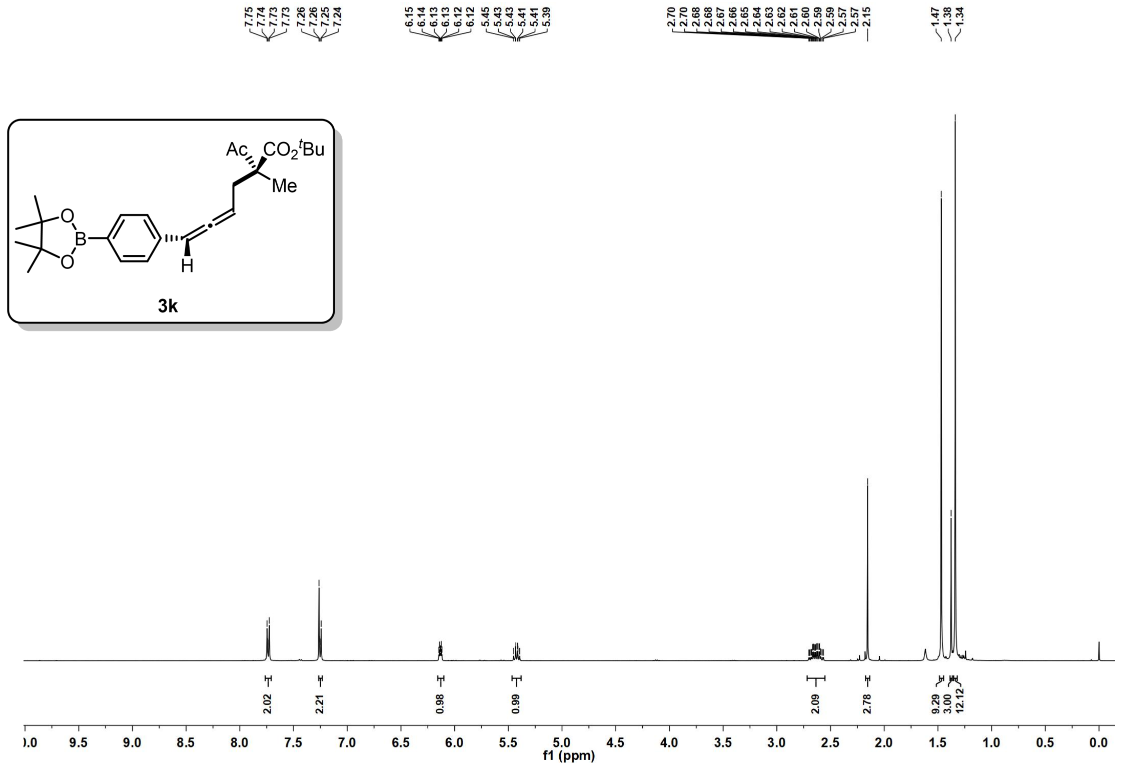

Supplementary Fig. 23. ^{1}H NMR spectra of compound **3k**. ^{1}H NMR (400 MHz, 298K) in $CDCl_3$

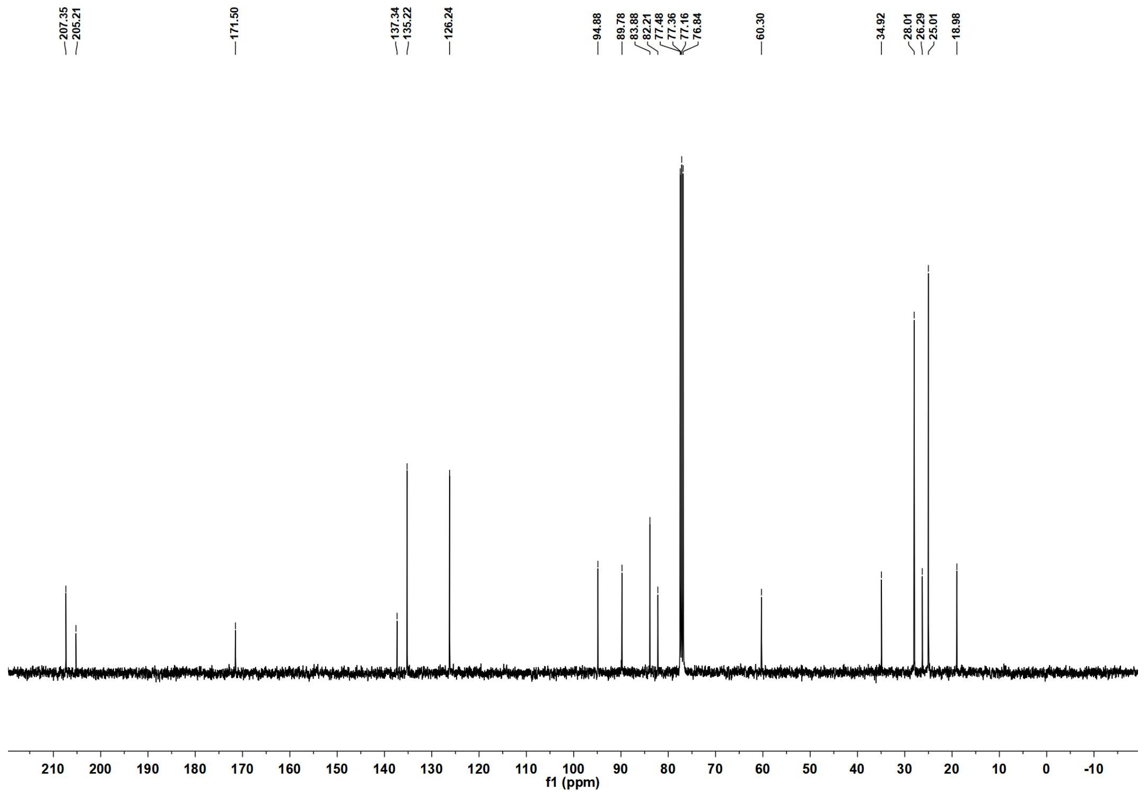

Supplementary Fig. 24. ^{13}C NMR spectra of compound **3k**. ^{13}C NMR (101 MHz, 298K) in $CDCl_3$

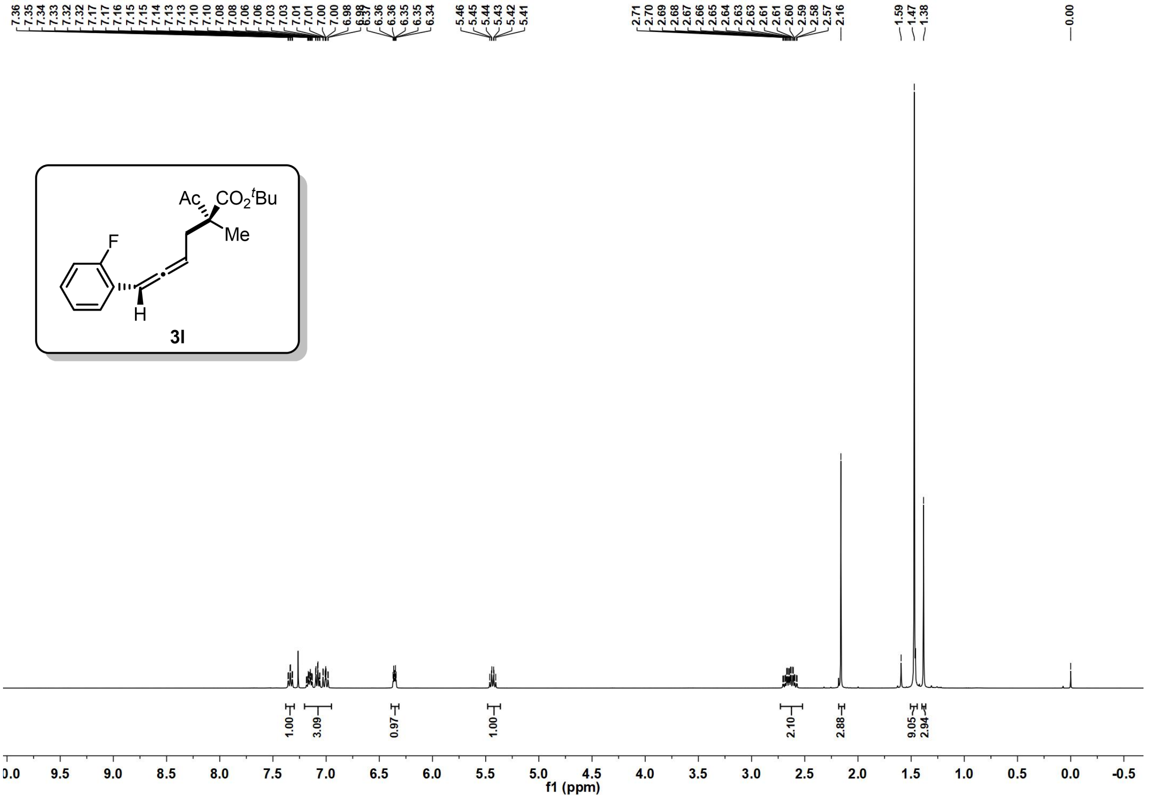

Supplementary Fig. 25. ^{1}H NMR spectra of compound **3l**. ^{1}H NMR (400 MHz, 298K) in $CDCl_3$

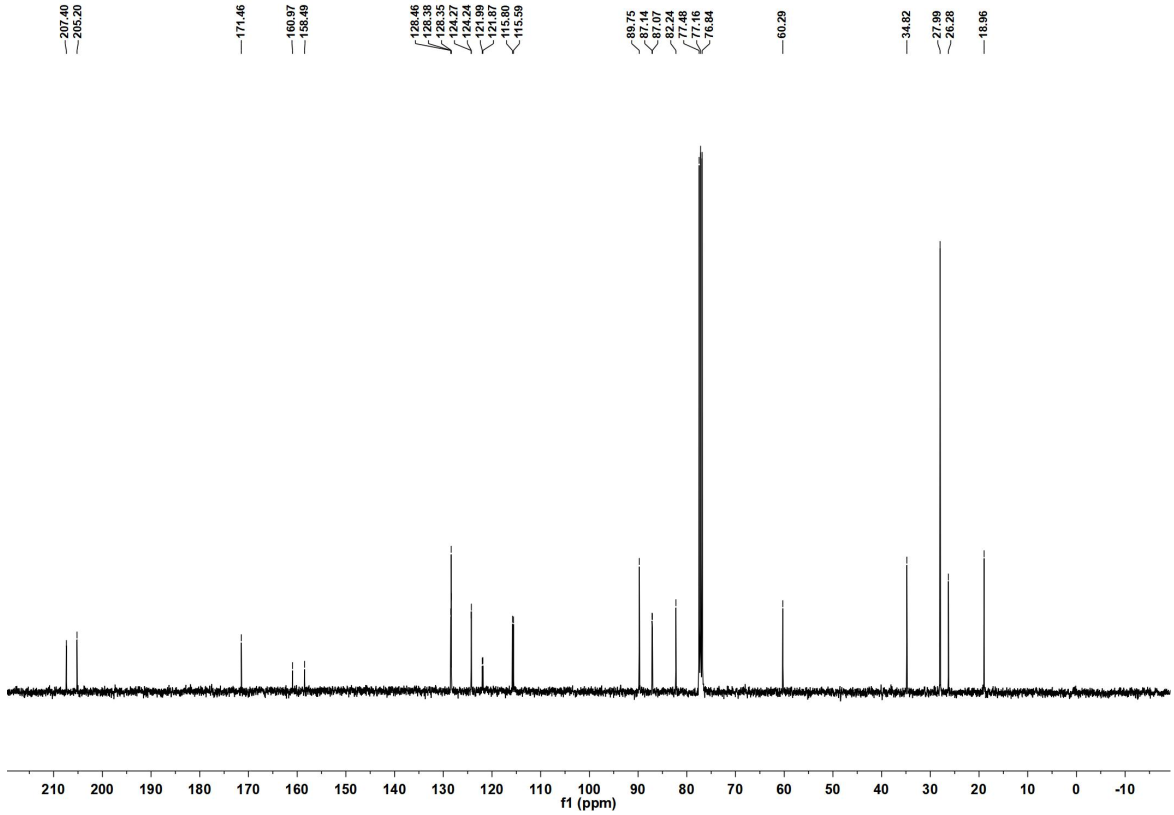

Supplementary Fig. 26. ^{1}H NMR spectra of compound **3l**. ^{1}H NMR (400 MHz, 298K) in $CDCl_3$

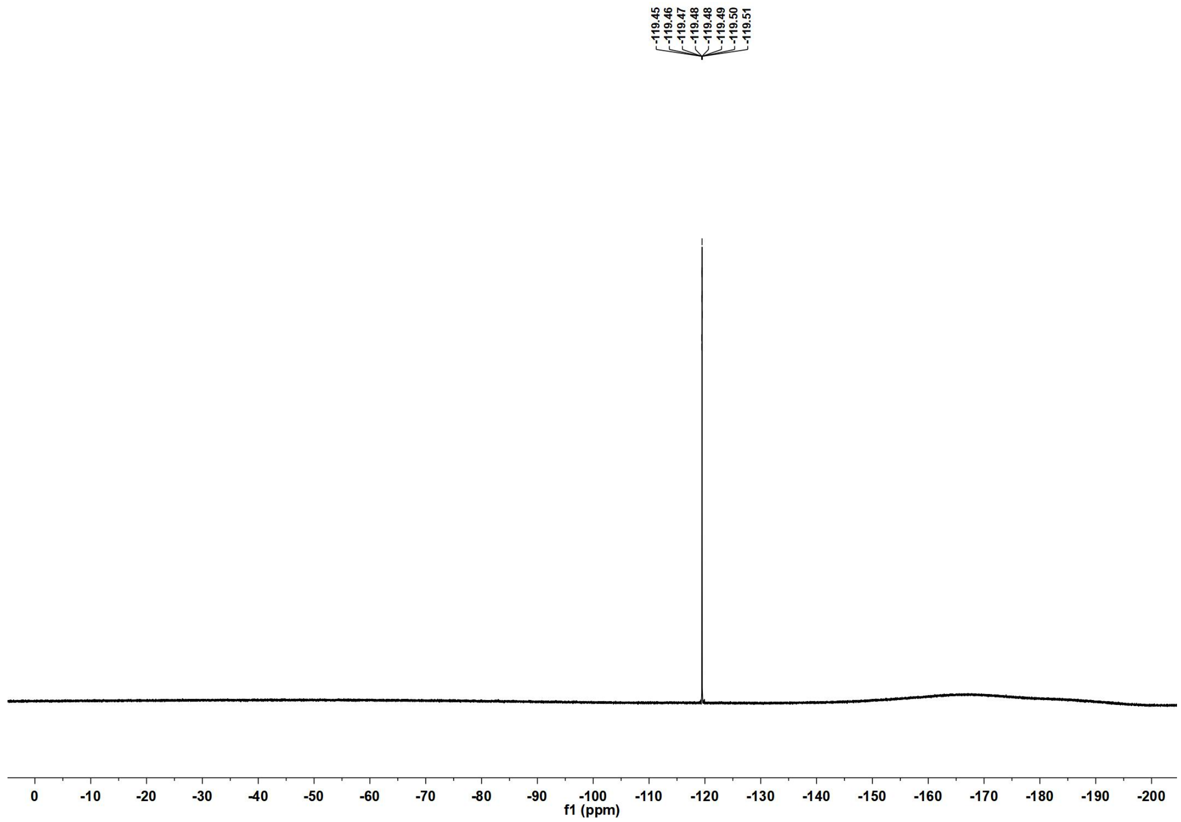

Supplementary Fig. 27. ^{19}F NMR spectra of compound **3l**. ^{19}F NMR (376 MHz, 298K) in $CDCl_3$

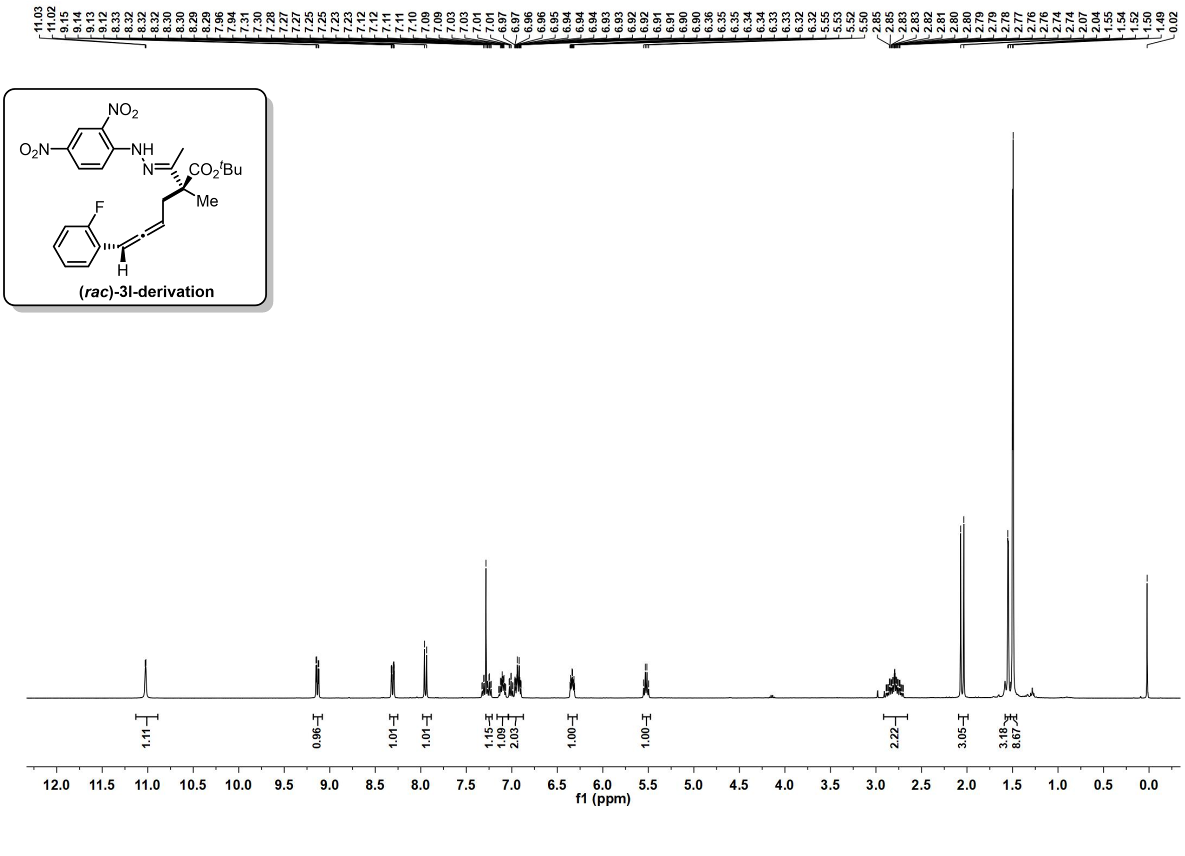

Supplementary Fig. 28. ^{1}H NMR spectra of compound **(*rac*)-3l-derivation**. ^{1}H NMR (400 MHz, 298K) in $CDCl_3$

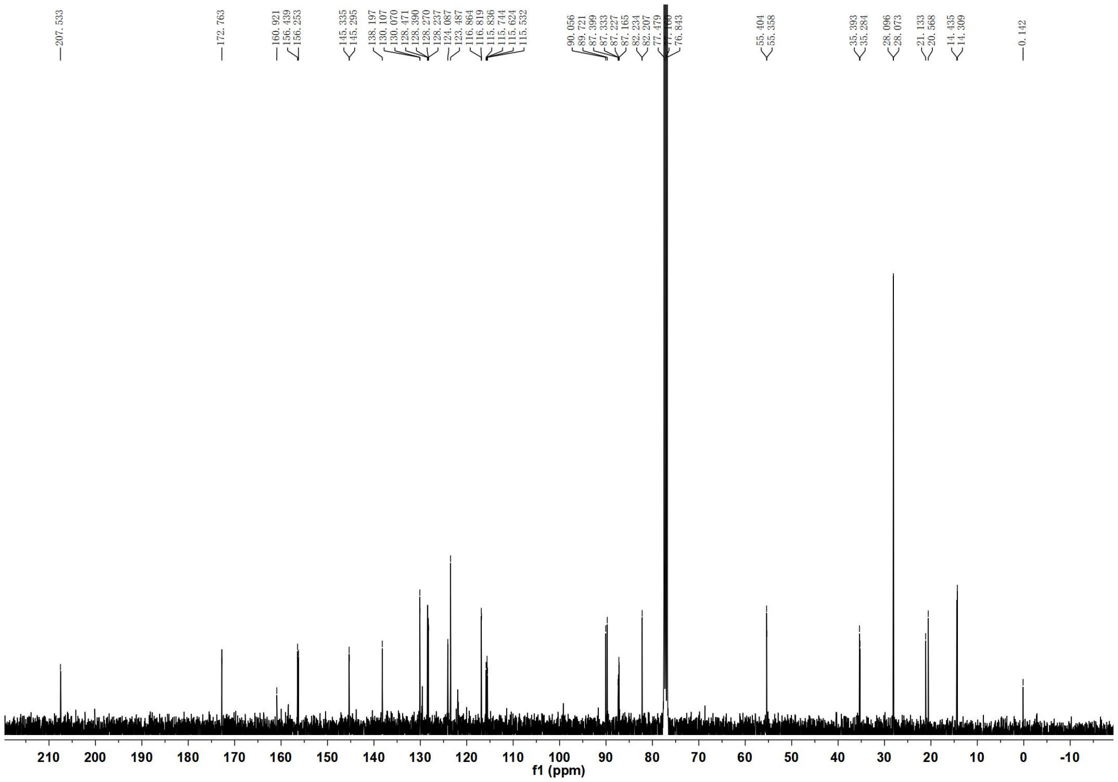

Supplementary Fig. 29. ^{1}H NMR spectra of compound **(*rac*)-3l-derivation**. ^{1}H NMR (400 MHz, 298K) in $CDCl_3$

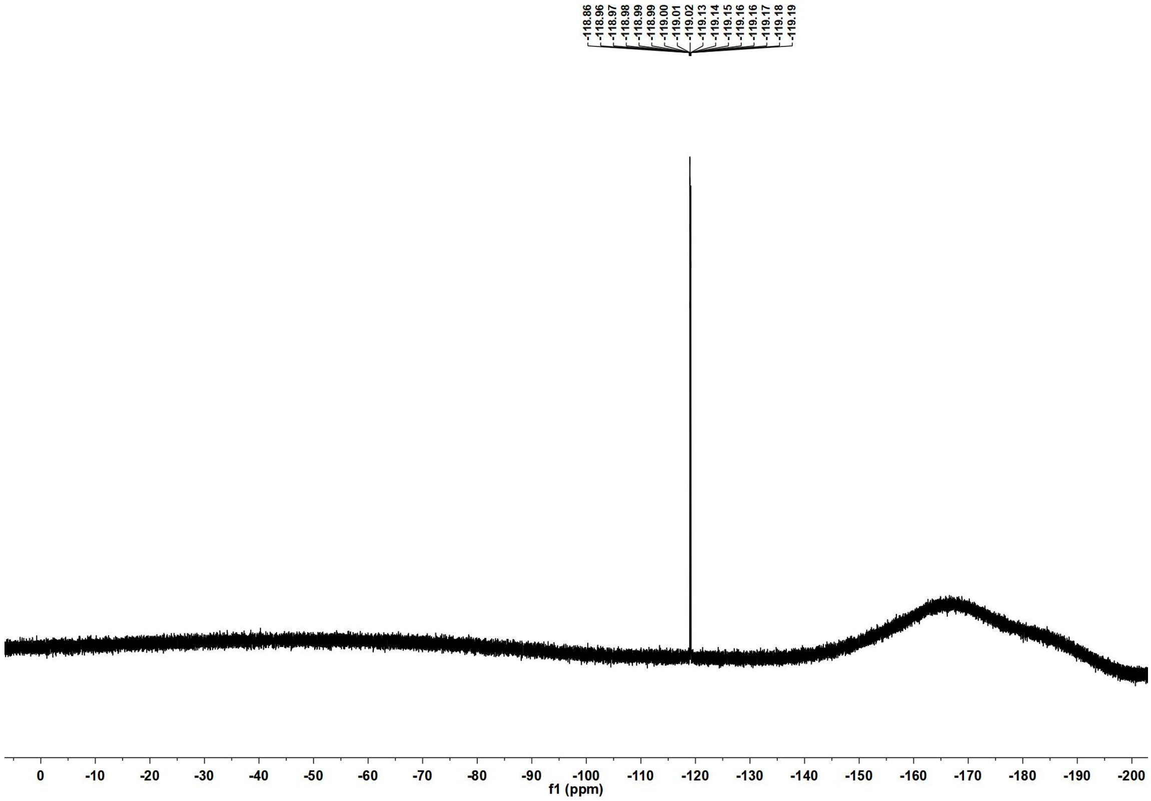

Supplementary Fig. 30. ^{19}F NMR spectra of compound **(*rac*)-3l-derivation**. ^{19}F NMR (376 MHz, 298K) in $CDCl_3$

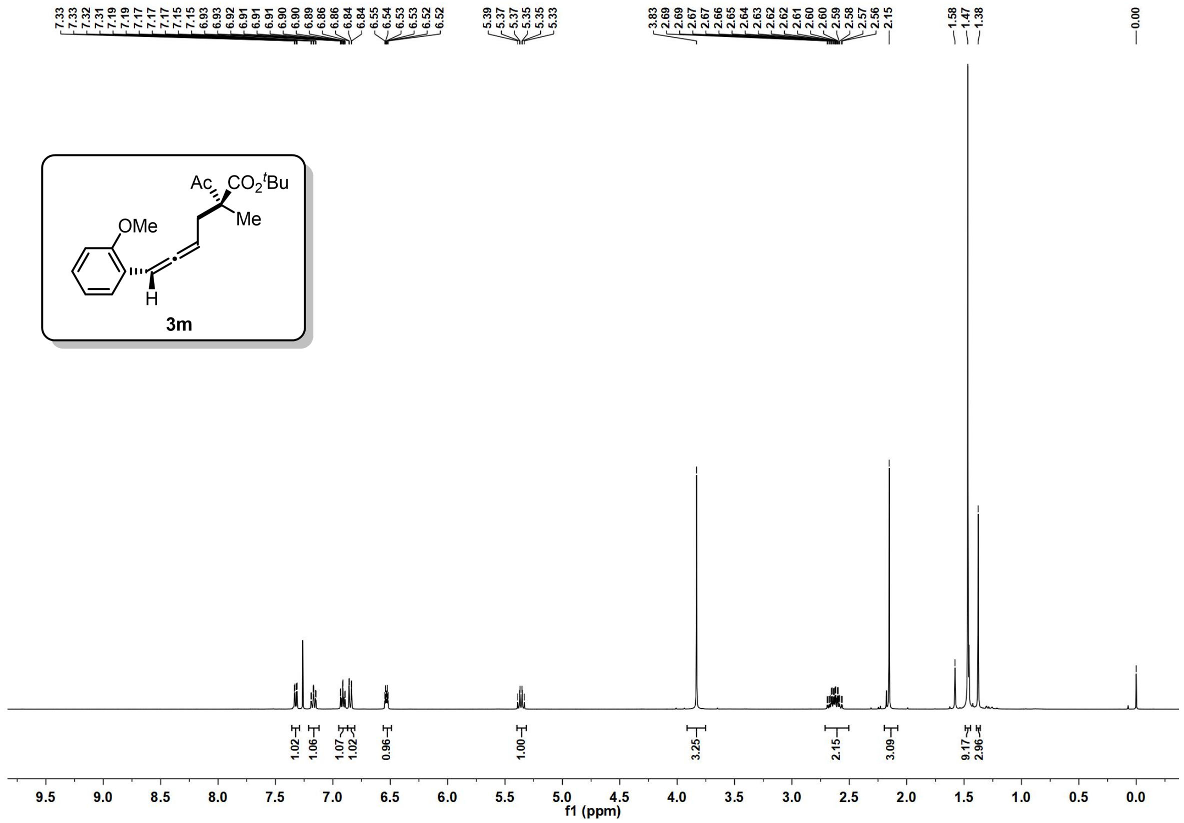

Supplementary Fig. 31. ^{1}H NMR spectra of compound **3m**. ^{1}H NMR (400 MHz, 298K) in $CDCl_3$

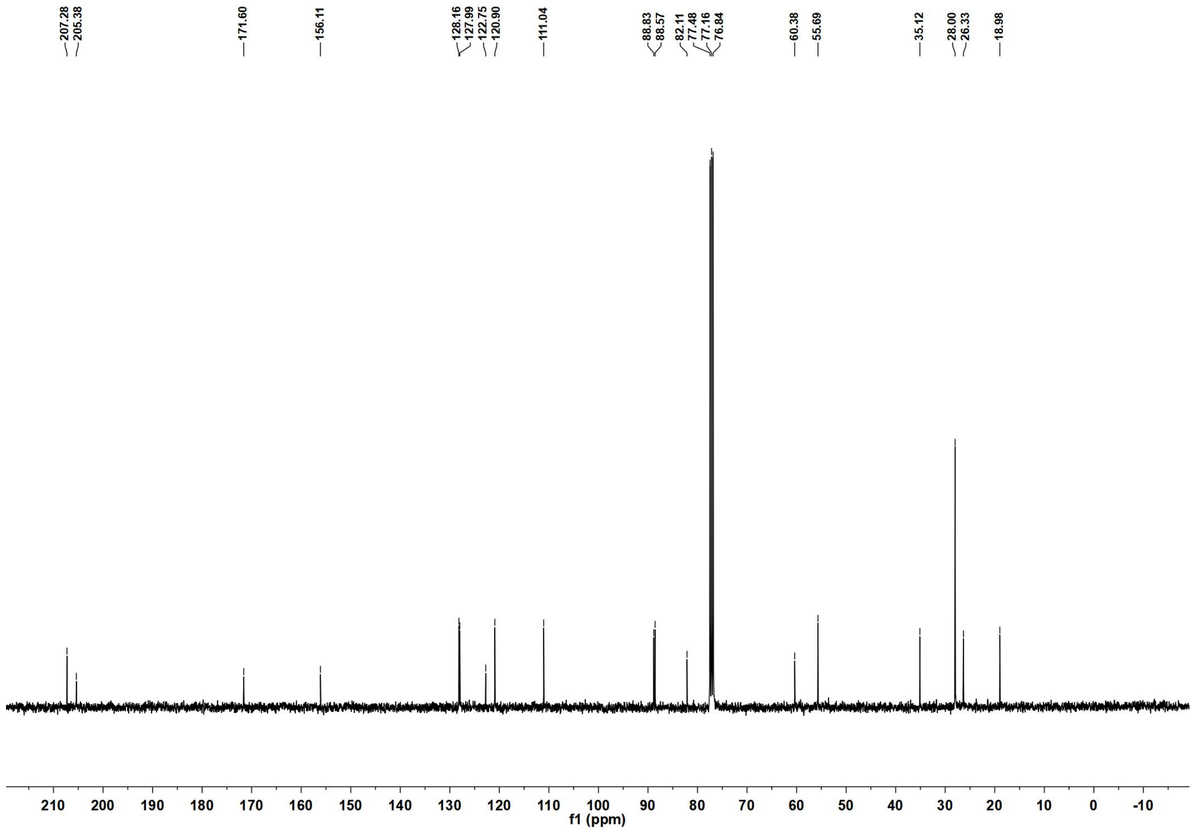

Supplementary Fig. 32. ^{13}C NMR spectra of compound **3m**. ^{13}C NMR (101 MHz, 298K) in $CDCl_3$

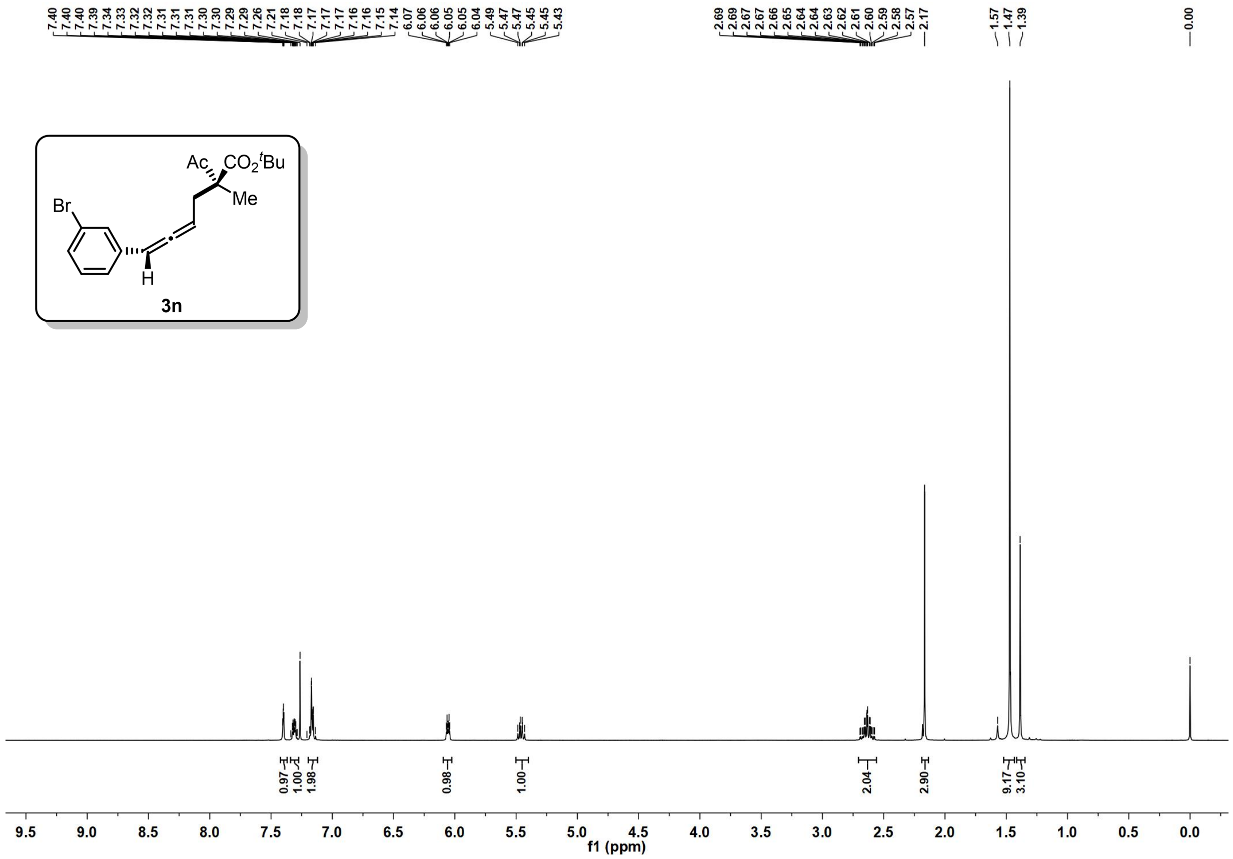

Supplementary Fig. 33. ^{1}H NMR spectra of compound **3n**. ^{1}H NMR (400 MHz, 298K) in $CDCl_3$

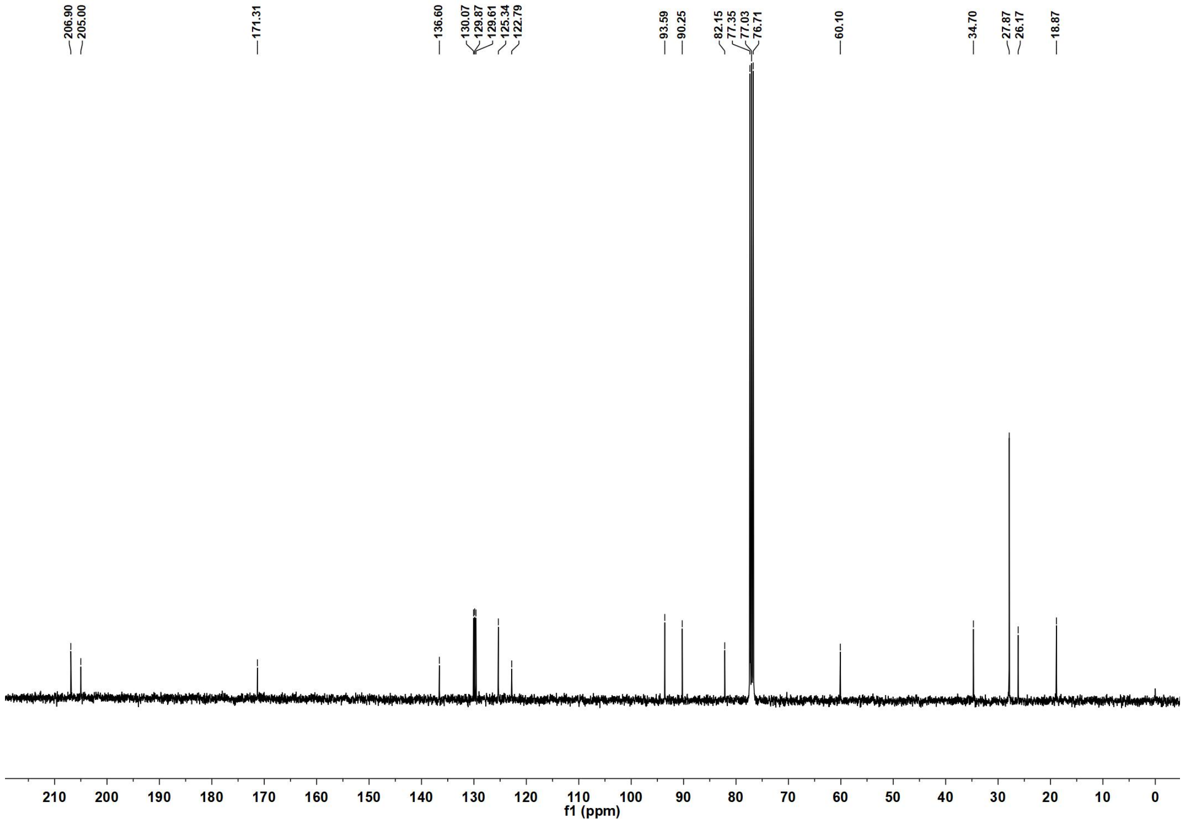

Supplementary Fig. 34. ^{13}C NMR spectra of compound **3n**. ^{13}C NMR (101 MHz, 298K) in $CDCl_3$

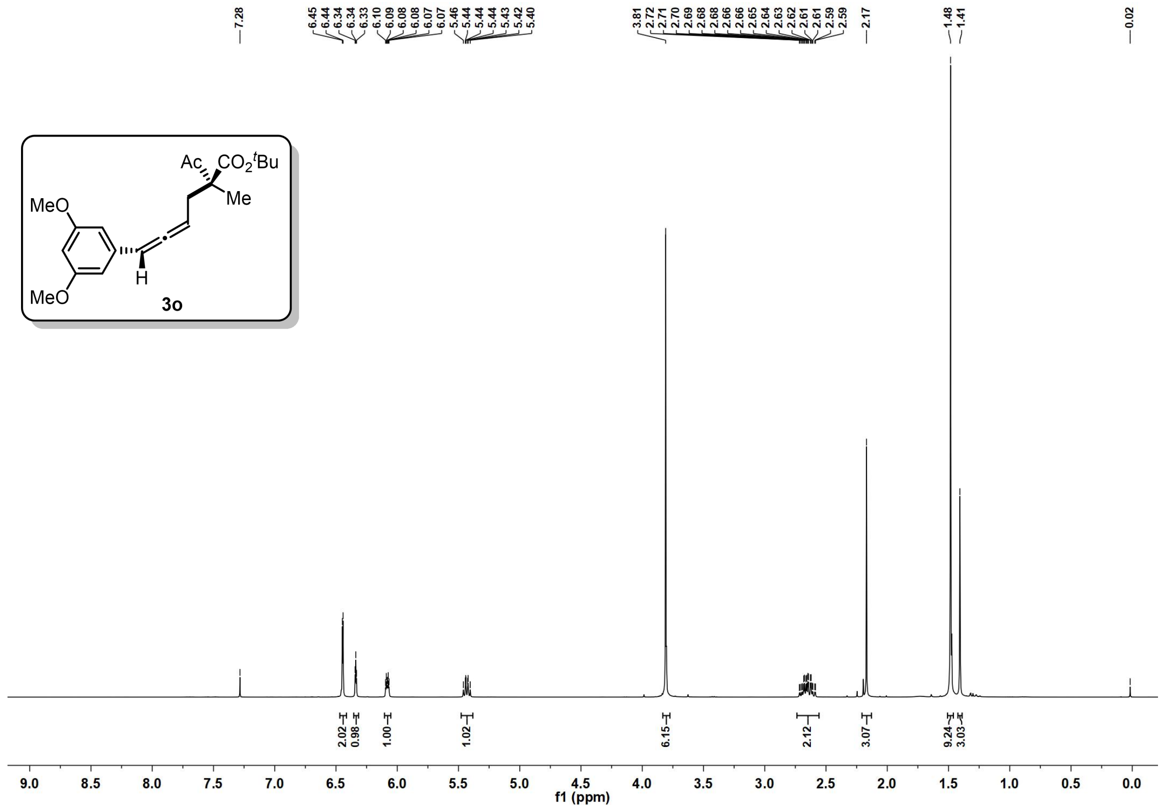

Supplementary Fig. 35. ^{1}H NMR spectra of compound **3o**. ^{1}H NMR (400 MHz, 298K) in $CDCl_3$

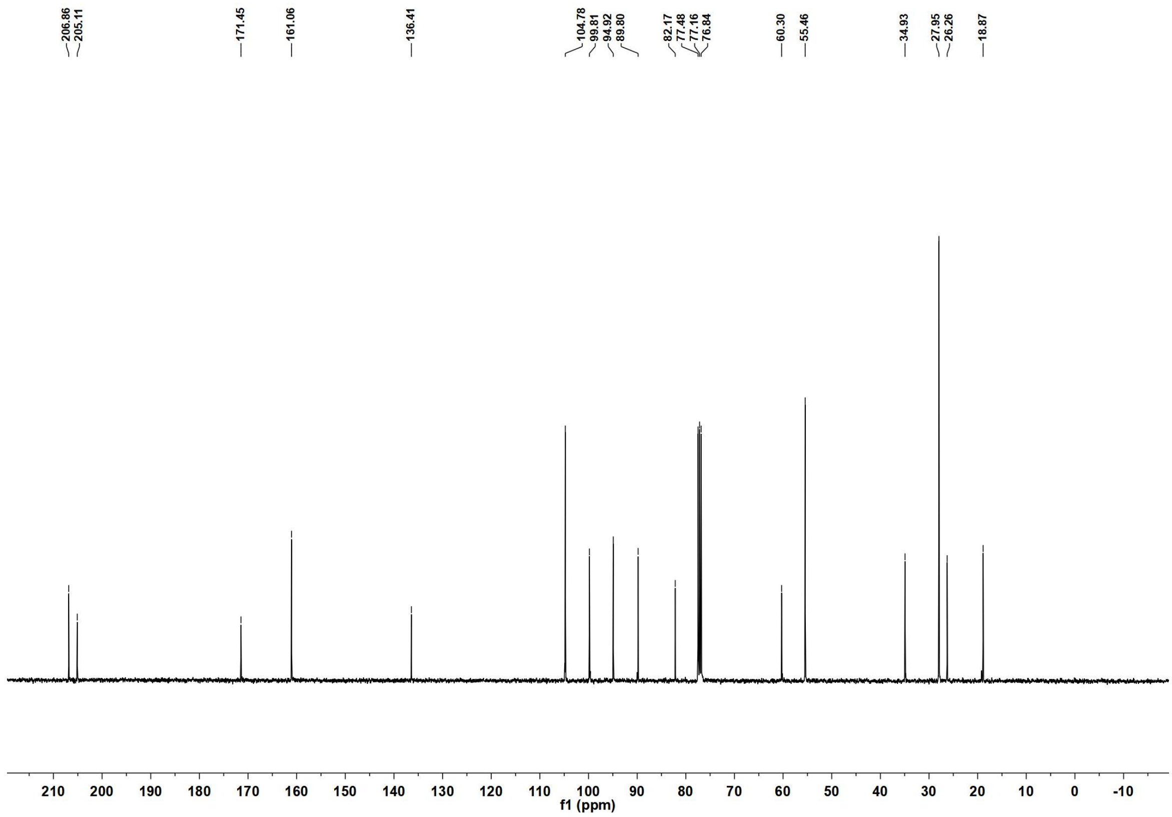

Supplementary Fig. 36. ^{13}C NMR spectra of compound **3o**. ^{13}C NMR (101 MHz, 298K) in $CDCl_3$

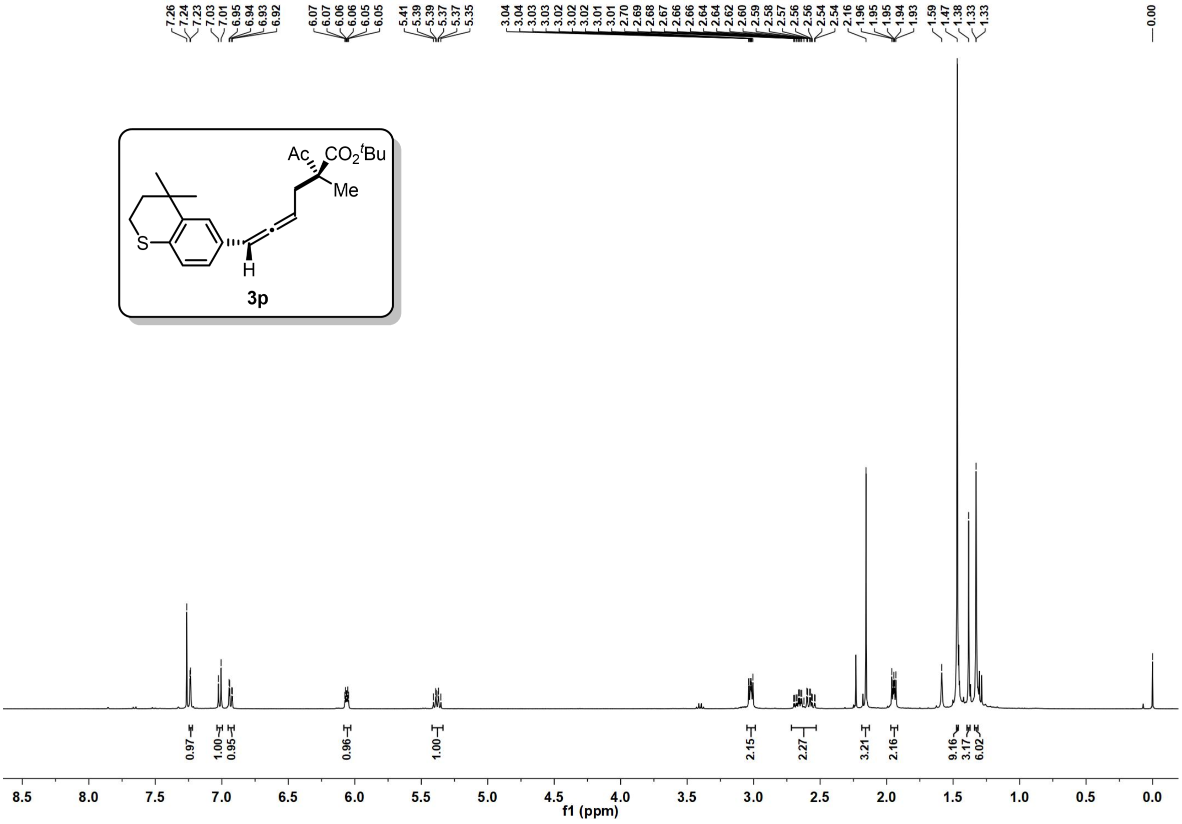

Supplementary Fig. 37. ¹H NMR spectra of compound **3p**. ¹H NMR (400 MHz, 298K) in $CDCl_3$

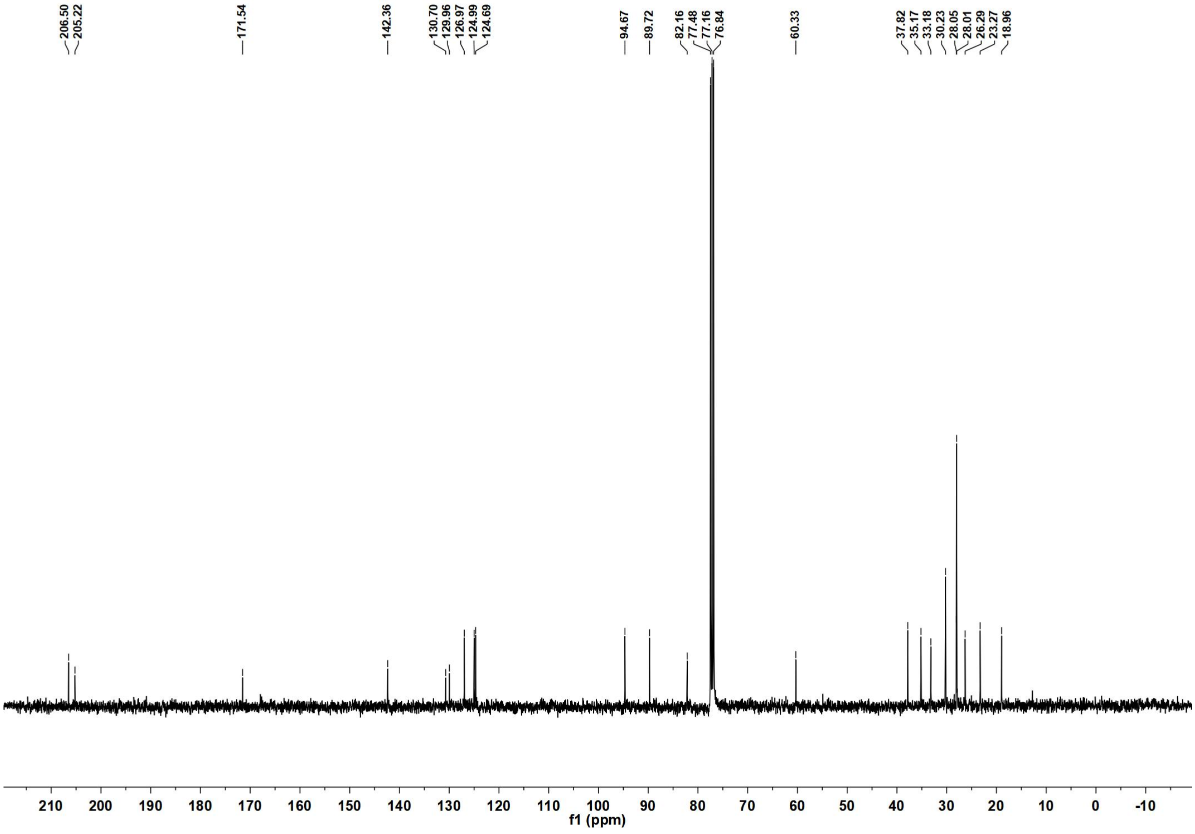

Supplementary Fig. 38. ¹³C NMR spectra of compound **3p**. ¹³C NMR (101 MHz, 298K) in $CDCl_3$

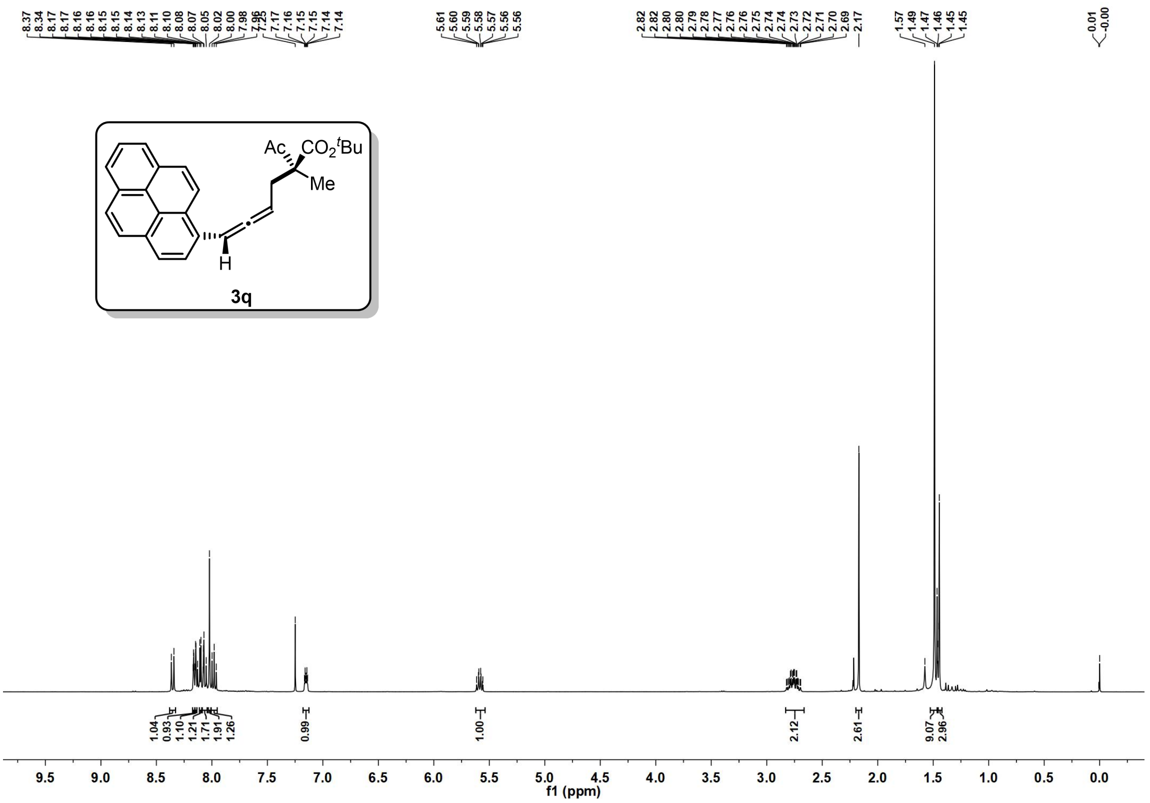

Supplementary Fig. 39. ^{1}H NMR spectra of compound **3q**. ^{1}H NMR (400 MHz, 298K) in $CDCl_3$

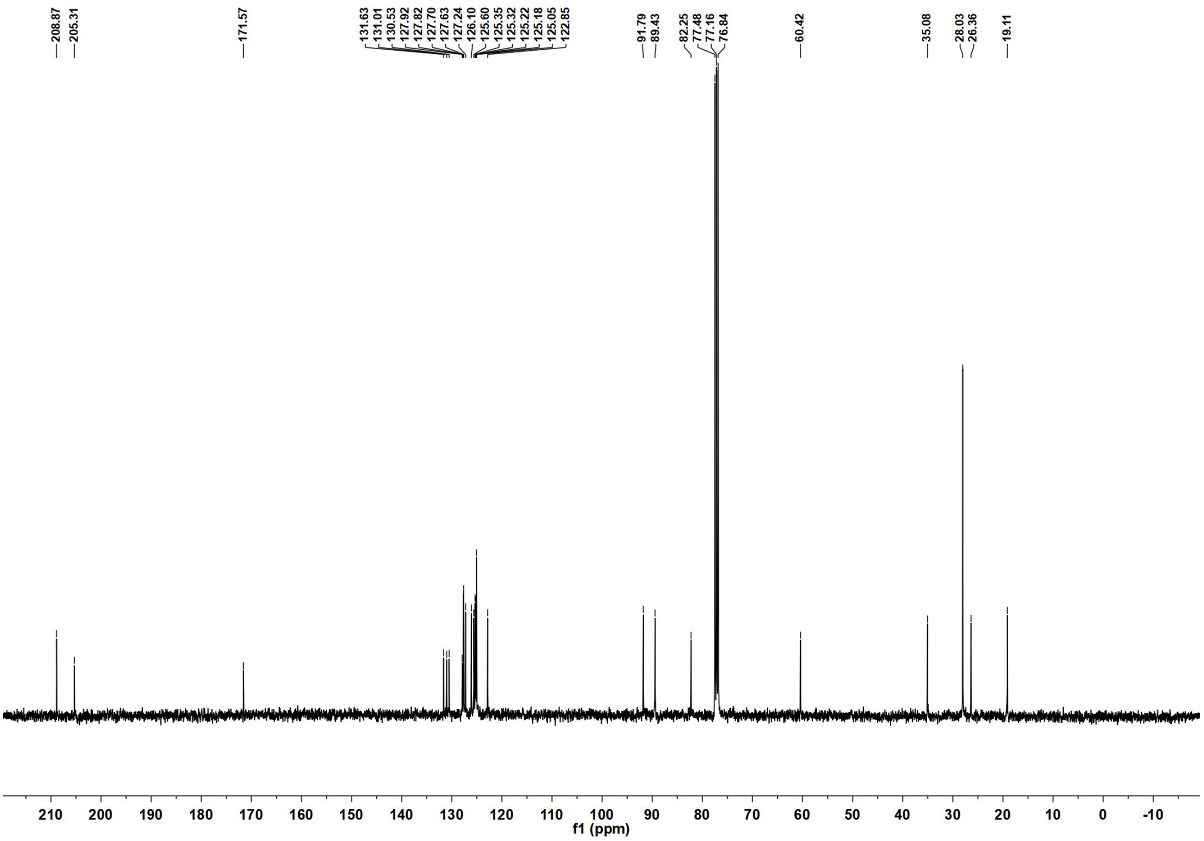

Supplementary Fig. 40. ^{13}C NMR spectra of compound **3q**. ^{13}C NMR (101 MHz, 298K) in $CDCl_3$

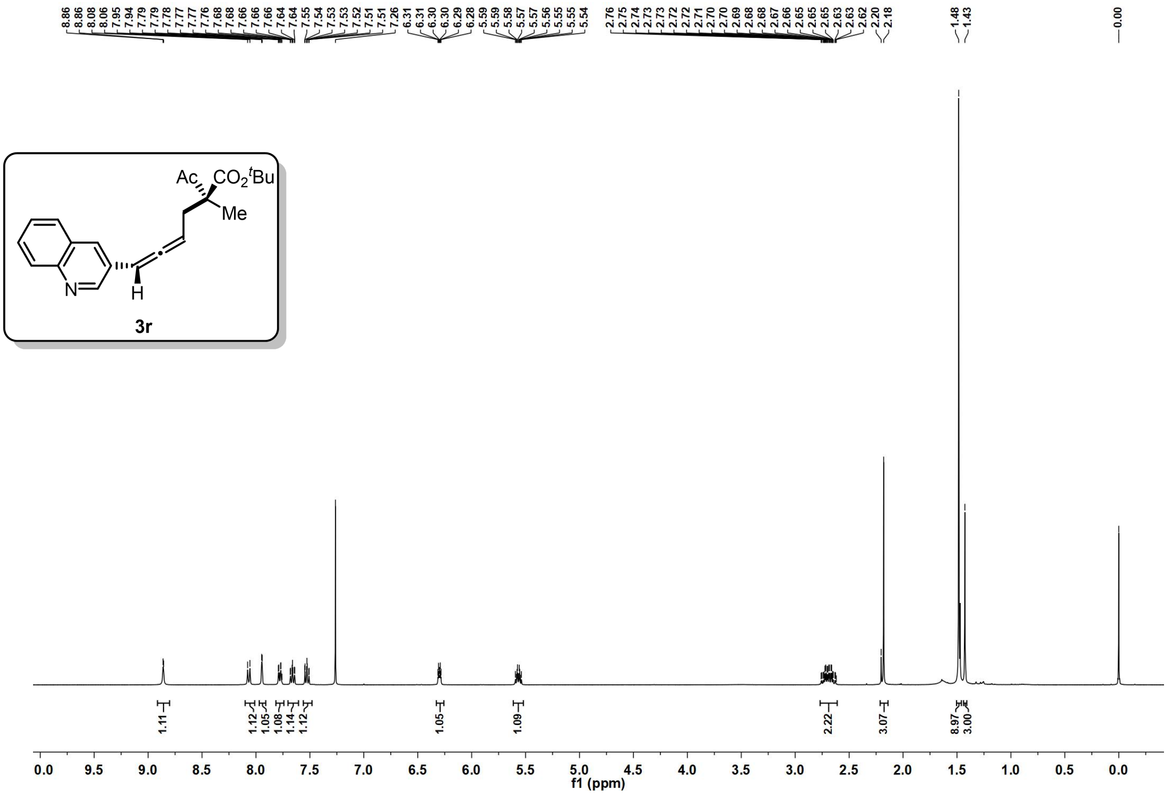

Supplementary Fig. 41. ^{1}H NMR spectra of compound **3r**. ^{1}H NMR (400 MHz, 298K) in $CDCl_3$

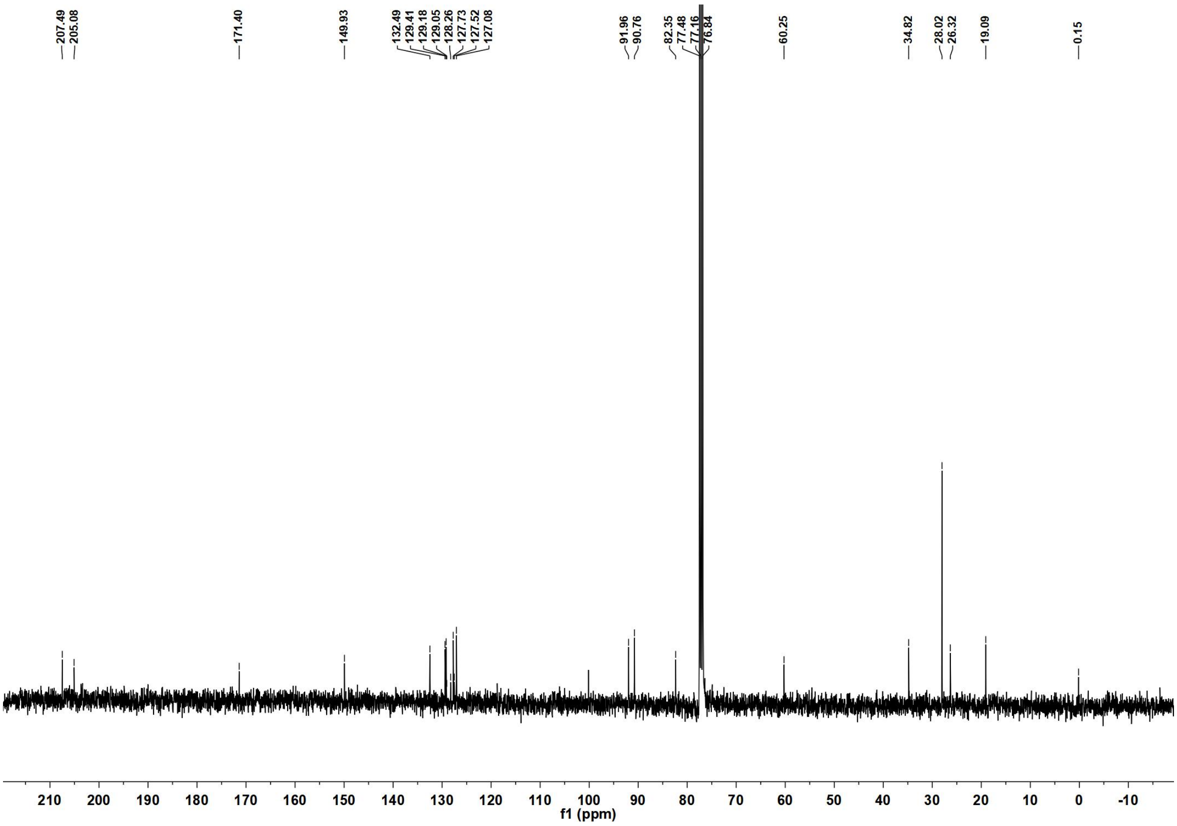

Supplementary Fig. 42. ^{13}C NMR spectra of compound **3r**. ^{13}C NMR (101 MHz, 298K) in $CDCl_3$

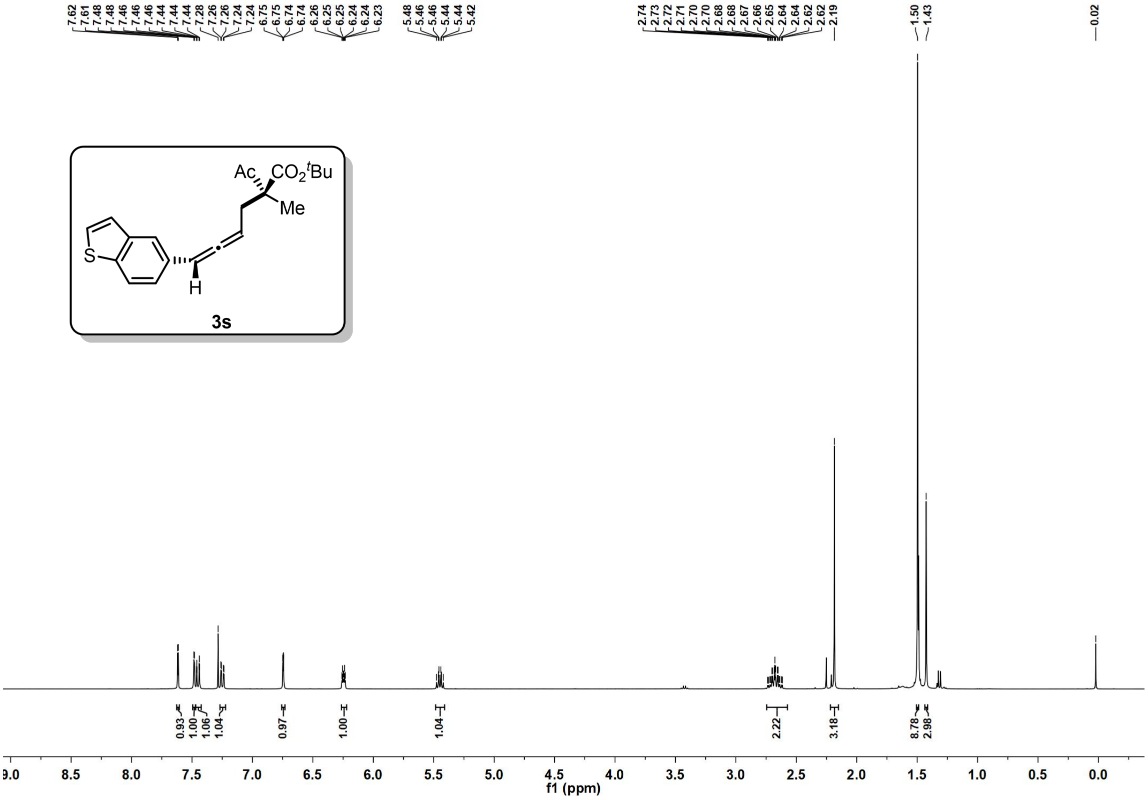

Supplementary Fig. 43. ^{1}H NMR spectra of compound **3s**. ^{1}H NMR (400 MHz, 298K) in $CDCl_3$

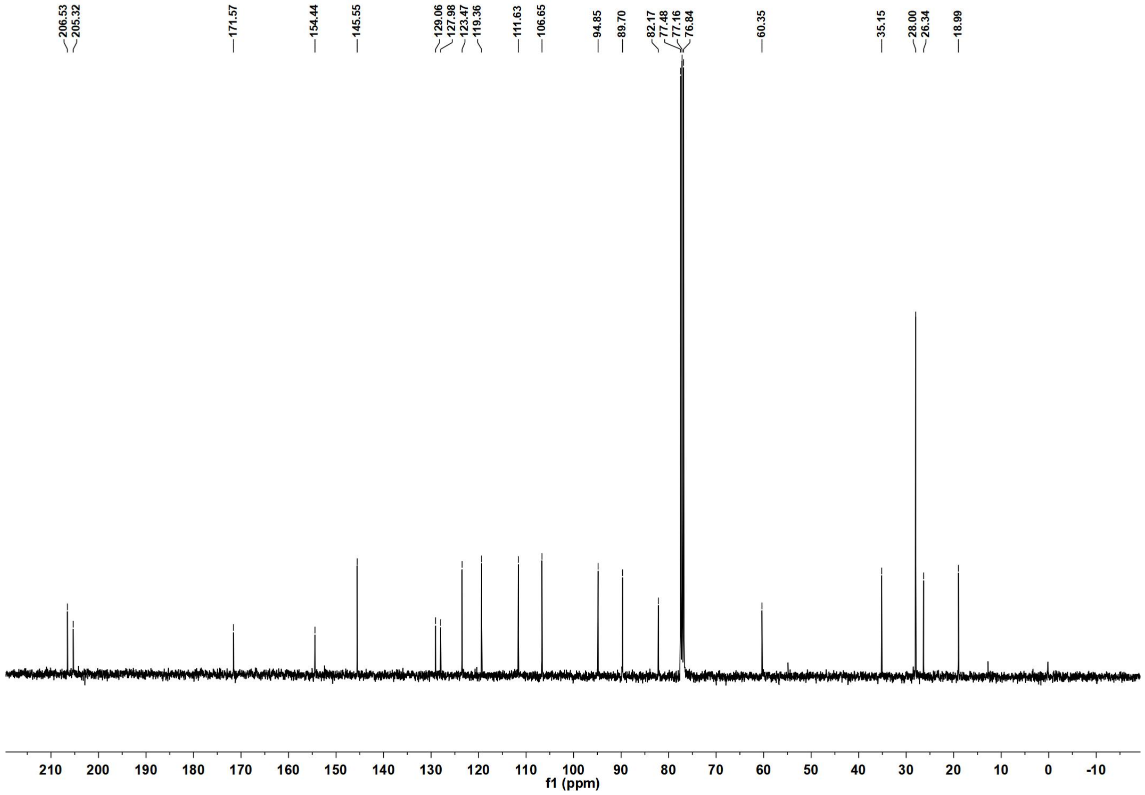

Supplementary Fig. 44. ^{13}C NMR spectra of compound **3s**. ^{13}C NMR (101 MHz, 298K) in $CDCl_3$

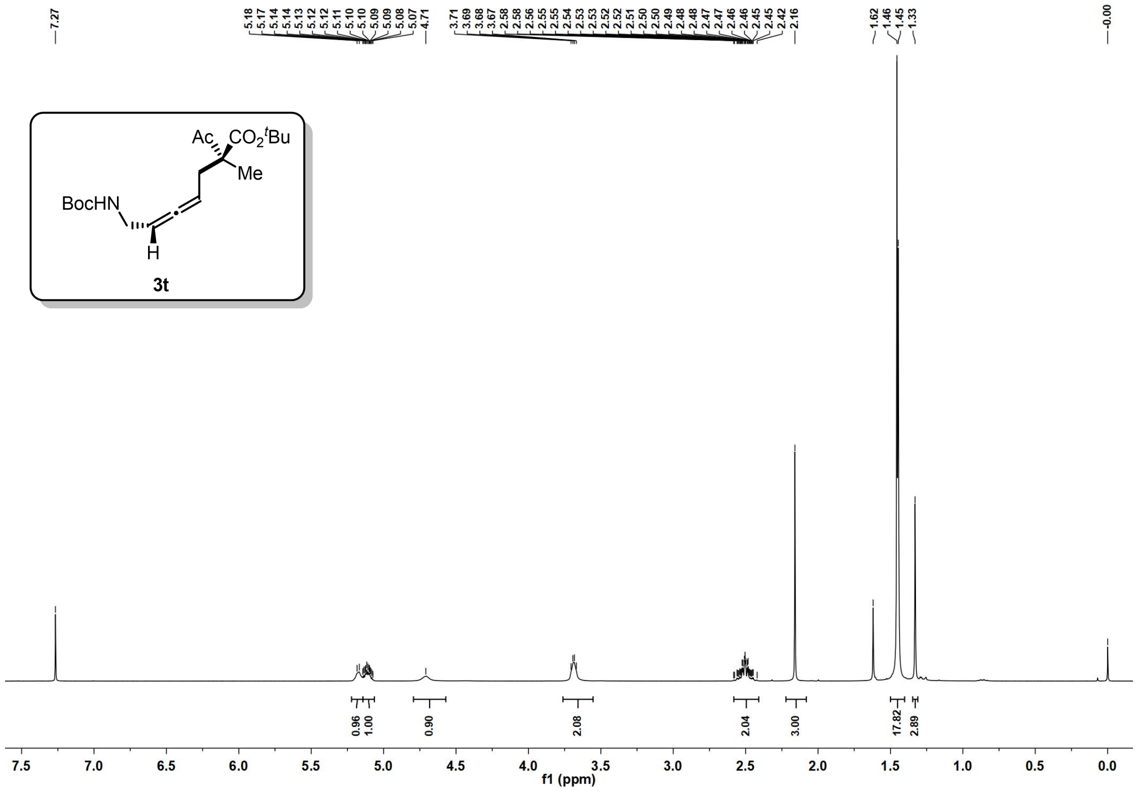

Supplementary Fig. 45. ^{1}H NMR spectra of compound **3t**. ^{1}H NMR (400 MHz, 298K) in $CDCl_3$

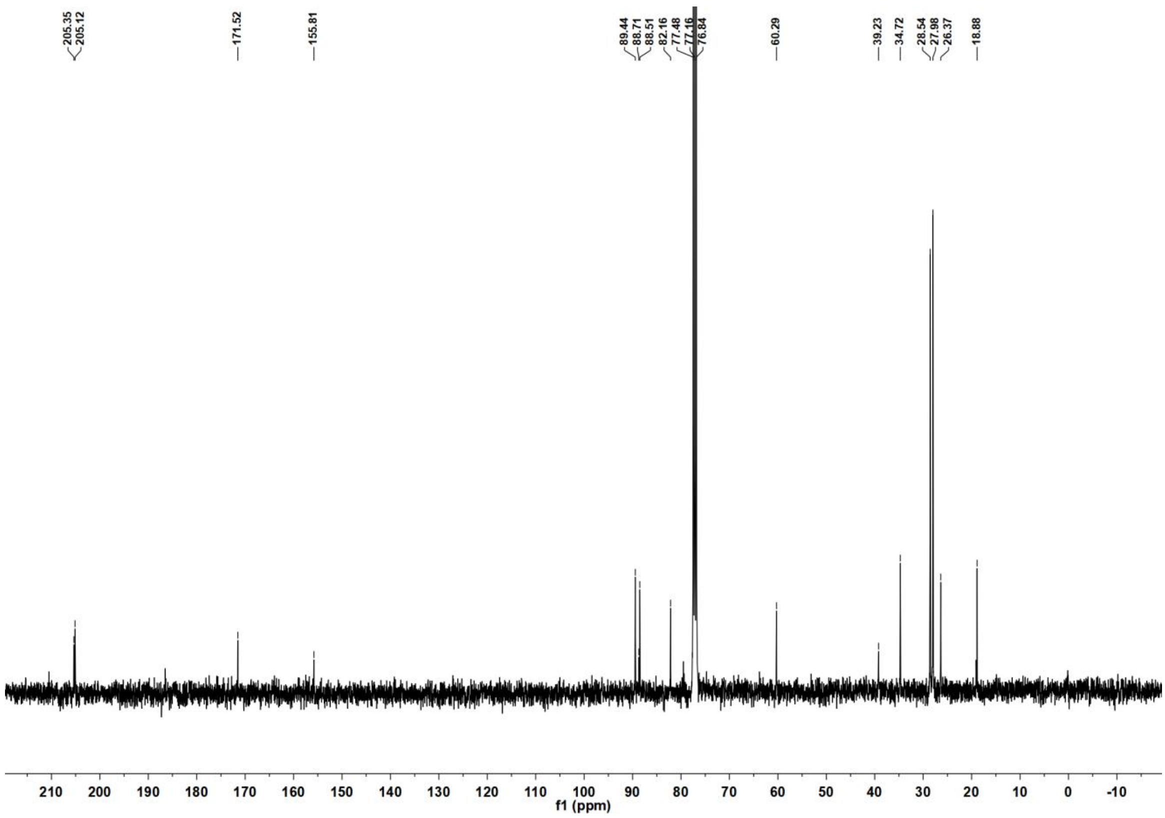

Supplementary Fig. 46. ^{13}C NMR spectra of compound **3t**. ^{13}C NMR (101 MHz, 298K) in $CDCl_3$

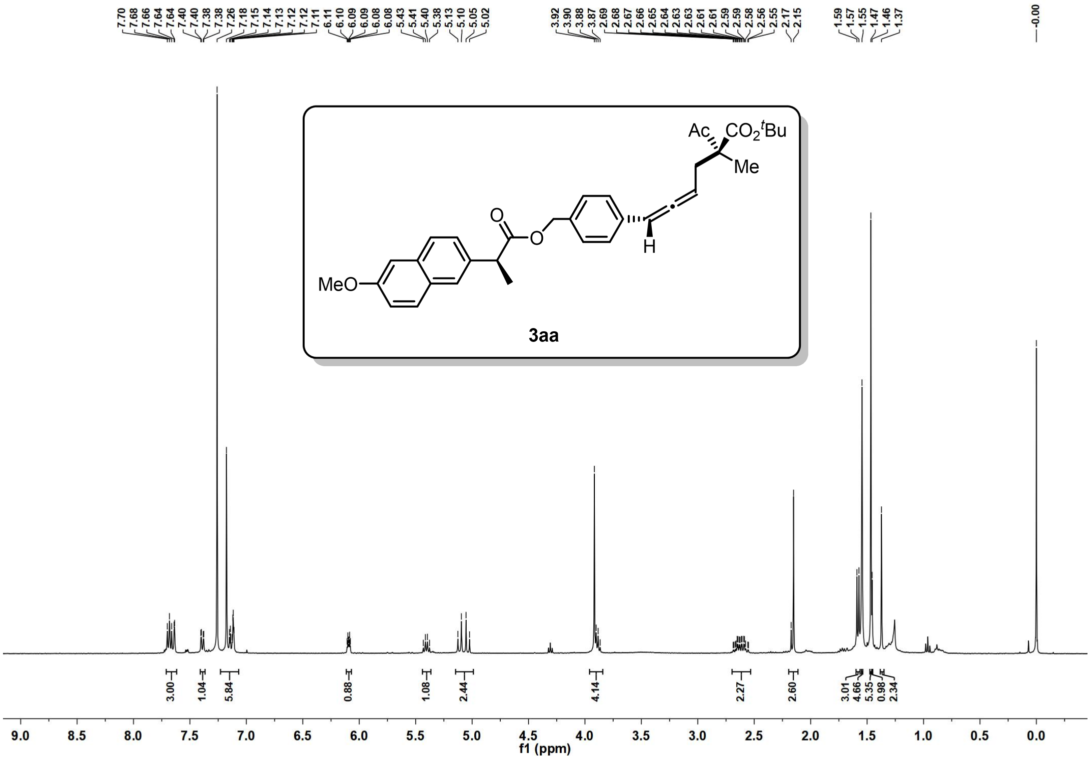

Supplementary Fig. 47. ^{1}H NMR spectra of compound **3aa**. ^{1}H NMR (400 MHz, 298K) in $CDCl_3$

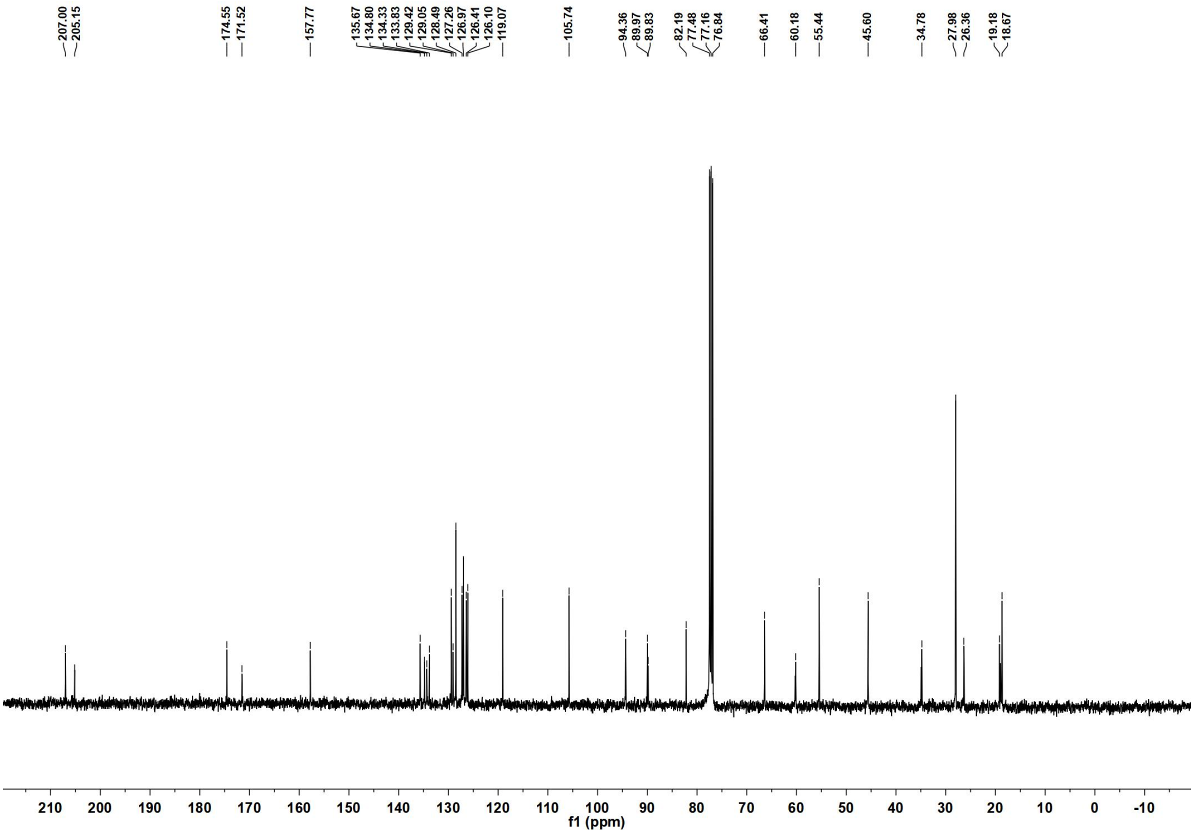

Supplementary Fig. 48. ^{13}C NMR spectra of compound **3aa**. ^{13}C NMR (101 MHz, 298K) in $CDCl_3$

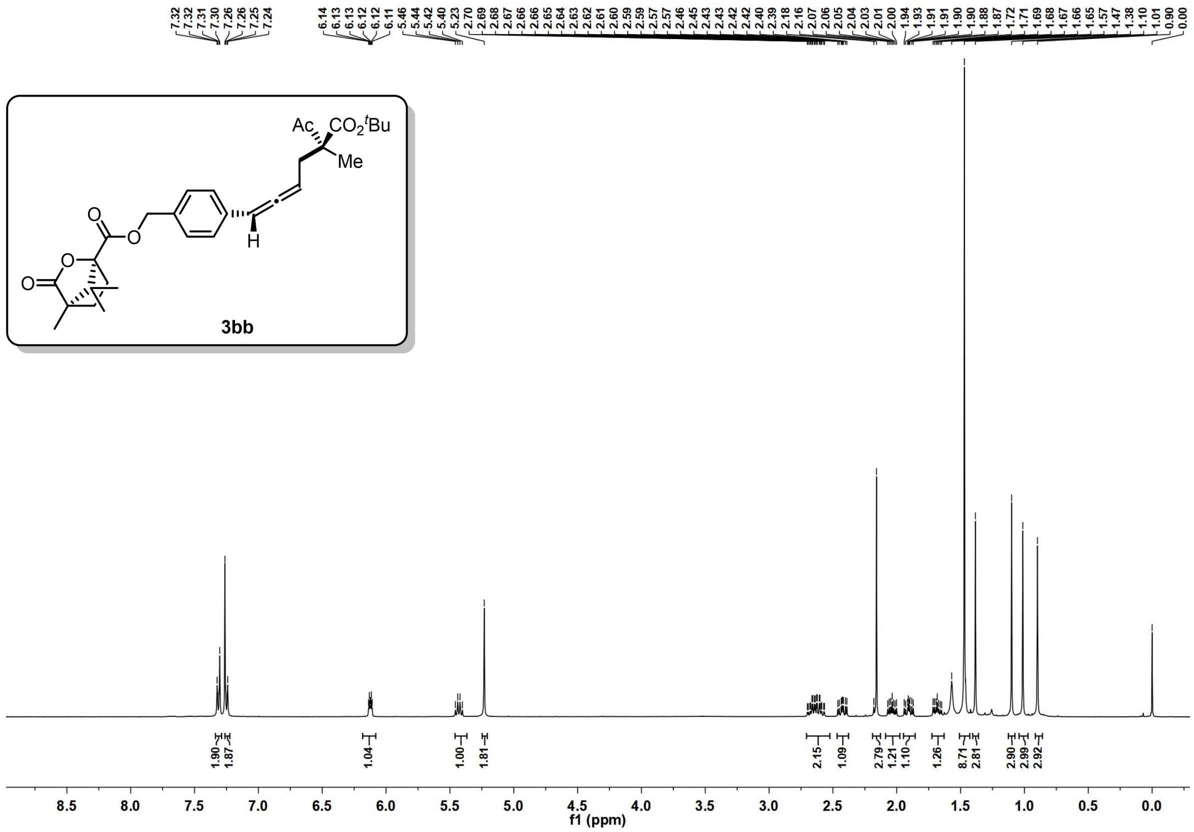

Supplementary Fig. 49. ^{1}H NMR spectra of compound **3bb**. ^{1}H NMR (400 MHz, 298K) in $CDCl_3$

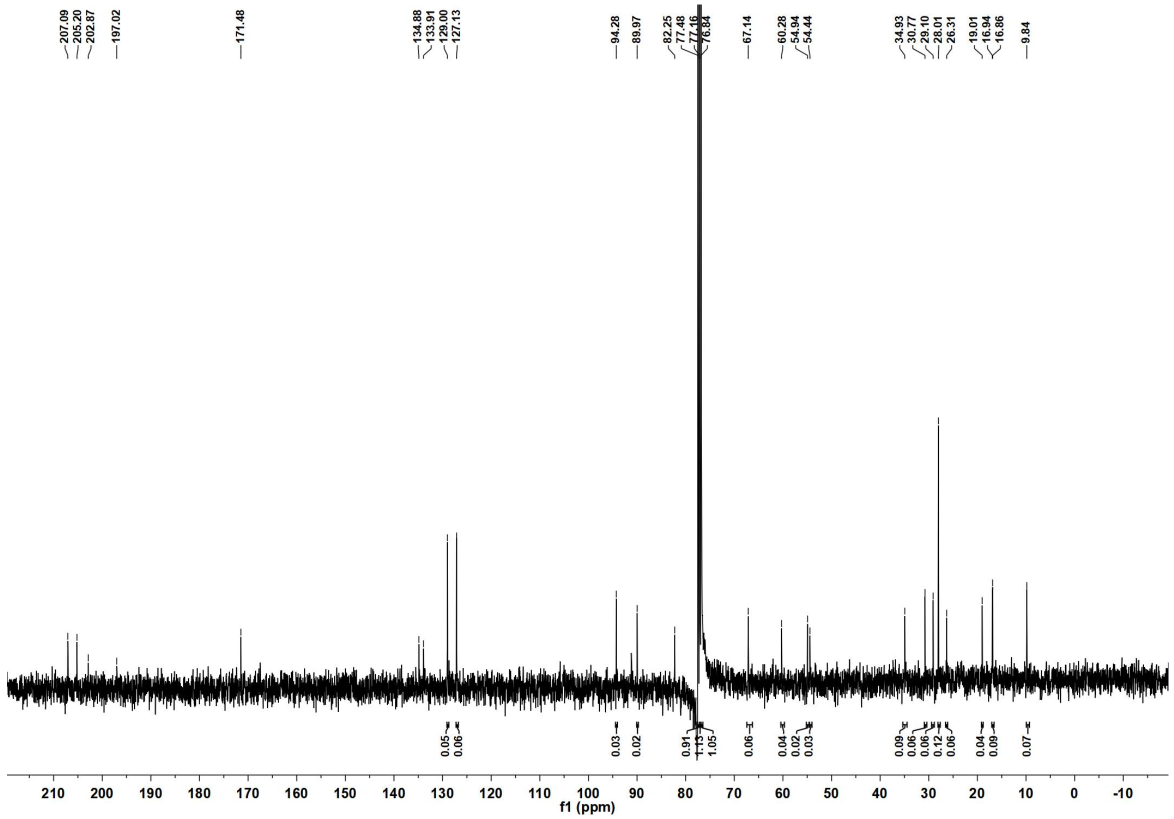

Supplementary Fig. 50. ^{13}C NMR spectra of compound **3bb**. ^{13}C NMR (101 MHz, 298K) in $CDCl_3$

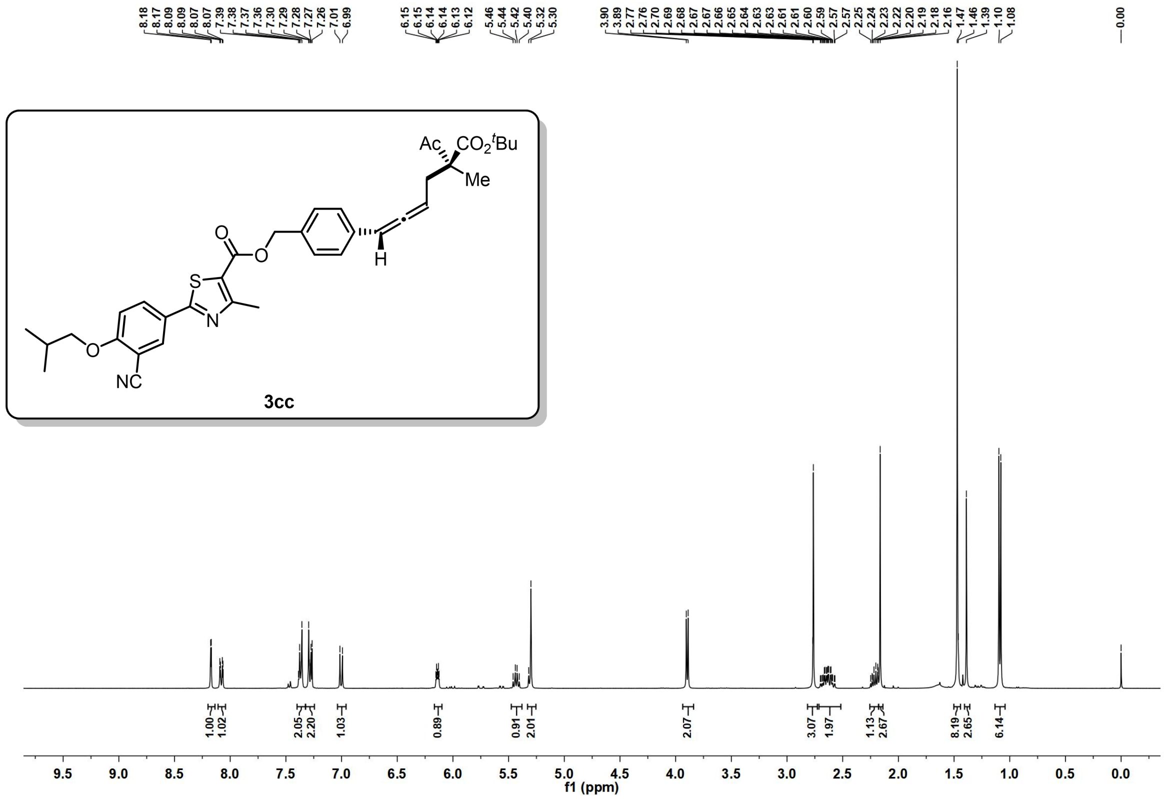

Supplementary Fig. 51. ^{1}H NMR spectra of compound **3cc**. ^{1}H NMR (400 MHz, 298K) in $CDCl_3$

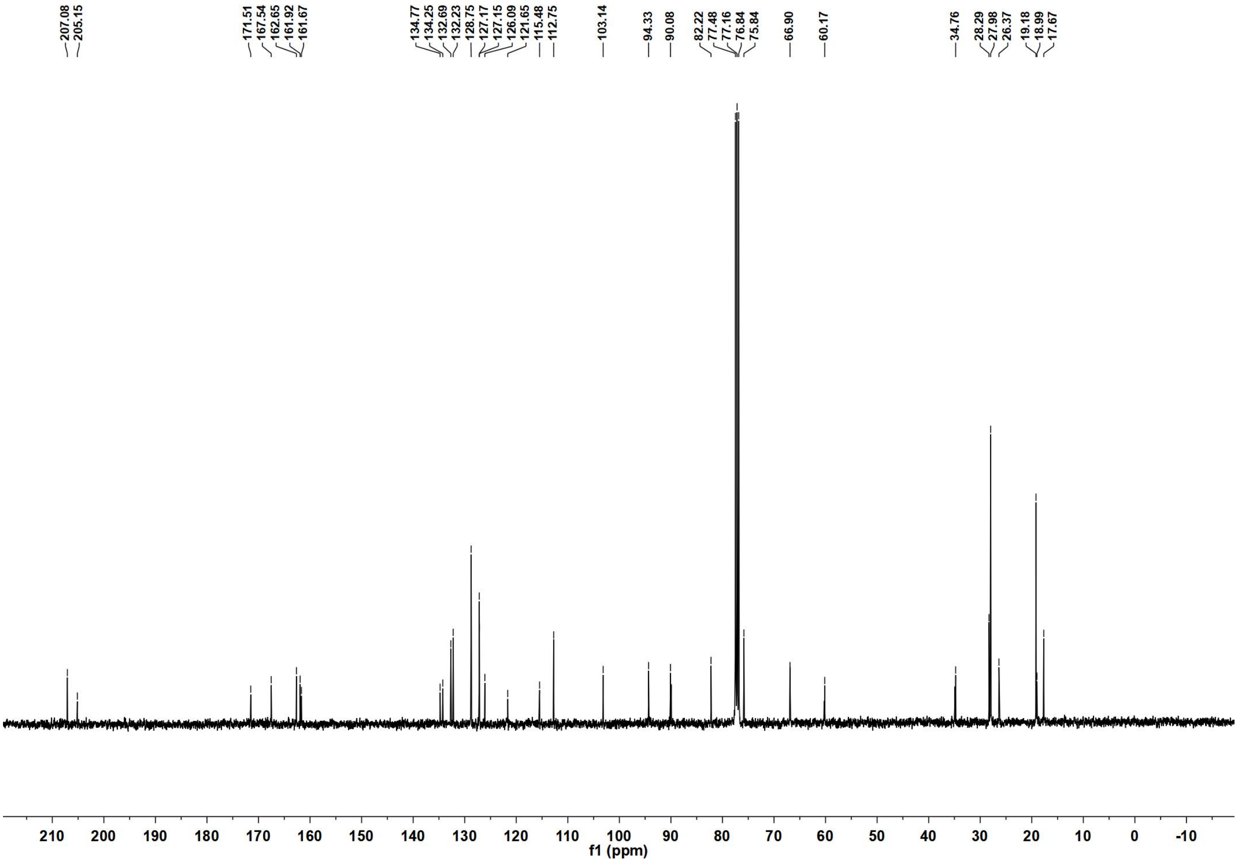

Supplementary Fig. 52. ^{13}C NMR spectra of compound **3cc**. ^{13}C NMR (101 MHz, 298K) in $CDCl_3$

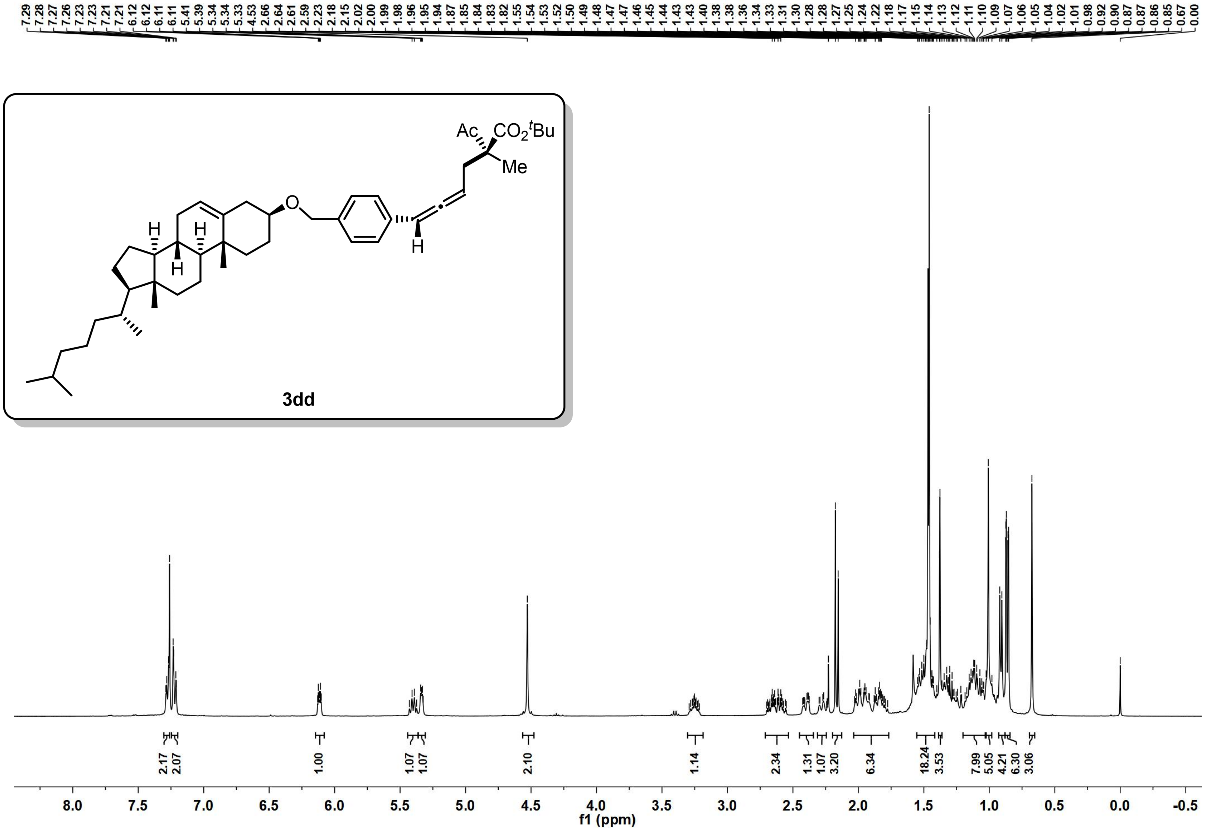

Supplementary Fig. 53. ^{1}H NMR spectra of compound **3dd**. ^{1}H NMR (400 MHz, 298K) in $CDCl_3$

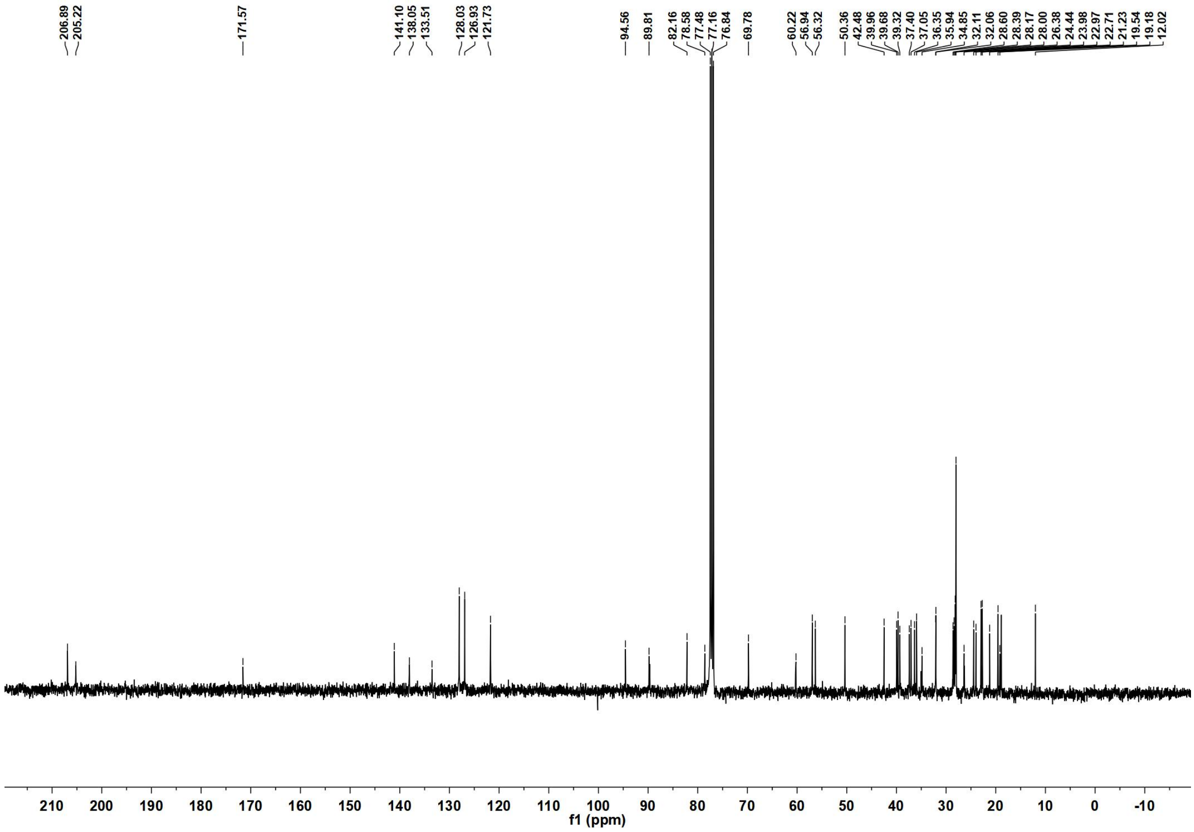

Supplementary Fig. 54. ^{13}C NMR spectra of compound **3dd**. ^{13}C NMR (101 MHz, 298K) in $CDCl_3$

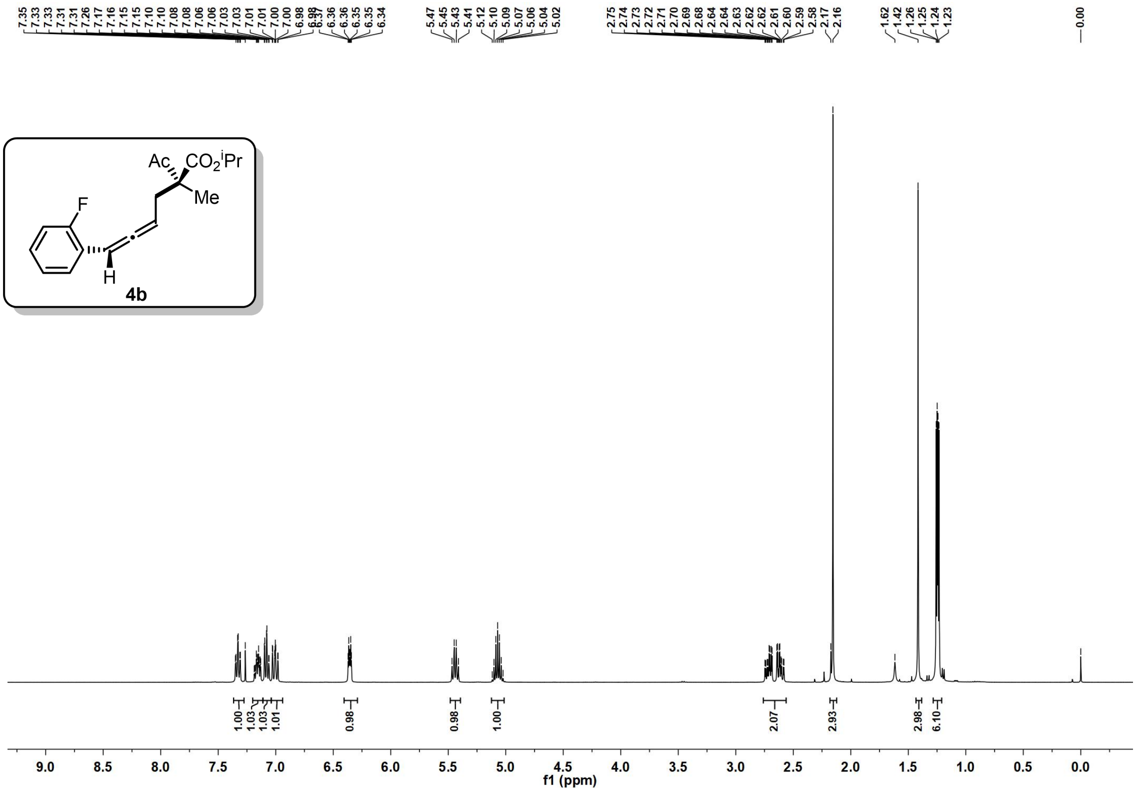

Supplementary Fig. 55. ^{1}H NMR spectra of compound **4b**. ^{1}H NMR (400 MHz, 298K) in $CDCl_3$

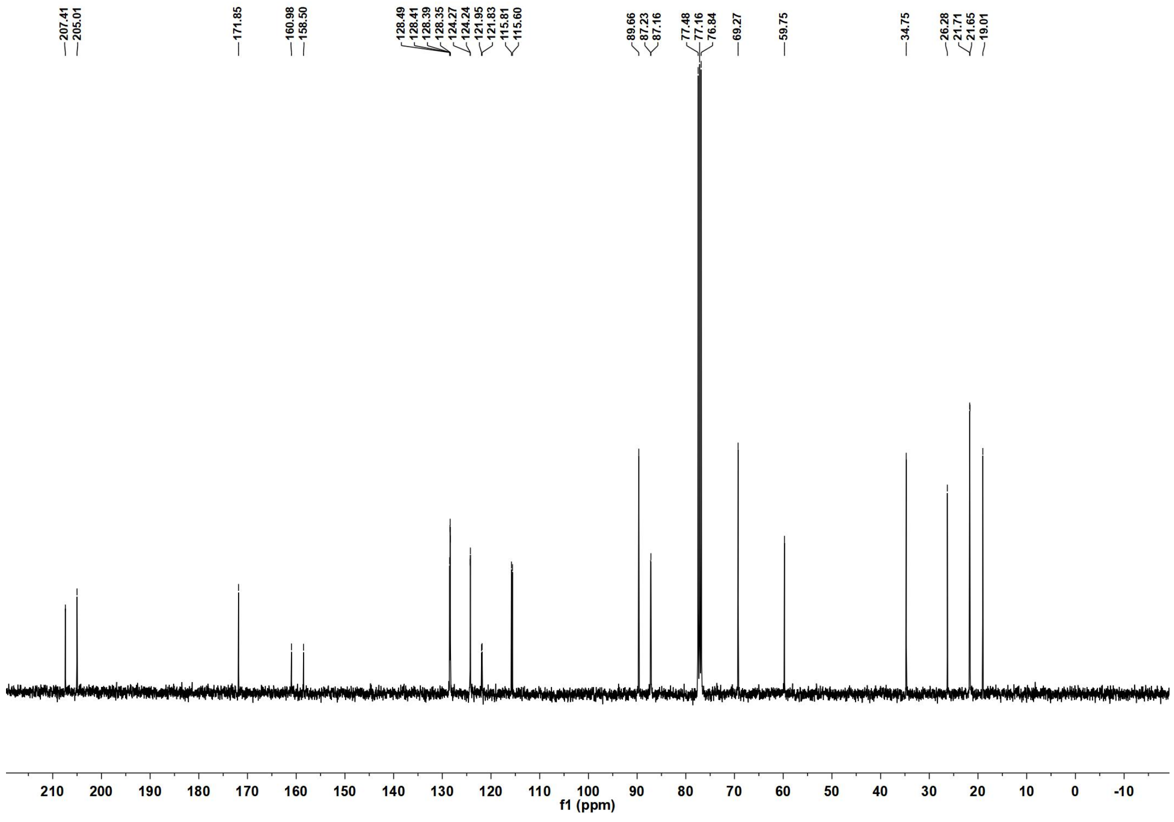

Supplementary Fig. 56. ^{13}C NMR spectra of compound **4b**. ^{13}C NMR (101 MHz, 298K) in $CDCl_3$

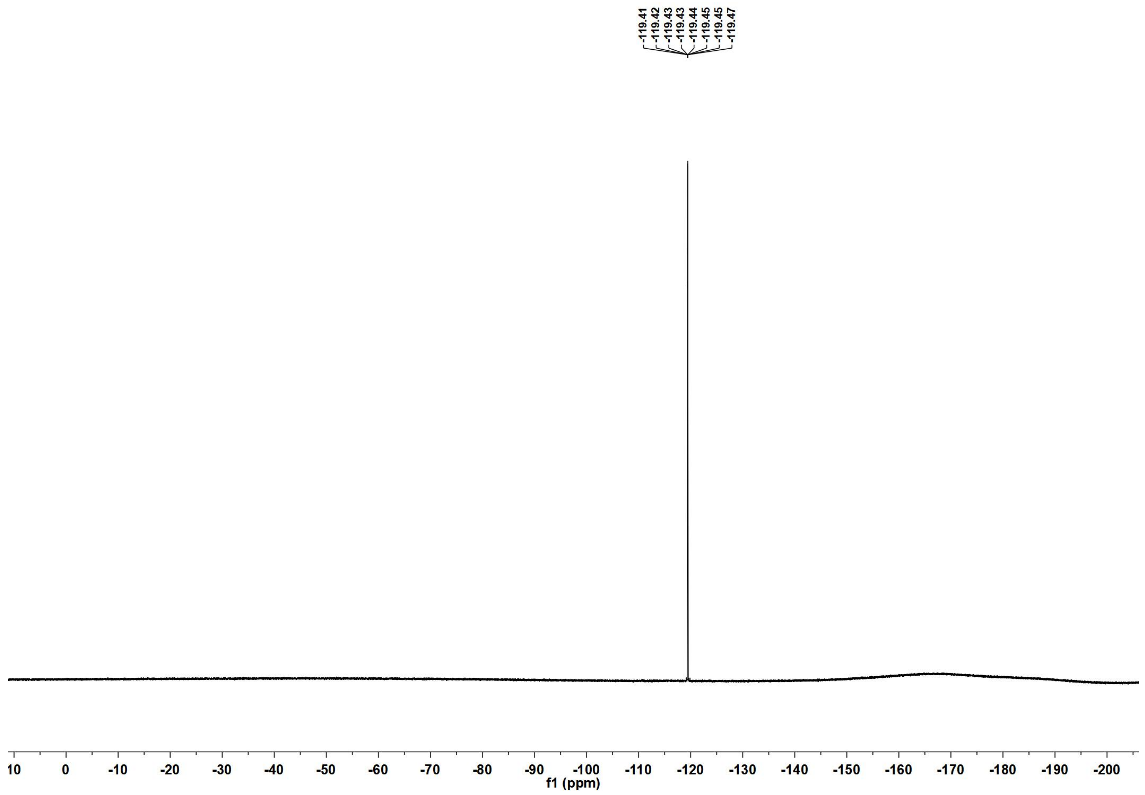

Supplementary Fig. 57. ^{19}F NMR spectra of compound **4b**. ^{19}F NMR (376 MHz, 298K) in $CDCl_3$

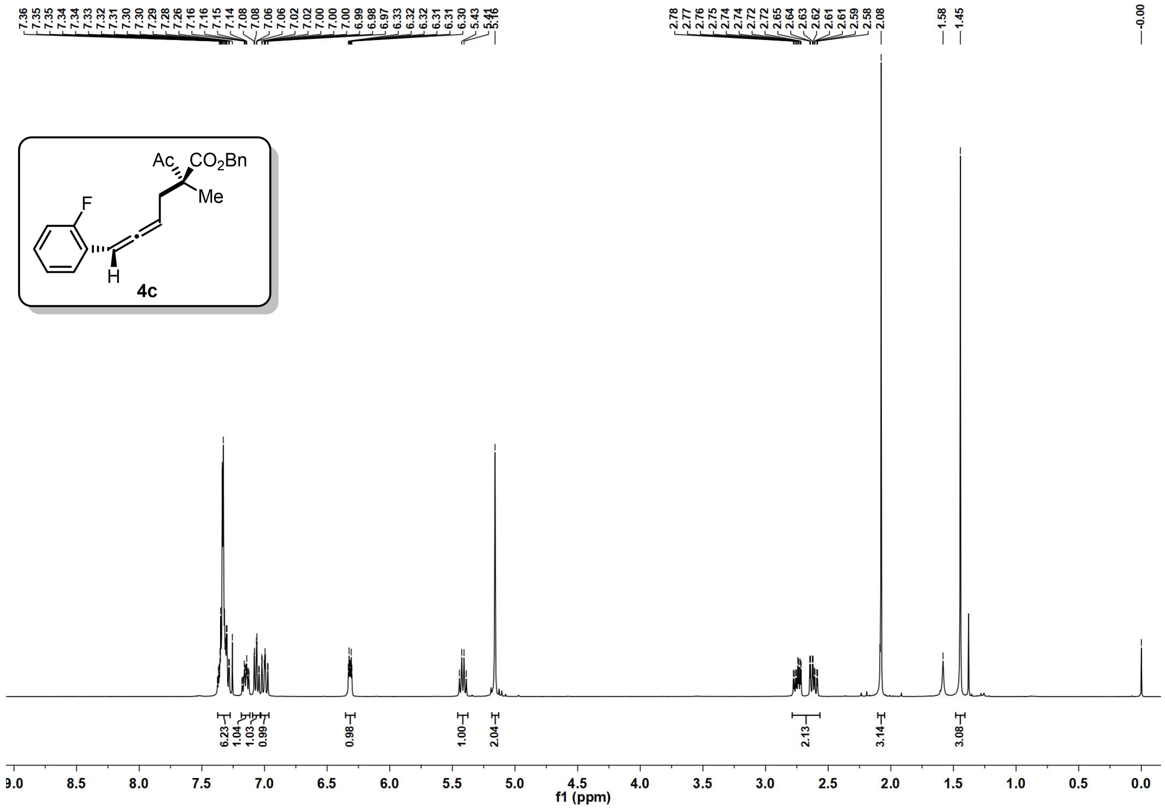

Supplementary Fig. 58. ^{1}H NMR spectra of compound **4c**. ^{1}H NMR (400 MHz, 298K) in $CDCl_3$

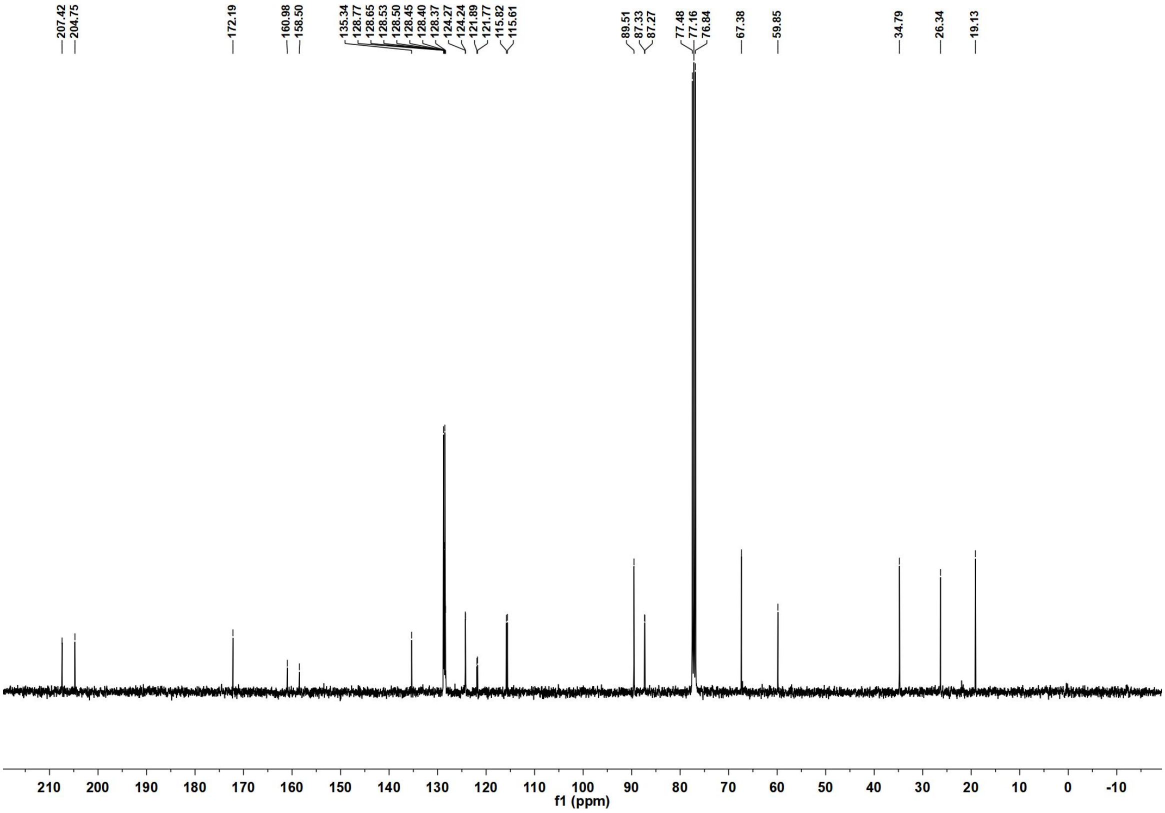

Supplementary Fig. 59. ^{13}C NMR spectra of compound **4c**. ^{13}C NMR (101 MHz, 298K) in $CDCl_3$

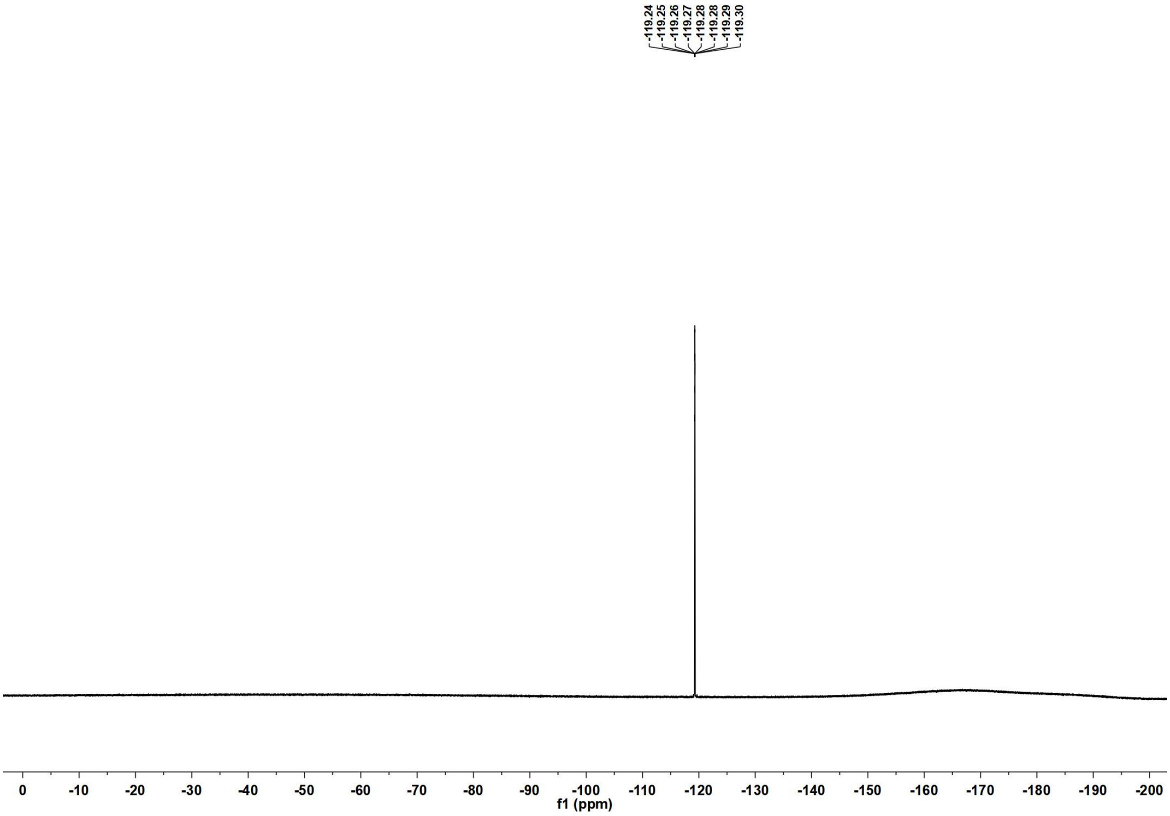

Supplementary Fig. 60. ^{19}F NMR spectra of compound **4c**. ^{19}F NMR (376 MHz, 298K) in $CDCl_3$

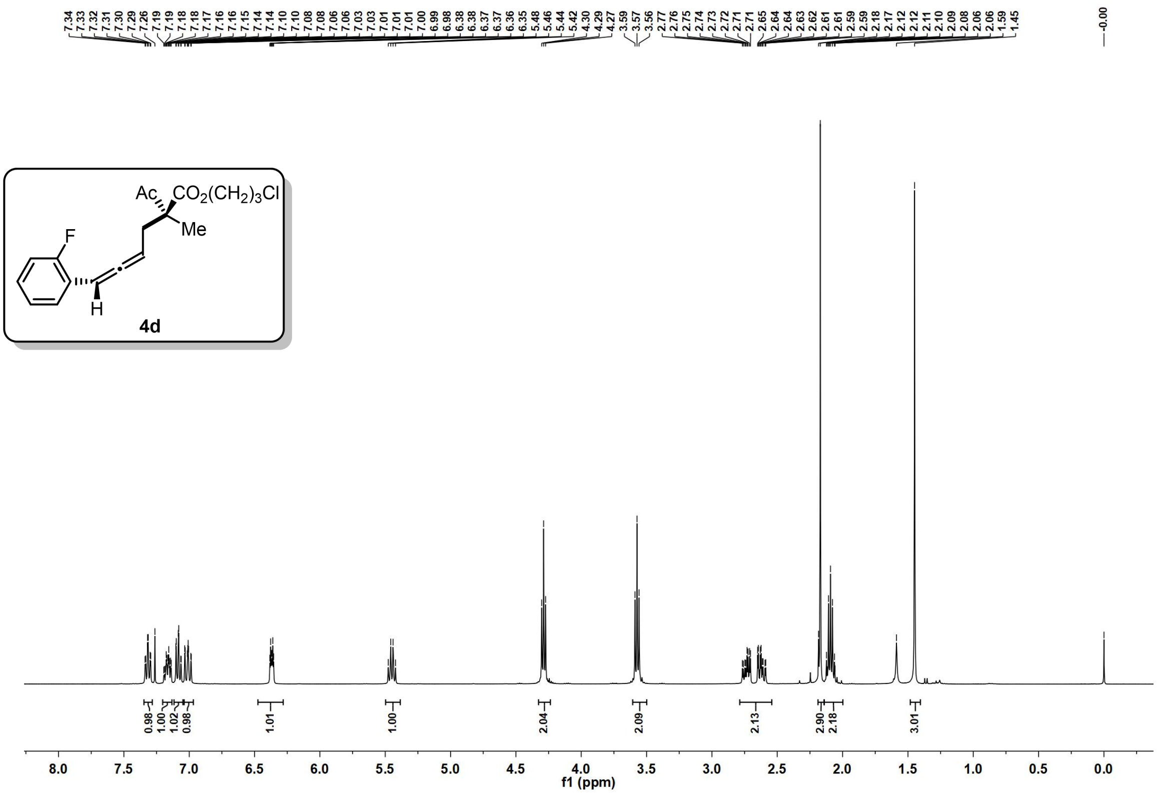

Supplementary Fig. 61. ^{1}H NMR spectra of compound **4d**. ^{1}H NMR (400 MHz, 298K) in $CDCl_3$

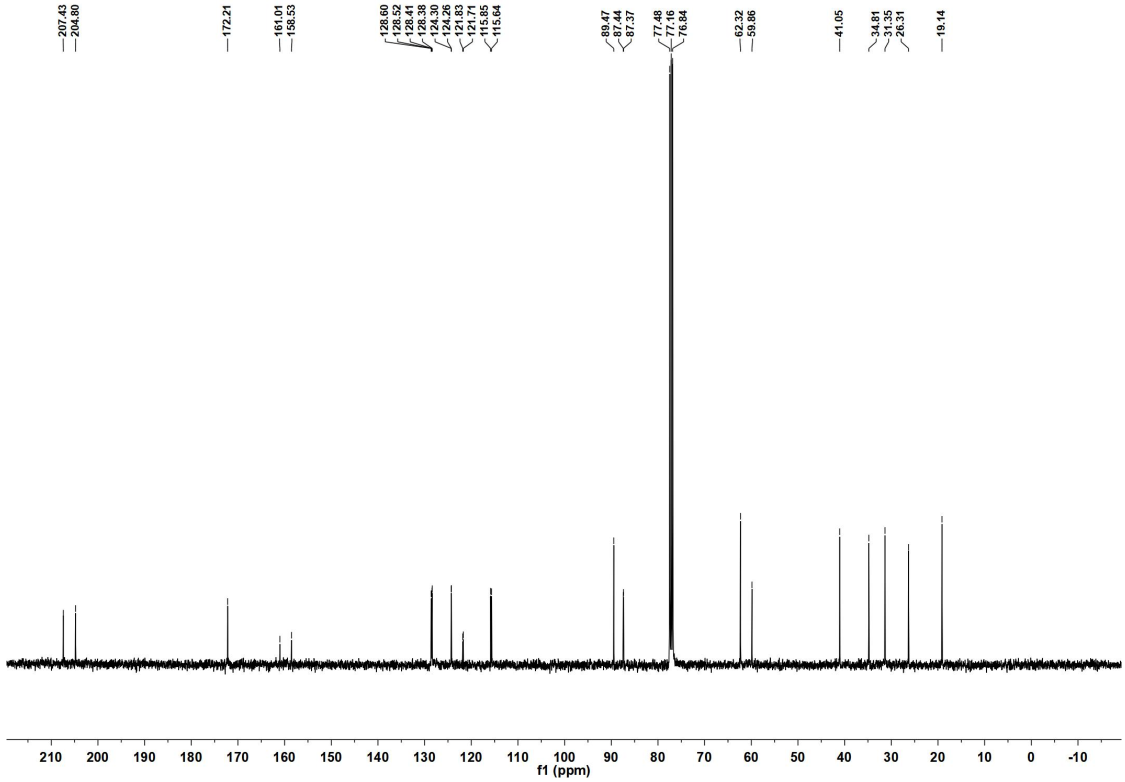

Supplementary Fig. 62. ^{13}C NMR spectra of compound **4d**. ^{13}C NMR (101 MHz, 298K) in $CDCl_3$

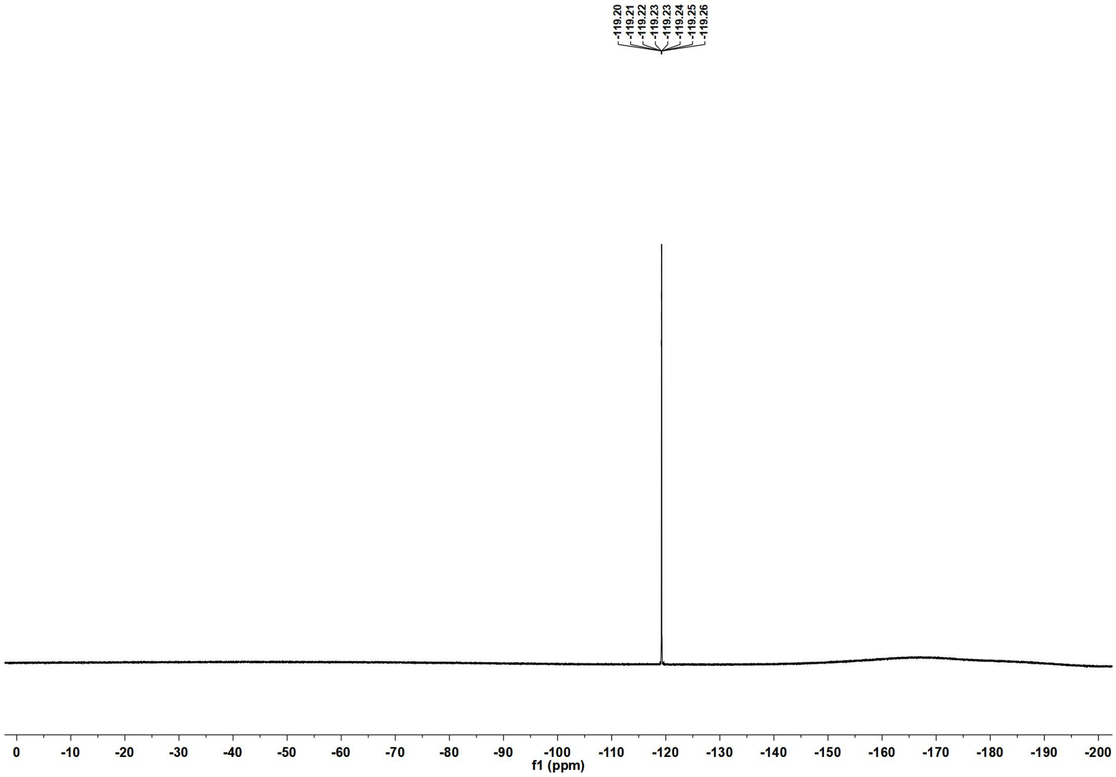

Supplementary Fig. 63. ^{19}F NMR spectra of compound **4d**. ^{19}F NMR (376 MHz, 298K) in $CDCl_3$

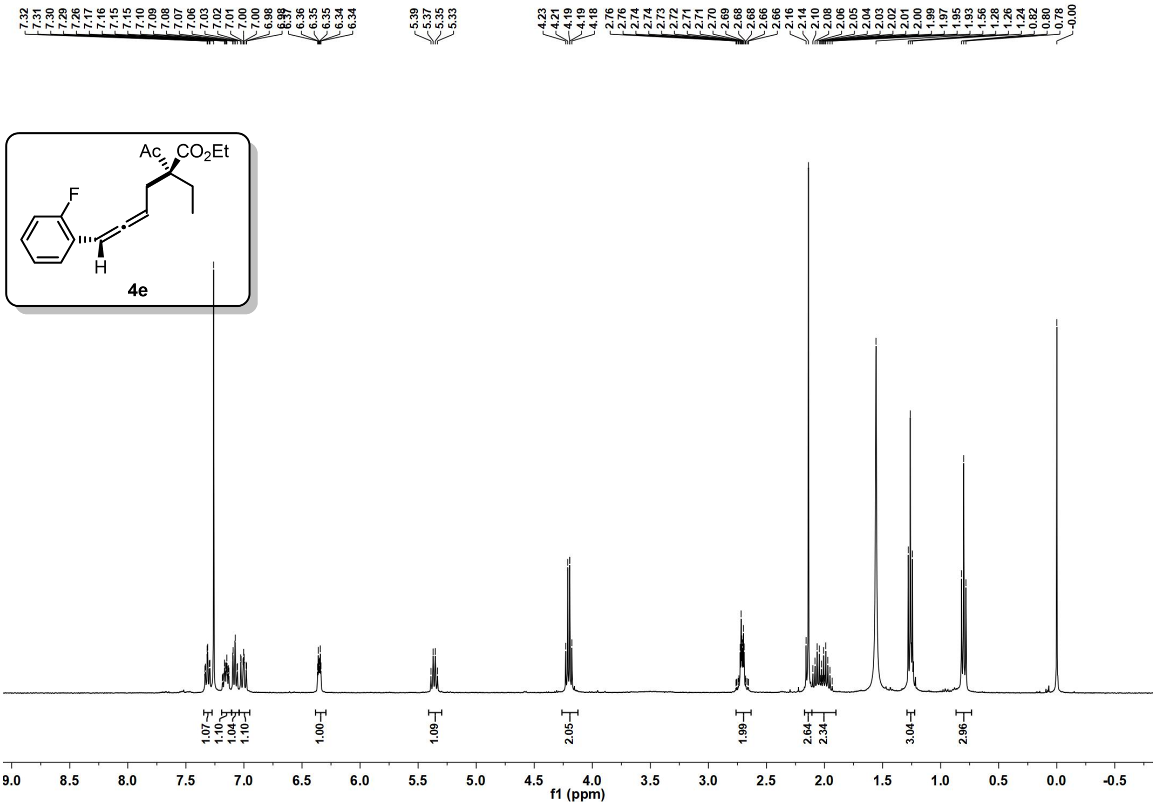

Supplementary Fig. 64. ^{1}H NMR spectra of compound **4e**. ^{1}H NMR (400 MHz, 298K) in $CDCl_3$

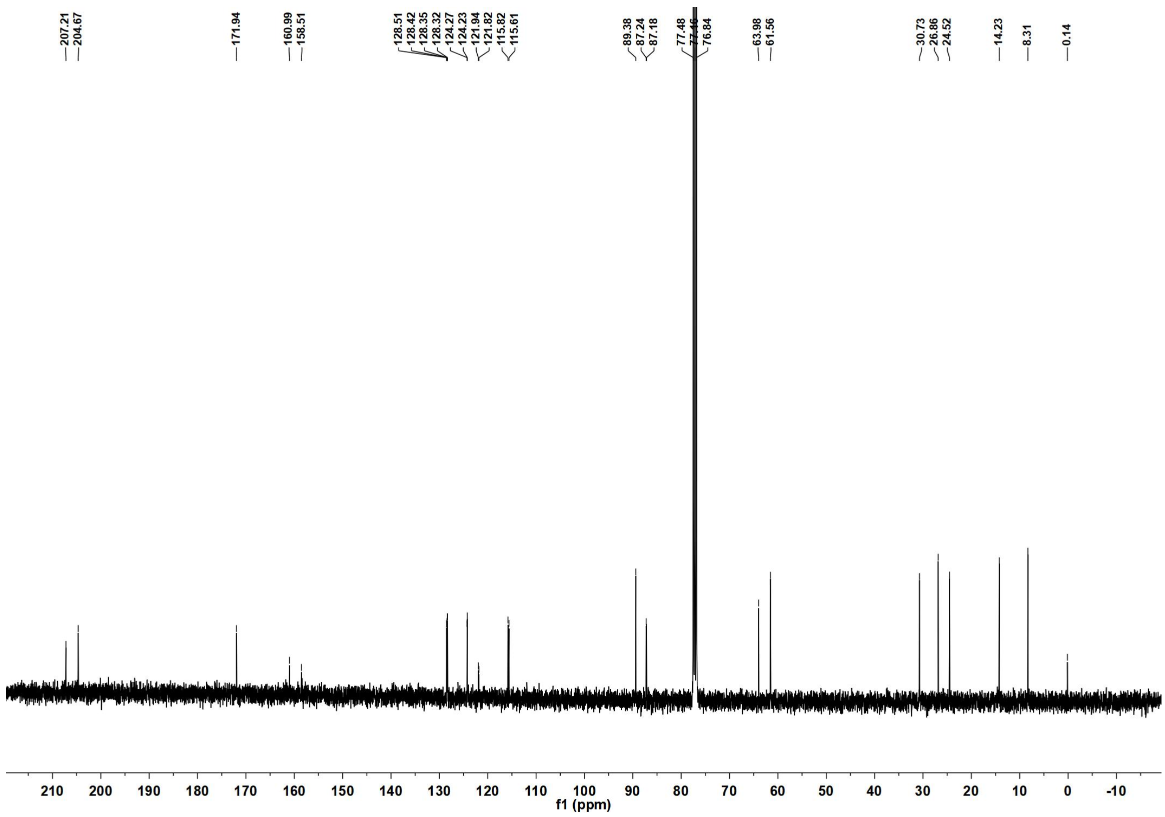

Supplementary Fig. 65. ^{13}C NMR spectra of compound **4e**. ^{13}C NMR (101 MHz, 298K) in $CDCl_3$

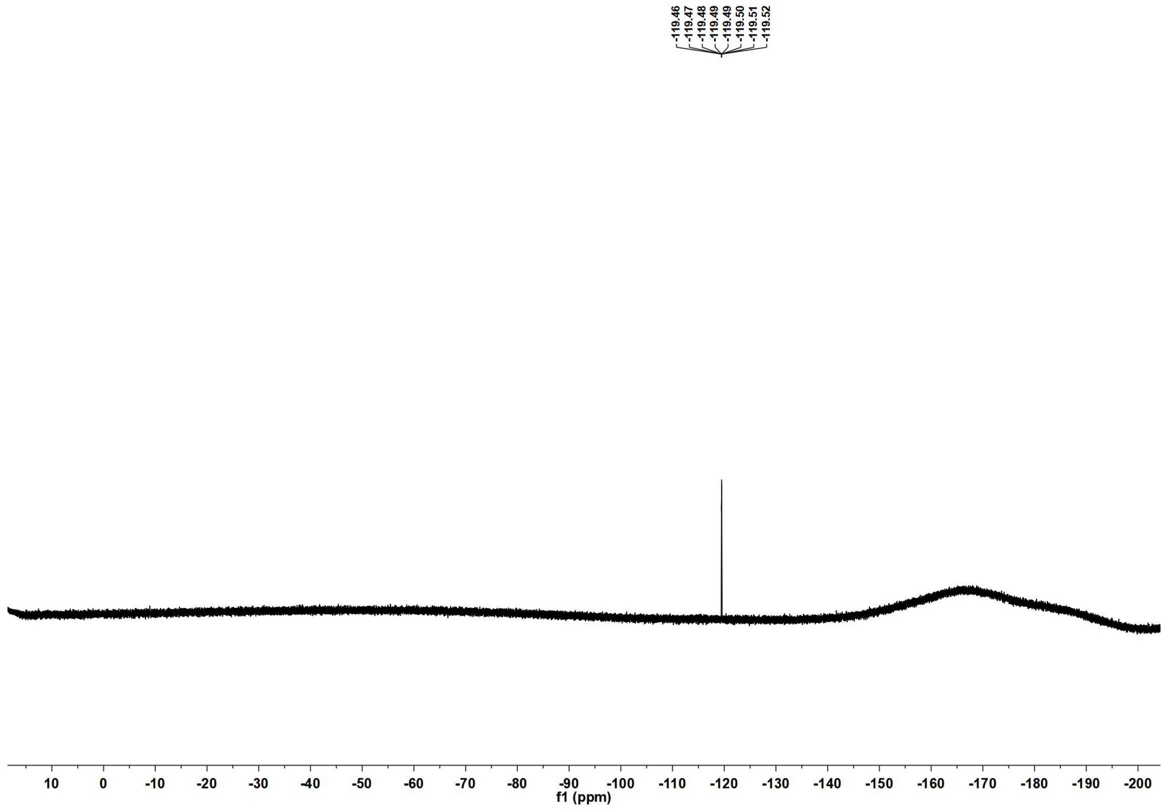

Supplementary Fig. 66. ^{19}F NMR spectra of compound **4e**. ^{19}F NMR (376 MHz, 298K) in $CDCl_3$

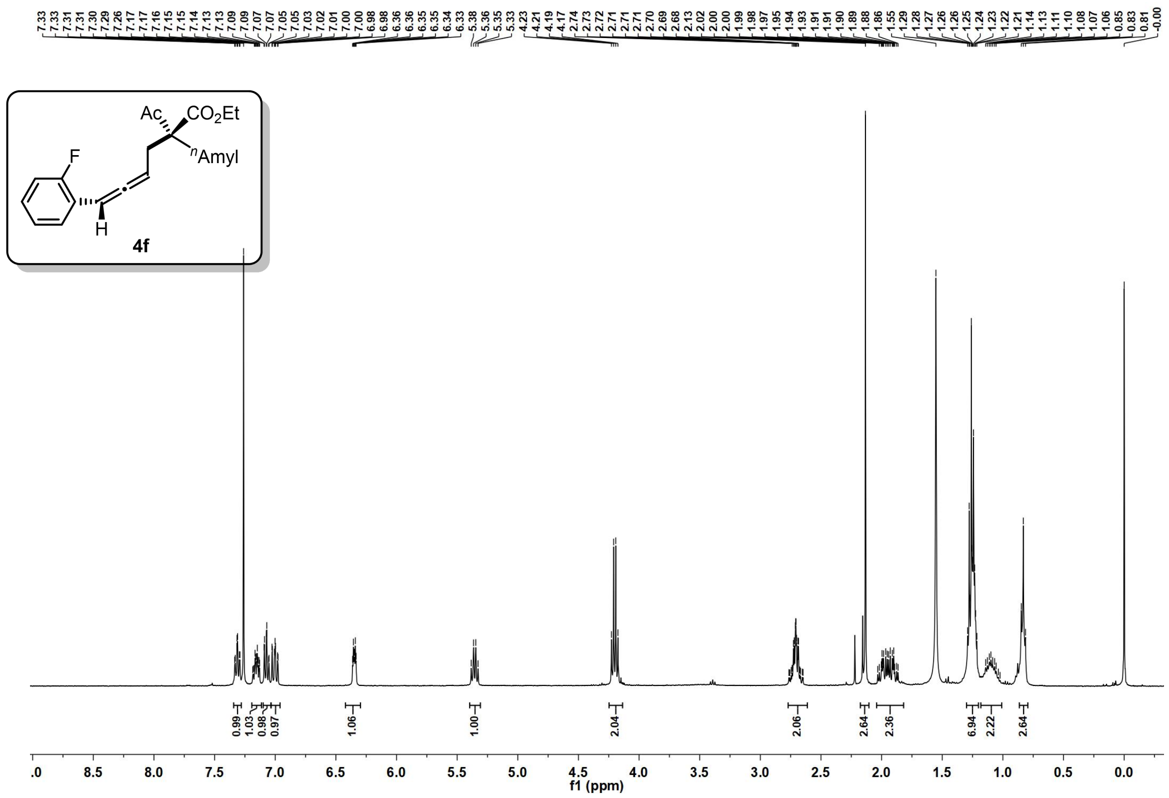

Supplementary Fig. 67. ^{1}H NMR spectra of compound **4f**. ^{1}H NMR (400 MHz, 298K) in $CDCl_3$

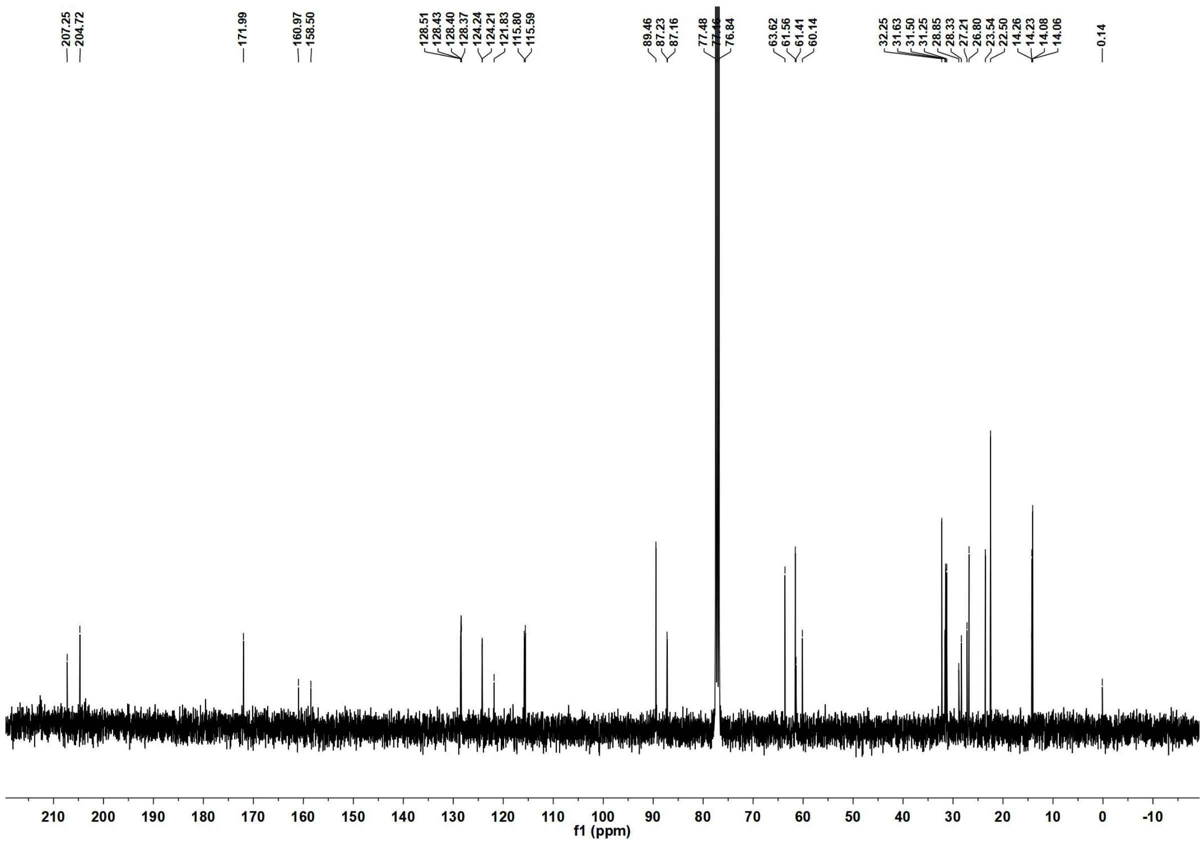

Supplementary Fig. 68. ^{13}C NMR spectra of compound **4f**. ^{13}C NMR (101 MHz, 298K) in $CDCl_3$

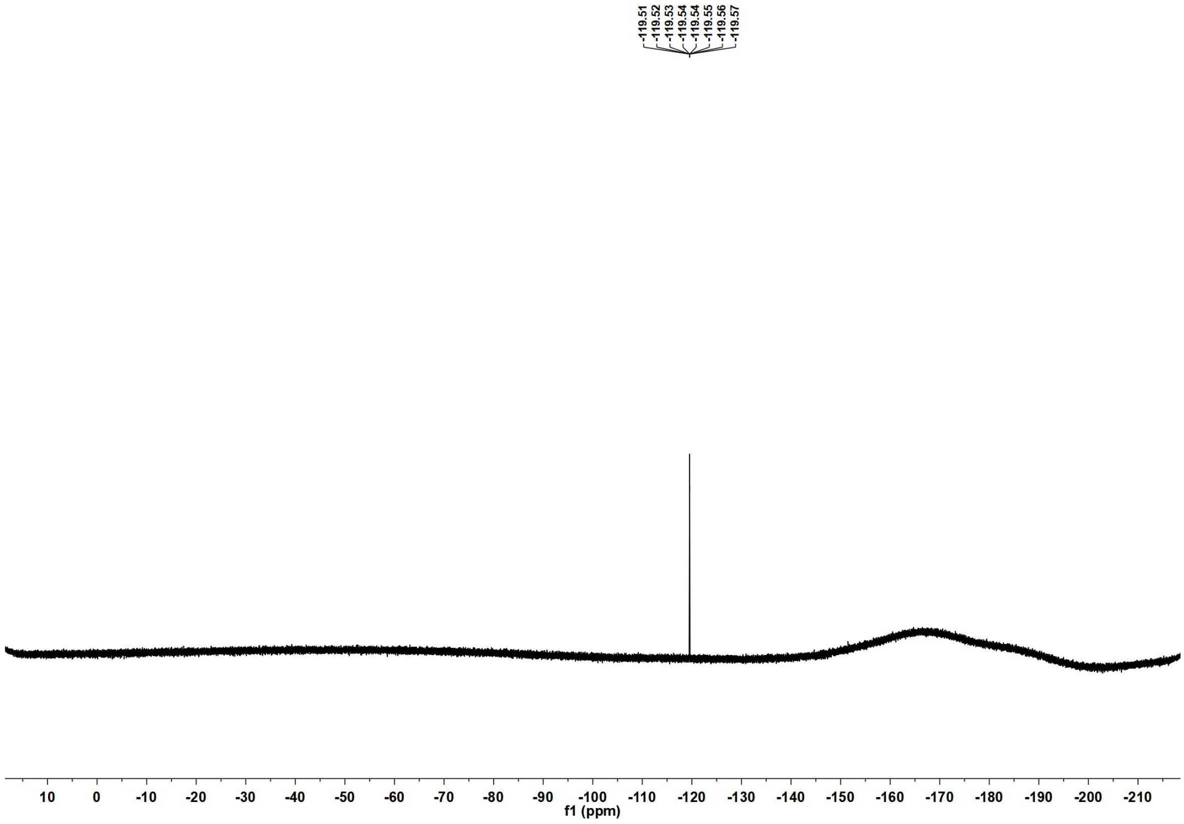

Supplementary Fig. 69. ^{19}F NMR spectra of compound **4f**. ^{19}F NMR (376 MHz, 298K) in $CDCl_3$

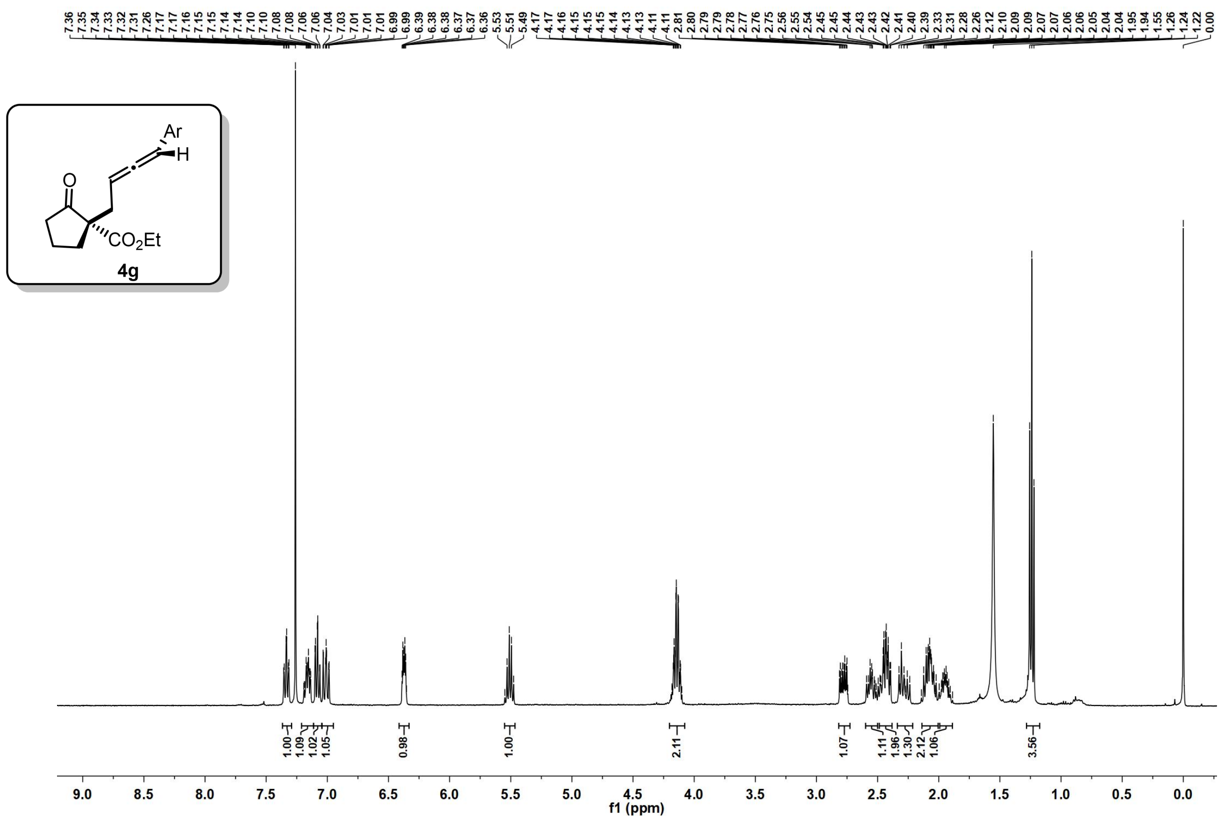

Supplementary Fig. 70. ^{1}H NMR spectra of compound **4g**. ^{1}H NMR (400 MHz, 298K) in $CDCl_3$

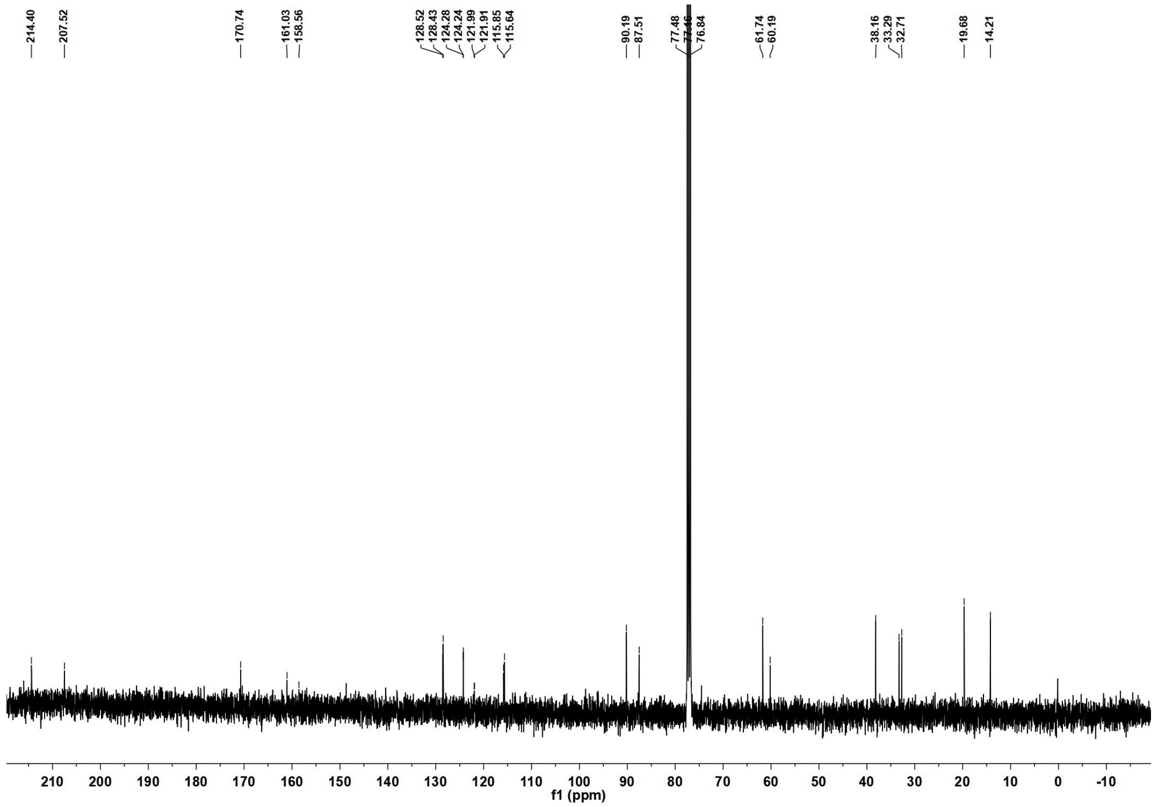

Supplementary Fig. 71. ^{13}C NMR spectra of compound **4g**. ^{13}C NMR (101 MHz, 298K) in $CDCl_3$

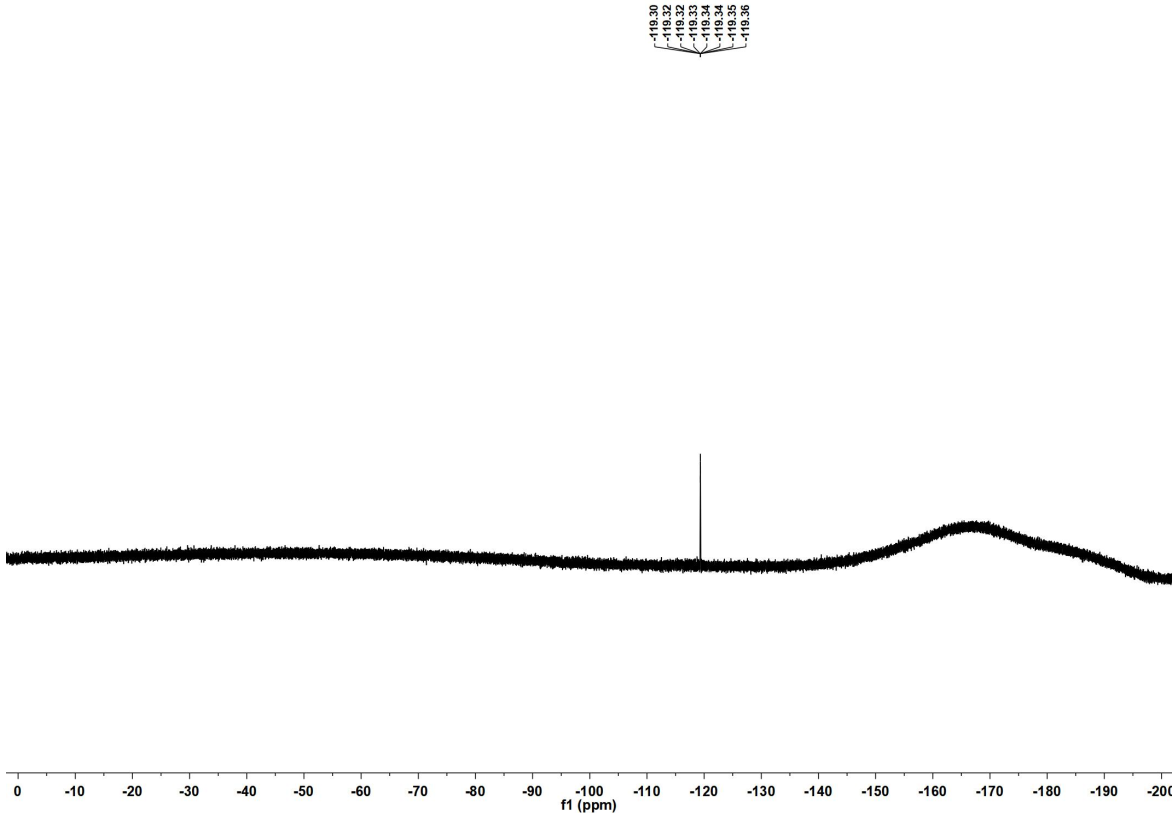

Supplementary Fig. 72. ^{19}F NMR spectra of compound **4g**. ^{19}F NMR (376 MHz, 298K) in $CDCl_3$

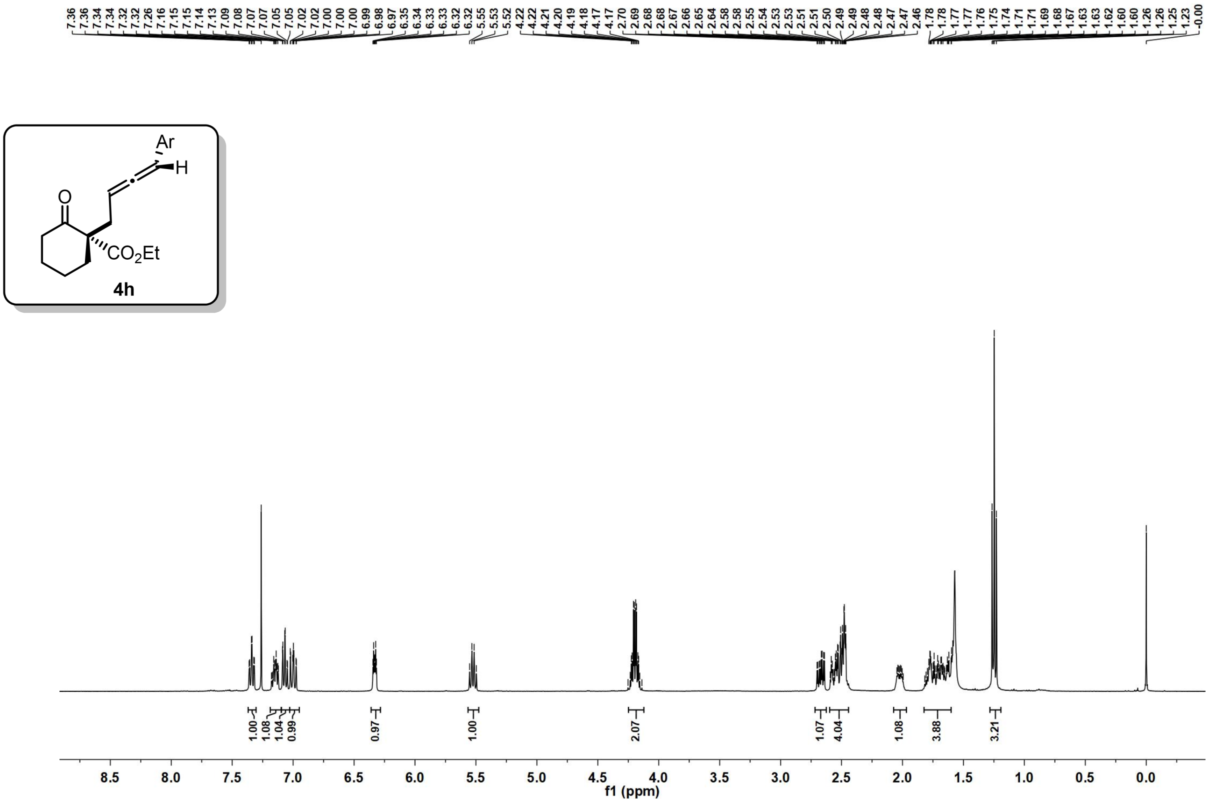

Supplementary Fig. 73. ^{1}H NMR spectra of compound **4h**. ^{1}H NMR (400 MHz, 298K) in $CDCl_3$

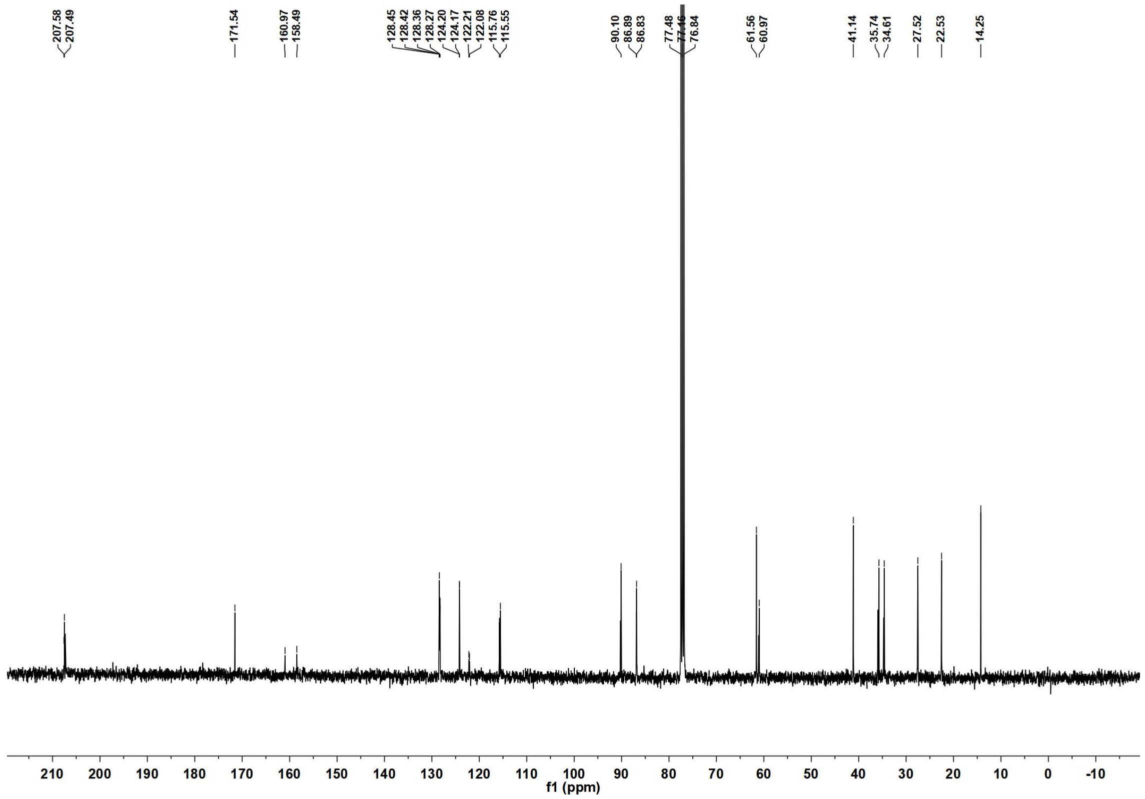

Supplementary Fig. 74. ^{13}C NMR spectra of compound **4h**. ^{13}C NMR (101 MHz, 298K) in $CDCl_3$

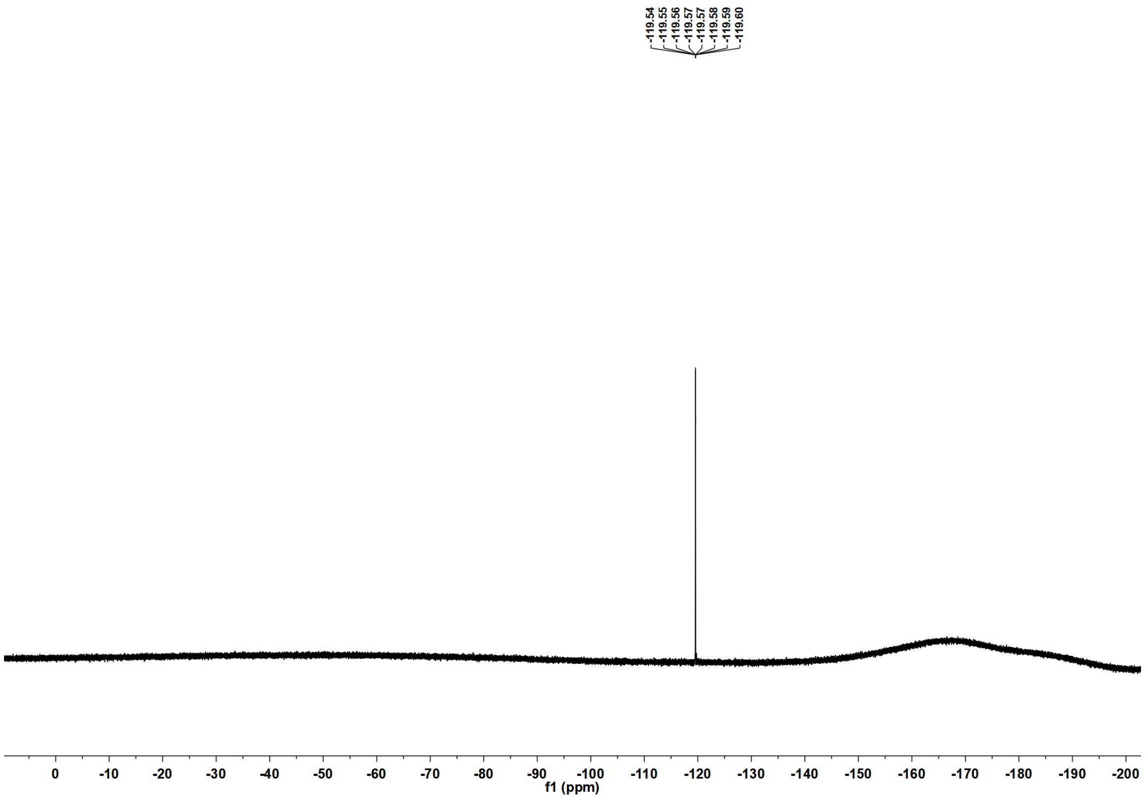

Supplementary Fig. 75. ^{19}F NMR spectra of compound **4h**. ^{19}F NMR (376 MHz, 298K) in $CDCl_3$

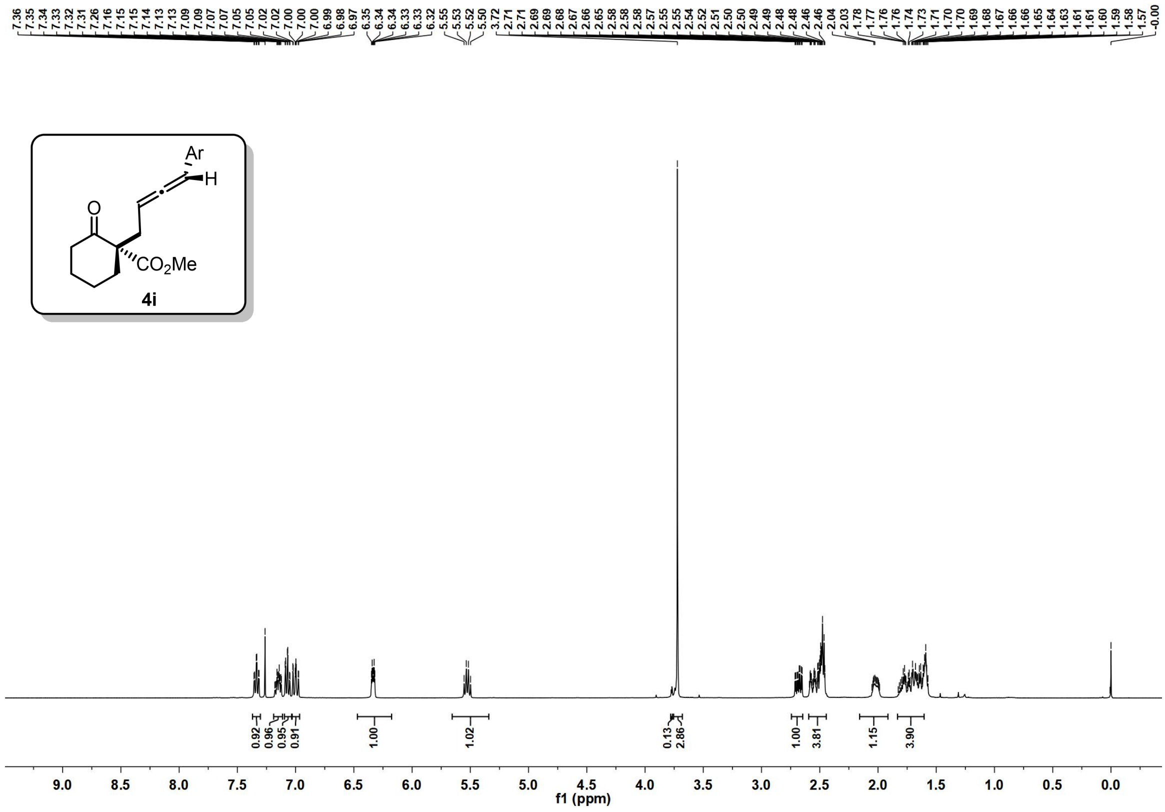

Supplementary Fig. 76. ^{1}H NMR spectra of compound **4i**. ^{1}H NMR (400 MHz, 298K) in $CDCl_3$

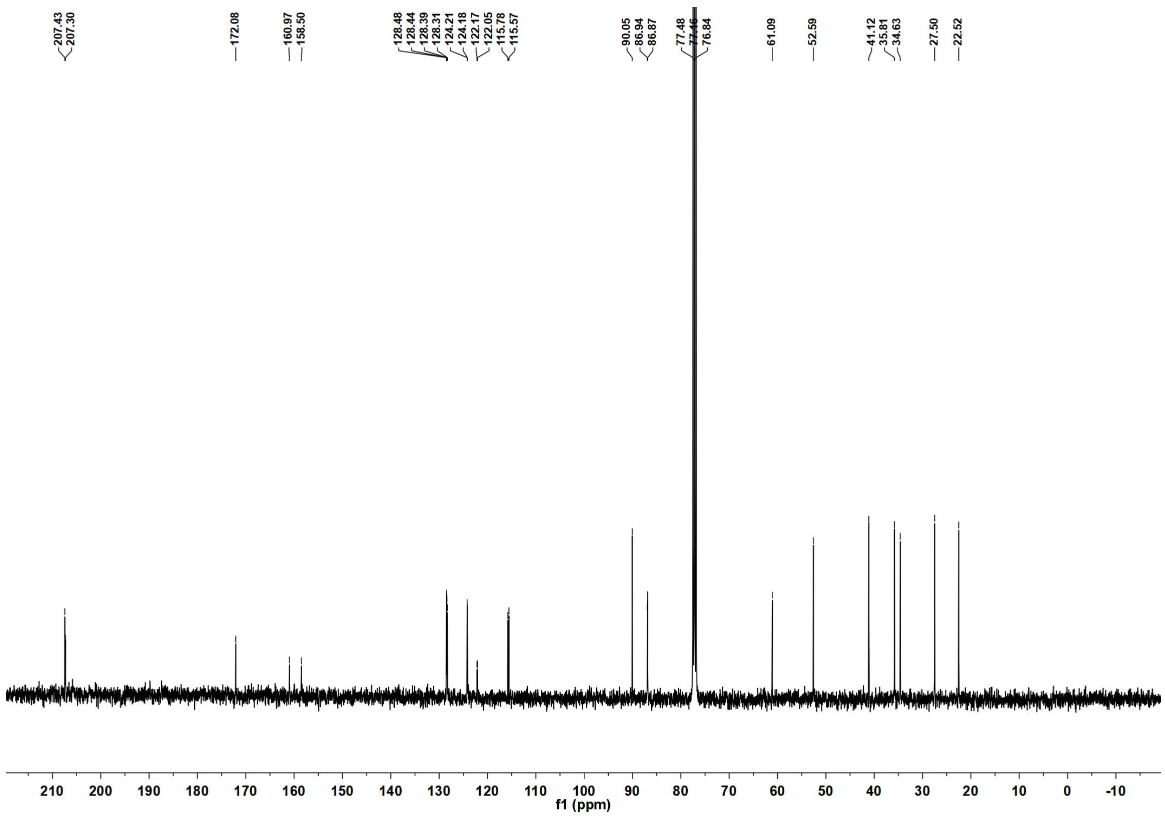

Supplementary Fig. 77. ^{13}C NMR spectra of compound **4i**. ^{13}C NMR (101 MHz, 298K) in $CDCl_3$

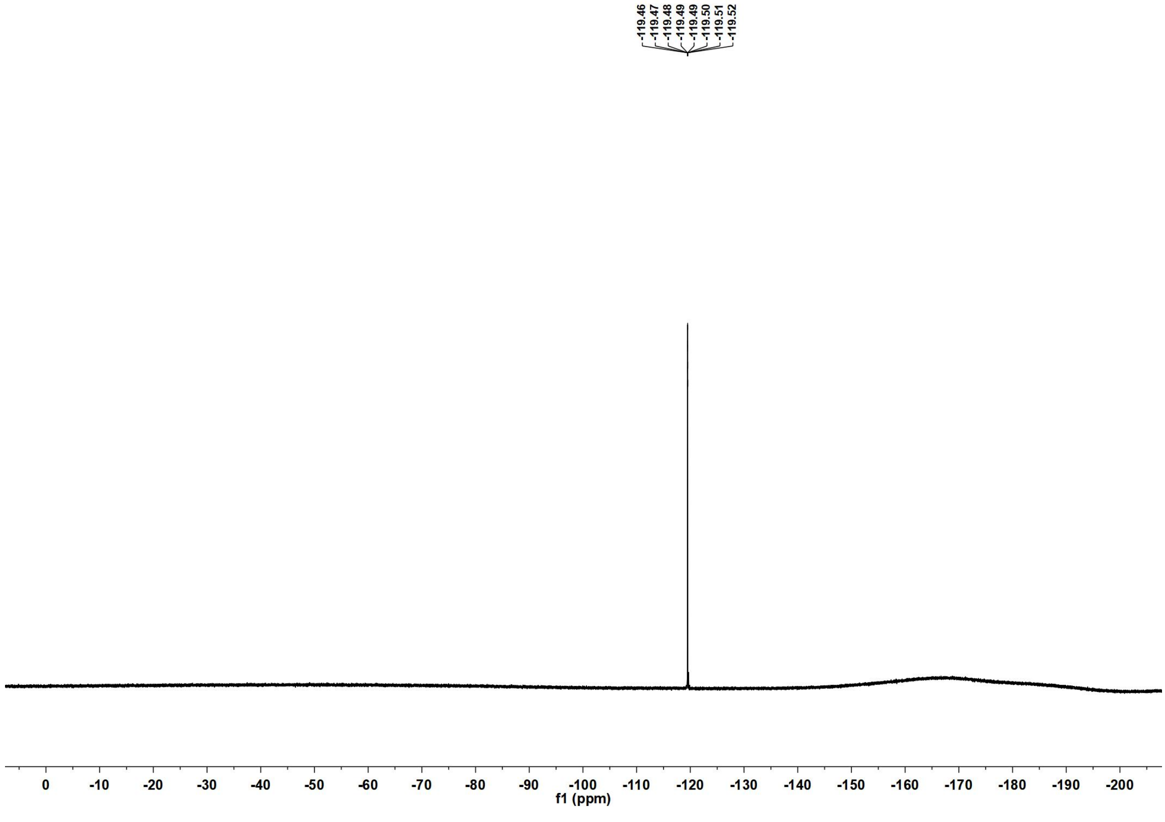

Supplementary Fig. 78. ^{19}F NMR spectra of compound **4i**. ^{19}F NMR (376 MHz, 298K) in $CDCl_3$

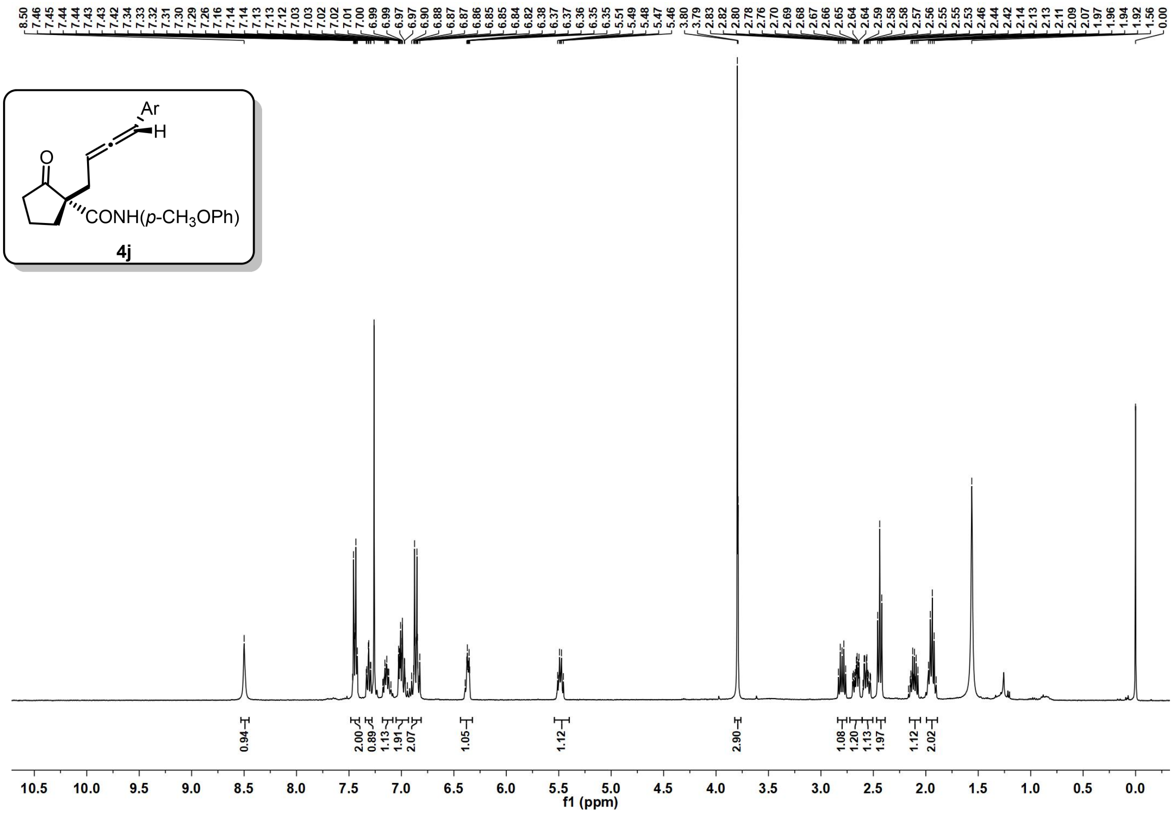

Supplementary Fig. 79. ^{1}H NMR spectra of compound **4j**. ^{1}H NMR (400 MHz, 298K) in $CDCl_3$

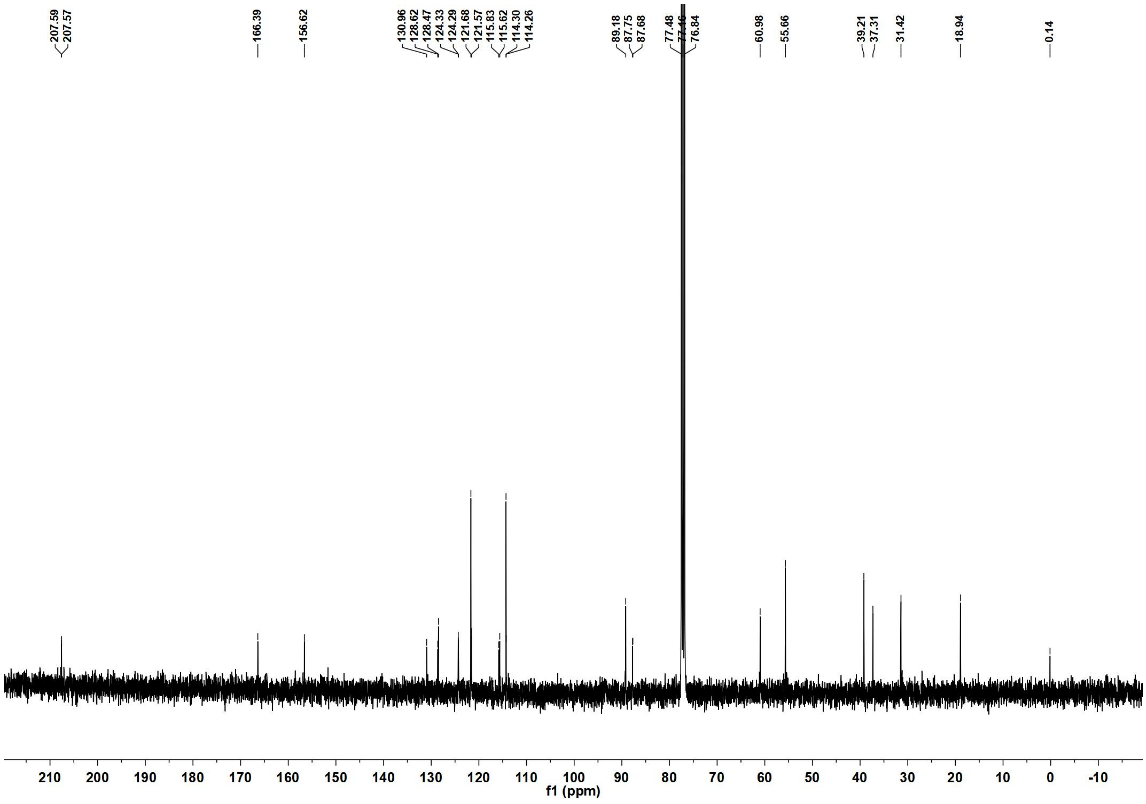

Supplementary Fig. 80. ^{13}C NMR spectra of compound **4j**. ^{13}C NMR (101 MHz, 298K) in $CDCl_3$

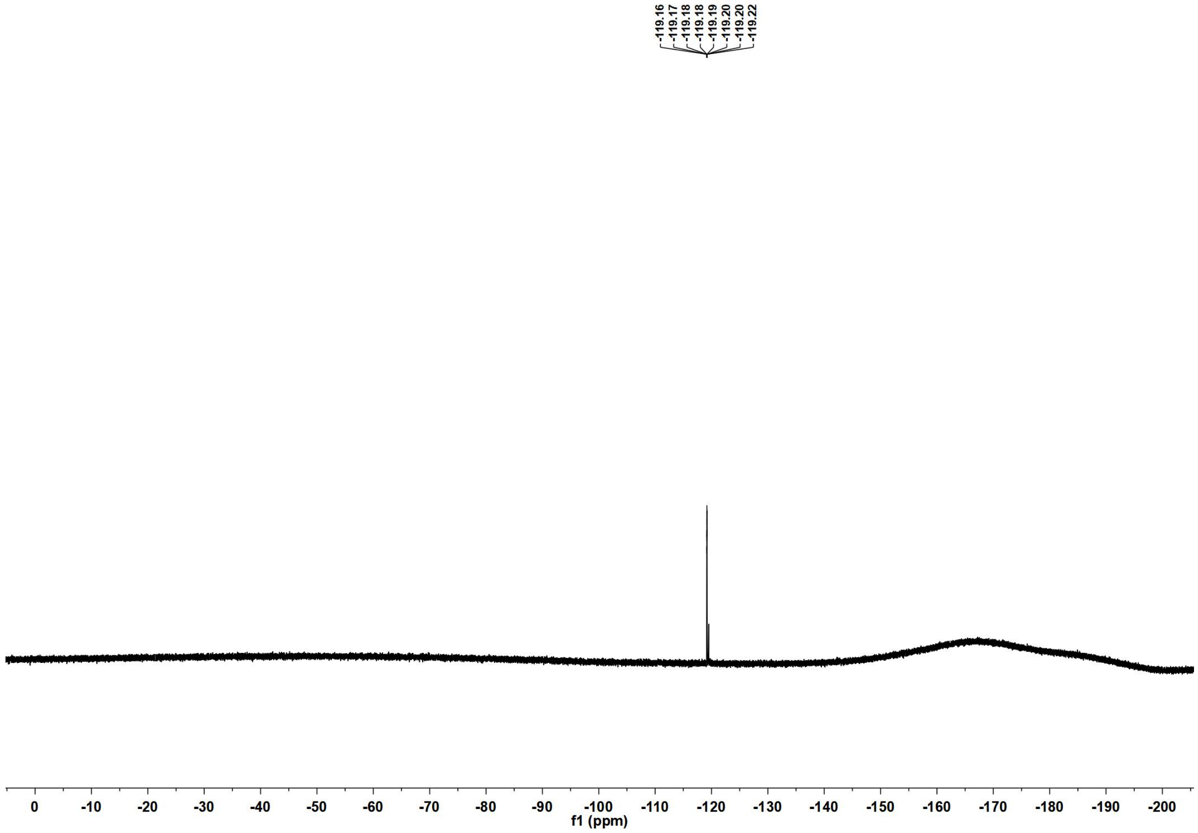

Supplementary Fig. 81. ^{19}F NMR spectra of compound **4j**. ^{19}F NMR (376 MHz, 298K) in $CDCl_3$

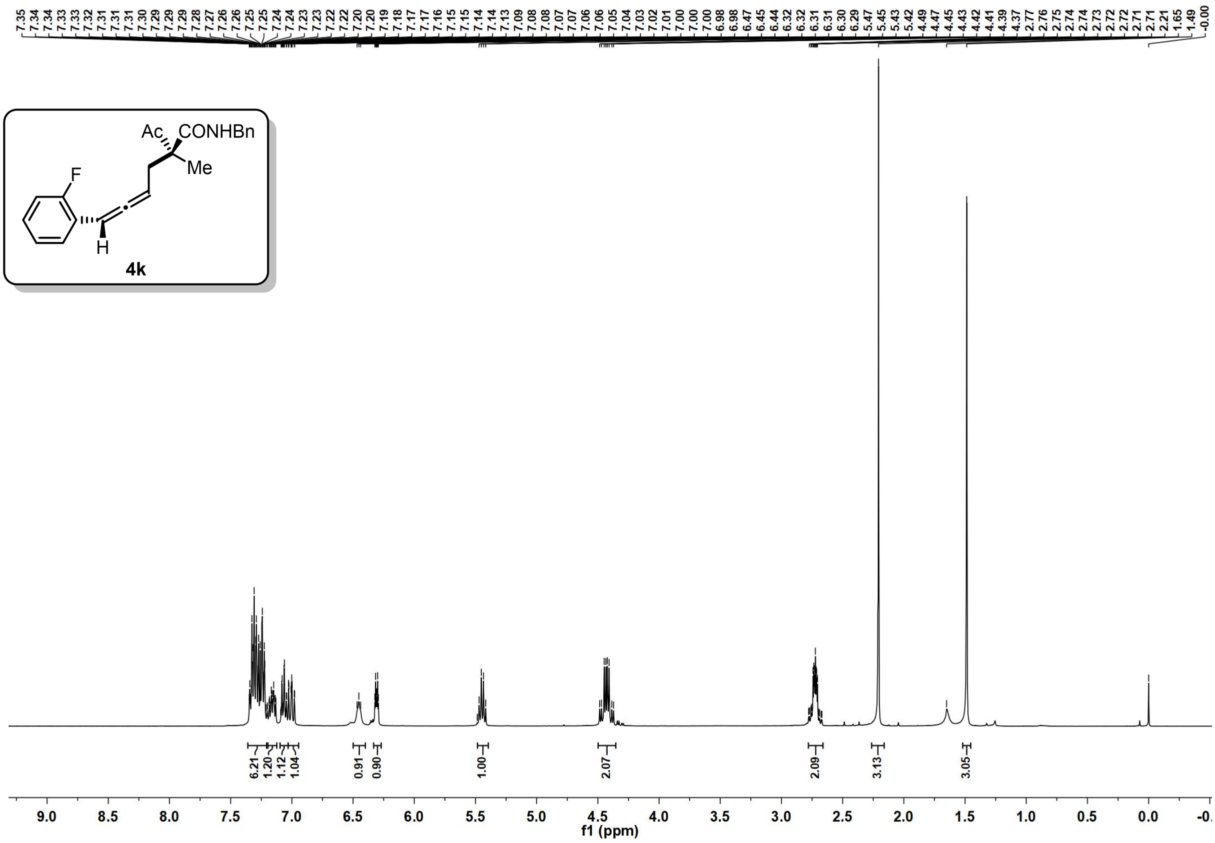

Supplementary Fig. 82. ^{1}H NMR spectra of compound **4k**. ^{1}H NMR (400 MHz, 298K) in $CDCl_3$

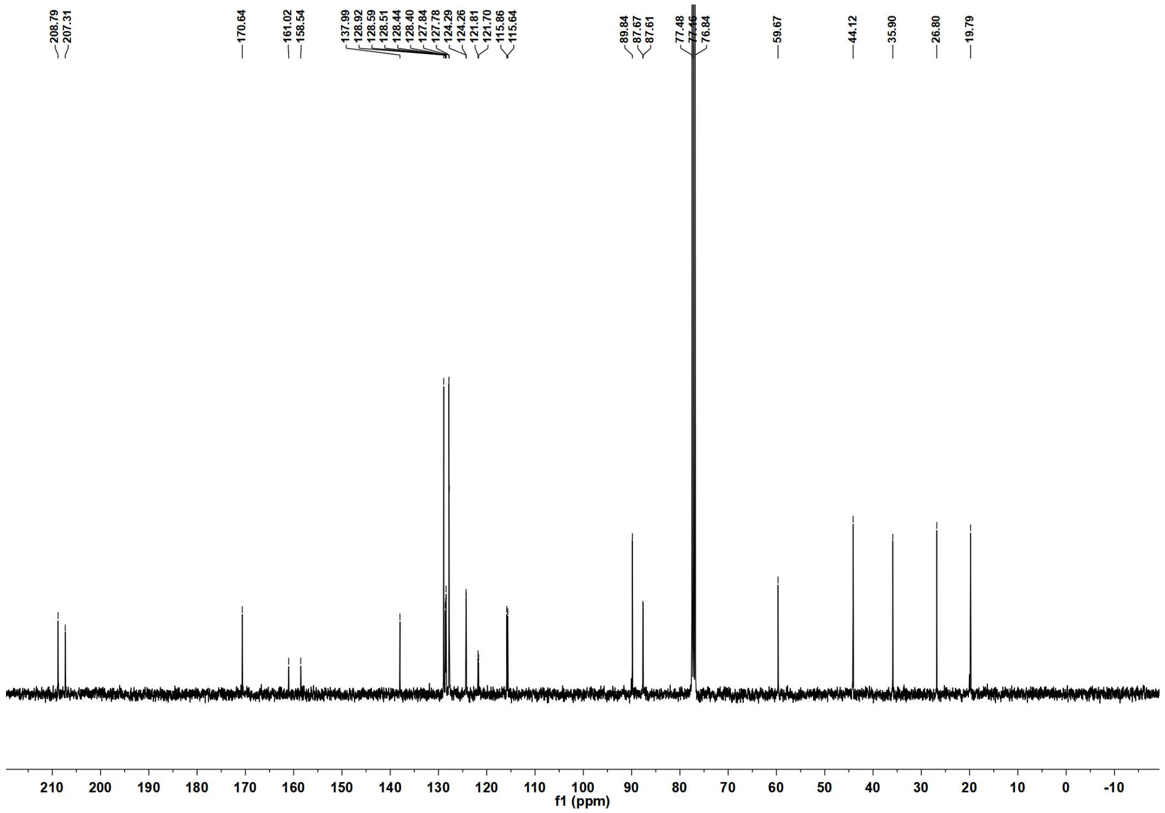

Supplementary Fig. 83. ^{13}C NMR spectra of compound **4k**. ^{13}C NMR (101 MHz, 298K) in $CDCl_3$

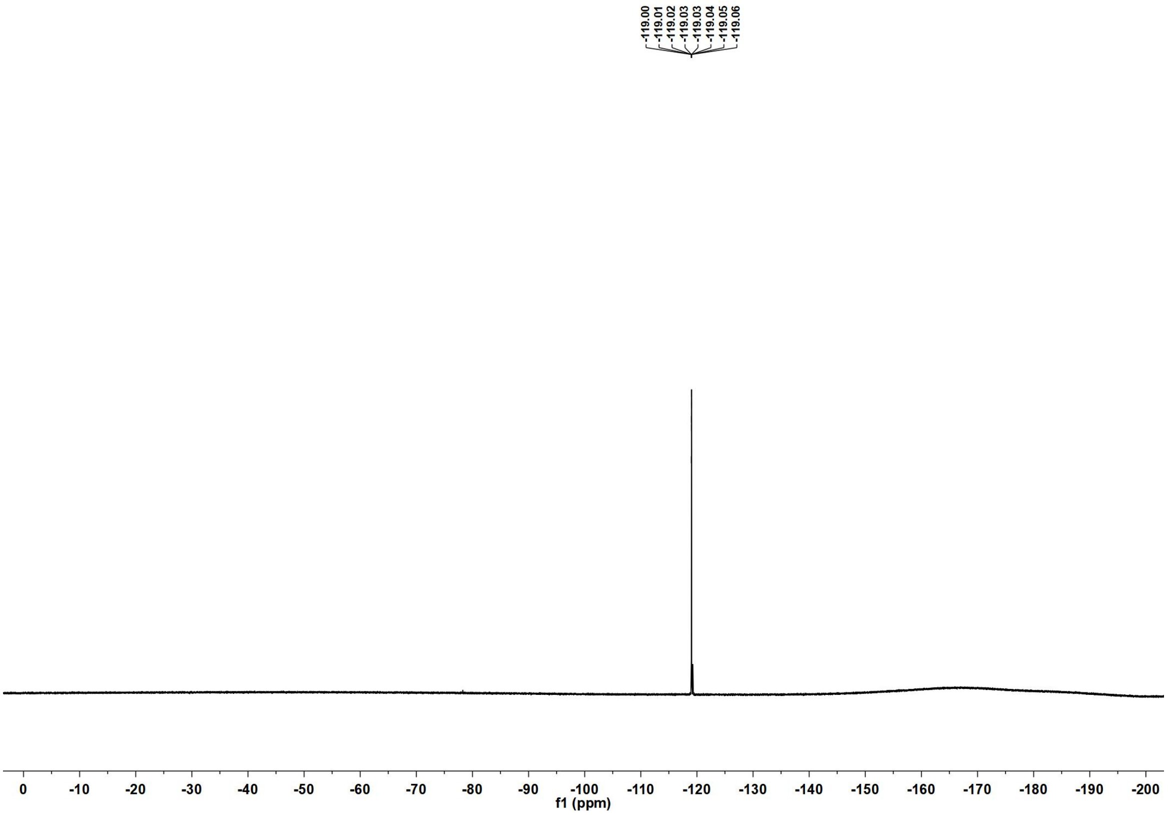

Supplementary Fig. 84. ^{19}F NMR spectra of compound **4k**. ^{19}F NMR (376 MHz, 298K) in $CDCl_3$

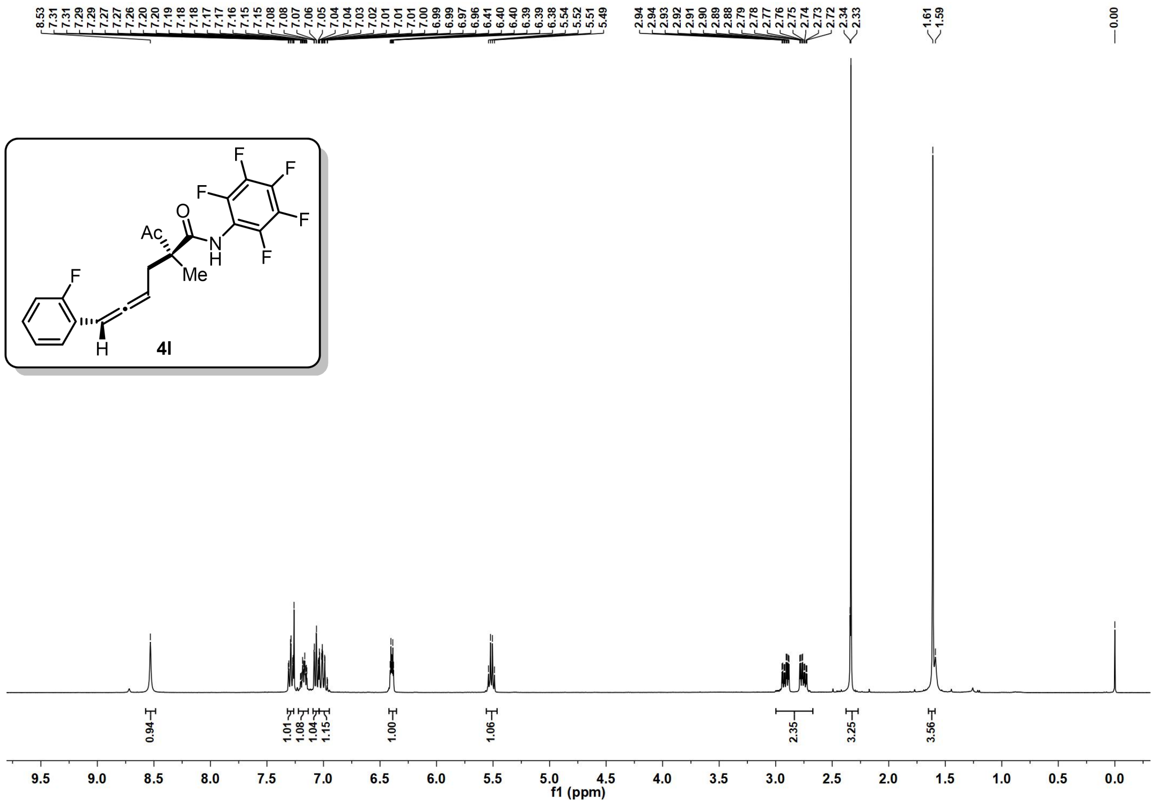

Supplementary Fig. 85. ^{1}H NMR spectra of compound **4l**. ^{1}H NMR (400 MHz, 298K) in $CDCl_3$

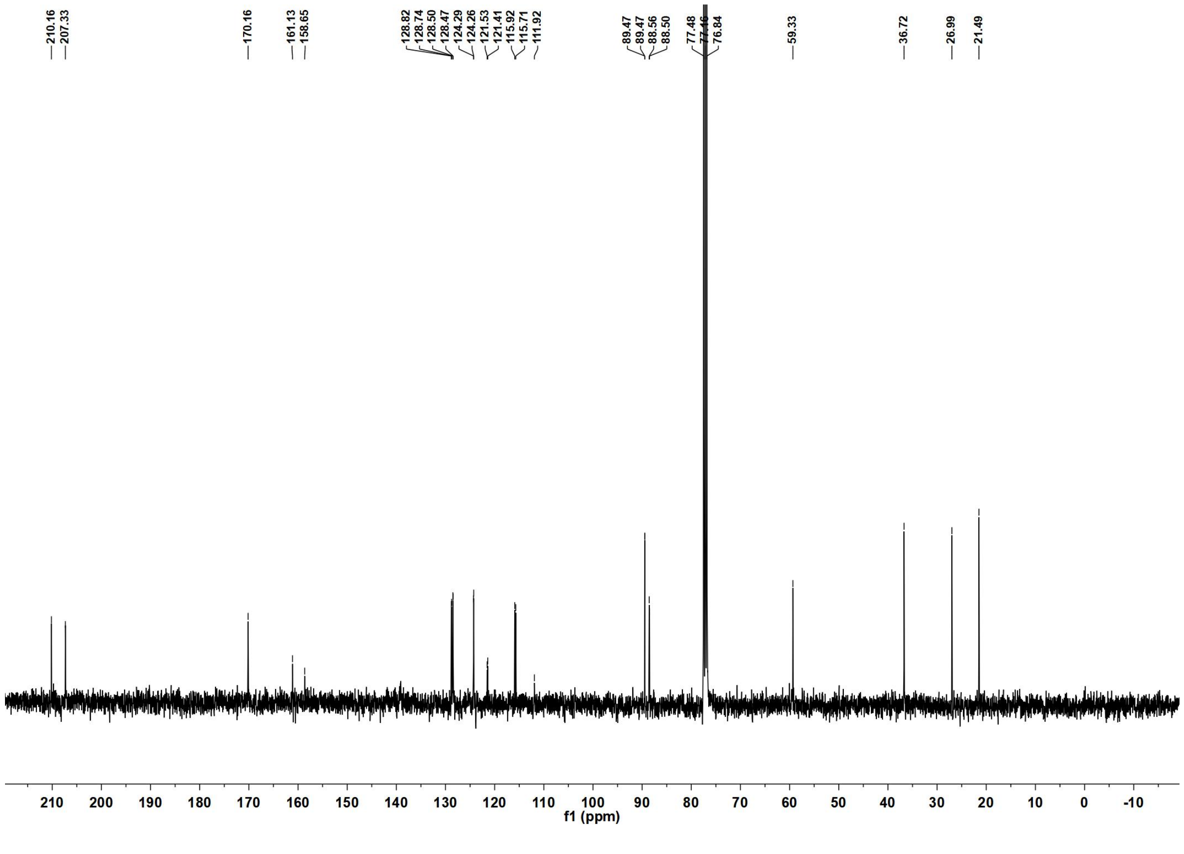

Supplementary Fig. 86. ^{13}C NMR spectra of compound **4l**. ^{13}C NMR (101 MHz, 298K) in $CDCl_3$

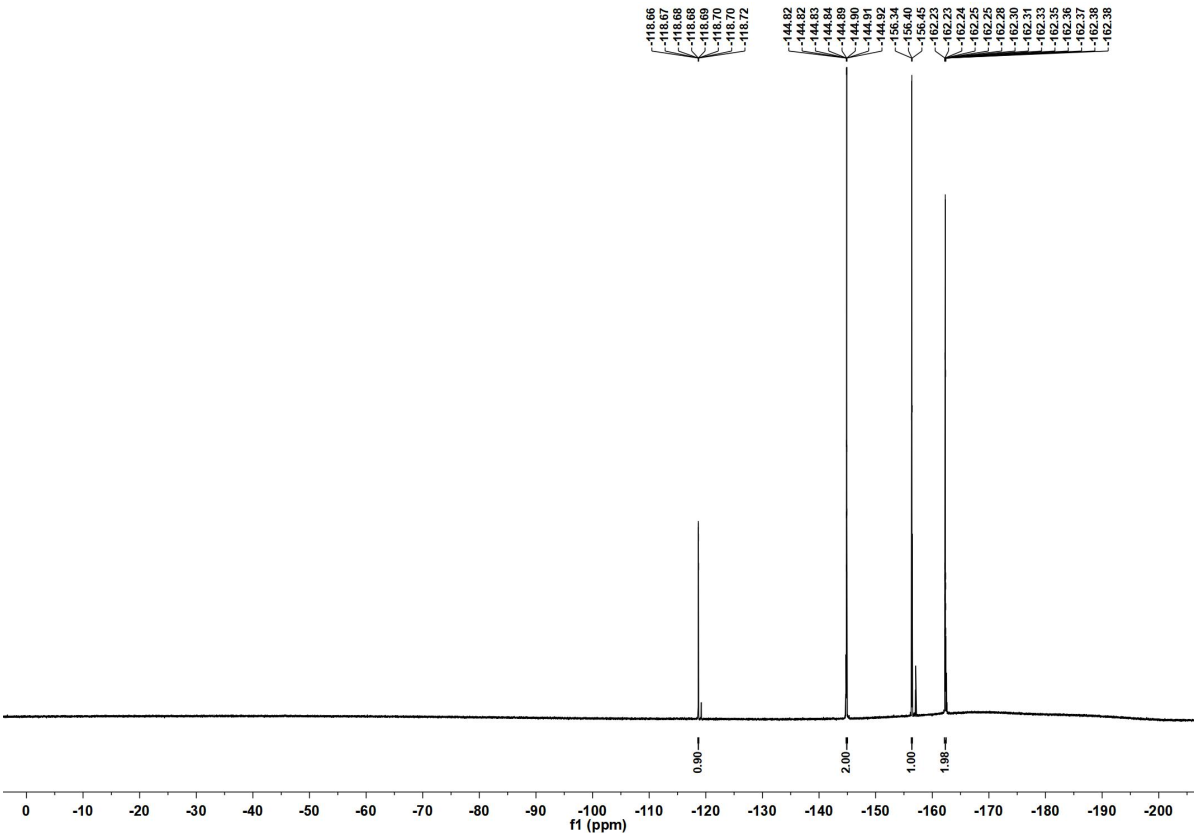

Supplementary Fig. 87. ^{19}F NMR spectra of compound **4l**. ^{19}F NMR (376 MHz, 298K) in $CDCl_3$

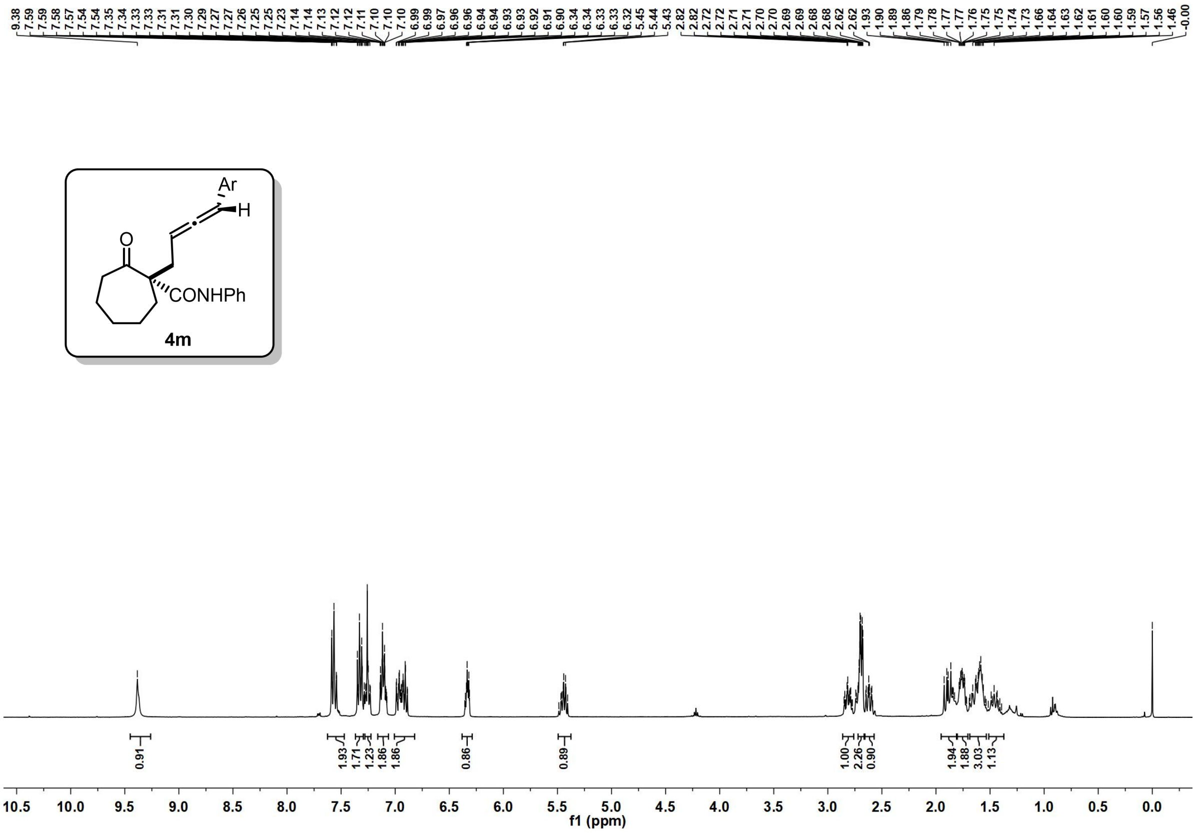

Supplementary Fig. 88. ^{1}H NMR spectra of compound **4m**. ^{1}H NMR (400 MHz, 298K) in $CDCl_3$

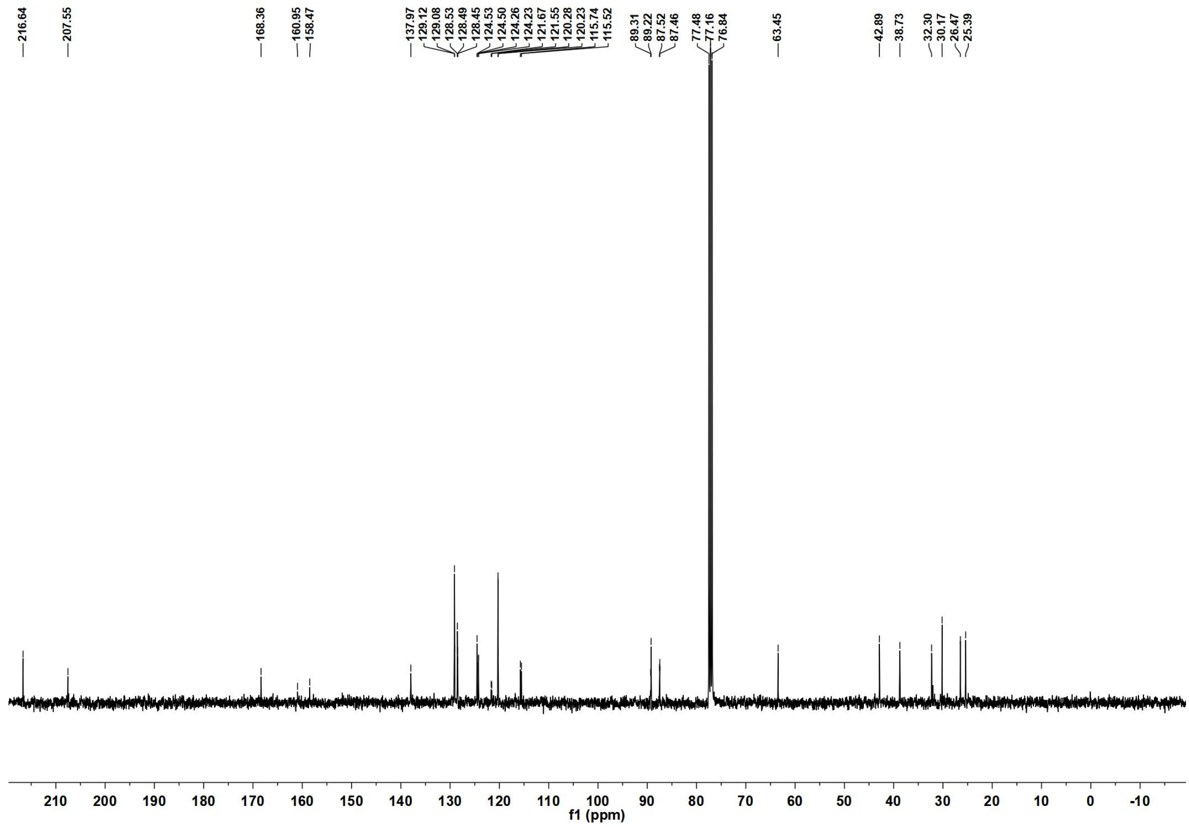

Supplementary Fig. 89. ^{13}C NMR spectra of compound **4m**. ^{13}C NMR (101 MHz, 298K) in $CDCl_3$

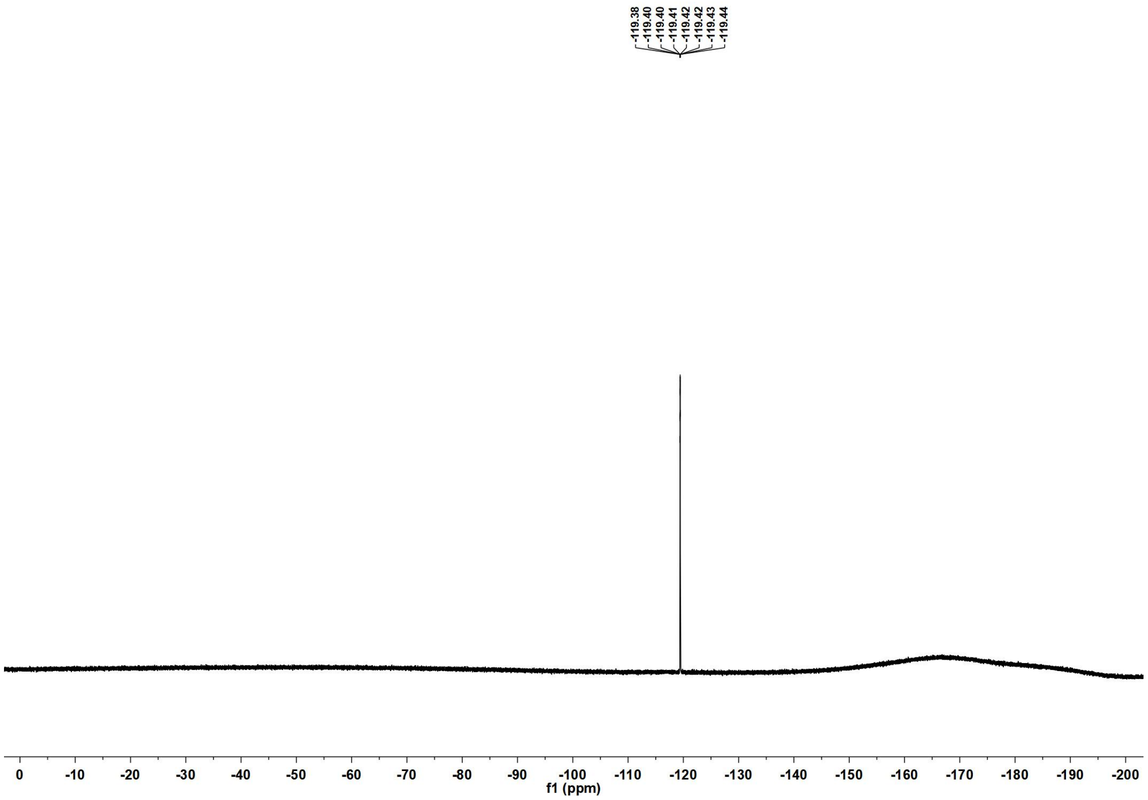

Supplementary Fig. 90. ^{19}F NMR spectra of compound **4m**. ^{19}F NMR (376 MHz, 298K) in $CDCl_3$

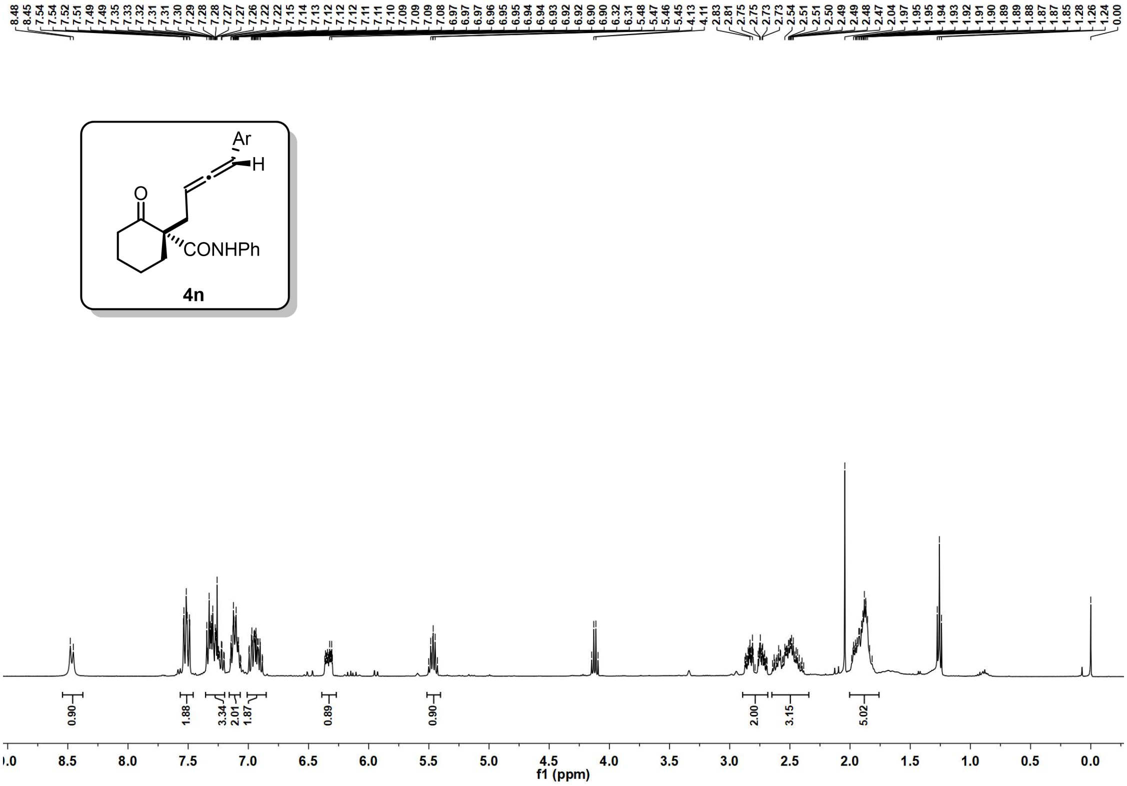

Supplementary Fig. 91. ^{1}H NMR spectra of compound **4n**. ^{1}H NMR (400 MHz, 298K) in $CDCl_3$

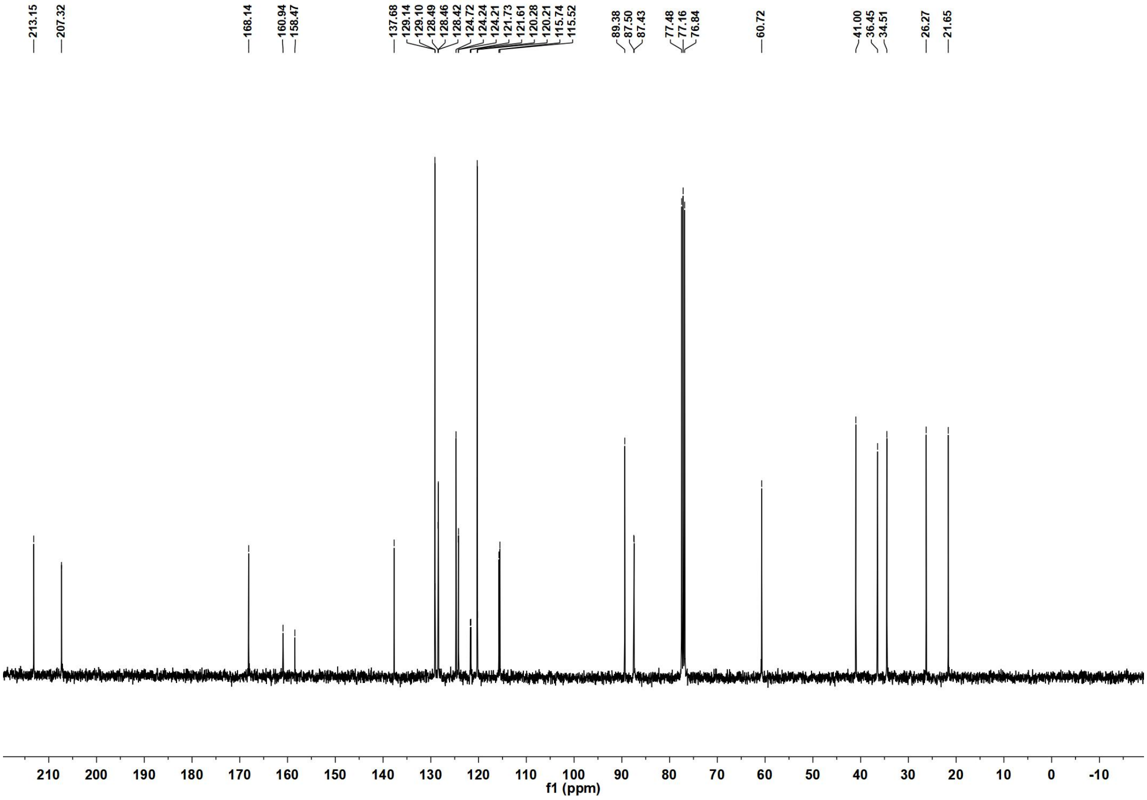

Supplementary Fig. 92. ^{13}C NMR spectra of compound **4n**. ^{13}C NMR (101 MHz, 298K) in $CDCl_3$

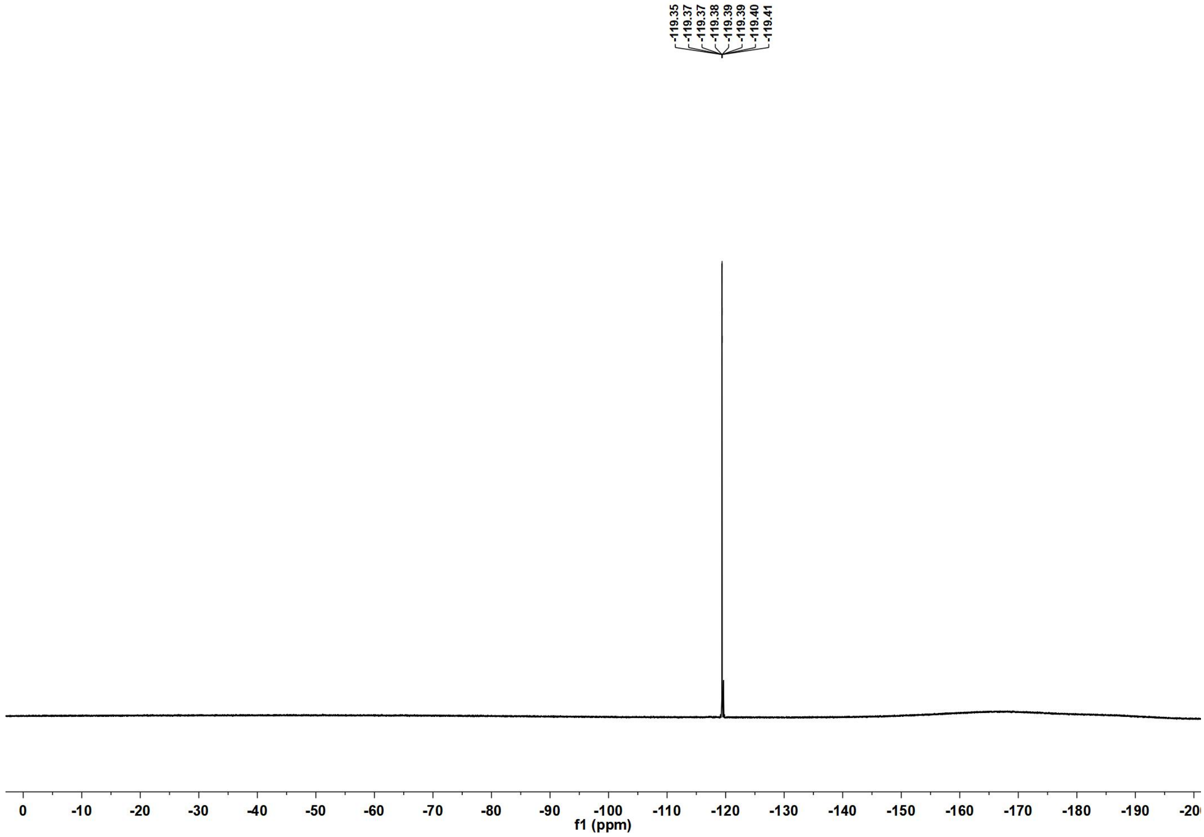

Supplementary Fig. 93. ^{19}F NMR spectra of compound **4n**. ^{19}F NMR (376 MHz, 298K) in $CDCl_3$

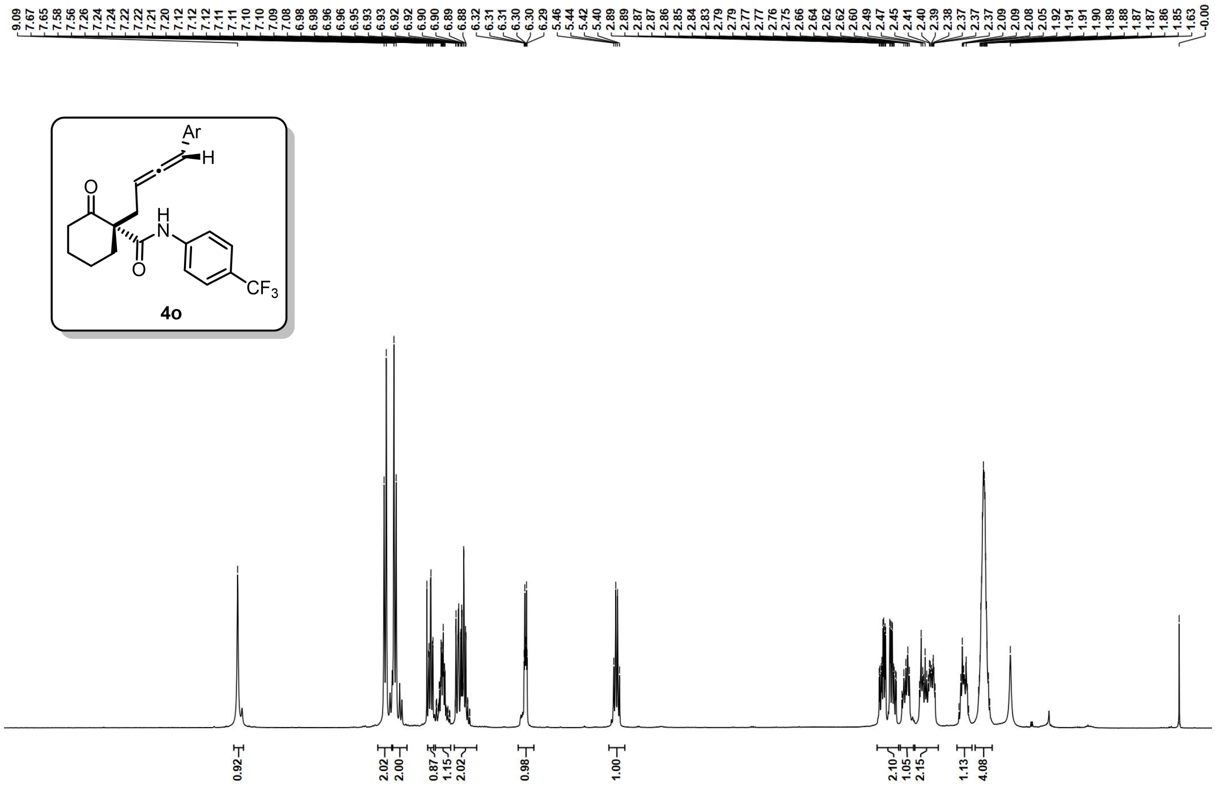

Supplementary Fig. 94. ^{1}H NMR spectra of compound **4o**. ^{1}H NMR (400 MHz, 298K) in $CDCl_3$

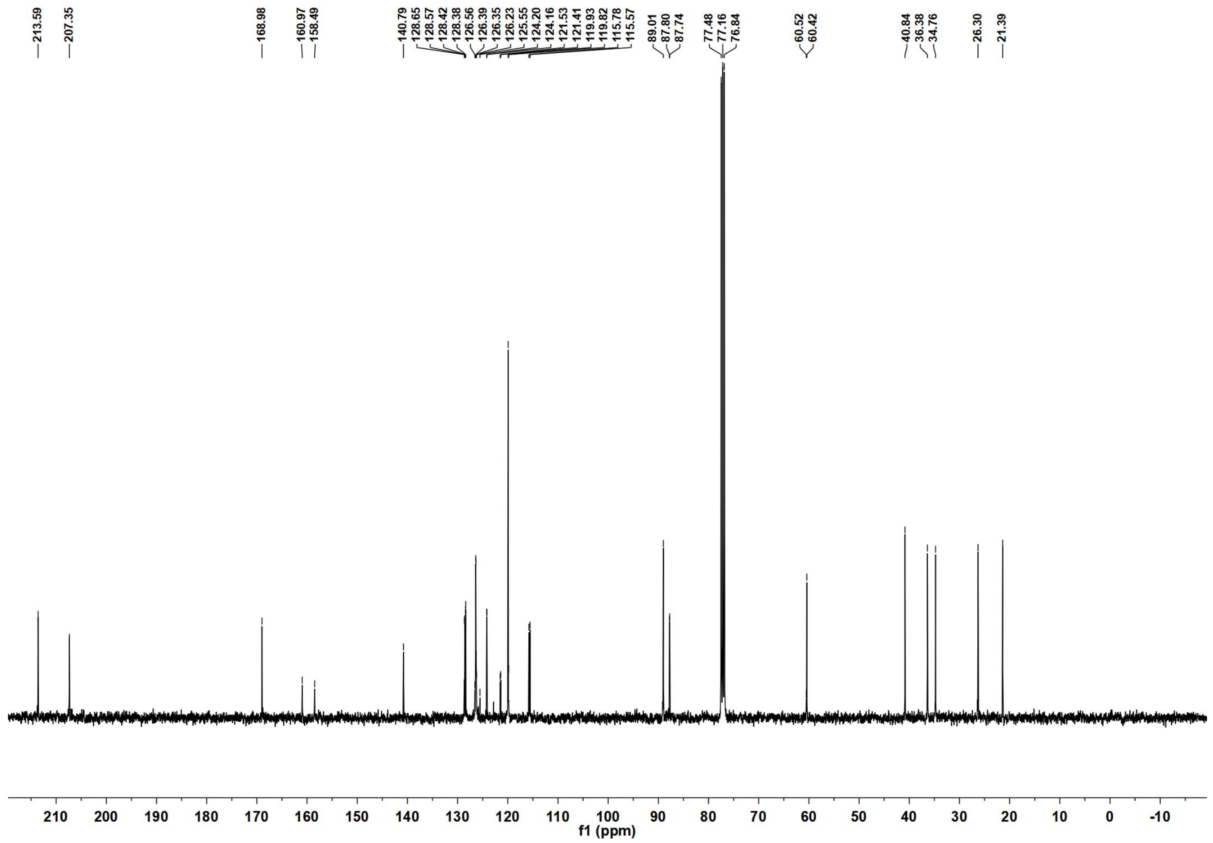

Supplementary Fig. 95. ^{13}C NMR spectra of compound **4o**. ^{13}C NMR (101 MHz, 298K) in $CDCl_3$

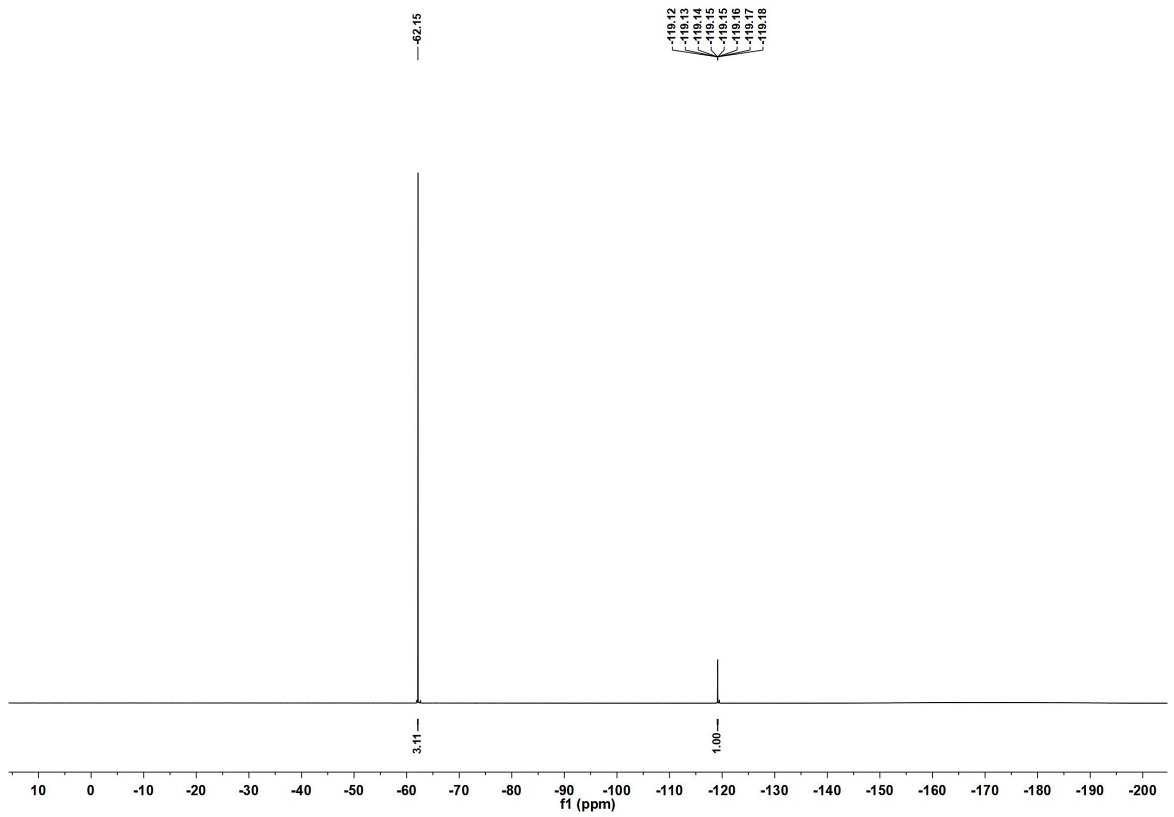

Supplementary Fig. 96. ^{19}F NMR spectra of compound **4o**. ^{19}F NMR (376 MHz, 298K) in $CDCl_3$

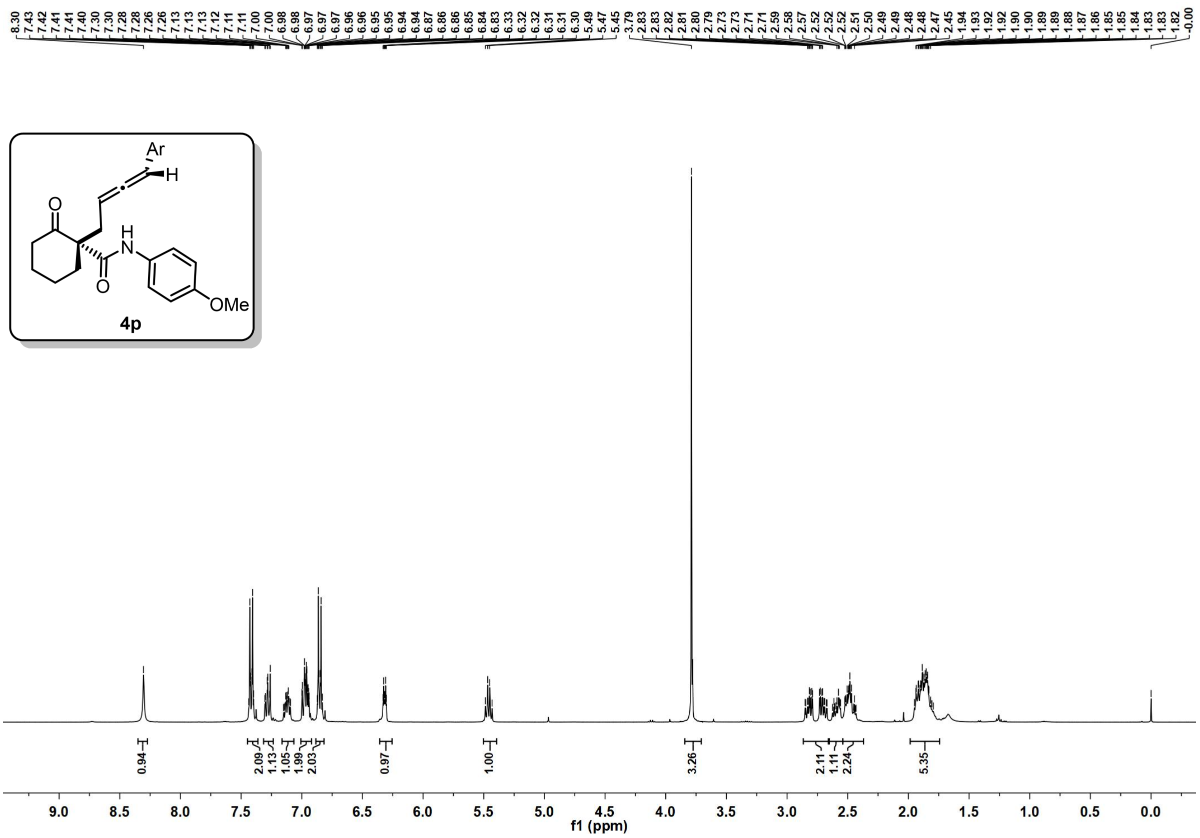

Supplementary Fig. 97. ^{1}H NMR spectra of compound **4p**. ^{1}H NMR (400 MHz, 298K) in $CDCl_3$

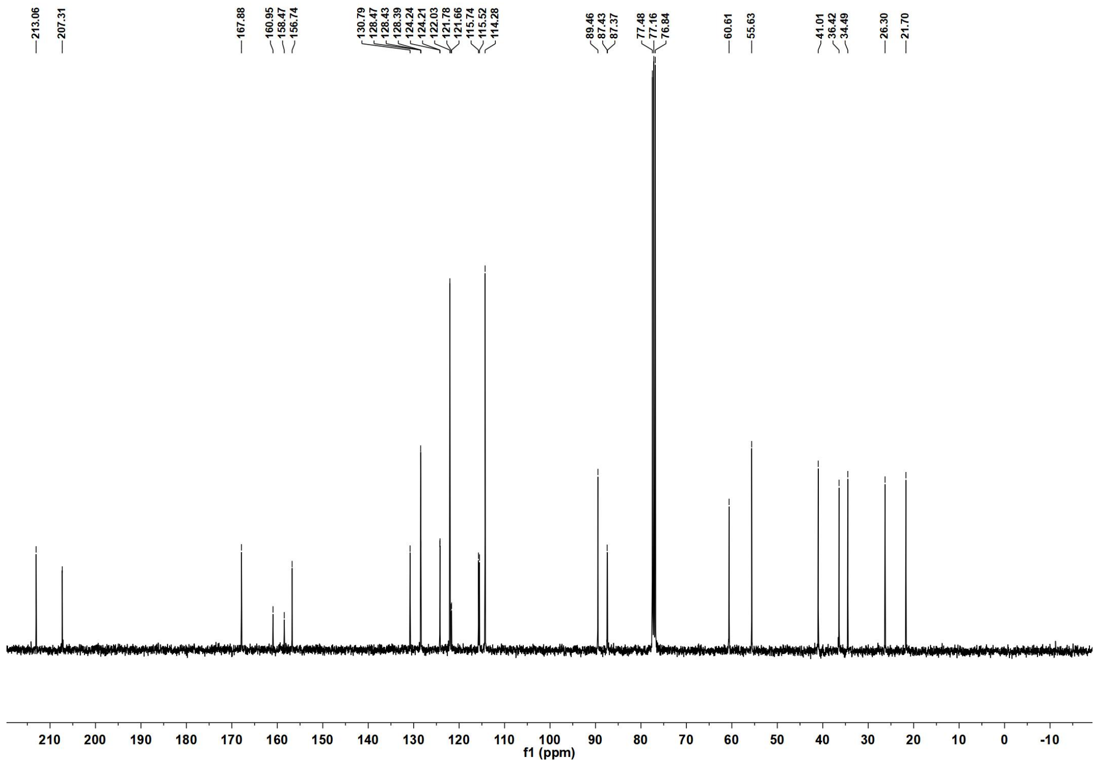

Supplementary Fig. 98. ^{13}C NMR spectra of compound **4p**. ^{13}C NMR (101 MHz, 298K) in $CDCl_3$

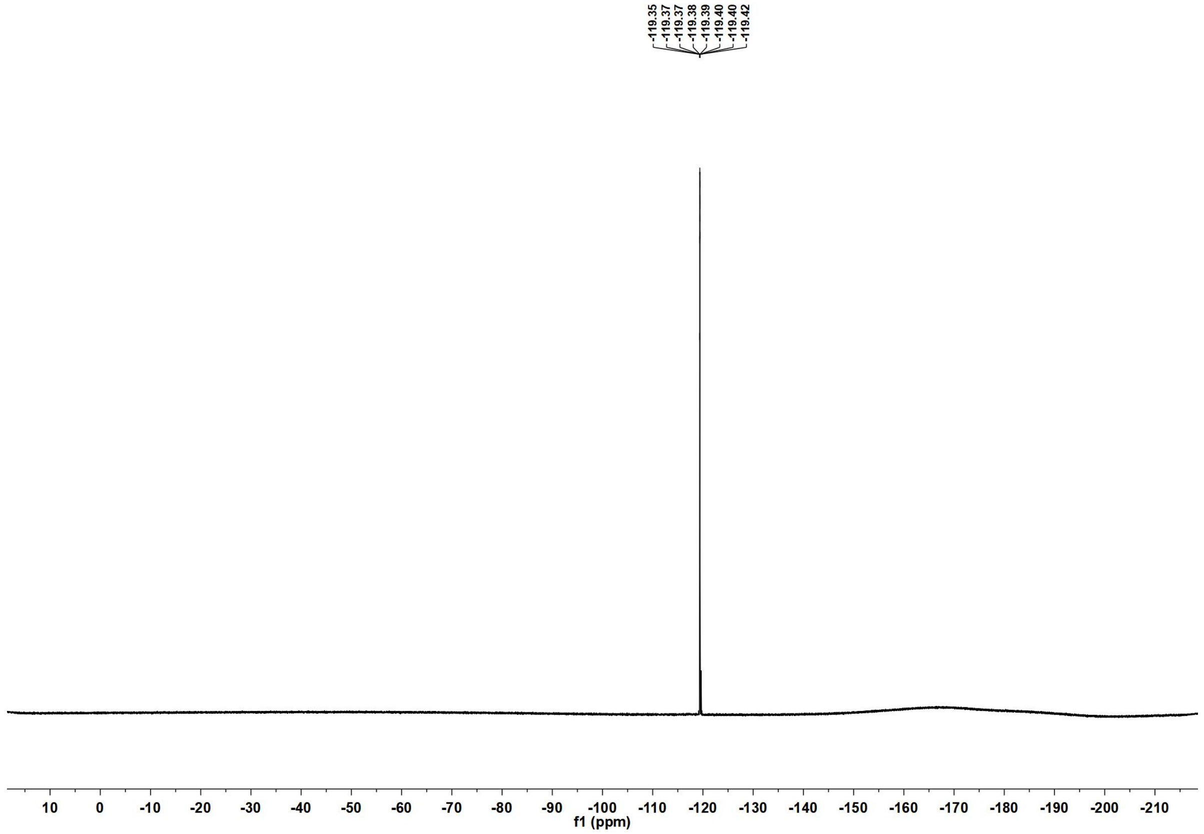

Supplementary Fig. 99. ^{19}F NMR spectra of compound **4p**. ^{19}F NMR (376 MHz, 298K) in $CDCl_3$

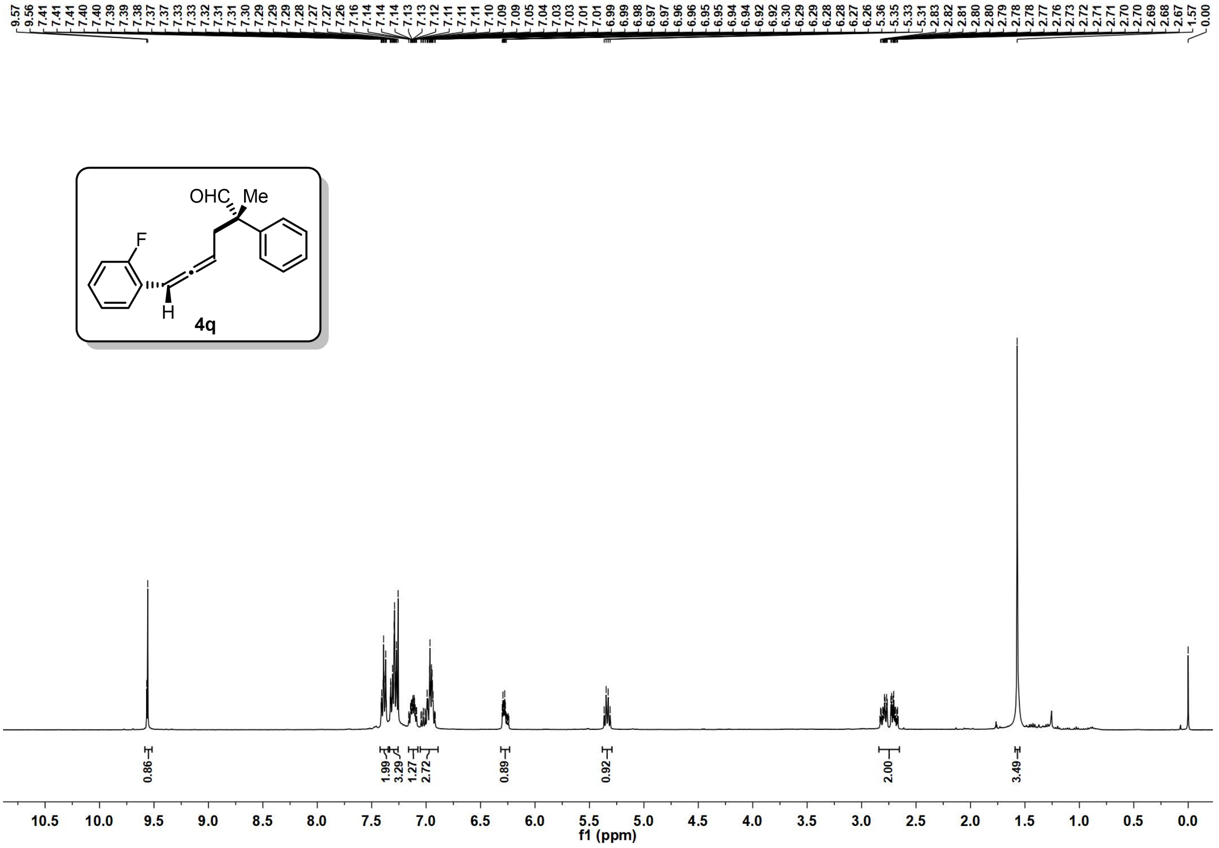

Supplementary Fig. 100. ^{1}H NMR spectra of compound **4q**. ^{1}H NMR (400 MHz, 298K) in $CDCl_3$

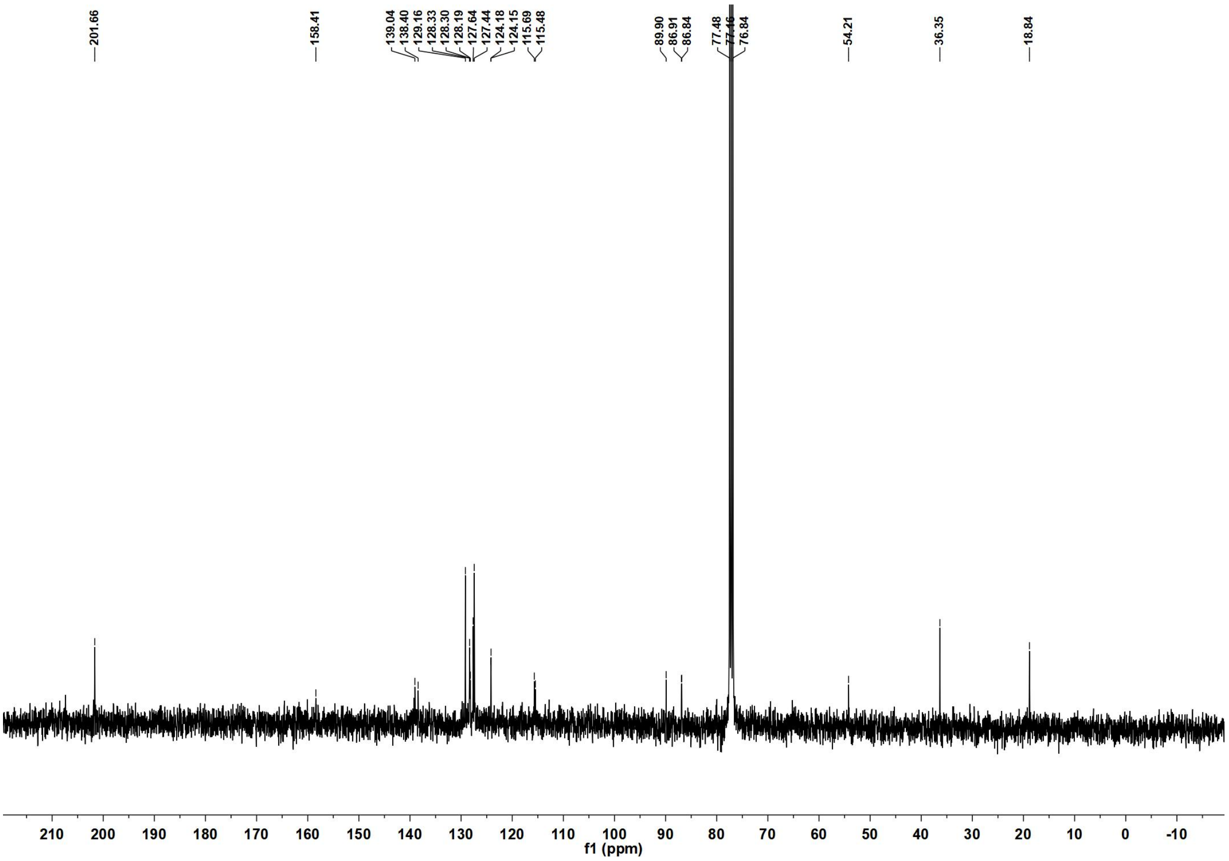

Supplementary Fig. 101. ^{13}C NMR spectra of compound **4q**. ^{13}C NMR (101 MHz, 298K) in $CDCl_3$

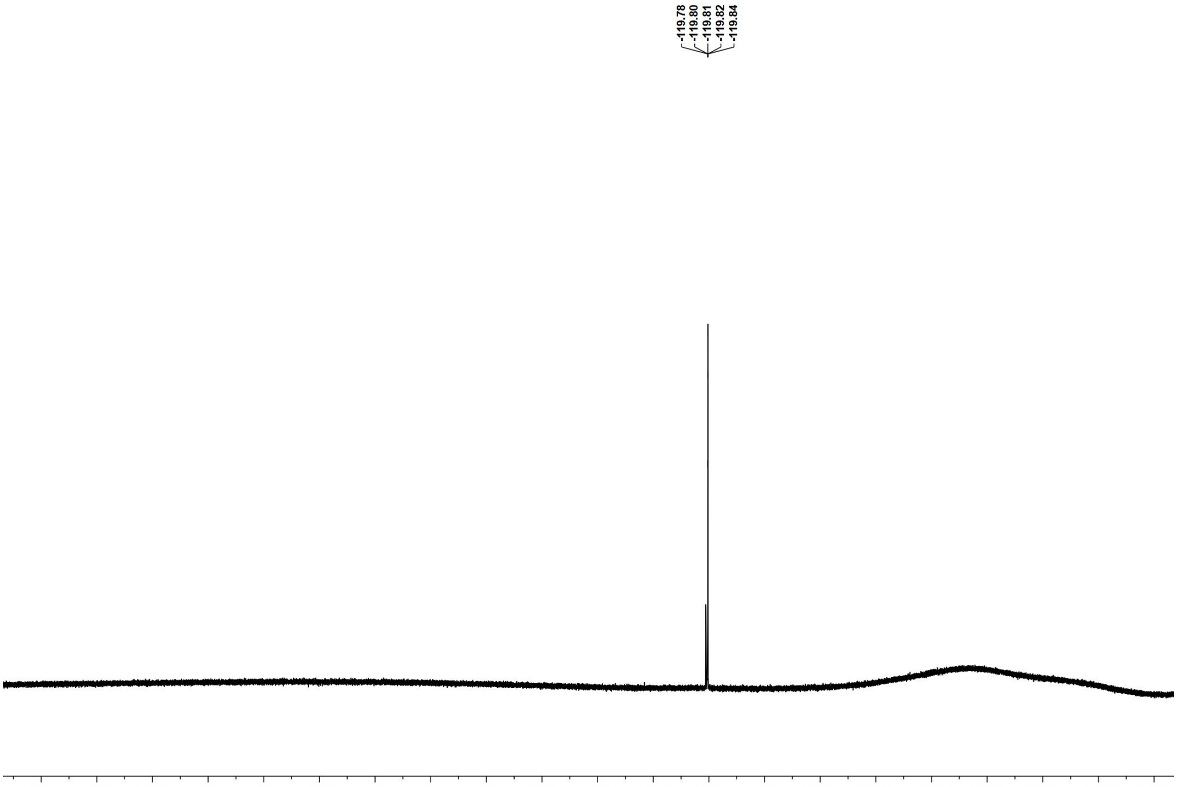

Supplementary Fig. 102. ^{19}F NMR spectra of compound **4q**. ^{19}F NMR (376 MHz, 298K) in $CDCl_3$

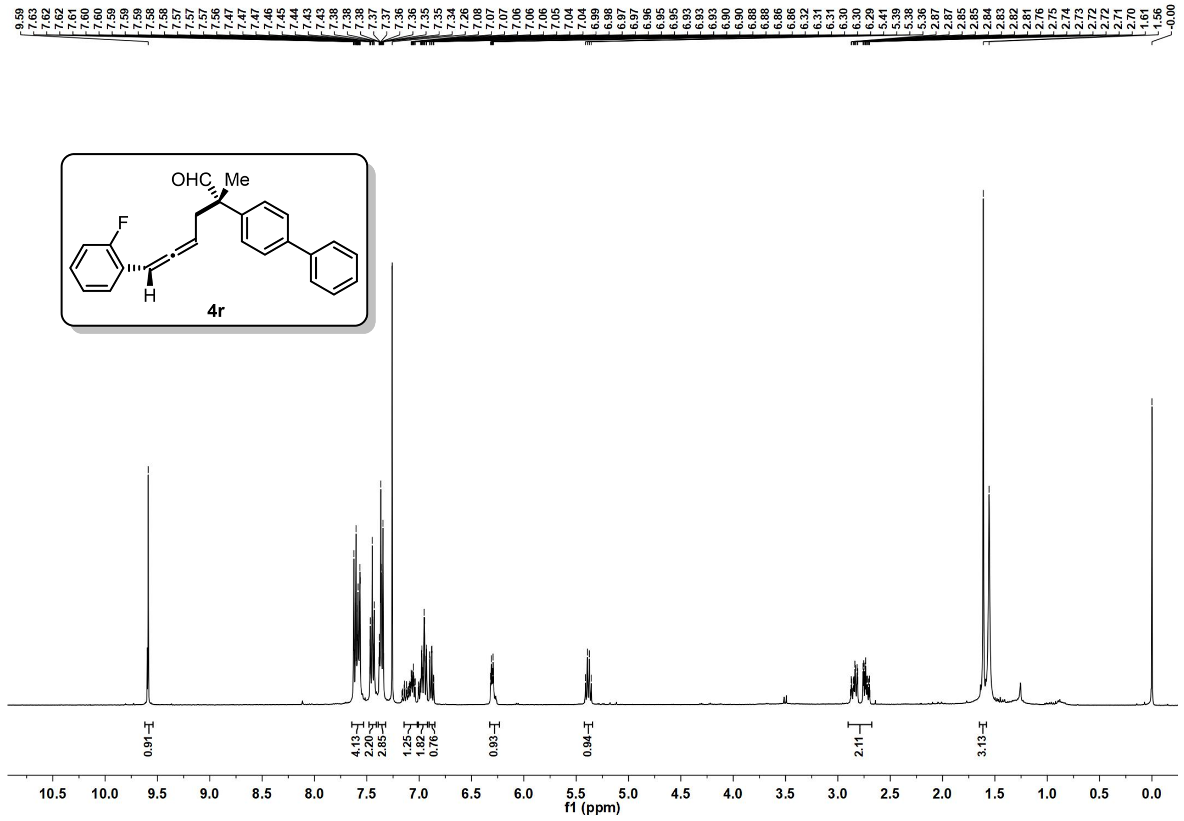

Supplementary Fig. 103. ^{1}H NMR spectra of compound **4r**. ^{1}H NMR (400 MHz, 298K) in $CDCl_3$

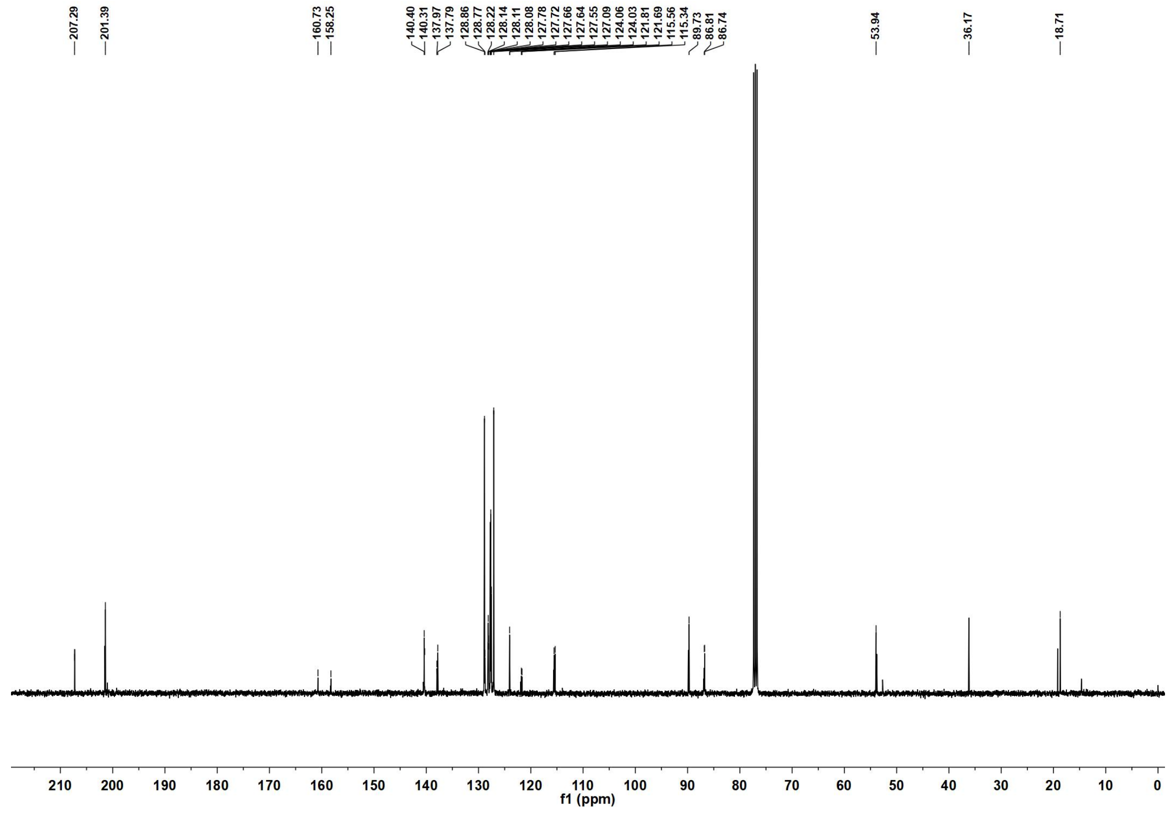

Supplementary Fig. 104. ^{13}C NMR spectra of compound **4r**. ^{13}C NMR (101 MHz, 298K) in $CDCl_3$

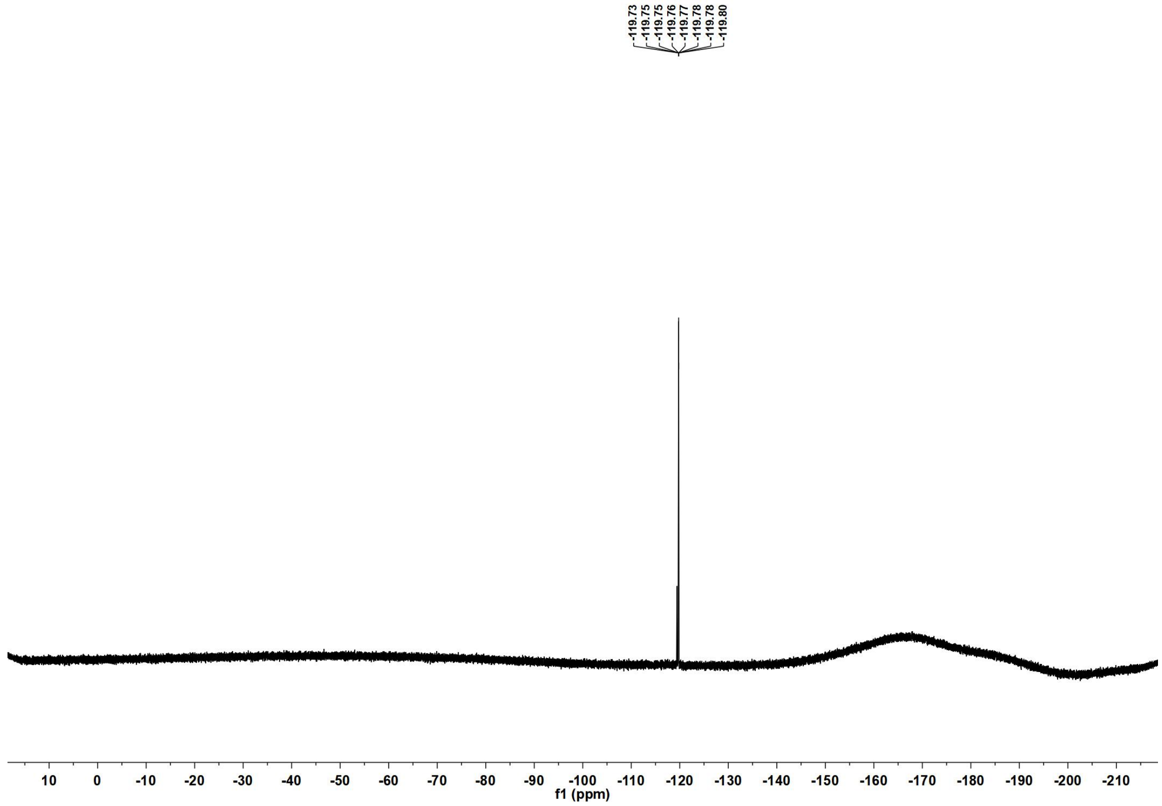

Supplementary Fig. 105. ^{19}F NMR spectra of compound **4r**. ^{19}F NMR (376 MHz, 298K) in $CDCl_3$

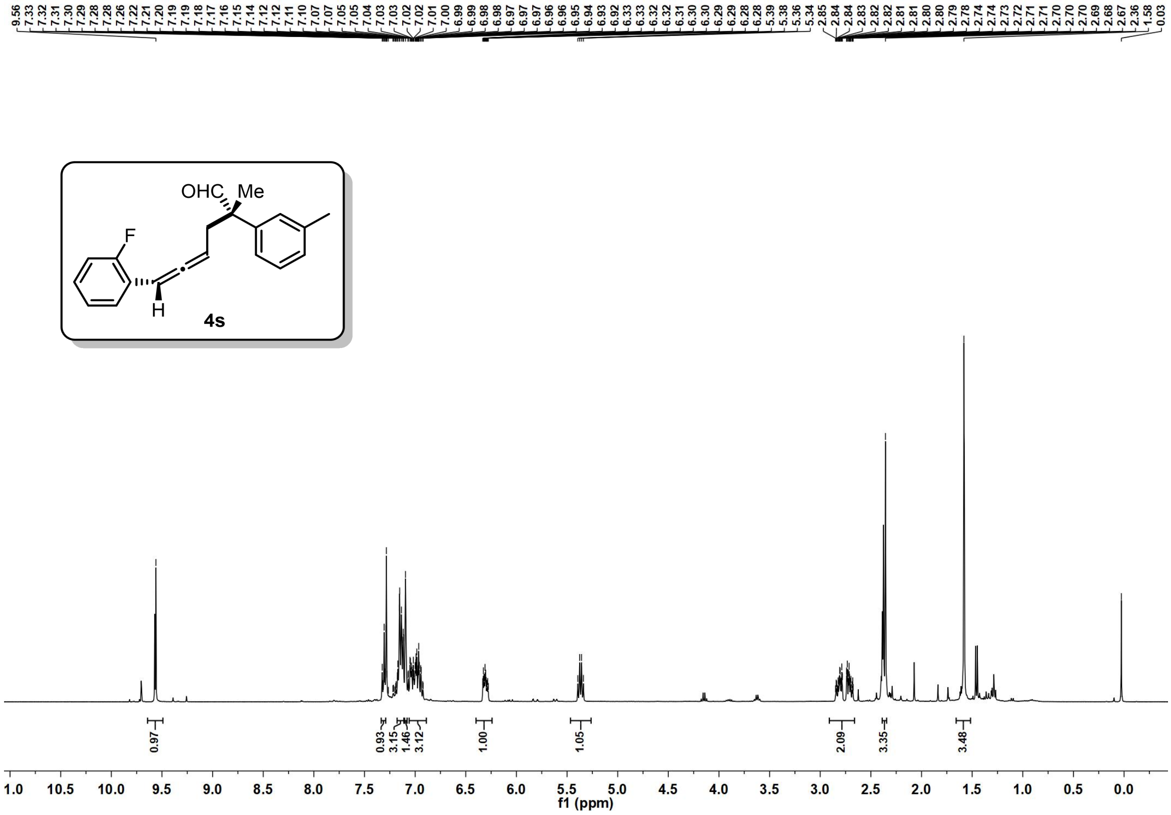

Supplementary Fig. 106. ^1H NMR spectra of compound **4s**. ^1H NMR (400 MHz, 298K) in $CDCl_3$

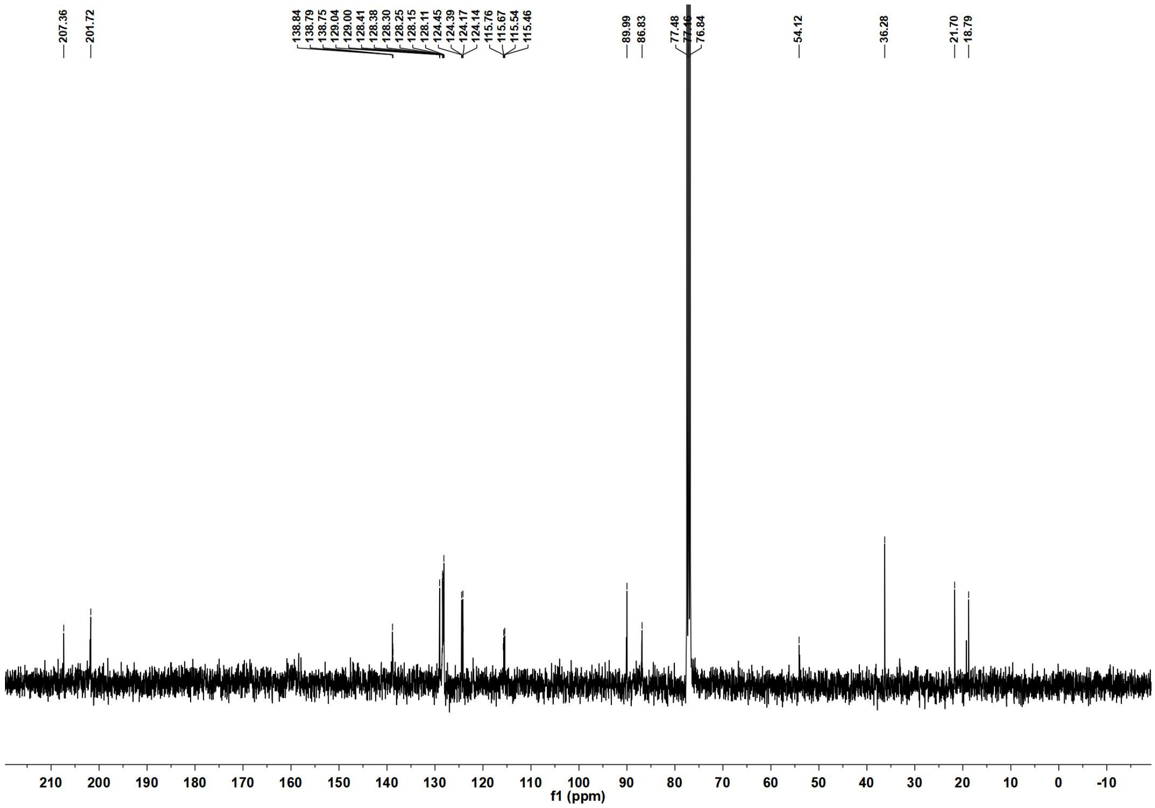

Supplementary Fig. 107. ^{13}C NMR spectra of compound **4s**. ^{13}C NMR (101 MHz, 298K) in $CDCl_3$

3

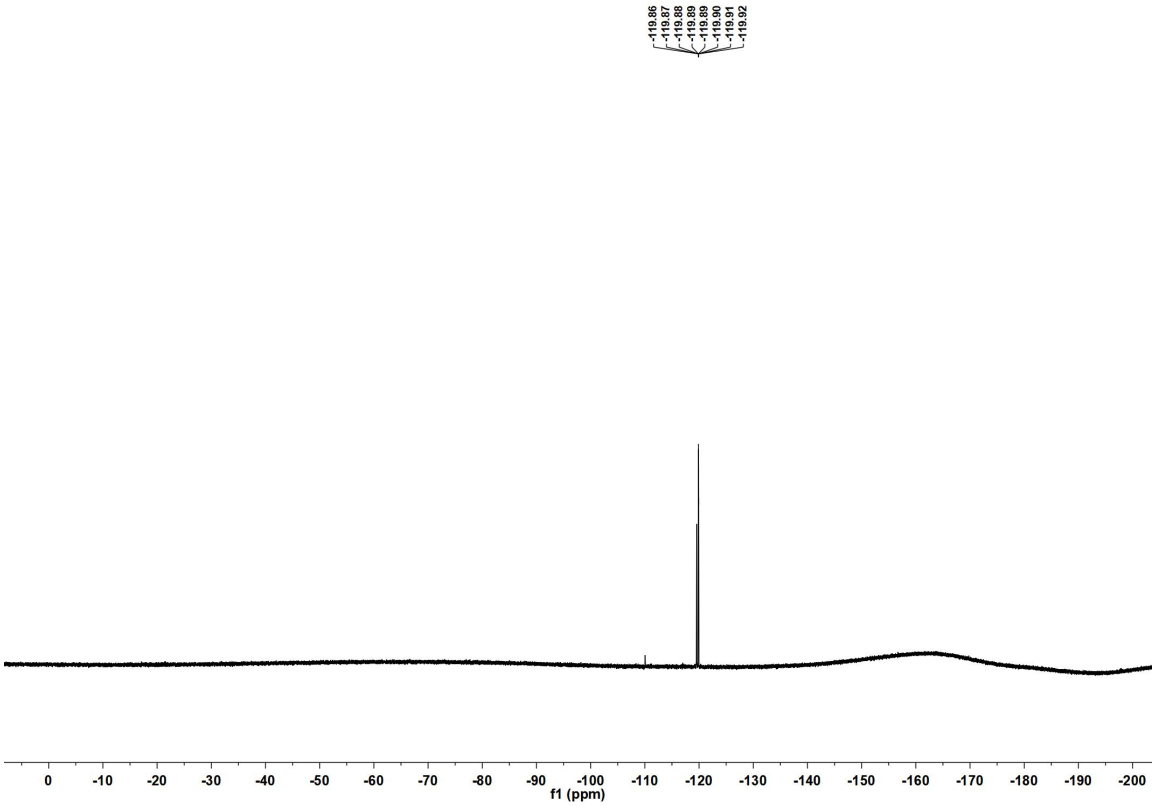

Supplementary Fig. 108. ^{19}F NMR spectra of compound **4s**. ^{19}F NMR (376 MHz, 298K) in $CDCl_3$

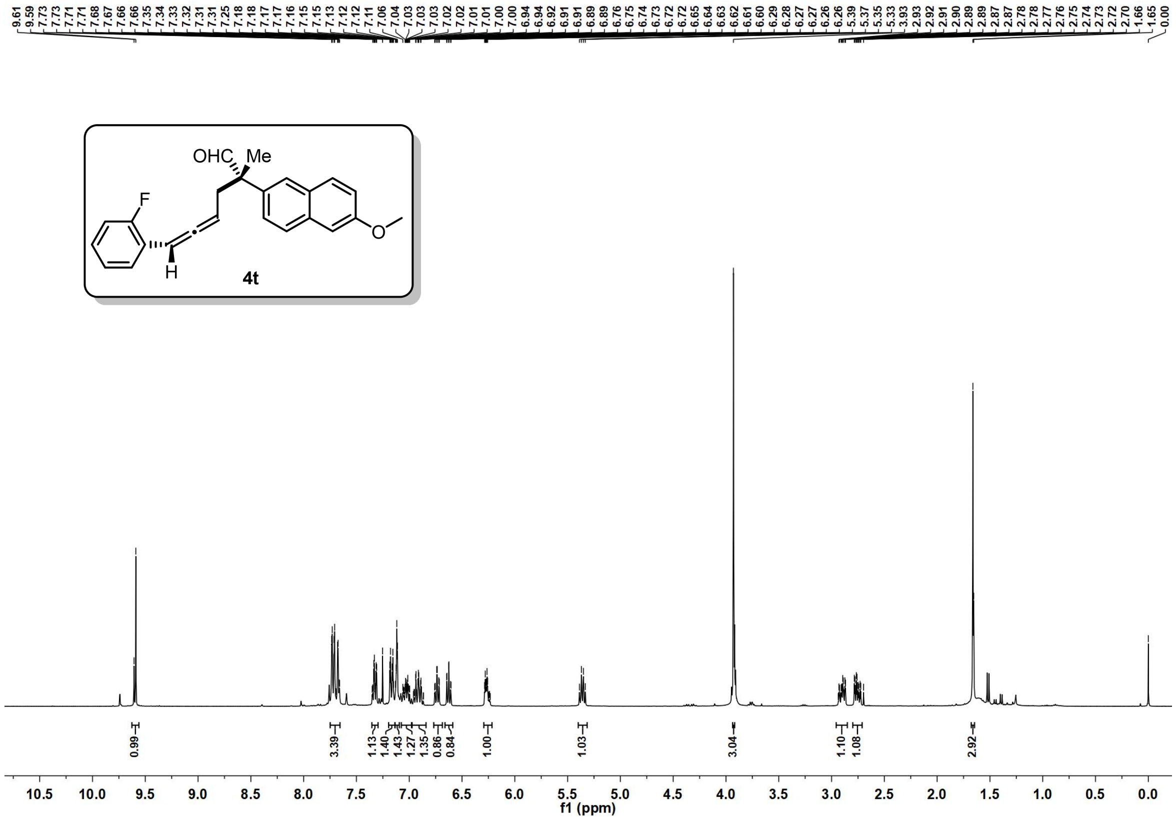

Supplementary Fig. 109. ^{1}H NMR spectra of compound **4t**. ^{1}H NMR (400 MHz, 298K) in $CDCl_3$

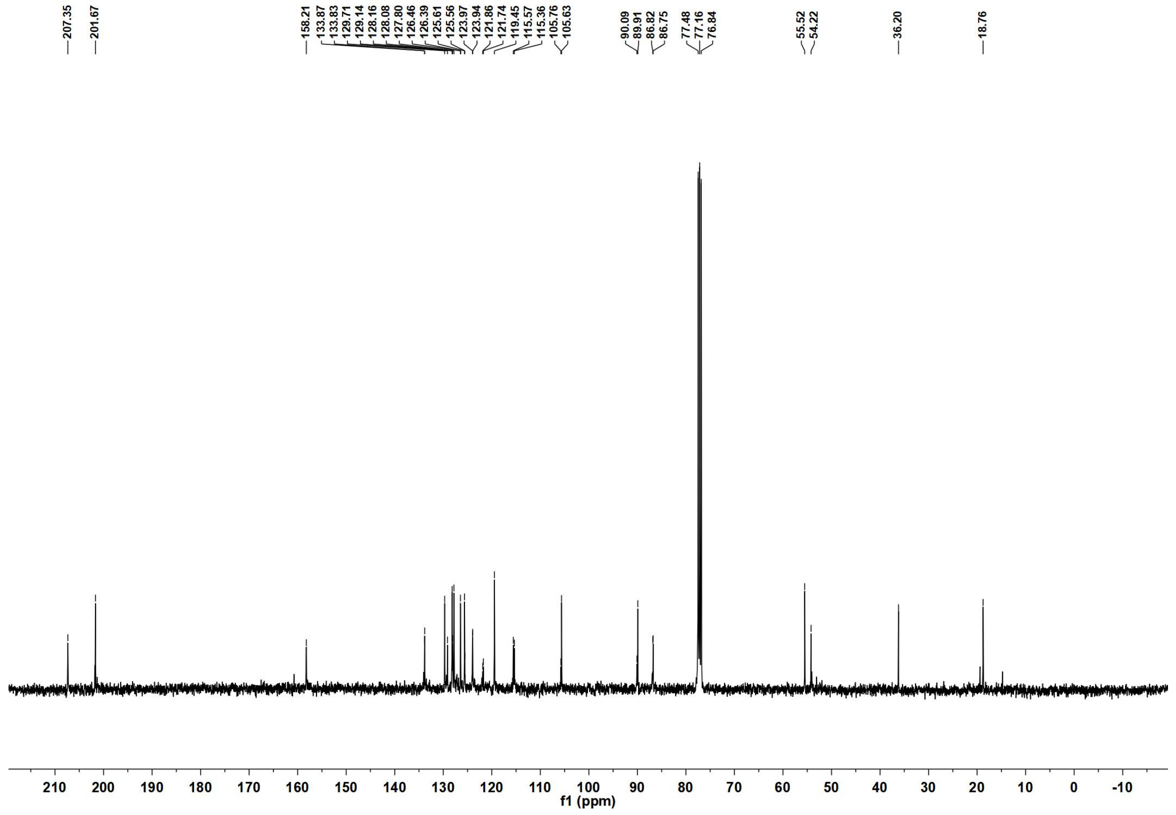

SSupplementary Fig. 110. ^{13}C NMR spectra of compound **4t**. ^{13}C NMR (101 MHz, 298K) in $CDCl_3$

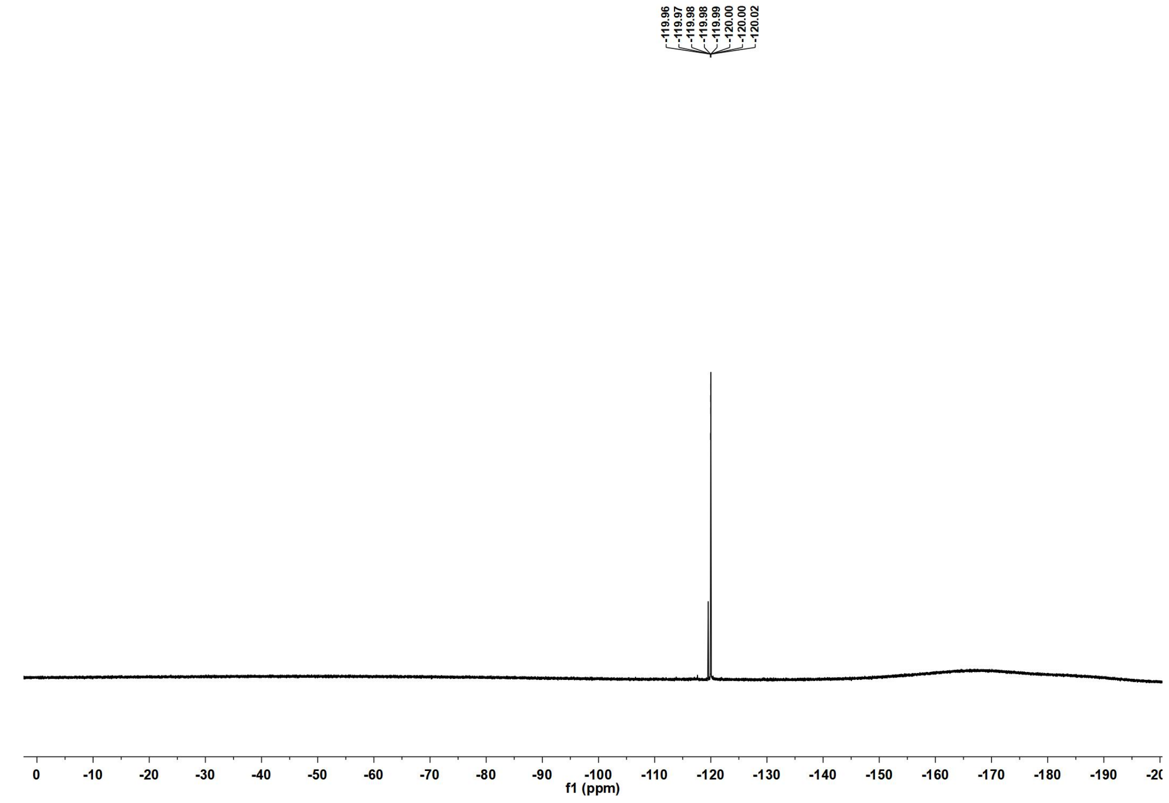

Supplementary Fig. 111. ^{19}F NMR spectra of compound **4t**. ^{19}F NMR (376 MHz, 298K) in $CDCl_3$

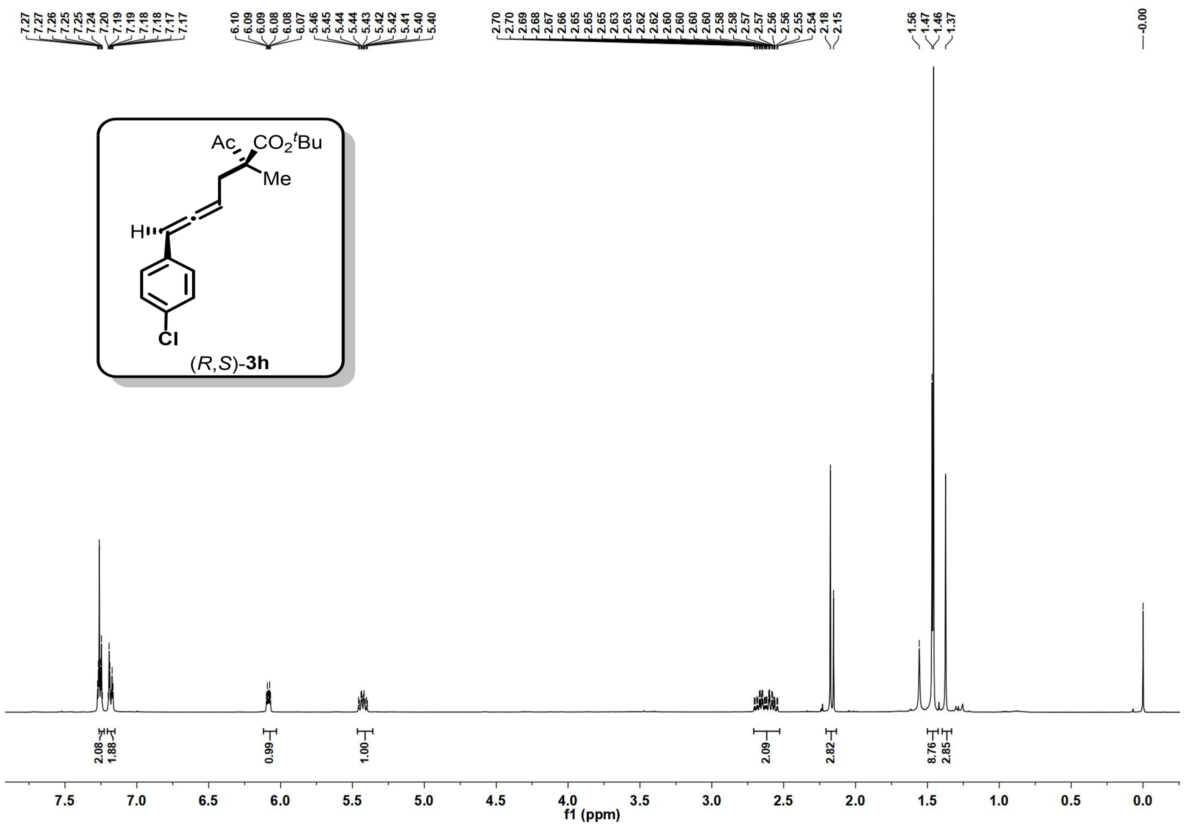

Supplementary Fig. 112. ^{1}H NMR spectra of compound (*R*,*S*)-**3h**. ^{1}H NMR (400 MHz, 298K) in $CDCl_3$

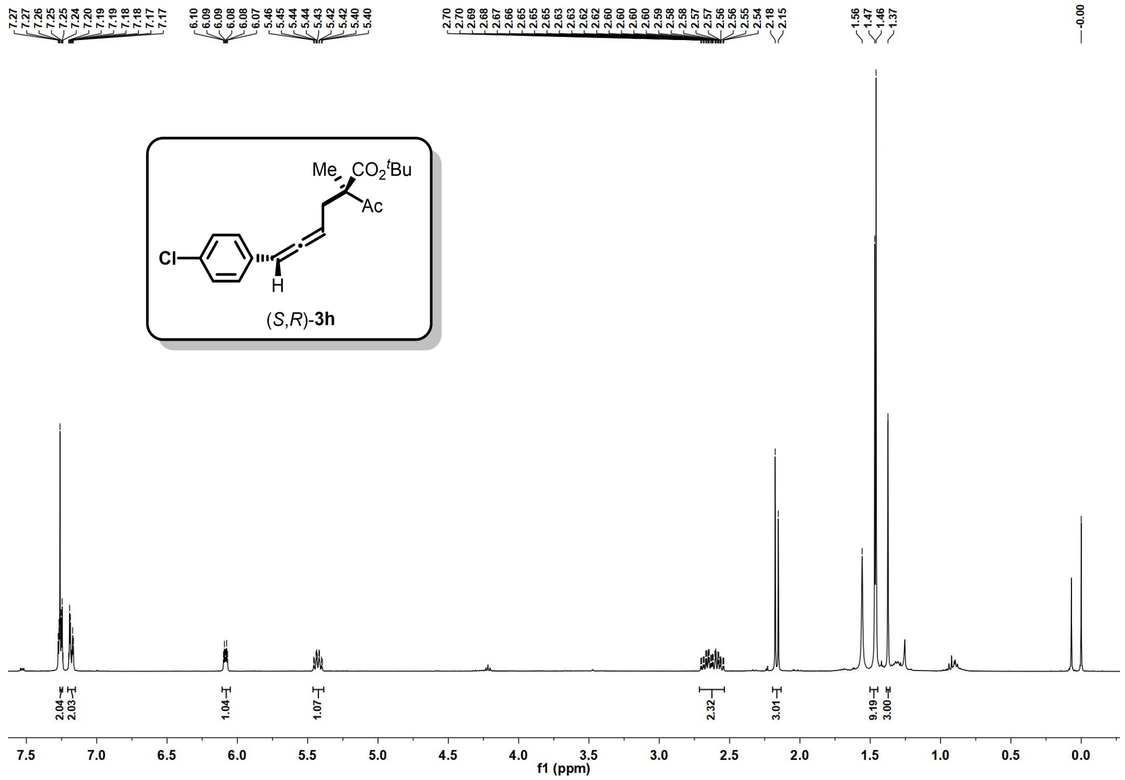

Supplementary Fig. 113. ^{1}H NMR spectra of compound (*S*,*R*)-**3h**. ^{1}H NMR (400 MHz, 298K) in $CDCl_3$

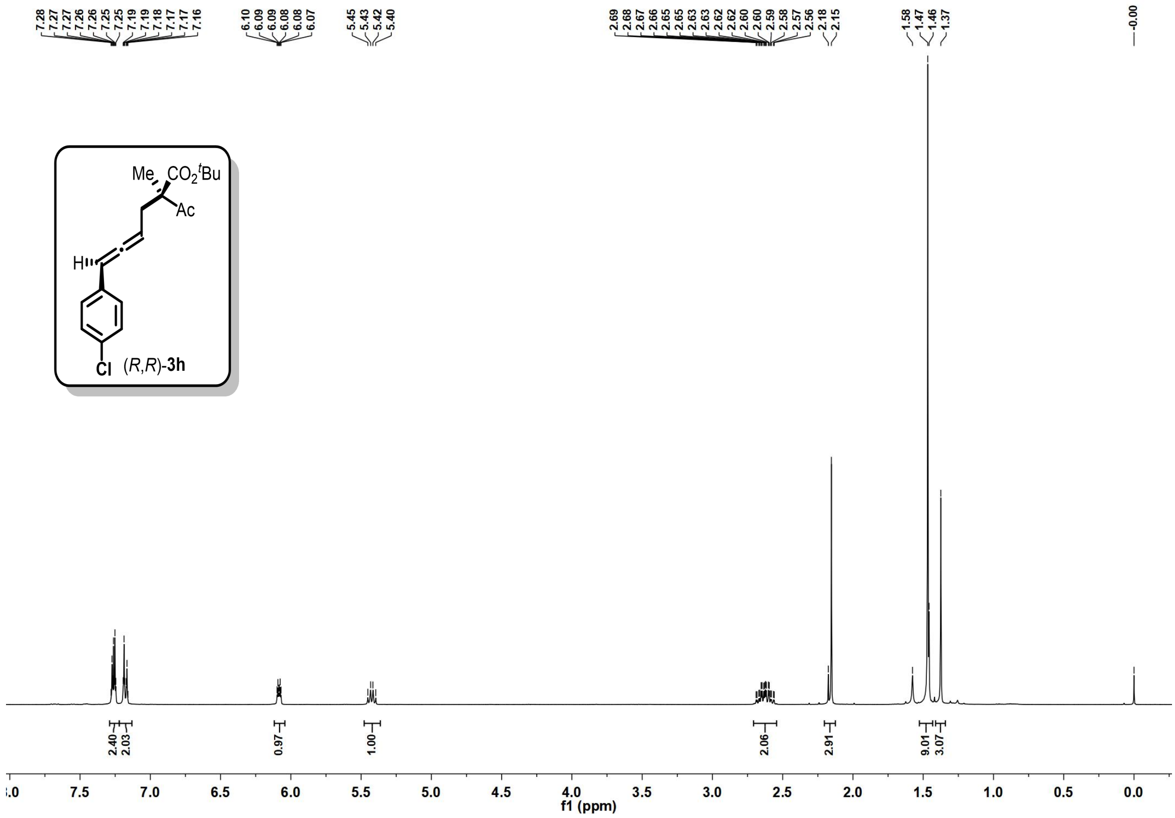

Supplementary Fig. 114. ^{1}H NMR spectra of compound (*R*,*R*)-**3h**. ^{1}H NMR (400 MHz, 298K) in $CDCl_3$

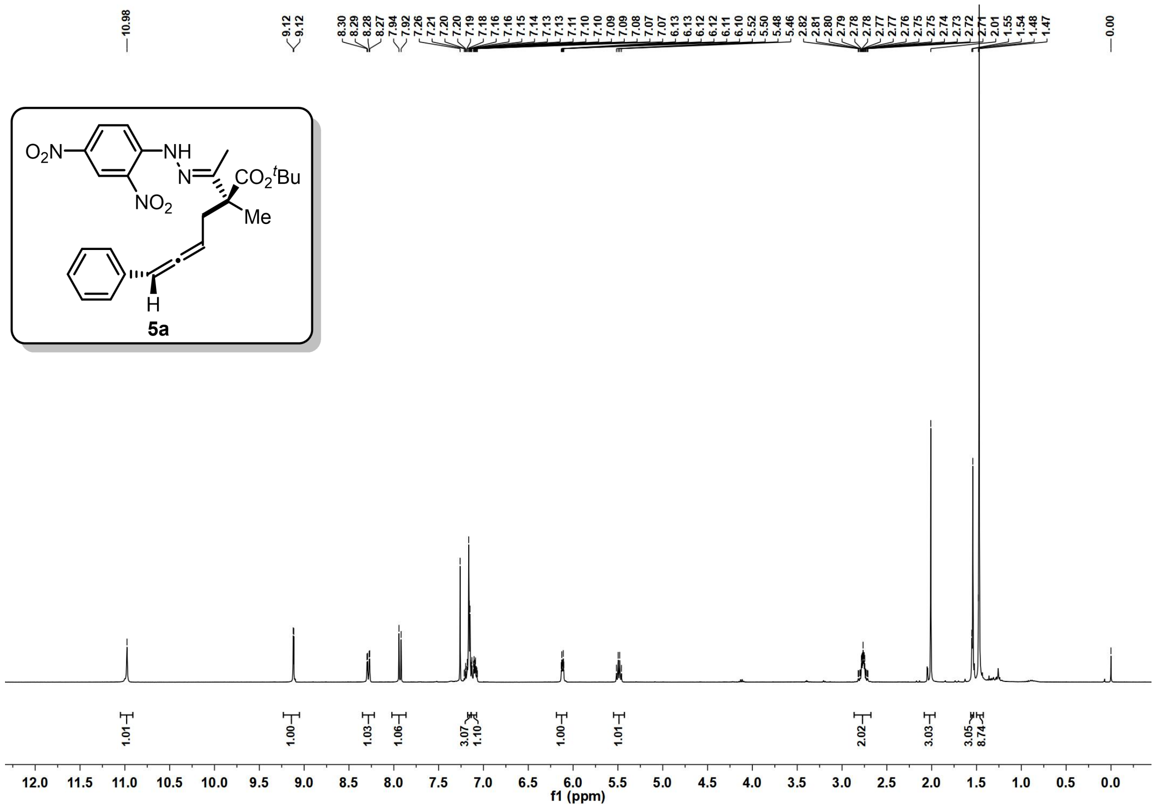

Supplementary Fig. 115. ^{1}H NMR spectra of compound **5a**. ^{1}H NMR (400 MHz, 298K) in $CDCl_3$

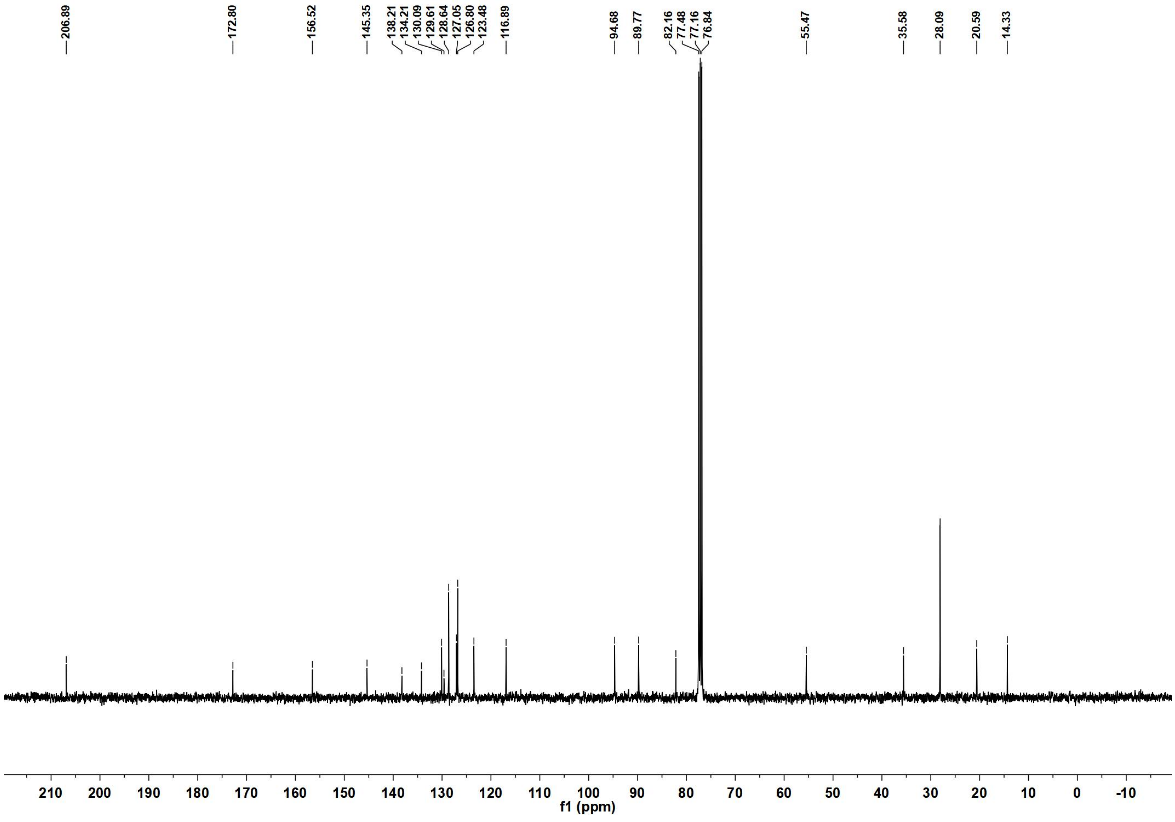

Supplementary Fig. 116. ^{13}C NMR spectra of compound **5a**. ^{13}C NMR (101 MHz, 298K) in $CDCl_3$

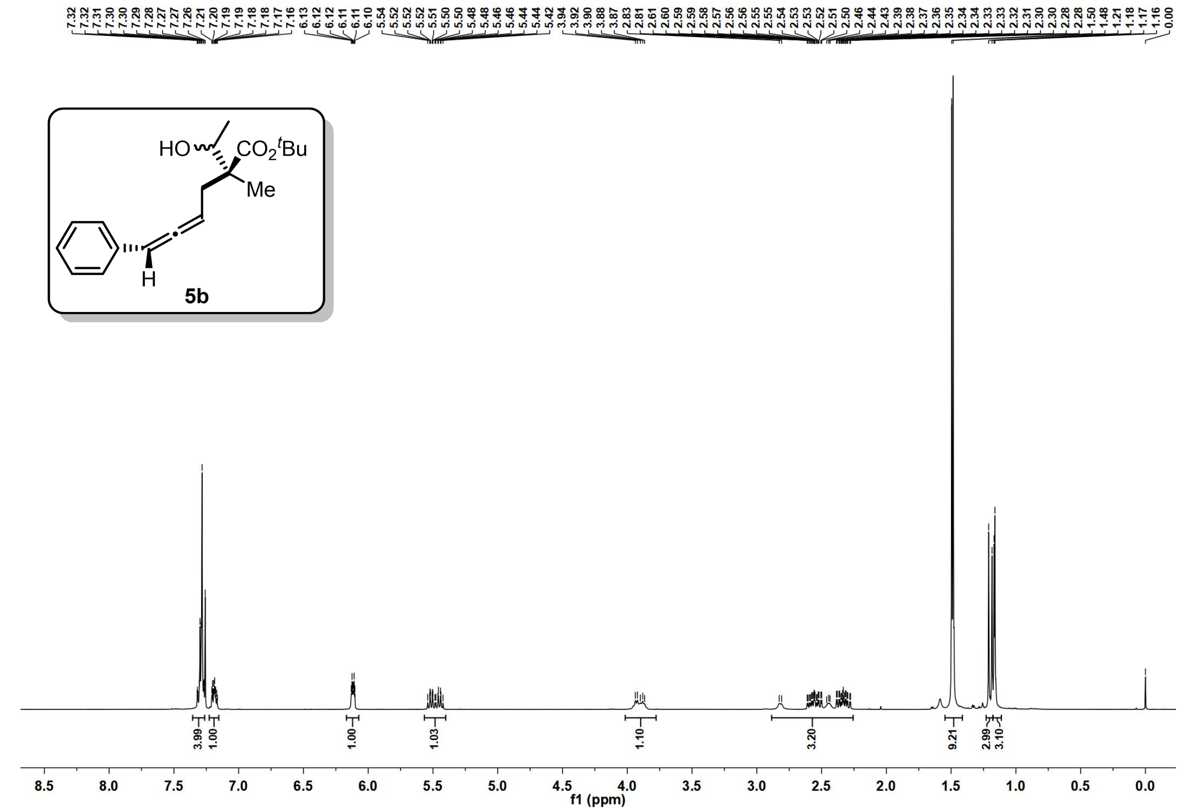

Supplementary Fig. 117. ^{1}H NMR spectra of compound **5b**. ^{1}H NMR (400 MHz, 298K) in $CDCl_3$

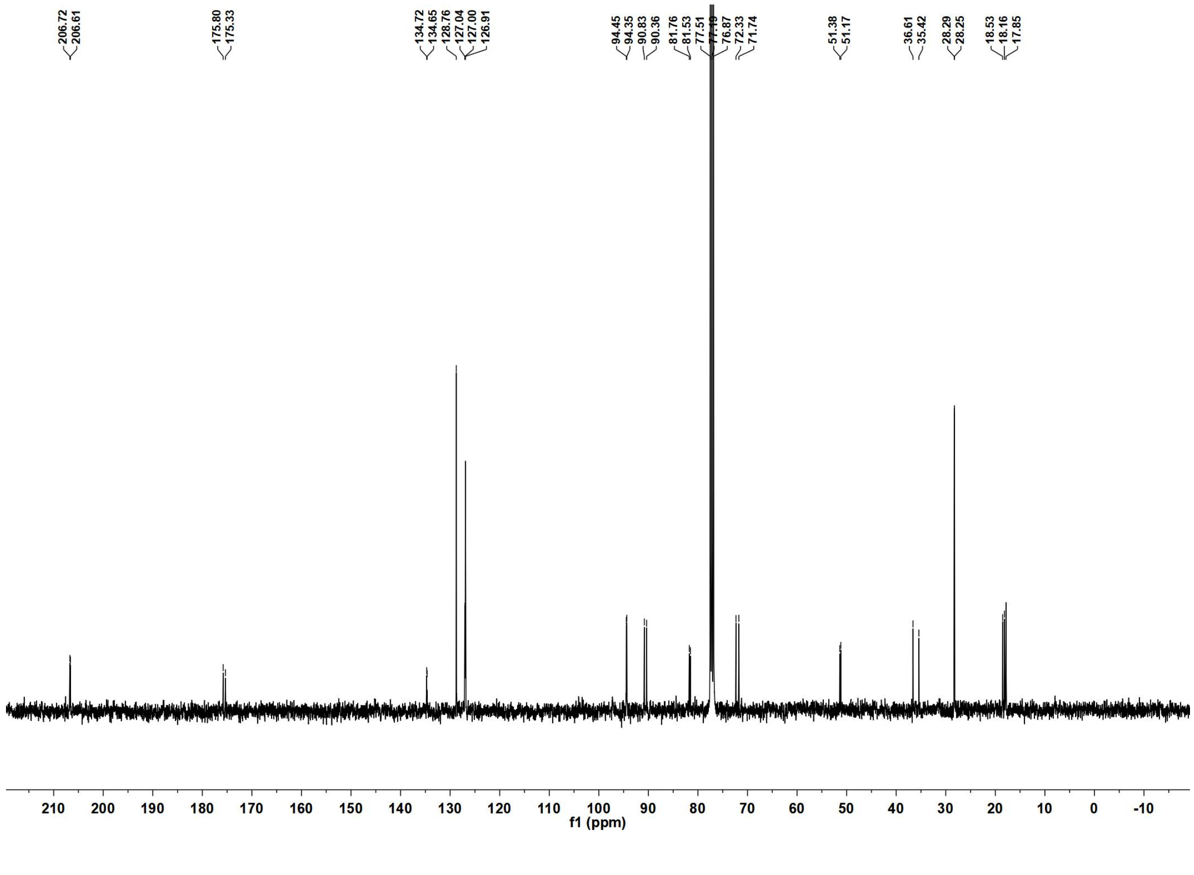

Supplementary Fig. 118. ^{13}C NMR spectra of compound **5b**. ^{13}C NMR (101 MHz, 298K) in $CDCl_3$

4.2 HPLC spectra

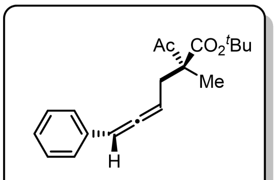

3a

<Chromatogram>

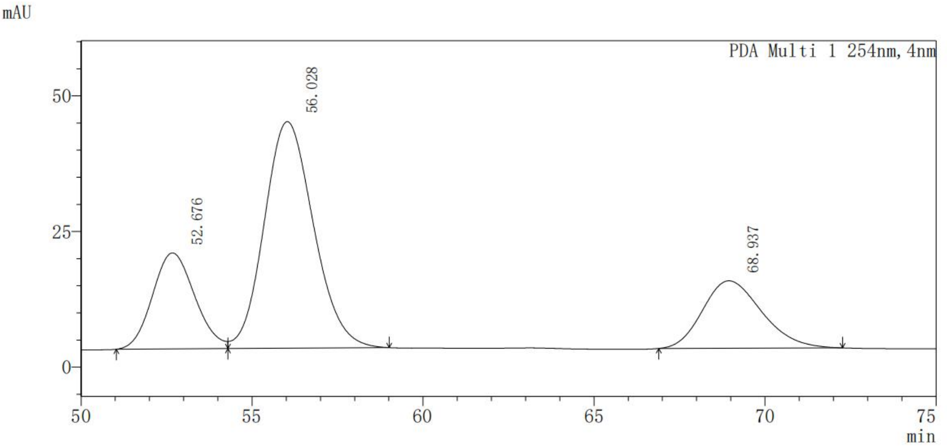

<Peak Results>

PDA Ch1 254nm

Index	Time/min	Height/mAU	Quantity/Area	Area %/%
1	52.676	17707	1522467	21.111
2	56.028	41797	4193261	58.144
3	68.937	12435	1496125	20.745

<Chromatogram>

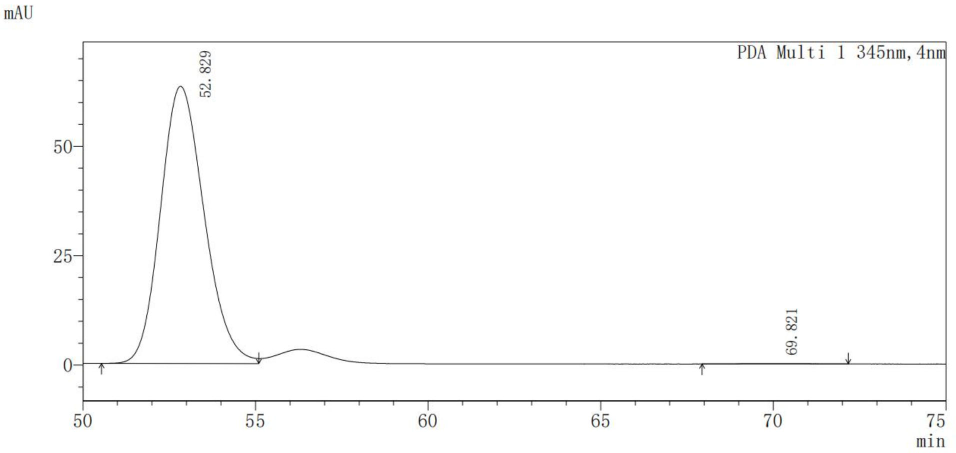

<Peak Results>

PDA Ch1 345nm

Index	Time/min	Height/mAU	Quantity/Area	Area %/%
1	52.829	63333	5578220	99.798
2	69.821	84	11273	0.202

Supplementary Fig. 119. HPLC chromatograms of compound **3a.**

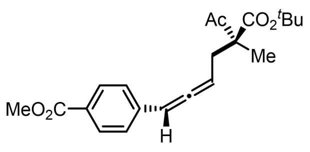

3b

<Chromatogram>

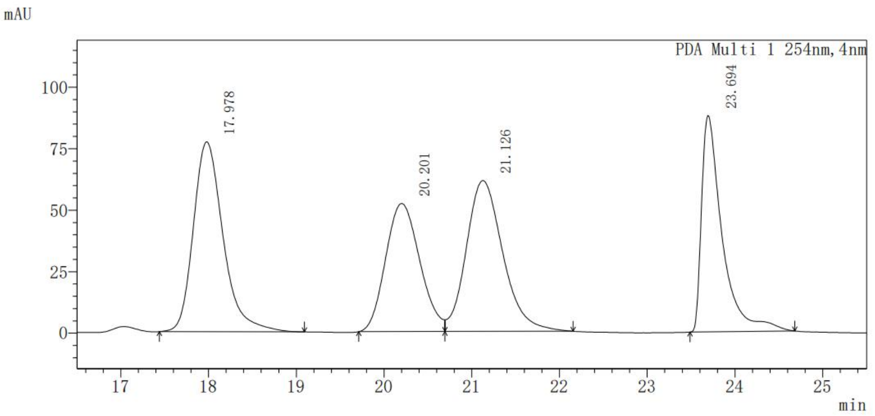

<Peak Results>

PDA Ch1 254nm

Index	Time/min	Height/mAU	Quantity/Area	Area %/%
1	17.978	77177	1821202	28.271
2	20.201	52081	1412845	21.932
3	21.126	61318	1774604	27.547
4	23.694	87983	1433341	22.250

<Chromatogram>

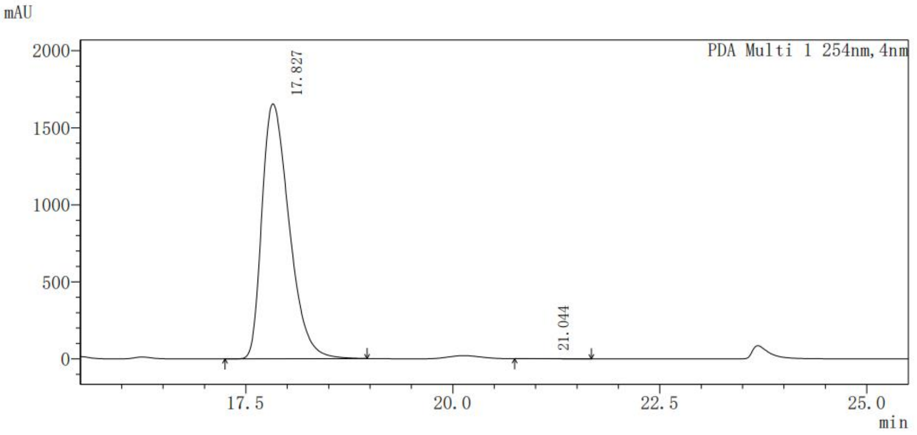

<Peak Results>

PDA Ch1 254nm

Index	Time/min	Height/mAU	Quantity/Area	Area %/%
1	17.827	1654594	37830945	99.940
2	21.044	1069	22540	0.060

Supplementary Fig. 120. HPLC chromatograms of compound **3b.**

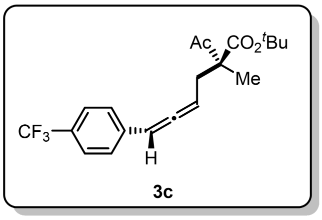

<Chromatogram>

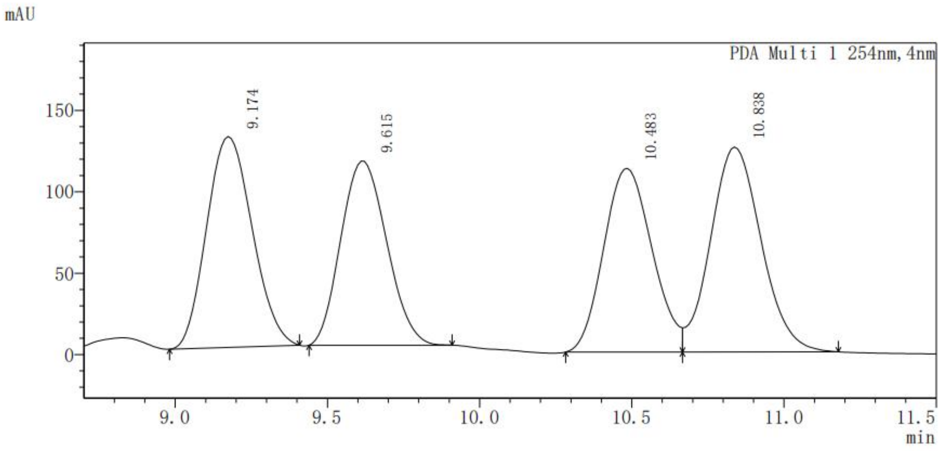

<Peak Results>

PDA Ch1 254nm

Index	Time/min	Height/mAU	Quantity/Area	Area %/%
1	9.174	129548	1352520	25.833
2	9.615	113118	1184527	22.624
3	10.483	112854	1250679	23.888
4	10.838	125870	1447876	27.654

<Chromatogram>

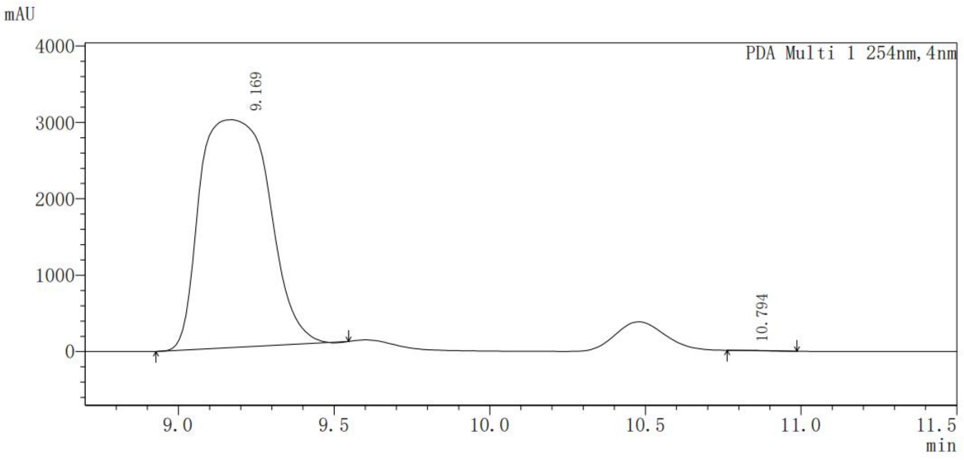

<Peak Results>

PDA Ch1 254nm

Index	Time/min	Height/mAU	Quantity/Area	Area %/%
1	9.169	2982153	46511773	99.957
2	10.794	1848	19929	0.043

Supplementary Fig. 121. HPLC chromatograms of compound **3c.**

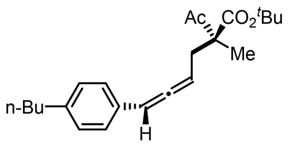

3d

<Chromatogram>

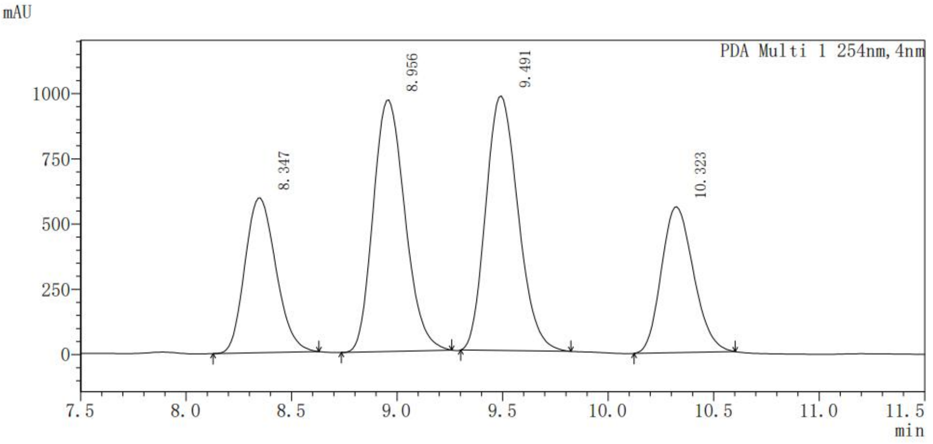

<Peak Results>

PDA Ch1 254nm

Index	Time/min	Height/mAU	Quantity/Area	Area %/%
1	8.347	592621	6077876	18.681
2	8.956	964075	10114784	31.089
3	9.491	975445	10403311	31.976
4	10.323	559177	5938780	18.254

<Chromatogram>

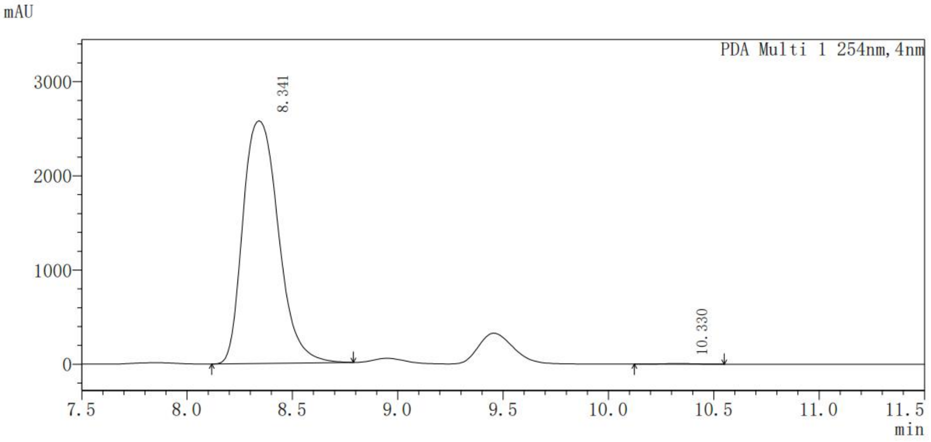

<Peak Results>

PDA Ch1 254nm

Index	Time/min	Height/mAU	Quantity/Area	Area %/%
1	8.341	2577535	30297810	99.775
2	10.330	6589	68388	0.225

Supplementary Fig. 122. HPLC chromatograms of compound **3d.**

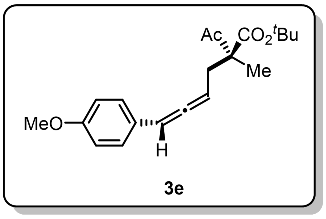

<Chromatogram>

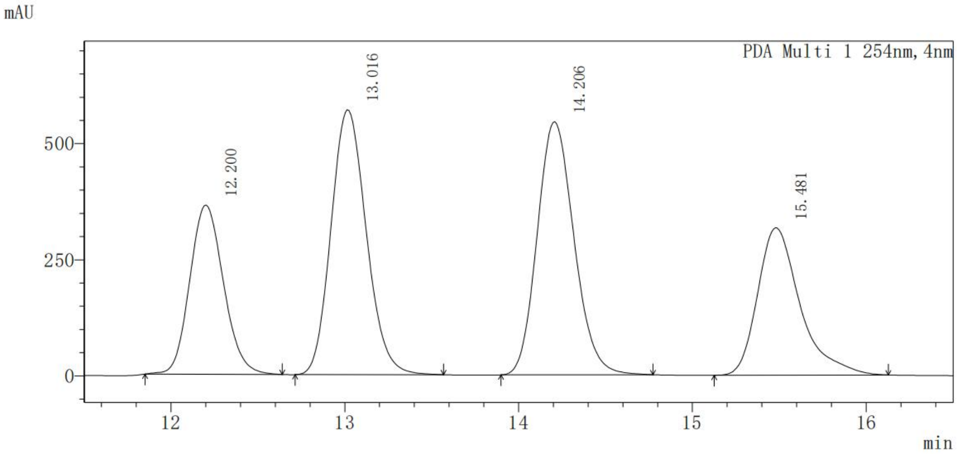

<Peak Results>

PDA Ch1 254nm

Index	Time/min	Height/mAU	Quantity/Area	Area %/%
1	12.200	364161	4918594	18.653
2	13.016	570632	8041790	30.497
3	14.206	545407	8028200	30.445
4	15.481	317292	5380548	20.405

<Chromatogram>

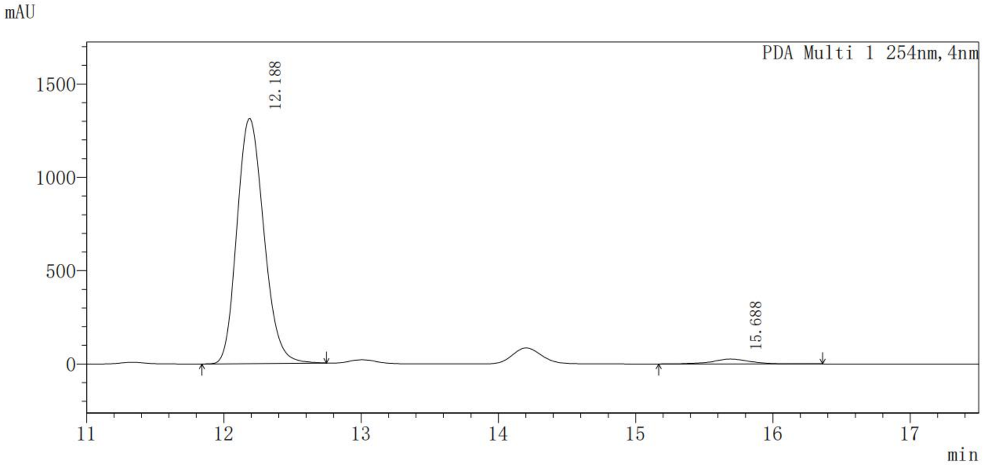

<Peak Results>

PDA Ch1 254nm

Index	Time/min	Height/mAU	Quantity/Area	Area %/%
1	12.188	1313572	17862868	97.307
2	15.688	26300	494451	2.693

Supplementary Fig. 123. HPLC chromatograms of compound **3e.**

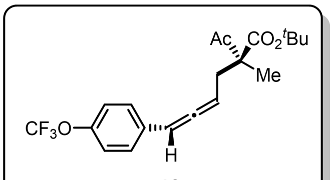

3f

<Chromatogram>

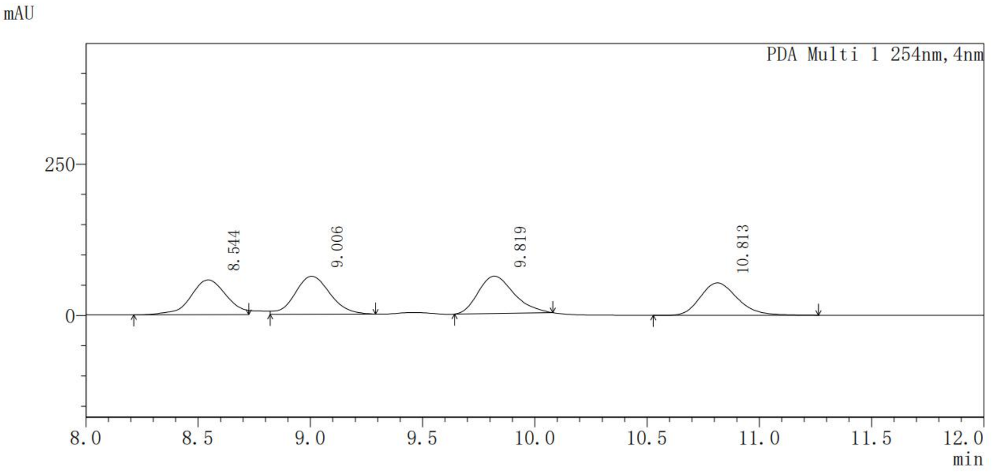

<Peak Results>

PDA Ch1 254nm

Index	Time/min	Height/mAU	Quantity/Area	Area %/%
1	8.544	57568	637815	24.291
2	9.006	62979	686662	26.151
3	9.819	61744	684461	26.068
4	10.813	53547	616774	23.490

<Chromatogram>

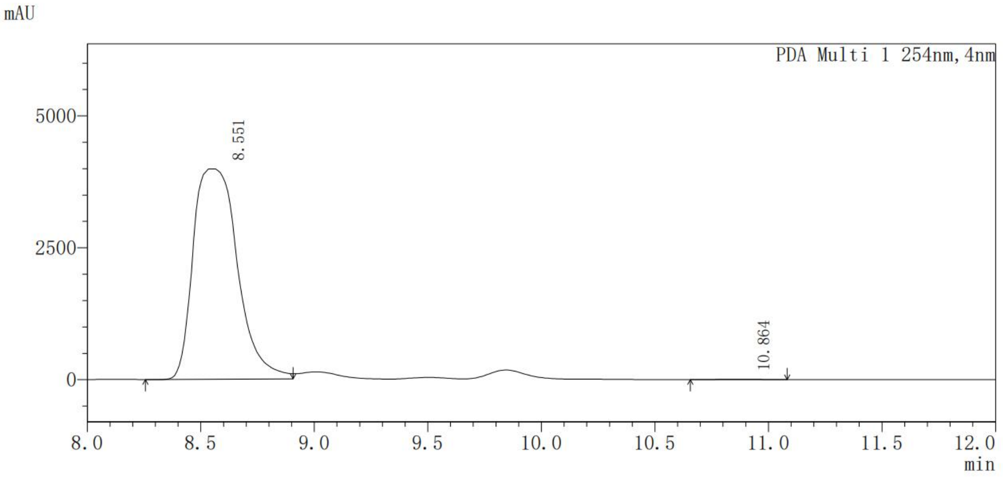

<Peak Results>

PDA Ch1 254nm

Index	Time/min	Height/mAU	Quantity/Area	Area %/%
1	8.551	3987655	54264151	99.853
2	10.864	7330	79931	0.147

Supplementary Fig. 124. HPLC chromatograms of compound **3f.**

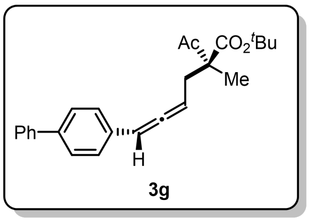

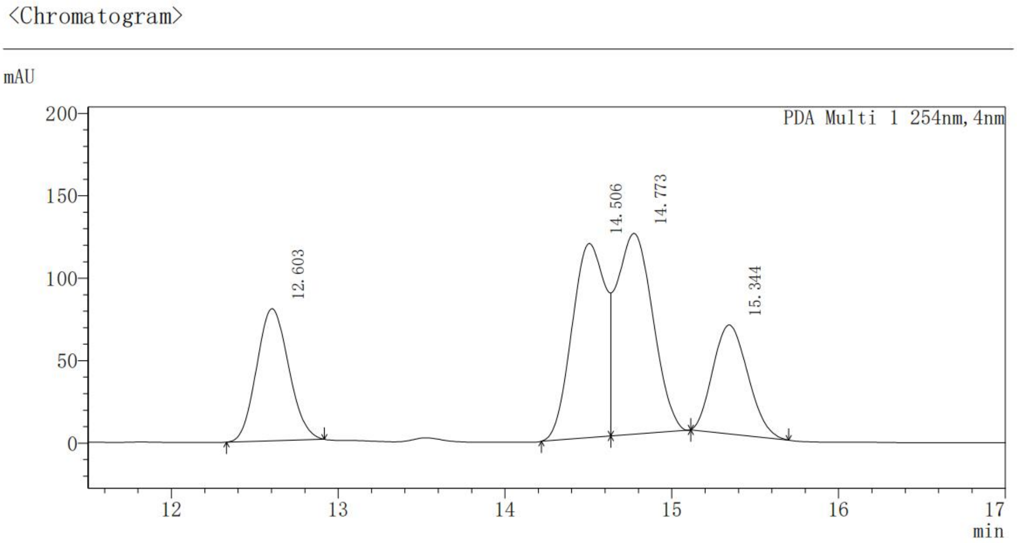

<Peak Results>

PDA Ch1 254nm

Index	Time/min	Height/mAU	Quantity/Area	Area %/%
1	12.603	80060	1072092	19.432
2	14.506	117775	1663169	30.145
3	14.773	121857	1819439	32.978
4	15.344	65973	962440	17.445

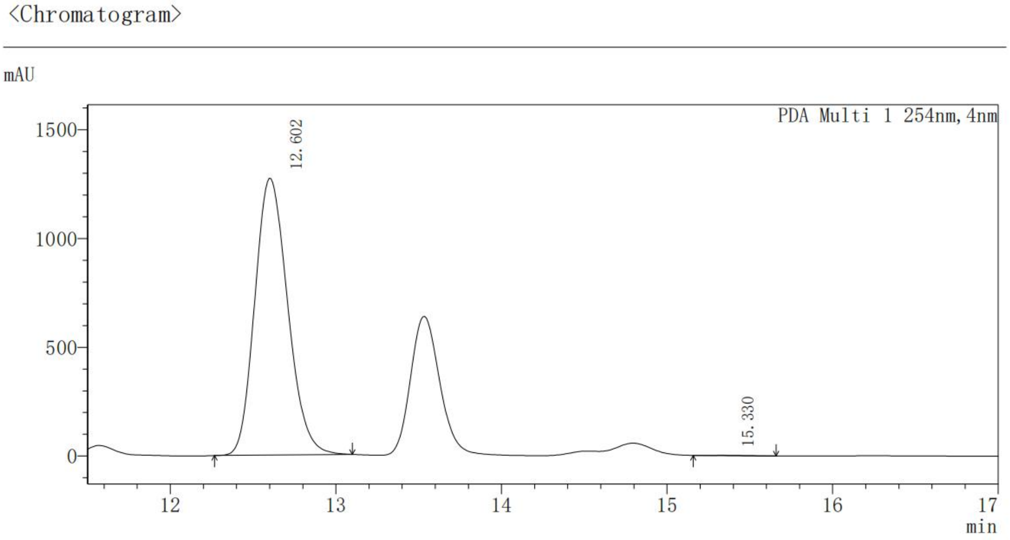

<Peak Results>

PDA Ch1 254nm

Index	Time/min	Height/mAU	Quantity/Area	Area %/%
1	12.602	1272336	17590112	99.927
2	15.330	1295	12890	0.073

Supplementary Fig. 125. HPLC chromatograms of compound **3g.**

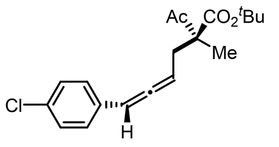

3h

<Chromatogram>

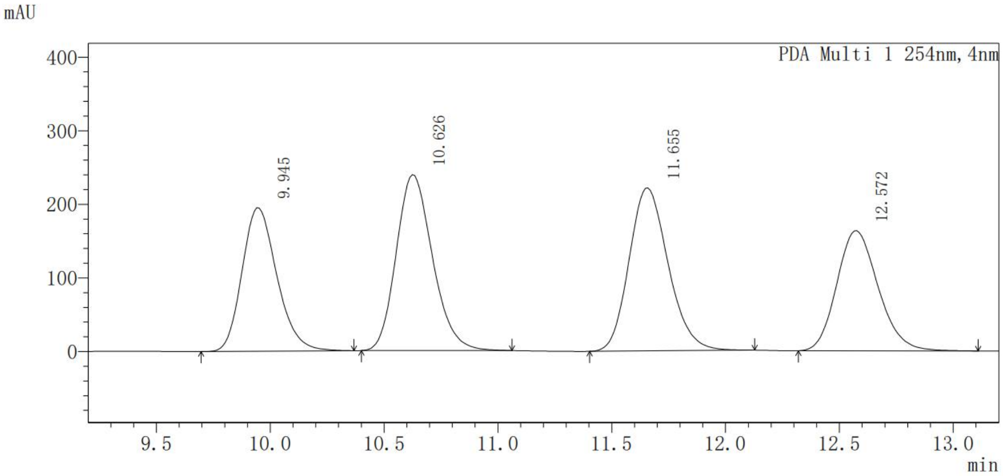

<Peak Results>

PDA Ch1 254nm

Index	Time/min	Height/mAU	Quantity/Area	Area %/%
1	9.945	195035	2077152	22.018
2	10.626	239114	2649189	28.082
3	11.655	221414	2644863	28.036
4	12.572	163205	2062588	21.864

<Chromatogram>

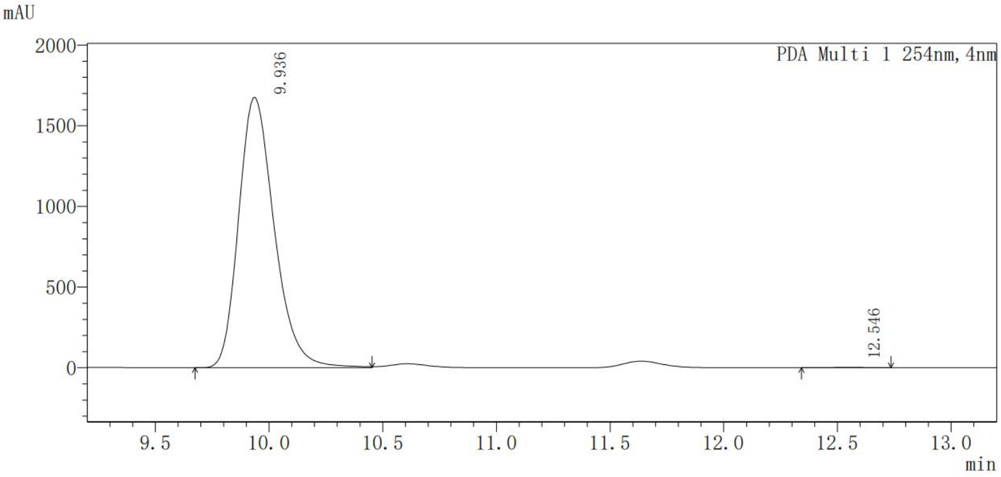

<Peak Results>

PDA Ch1 254nm

Index	Time/min	Height/mAU	Quantity/Area	Area %/%
1	9.936	1675510	18307158	99.894
2	12.546	1722	19391	0.106

Supplementary Fig. 126. HPLC chromatograms of compound **3h.**

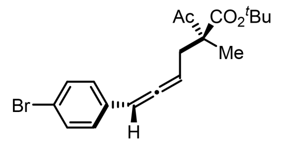

3i

<Chromatogram>

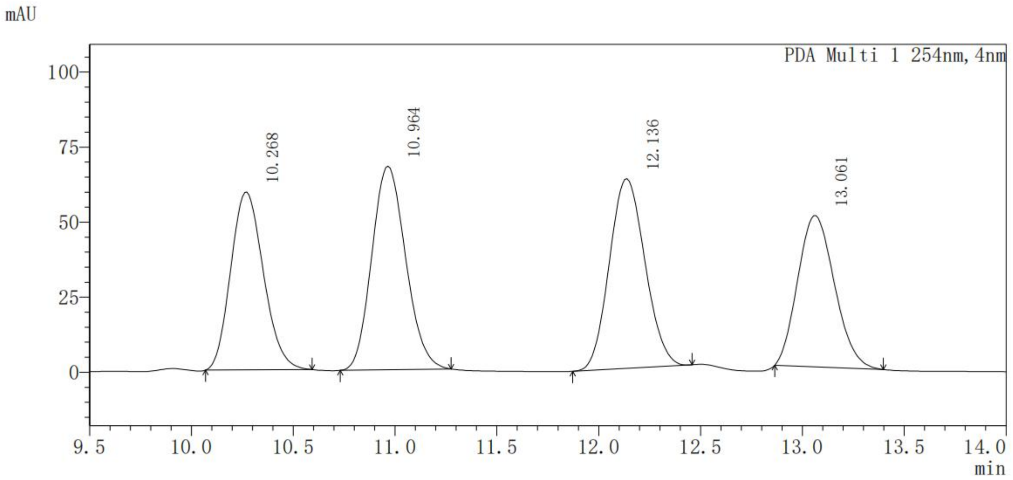

<Peak Results>

PDA Ch1 254nm

Index	Time/min	Height/mAU	Quantity/Area	Area %/%
1	10.268	59299	663728	23.269
2	10.964	67866	786599	27.576
3	12.136	63238	770162	27.000
4	13.061	50352	631947	22.155

<Chromatogram>

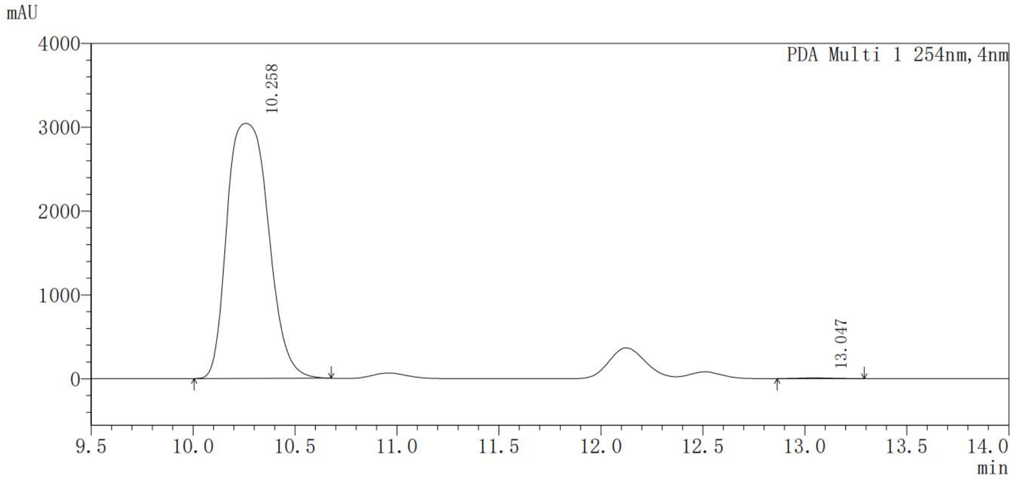

<Peak Results>

PDA Ch1 254nm

Index	Time/min	Height/mAU	Quantity/Area	Area %/%
1	10.258	3044358	43055483	99.759
2	13.047	8687	104177	0.241

Supplementary Fig. 127. HPLC chromatograms of compound **3i.**

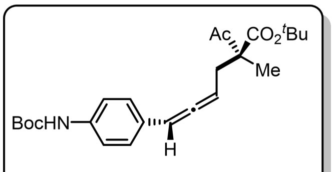

3j

<Chromatogram>

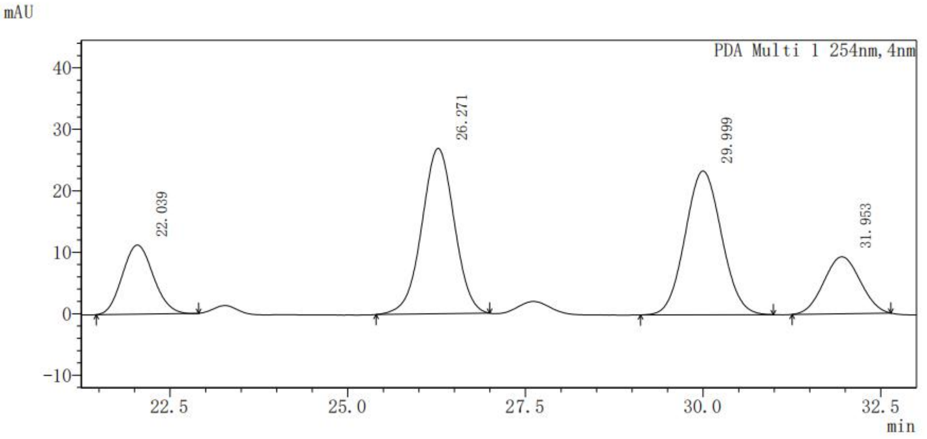

<Peak Results>

PDA Ch1 254nm

Index	Time/min	Height/mAU	Quantity/Area	Area %/%
1	22.039	11242	341095	14.424
2	26.271	26927	856942	36.239
3	29.999	23397	835855	35.347
4	31.953	9265	330817	13.990

<Chromatogram>

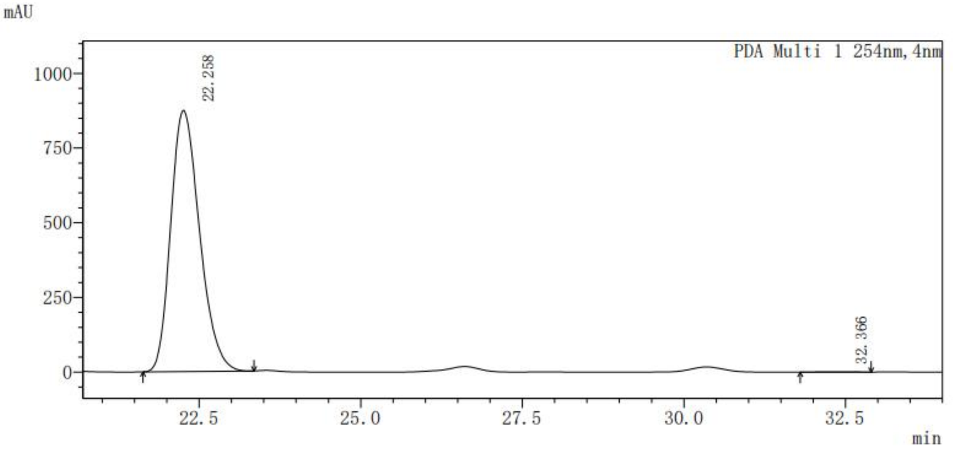

<Peak Results>

PDA Ch1 254nm

Index	Time/min	Height/mAU	Quantity/Area	Area %/%
1	22.258	874570	27132728	99.910
2	32.366	789	24399	0.090

Supplementary Fig. 128. HPLC chromatograms of compound **3j.**

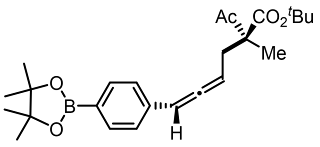

3k

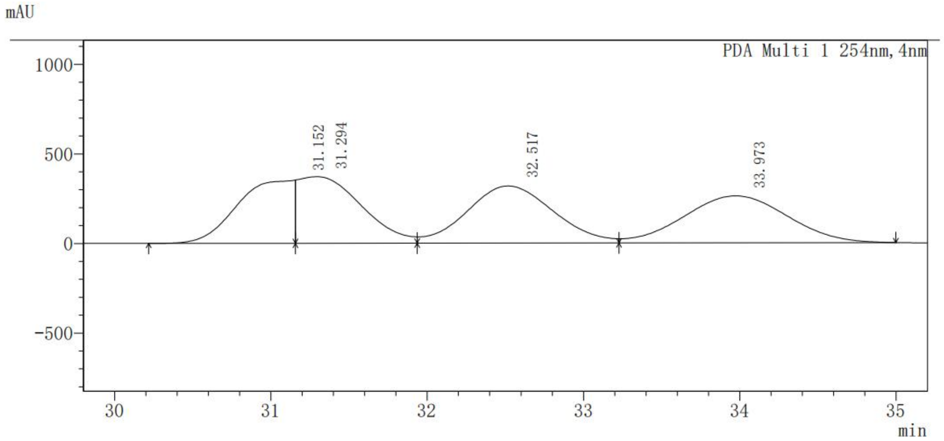

<Peak Results>

PDA Ch1 254nm

Index	Time/min	Height/mAU	Quantity/Area	Area %/%
1	31.152	351384	8829443	20.340
2	31.294	371348	10260413	23.636
3	32.517	318375	12247132	28.213
4	33.973	261755	12072988	27.812

<Chromatogram>

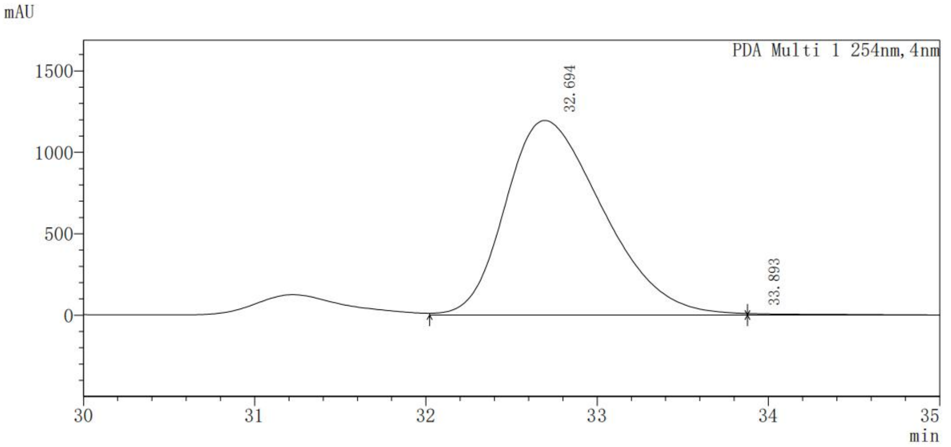

<Peak Results>

PDA Ch1 254nm

Index	Time/min	Height/mAU	Quantity/Area	Area %/%
1	32.694	1194481	47398552	99.481
2	33.893	8918	247308	0.519

Supplementary Fig. 129. HPLC chromatograms of compound **3k.**

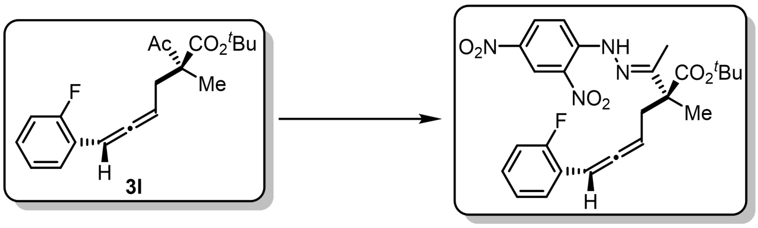

<Chromatogram>

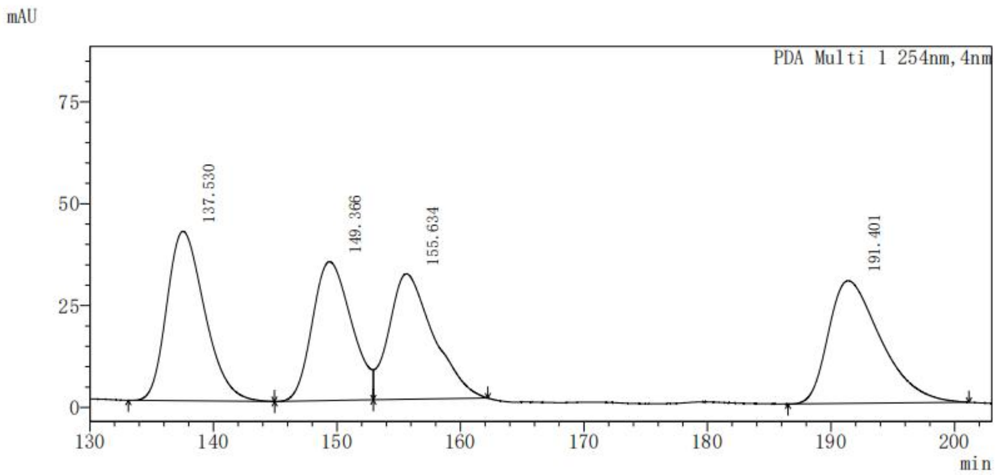

<Peak Results>

PDA Ch1 254nm

Index	Time/min	Height/mAU	Quantity/Area	Area %/%
1	137.530	41605	8853756	26.848
2	149.366	34085	7503264	22.753
3	155.634	30830	7731627	23.446
4	191.401	30122	8888120	26.953

<Chromatogram>

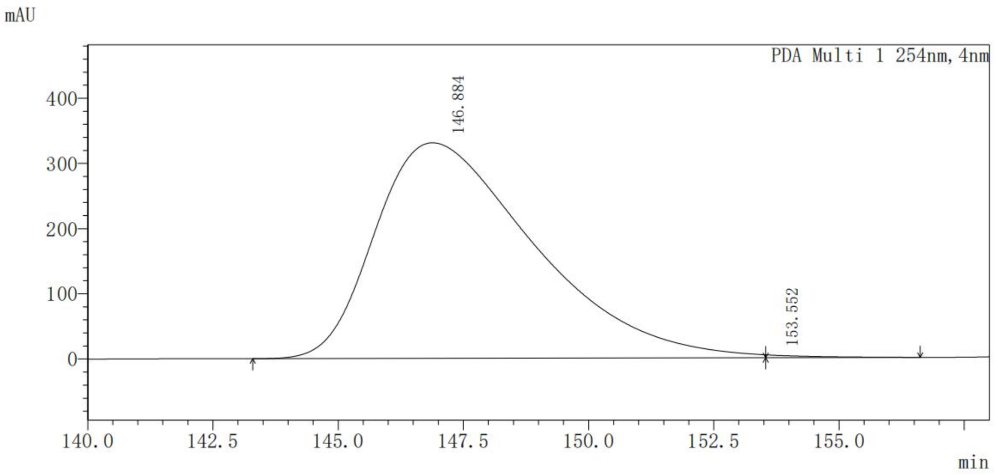

<Peak Results>

PDA Ch1 254nm

Index	Time/min	Height/mAU	Quantity/Area	Area %/%
1	146.884	330704	73435289	99.662
2	153.552	4331	248898	0.338

Supplementary Fig. 130. HPLC chromatograms of compound **3l.**

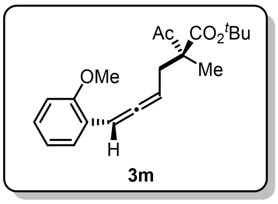

3m

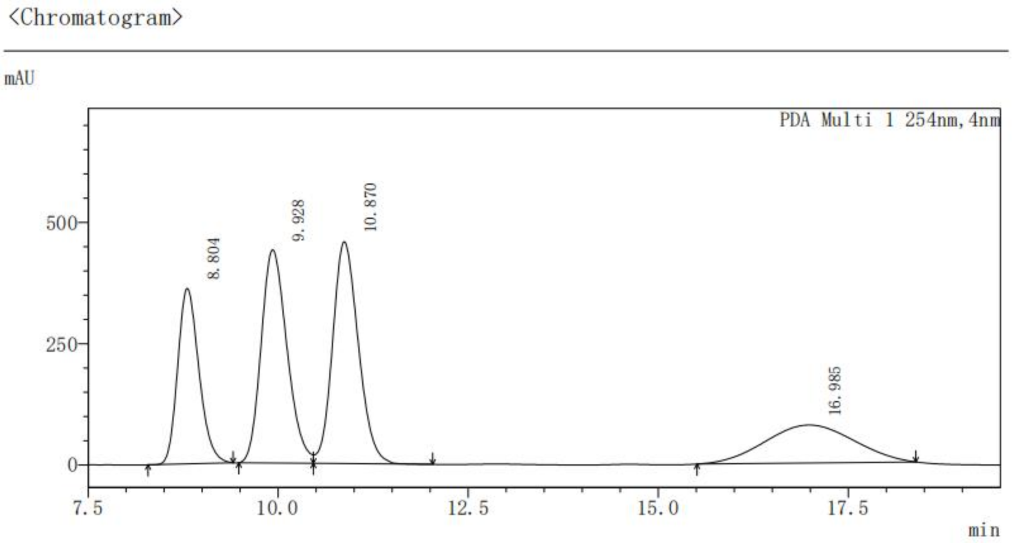

<Peak Results>

PDA Ch1 254nm

Index	Time/min	Height/mAU	Quantity/Area	Area %/%
1	8.804	361619	7040376	20.373
2	9.928	439271	10394567	30.079
3	10.870	457349	10771663	31.170
4	16.985	78222	6351472	18.379

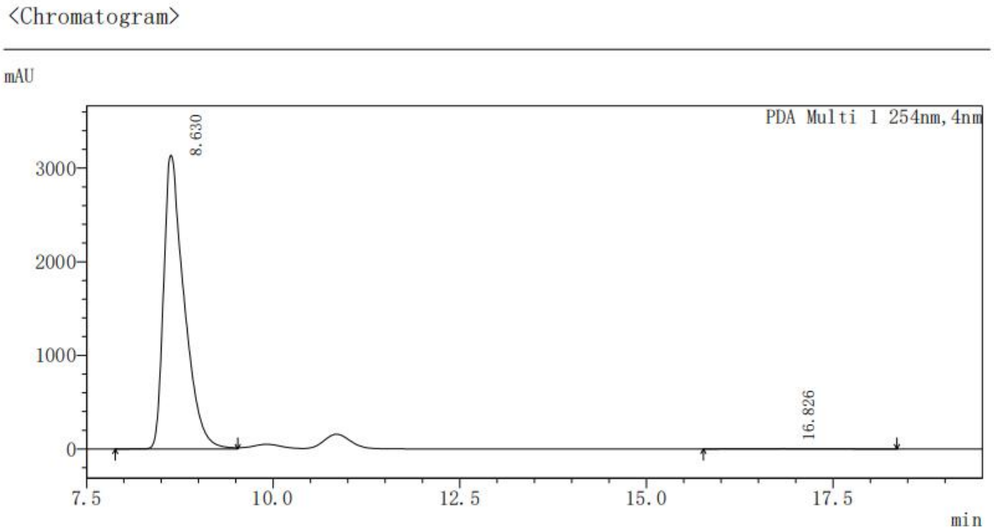

<Peak Results>

PDA Ch1 254nm

Index	Time/min	Height/mAU	Quantity/Area	Area %/%
1	8.630	3136468	60110107	99.631
2	16.826	3152	222770	0.369

Supplementary Fig. 131. HPLC chromatograms of compound **3m.**

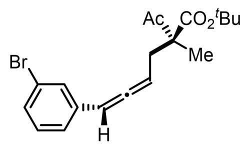

3n

<Chromatogram>

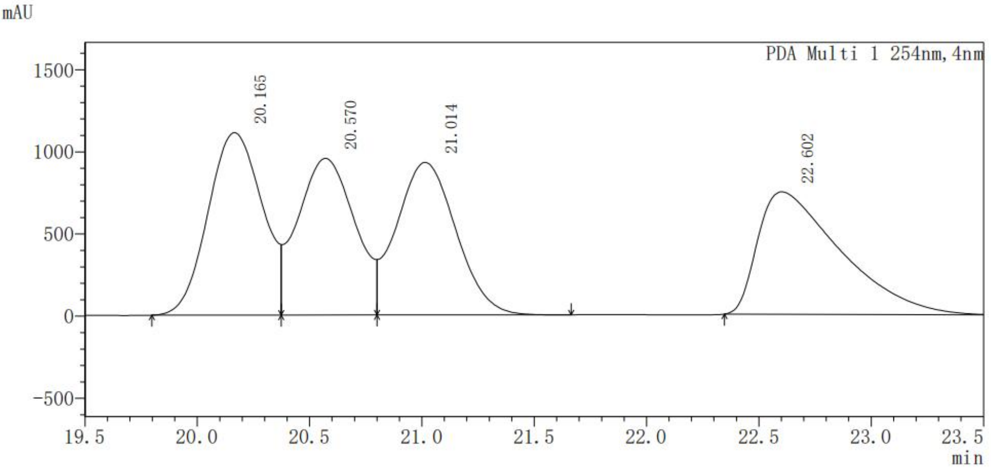

<Peak Results>

PDA Ch1 254nm

Index	Time/min	Height/mAU	Quantity/Area	Area %/%
1	20.165	1109931	18756868	26.137
2	20.570	952615	16902155	23.553
3	21.014	929020	17025009	23.724
4	22.602	744926	19078359	26.585

<Chromatogram>

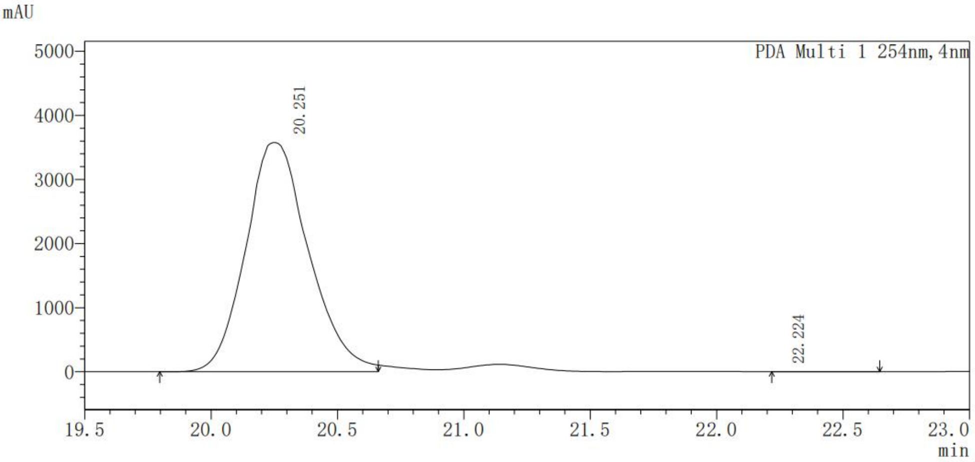

<Peak Results>

PDA Ch1 254nm

Index	Time/min	Height/mAU	Quantity/Area	Area %/%
1	20.251	3576177	61992253	99.964
2	22.224	1759	22554	0.036

Supplementary Fig. 132. HPLC chromatograms of compound **3n.**

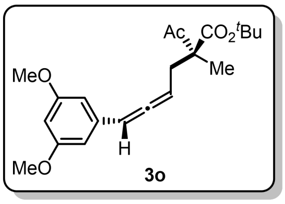

<Chromatogram>

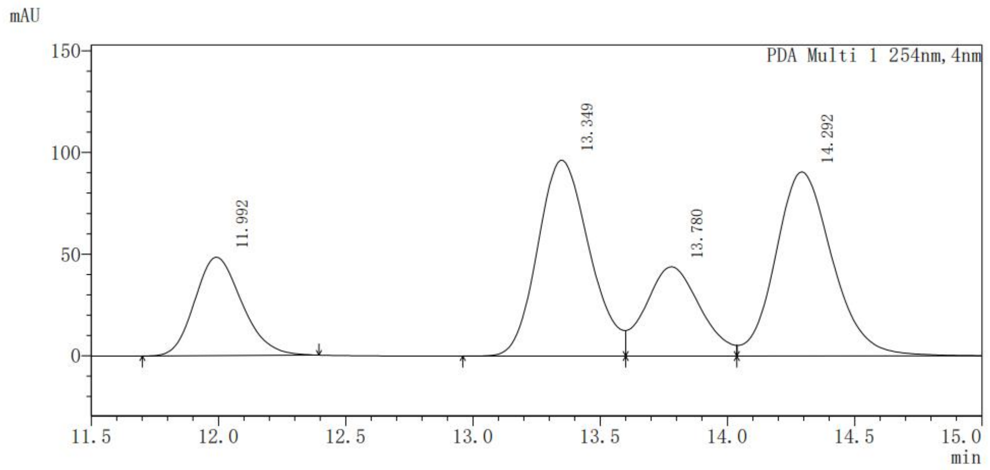

<Peak Results>

PDA Ch1 254nm

Index	Time/min	Height/mAU	Quantity/Area	Area %/%
1	11.992	48412	625046	15.468
2	13.349	96347	1364648	33.771
3	13.780	43944	648543	16.049
4	14.292	90585	1402678	34.712

<Chromatogram>

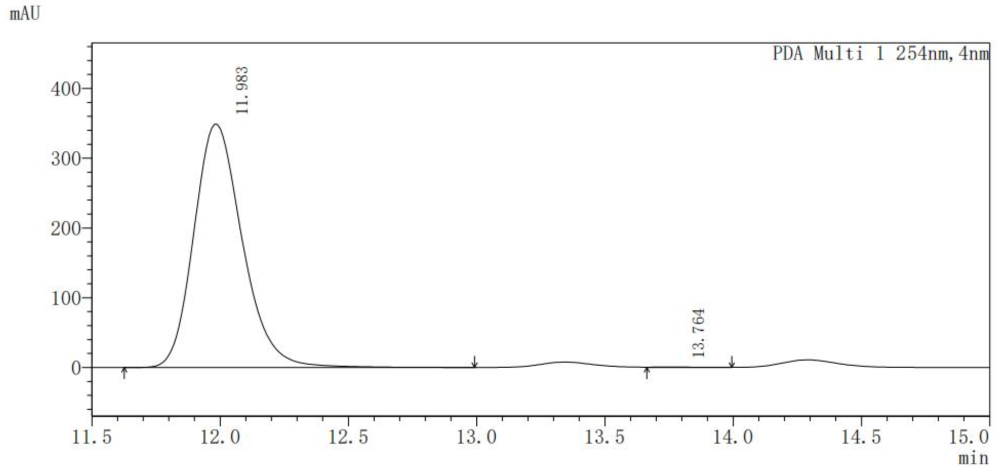

<Peak Results>

PDA Ch1 254nm

Index	Time/min	Height/mAU	Quantity/Area	Area %/%
1	11.983	348866	4584453	99.856
2	13.764	515	6626	0.144

Supplementary Fig. 133. HPLC chromatograms of compound **3o.**

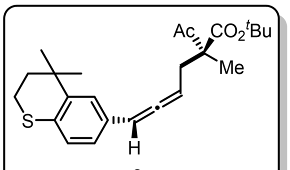

3p

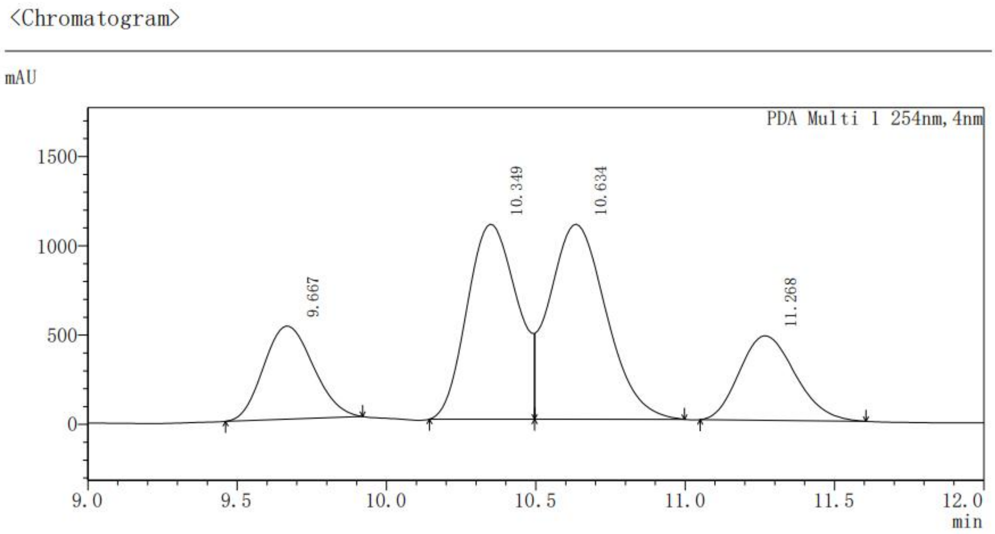

<Peak Results>

PDA Ch1 254nm

Index	Time/min	Height/mAU	Quantity/Area	Area %/%
1	9.667	521471	6035441	15.288
2	10.349	1091672	12935009	32.765
3	10.634	1091766	14361196	36.378
4	11.268	473135	6146473	15.569

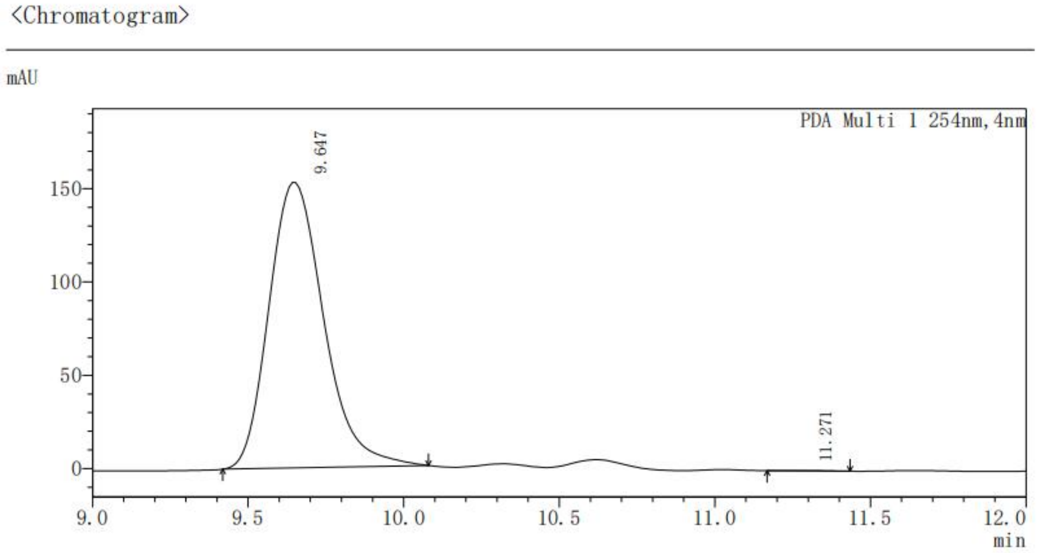

<Peak Results>

PDA Ch1 254nm

Index	Time/min	Height/mAU	Quantity/Area	Area %/%
1	9.647	153011	1873953	99.914
2	11.271	183	1621	0.086

Supplementary Fig. 134. HPLC chromatograms of compound **3p.**

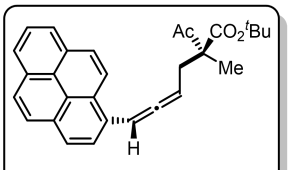

3q

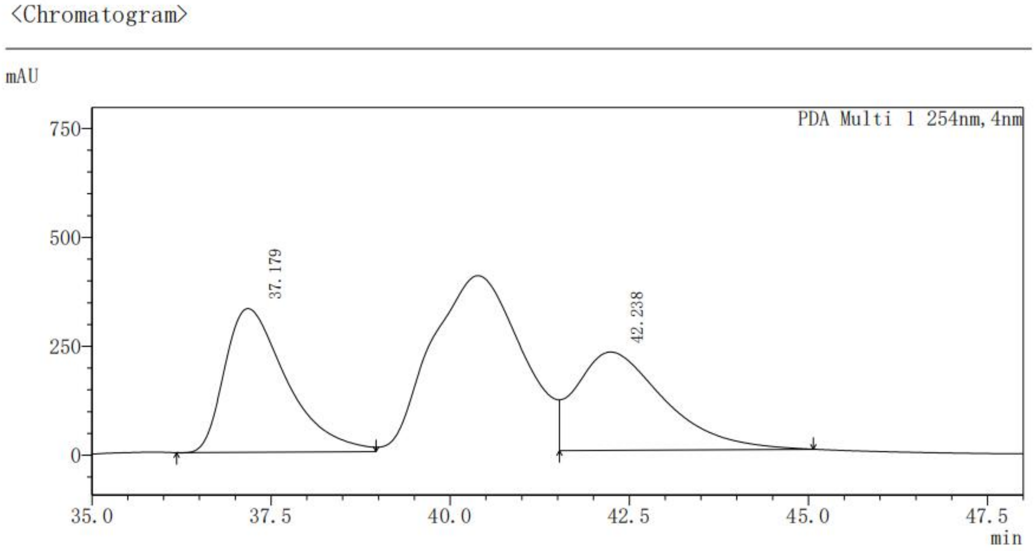

<Peak Results>

PDA Ch1 254nm

Index	Time/min	Height/mAU	Quantity/Area	Area %/%
1	37.179	329994	19873794	50.597
2	42.238	225650	19404695	49.403

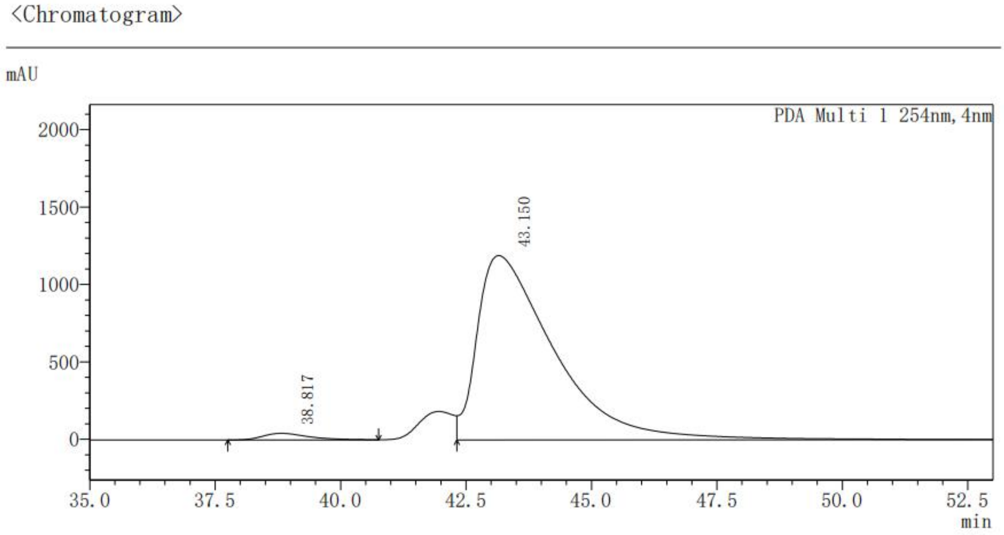

<Peak Results>

PDA Ch1 254nm

Index	Time/min	Height/mAU	Quantity/Area	Area %/%
1	38.817	42921	2746657	2.110
2	43.150	1190317	127416024	97.890

Supplementary Fig. 135. HPLC chromatograms of compound **3q.**

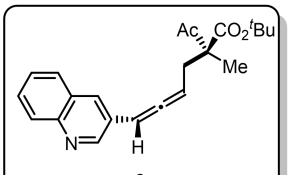

3r

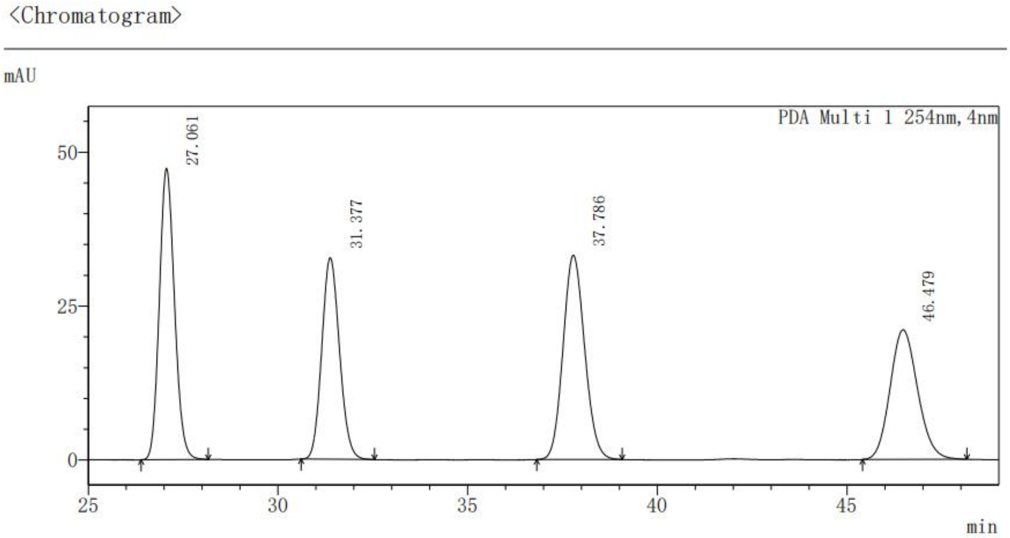

<Peak Results>

PDA Ch1 254nm

Index	Time/min	Height/mAU	Quantity/Area	Area %/%
1	27.061	47303	1312887	27.621
2	31.377	32738	1063480	22.374
3	37.786	33178	1314210	27.648
4	46.479	21068	1062720	22.358

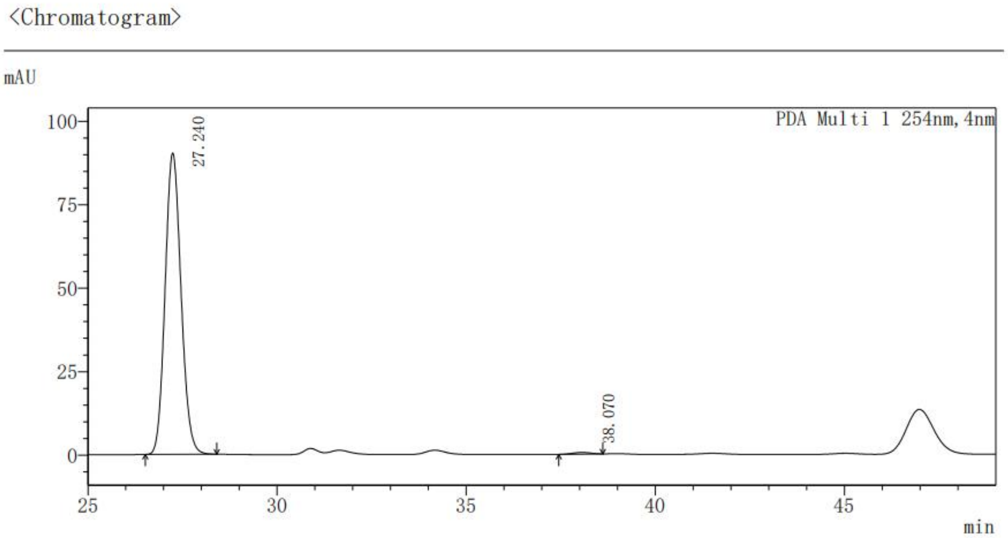

<Peak Results>

PDA Ch1 254nm

Index	Time/min	Height/mAU	Quantity/Area	Area %/%
1	27.240	90289	2640185	99.377
2	38.070	492	16556	0.623

Supplementary Fig. 136. HPLC chromatograms of compound **3r.**

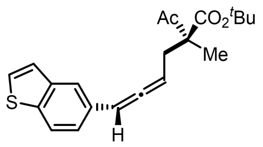

3s

<Chromatogram>

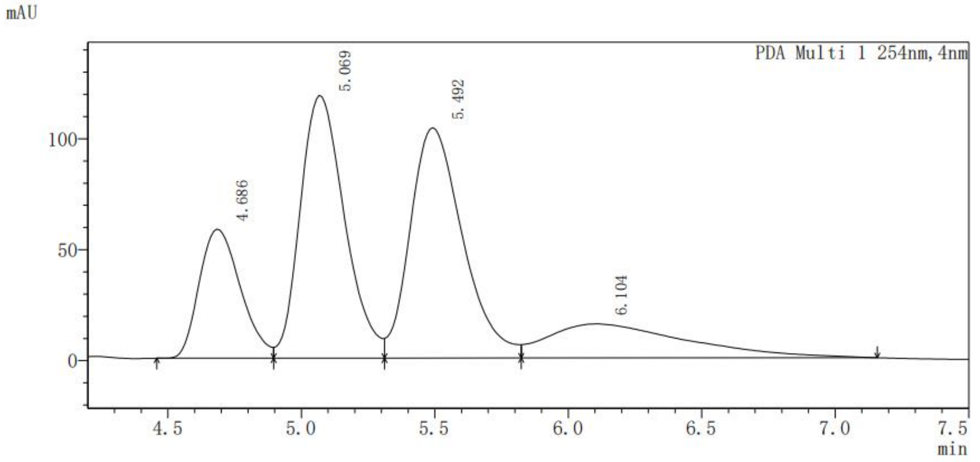

<Peak Results>

PDA Ch1 254nm

Index	Time/min	Height/mAU	Quantity/Area	Area %/%
1	4.686	58143	626070	15.798
2	5.069	118377	1366656	34.487
3	5.492	103785	1416674	35.749
4	6.104	15322	553458	13.966

<Chromatogram>

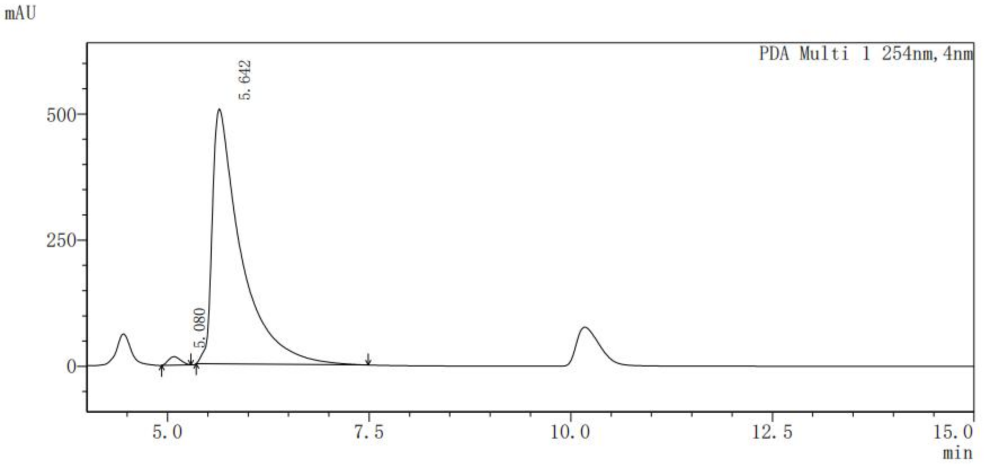

<Peak Results>

PDA Ch1 254nm

Index	Time/min	Height/mAU	Quantity/Area	Area %/%
1	5.080	16889	178083	1.398
2	5.642	504748	12560033	98.602

Supplementary Fig. 137. HPLC chromatograms of compound **3s.**

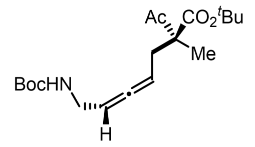

3t

<Chromatogram>

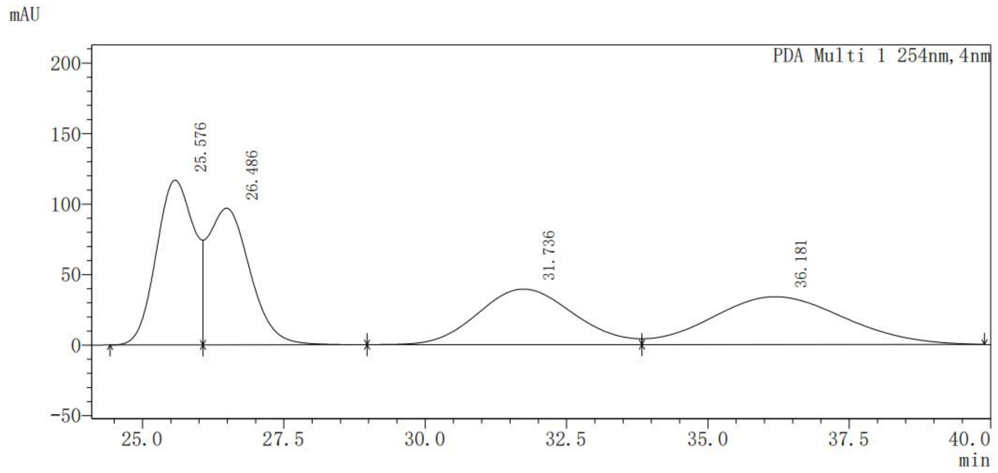

<Peak Results>

PDA Ch1 254nm

Index	Time/min	Height/mAU	Quantity/Area	Area %/%
1	25.576	116792	5504386	26.621
2	26.486	96846	4972384	24.048
3	31.736	39347	4655661	22.516
4	36.181	33899	5544288	26.814

<Chromatogram>

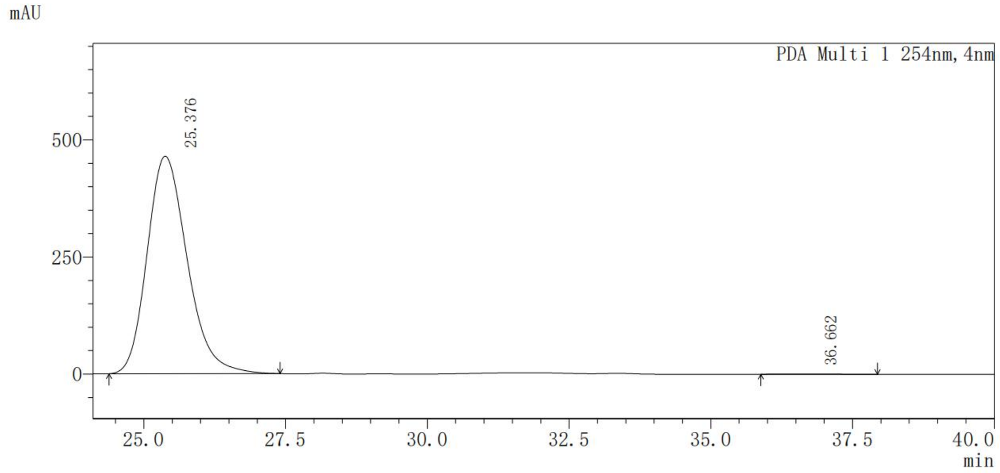

<Peak Results>

PDA Ch1 254nm

Index	Time/min	Height/mAU	Quantity/Area	Area %/%
1	25.376	464509	22864785	99.916
2	36.662	286	19266	0.084

Supplementary Fig. 138. HPLC chromatograms of compound **3t.**

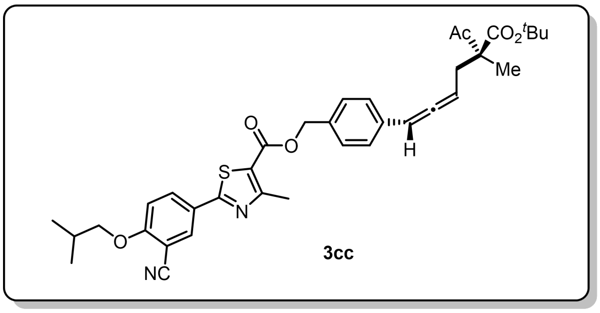

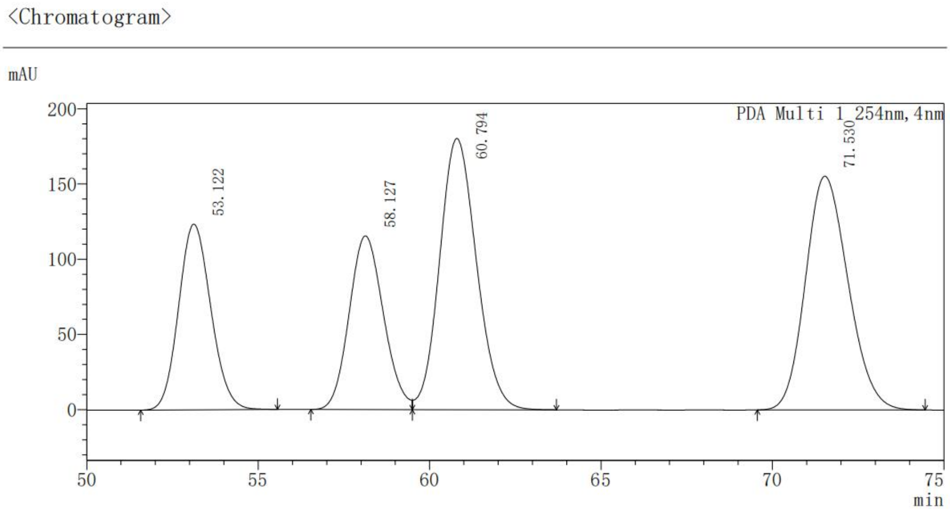

<Peak Results>

PDA Ch1 254nm

Index	Time/min	Height/mAU	Quantity/Area	Area %/%
1	53.122	123431	8083016	18.778
2	58.127	115414	8019983	18.631
3	60.794	180248	13491834	31.343
4	71.530	155342	13450658	31.248

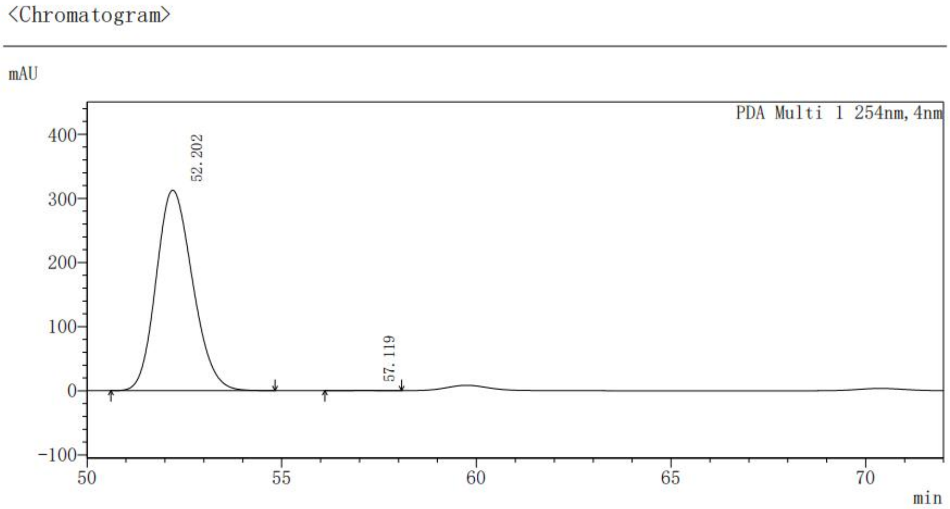

<Peak Results>

PDA Ch1 254nm

Index	Time/min	Height/mAU	Quantity/Area	Area %/%
1	52.202	312724	20389145	99.880
2	57.119	366	24476	0.120

Supplementary Fig. 139. HPLC chromatograms of compound **3cc.**

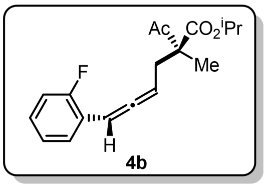

<Chromatogram>

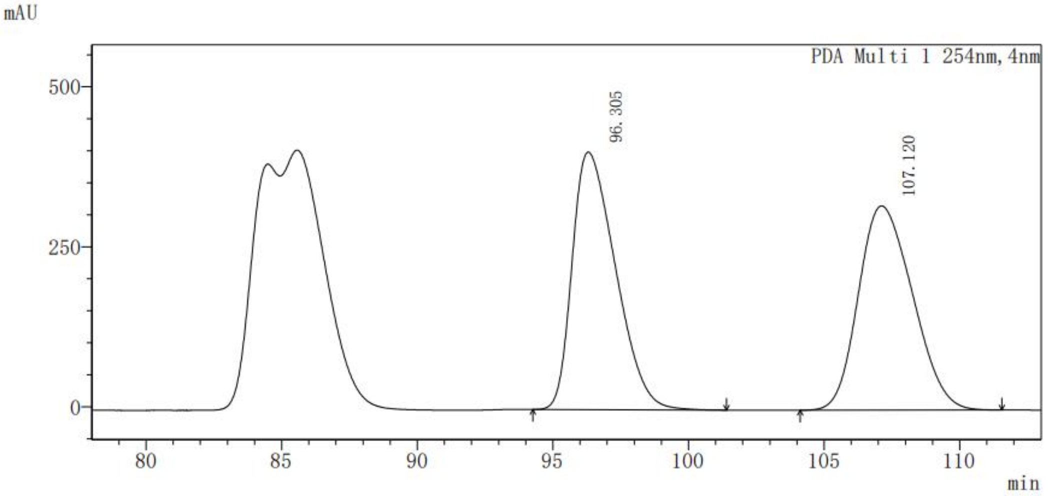

<Peak Results>

PDA Ch1 254nm

Index	Time/min	Height/mAU	Quantity/Area	Area %/%
1	96.305	402482	44764492	50.248
2	107.120	319120	44323144	49.752

<Chromatogram>

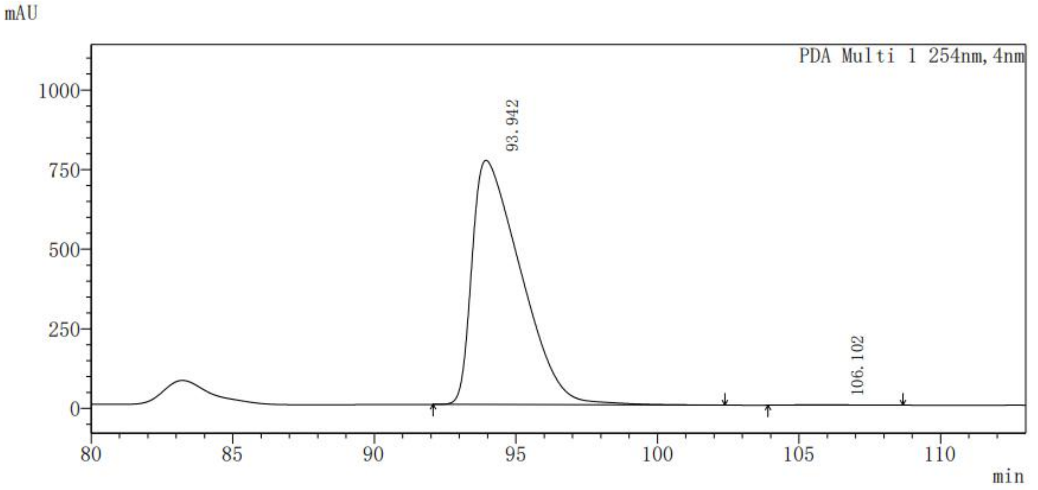

<Peak Results>

PDA Ch1 254nm

Index	Time/min	Height/mAU	Quantity/Area	Area %/%
1	93.942	767050	91760621	99.818
2	106.102	1297	166963	0.182

Supplementary Fig. 140. HPLC chromatograms of compound **4b.**

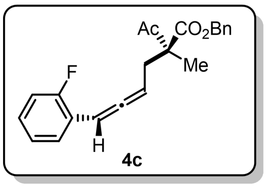

<Chromatogram>

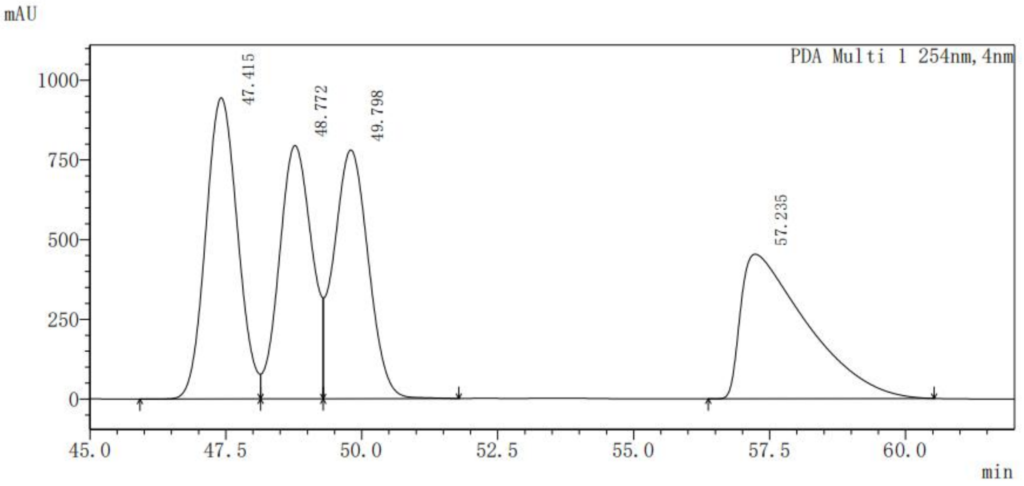

<Peak Results>

PDA Ch1 254nm

Index	Time/min	Height/mAU	Quantity/Area	Area %/%
1	47.415	944927	38441114	26.652
2	48.772	794876	33023311	22.896
3	49.798	780713	34400591	23.851
4	57.235	453657	38365834	26.600

<Chromatogram>

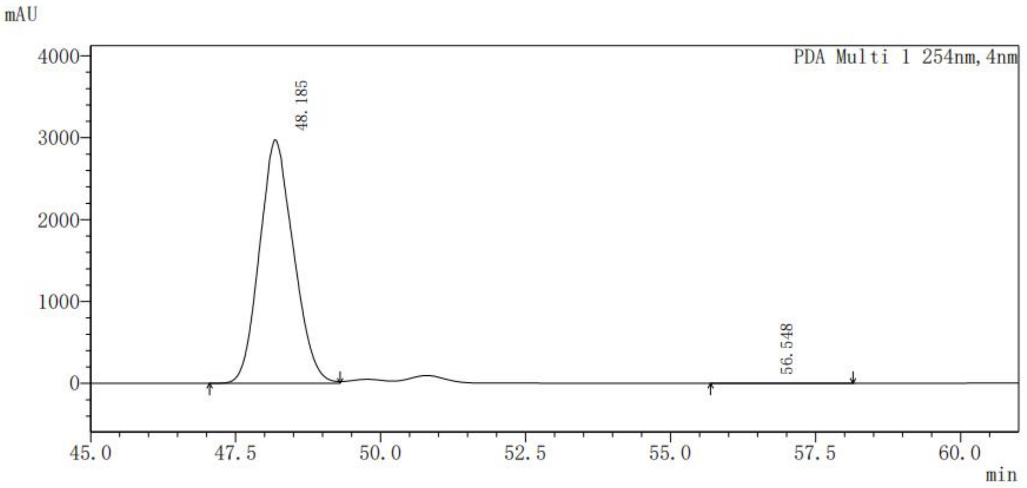

<Peak Results>

PDA Ch1 254nm

Index	Time/min	Height/mAU	Quantity/Area	Area %/%
1	48.185	2972532	121379664	99.975
2	56.548	552	30210	0.025

Supplementary Fig. 141. HPLC chromatograms of compound **4c.**

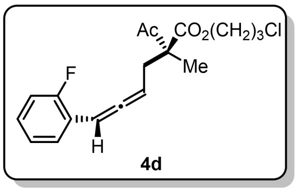

<Chromatogram>

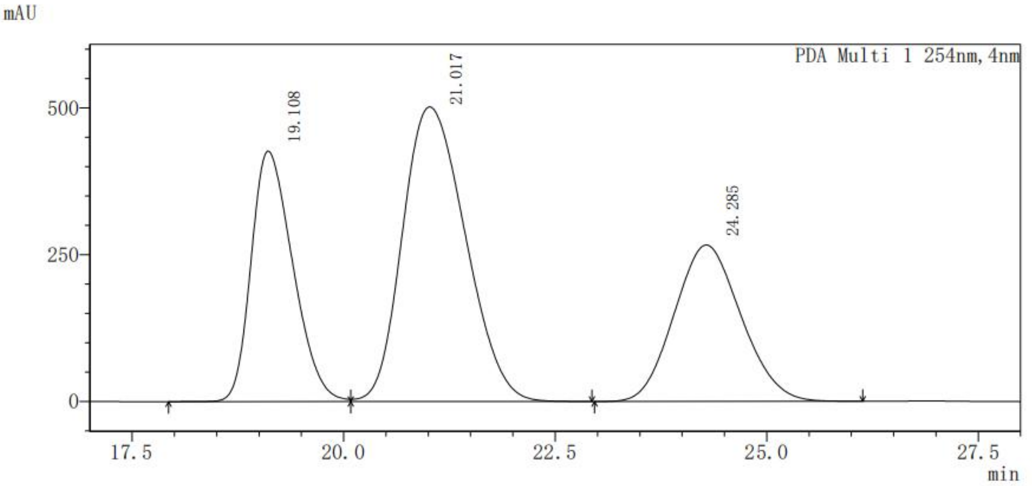

<Peak Results>

PDA Ch1 254nm

Index	Time/min	Height/mAU	Quantity/Area	Area %/%
1	19.108	426412	14633168	26.514
2	21.017	501550	25958037	47.033
3	24.285	266273	14600136	26.454

<Chromatogram>

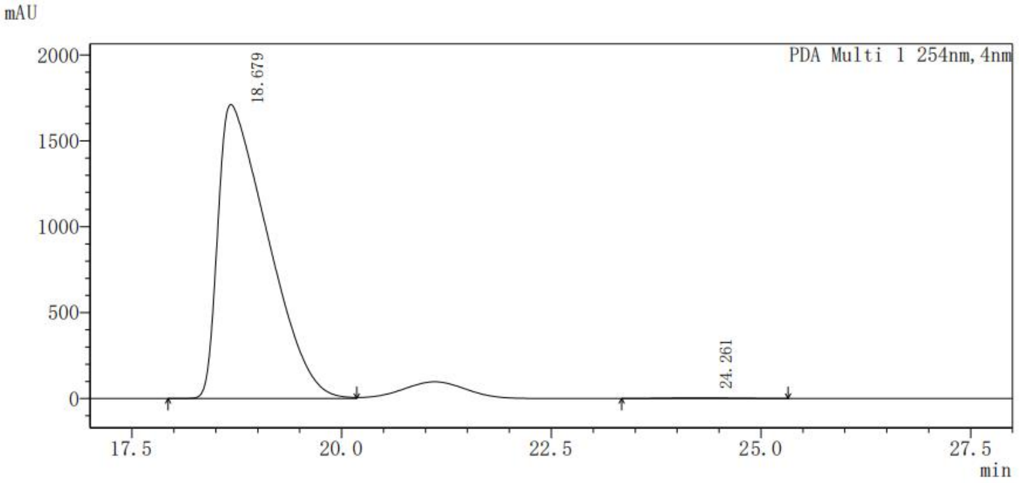

<Peak Results>

PDA Ch1 254nm

Index	Time/min	Height/mAU	Quantity/Area	Area %/%
1	18.679	1712369	70799013	99.745
2	24.261	3362	181206	0.255

Supplementary Fig. 142. HPLC chromatograms of compound **4d.**

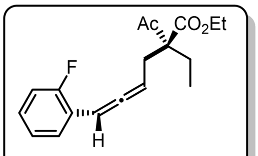

4e

<Chromatogram>

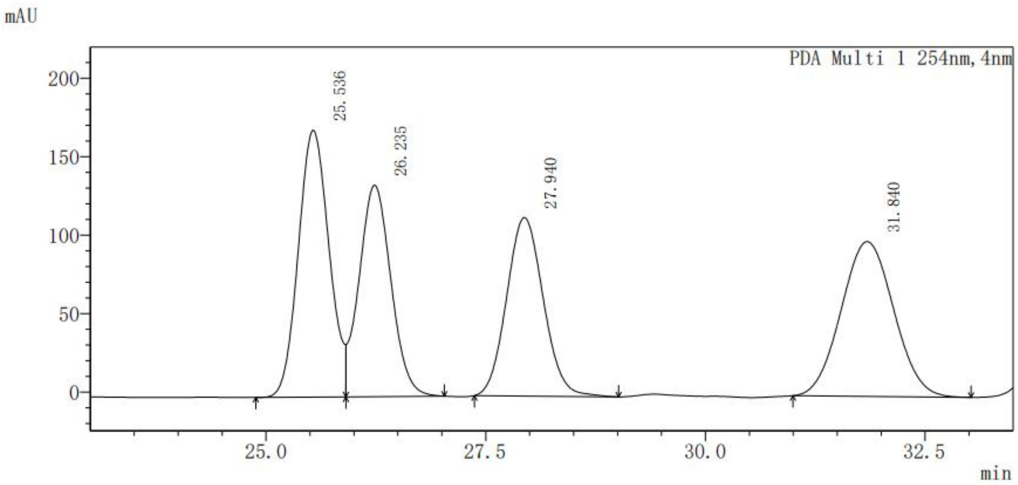

<Peak Results>

PDA Ch1 254nm

Index	Time/min	Height/mAU	Quantity/Area	Area %/%
1	25.536	170129	4141228	27.720
2	26.235	134733	3392706	22.709
3	27.940	113736	3300803	22.094
4	31.840	98659	4104951	27.477

<Chromatogram>

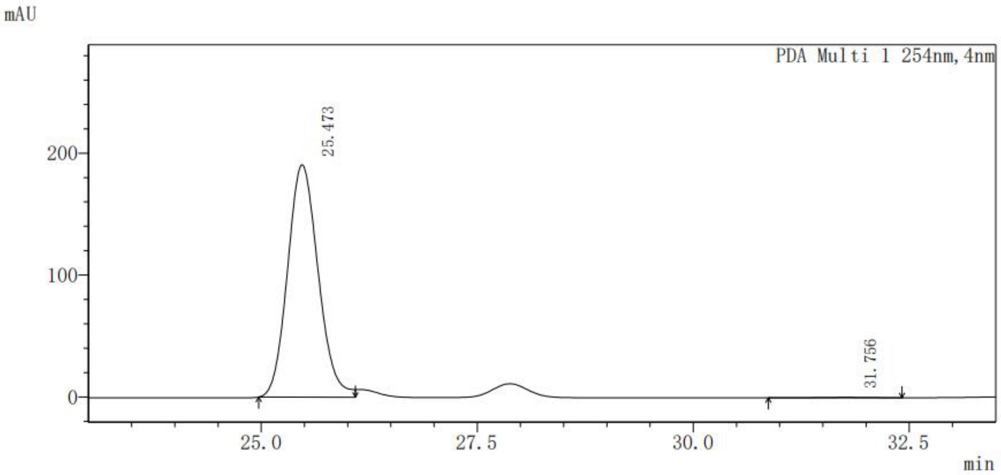

<Peak Results>

PDA Ch1 254nm

Index	Time/min	Height/mAU	Quantity/Area	Area %/%
1	25.473	190687	4696992	99.741
2	31.756	325	12207	0.259

Supplementary Fig. 143. HPLC chromatograms of compound **4e.**

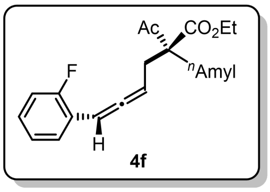

<Chromatogram>

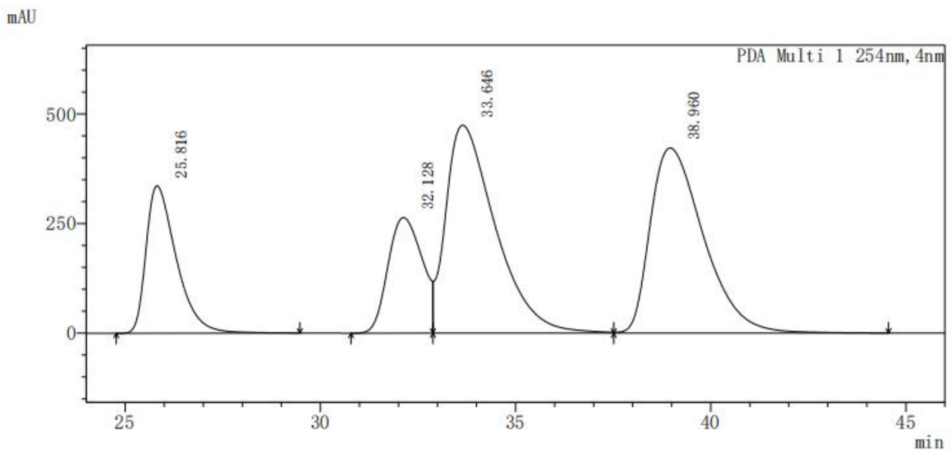

<Peak Results>

PDA Ch1 254nm

Index	Time/min	Height/mAU	Quantity/Area	Area %/%
1	25.816	336480	18172045	15.605
2	32.128	263770	16129478	13.851
3	33.646	474393	42000277	36.068
4	38.960	422582	40146618	34.476

<Chromatogram>

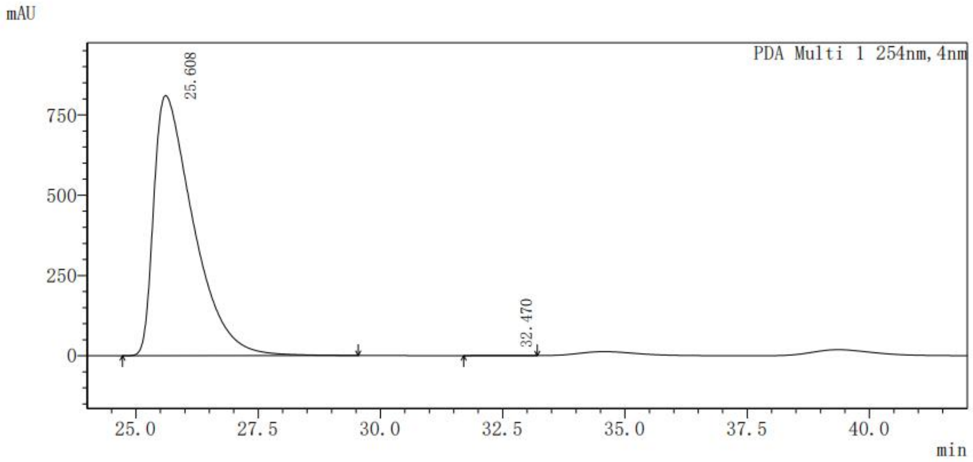

<Peak Results>

PDA Ch1 254nm

Index	Time/min	Height/mAU	Quantity/Area	Area %/%
1	25.608	809989	45757943	99.906
2	32.470	776	43138	0.094

Supplementary Fig. 144. HPLC chromatograms of compound **4f.**

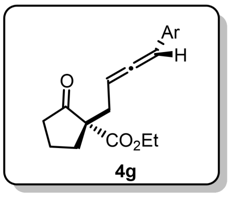

〈Chromatogram〉

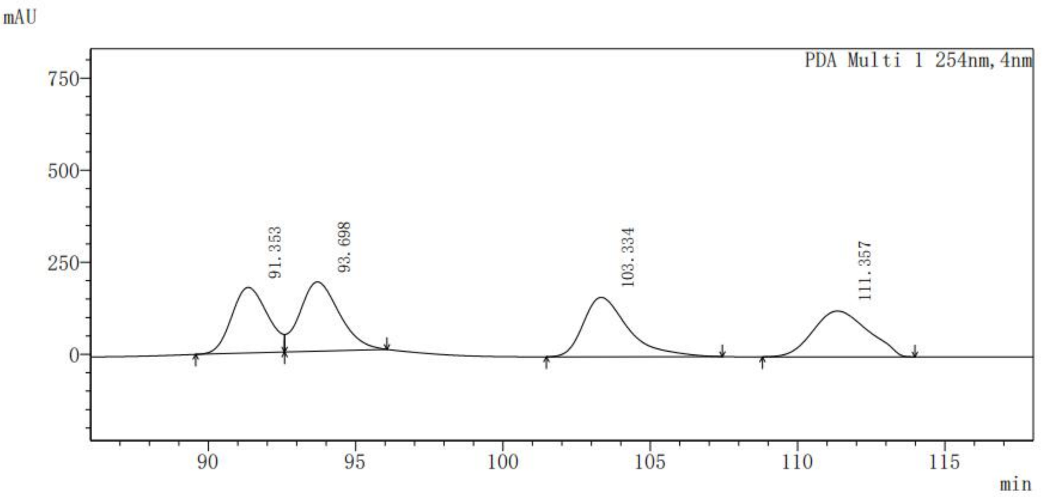

〈Peak Results〉

PDA Ch1 254nm

Index	Time/min	Height/mAU	Quantity/Area	Area %/%
1	91.353	178077	15146084	22.921
2	93.698	188384	17624669	26.672
3	103.334	161210	17126632	25.919
4	111.357	124476	16180833	24.487

〈Chromatogram〉

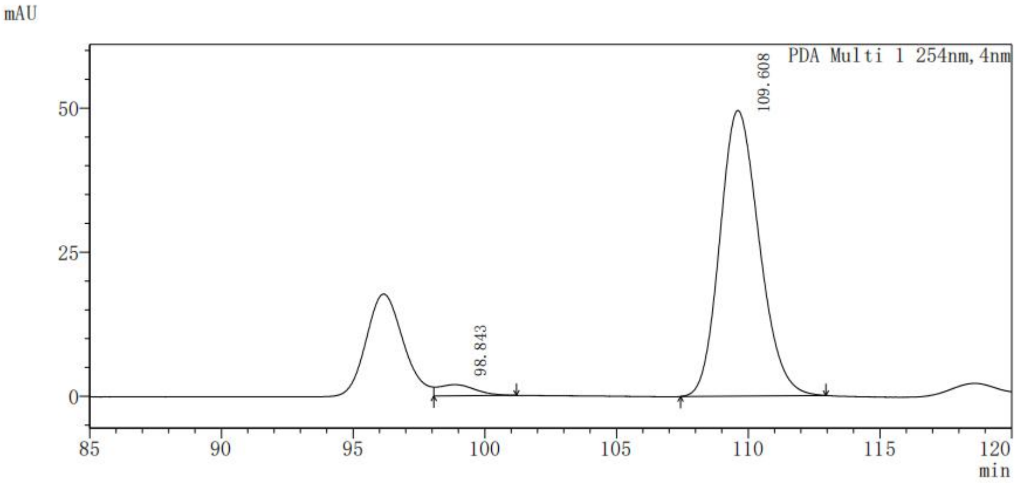

〈Peak Results〉

PDA Ch1 254nm

Index	Time/min	Height/mAU	Quantity/Area	Area %/%
1	98.843	1936	186041	3.482
2	109.608	49561	5156484	96.518

Supplementary Fig. 145. HPLC chromatograms of compound **4g.**

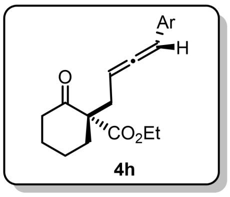

<Chromatogram>

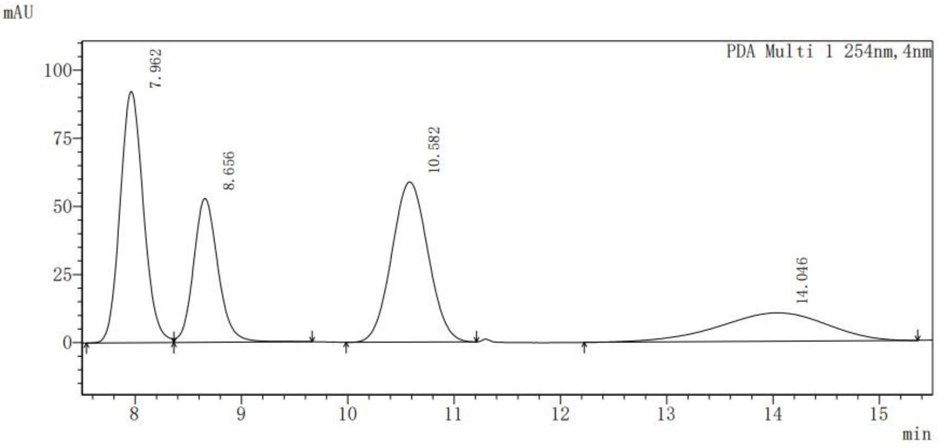

<Peak Results>

PDA Ch1 254nm

Index	Time/min	Height/mAU	Quantity/Area	Area %/%
1	7.962	92179	1408694	32.137
2	8.656	52763	842494	19.220
3	10.582	58853	1395879	31.845
4	14.046	10417	736321	16.798

<Chromatogram>

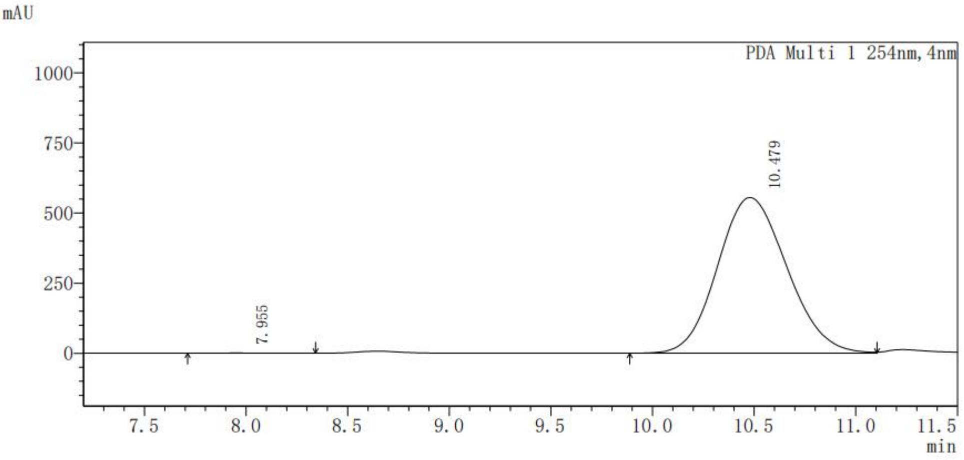

<Peak Results>

PDA Ch1 254nm

Index	Time/min	Height/mAU	Quantity/Area	Area %/%
1	7.955	686	9981	0.076
2	10.479	554937	13163567	99.924

Supplementary Fig. 146. HPLC chromatograms of compound **4h.**

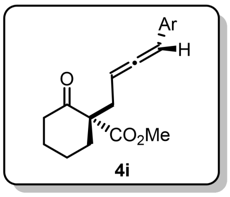

⟨Chromatogram⟩

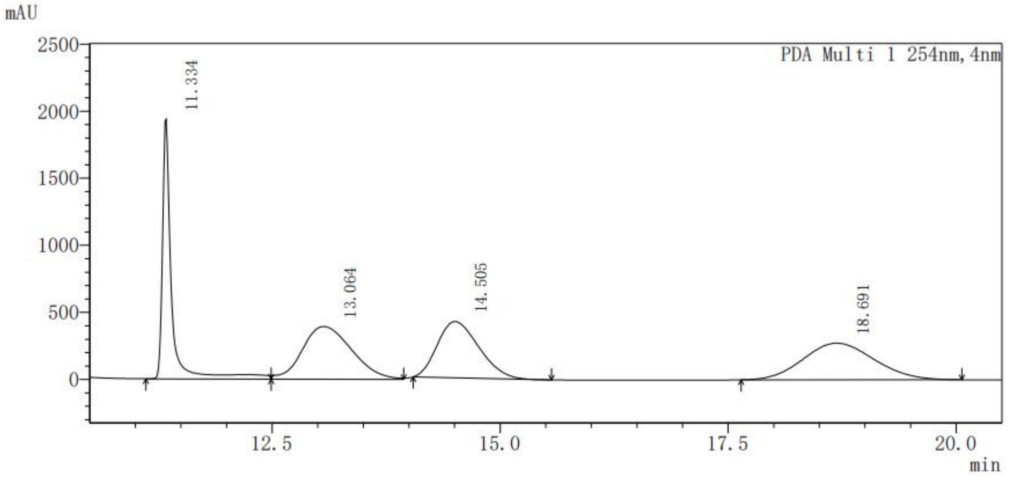

⟨Peak Results⟩

PDA Ch1 254nm

Index	Time/min	Height/mAU	Quantity/Area	Area %/%
1	11.334	1933806	13406534	23.800
2	13.064	392953	14784352	26.246
3	14.505	419043	13288758	23.591
4	18.691	273421	14850136	26.363

⟨Chromatogram⟩

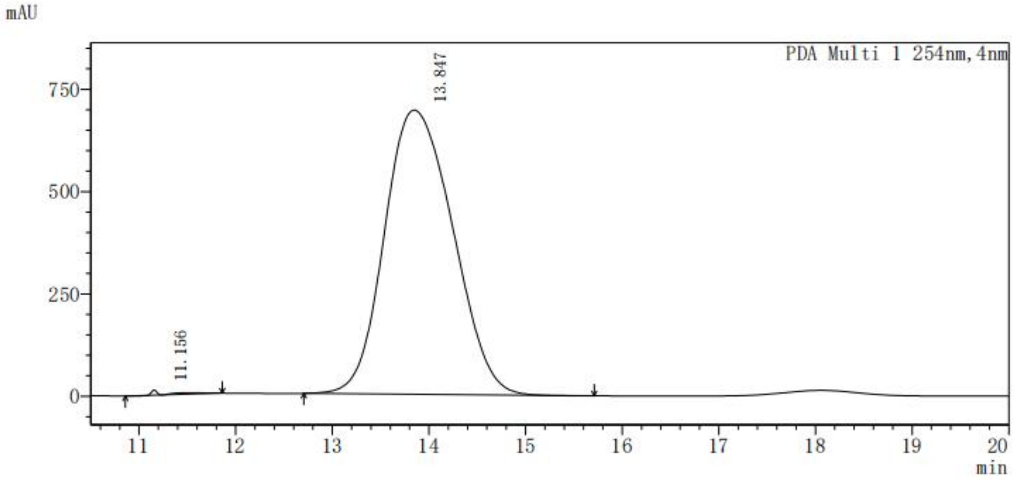

⟨Peak Results⟩

PDA Ch1 254nm

Index	Time/min	Height/mAU	Quantity/Area	Area %/%
1	11.156	12728	115917	0.337
2	13.847	694603	34230038	99.663

Supplementary Fig. 147. HPLC chromatograms of compound **4i.**

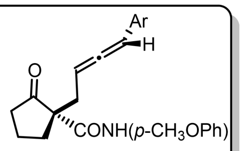

4j

<Chromatogram>

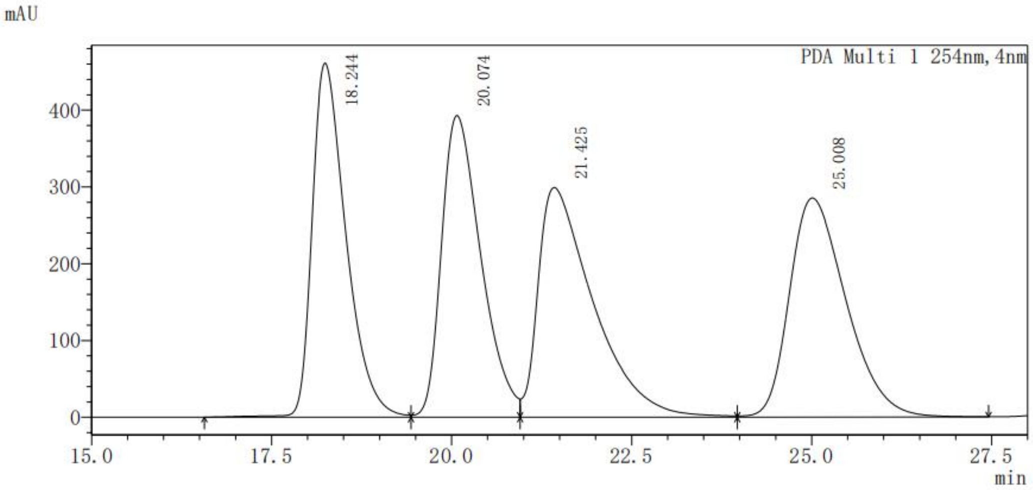

<Peak Results>

PDA Ch1 254nm

Index	Time/min	Height/mAU	Quantity/Area	Area %/%
1	18.244	461000	15078380	24.541
2	20.074	392757	14893535	24.240
3	21.425	298953	15846556	25.791
4	25.008	285197	15623396	25.428

<Chromatogram>

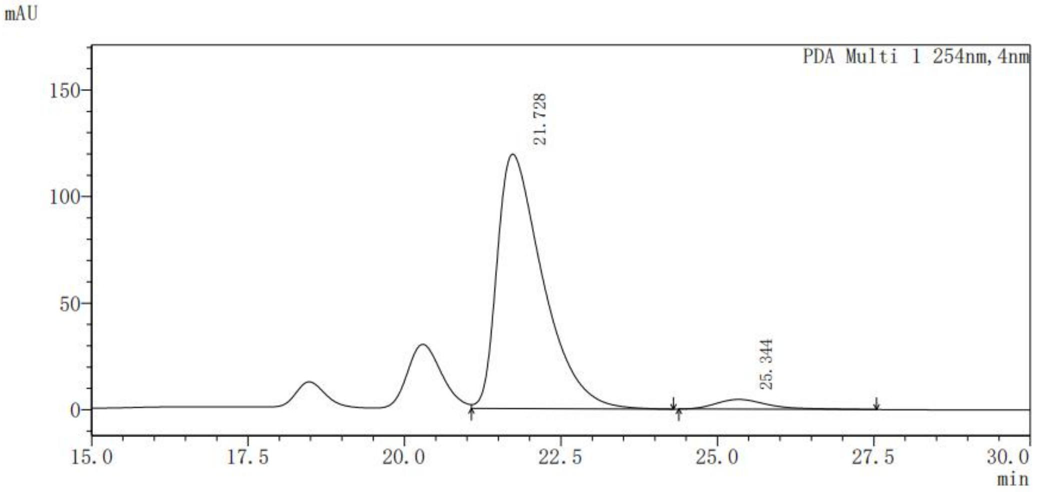

<Peak Results>

PDA Ch1 254nm

Index	Time/min	Height/mAU	Quantity/Area	Area %/%
1	21.728	119420	6075365	95.774
2	25.344	4593	268061	4.226

Supplementary Fig. 148. HPLC chromatograms of compound **4j.**

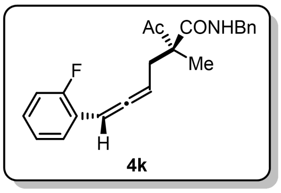

<Chromatogram>

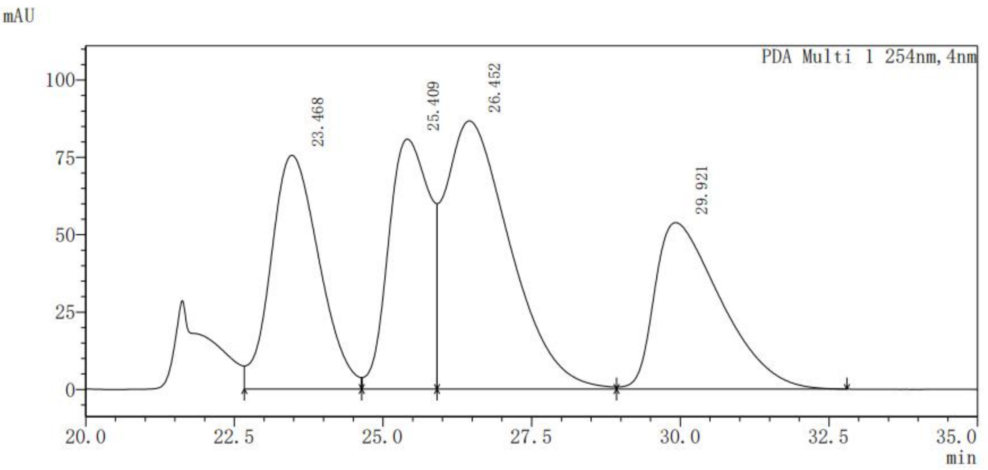

<Peak Results>

PDA Ch1 254nm

Index	Time/min	Height/mAU	Quantity/Area	Area %/%
1	23.468	75600	4252975	22.465
2	25.409	80831	3912594	20.667
3	26.452	86731	6594721	34.835
4	29.921	53829	4171076	22.033

<Chromatogram>

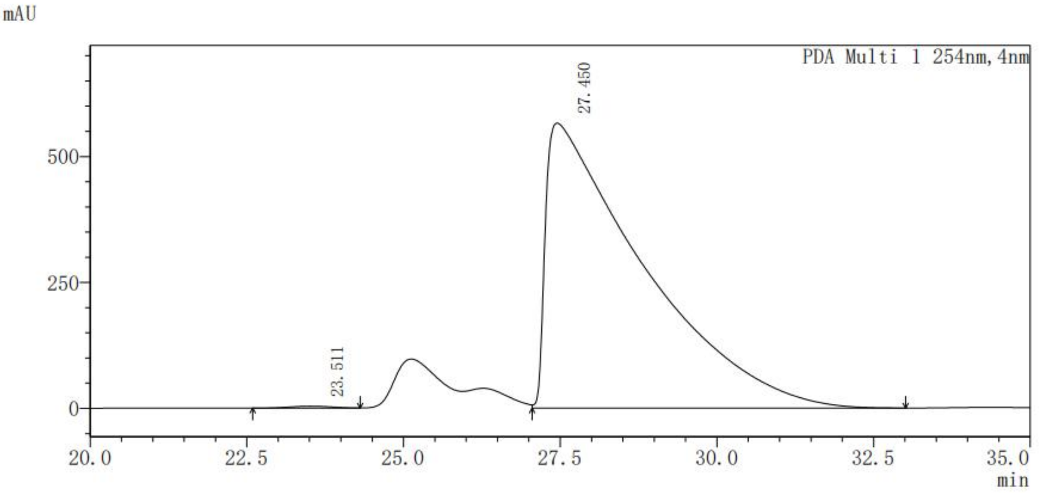

<Peak Results>

PDA Ch1 254nm

Index	Time/min	Height/mAU	Quantity/Area	Area %/%
1	23.511	3957	193040	0.315
2	27.450	565814	61137489	99.685

Supplementary Fig. 149. HPLC chromatograms of compound **4k.**

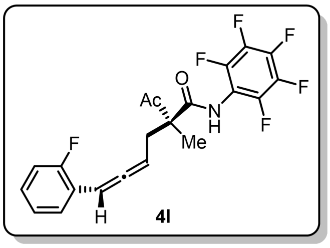

<Chromatogram>

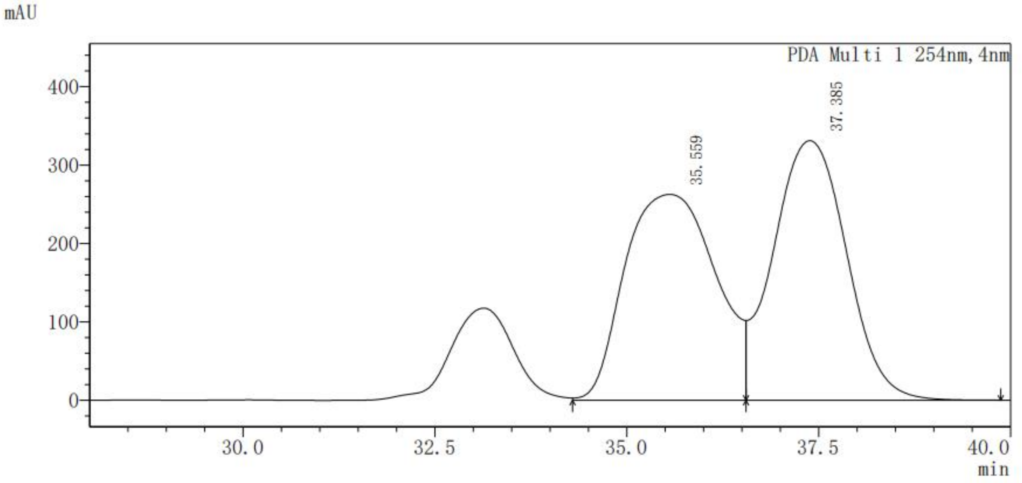

<Peak Results>

PDA Ch1 254nm

Index	Time/min	Height/mAU	Quantity/Area	Area %/%
1	35.559	262465	21667377	49.575
2	37.385	330911	22038638	50.425

<Chromatogram>

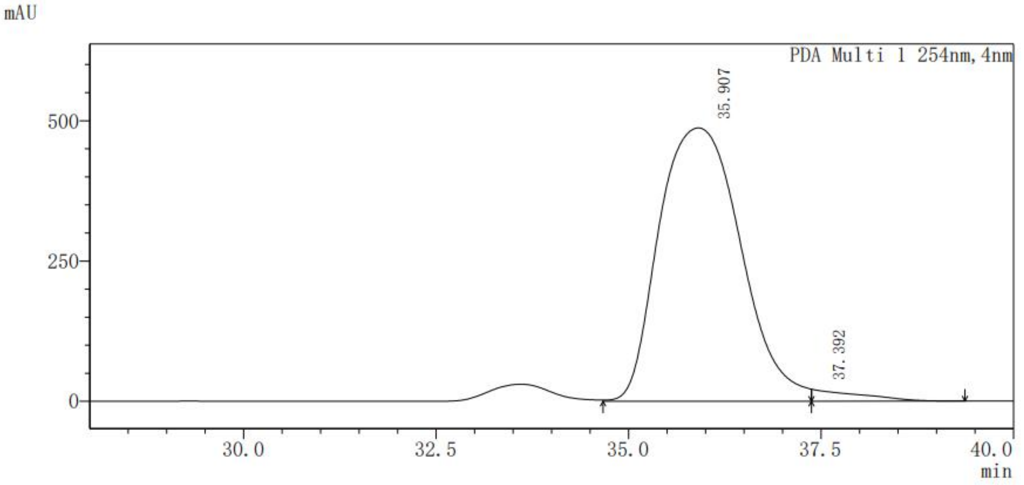

<Peak Results>

PDA Ch1 254nm

Index	Time/min	Height/mAU	Quantity/Area	Area %/%
1	35.907	486857	36016671	97.621
2	37.392	21036	877792	2.379

Supplementary Fig. 150. HPLC chromatograms of compound **4l.**

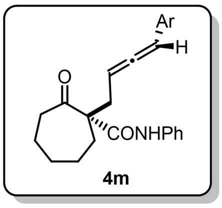

<Chromatogram>

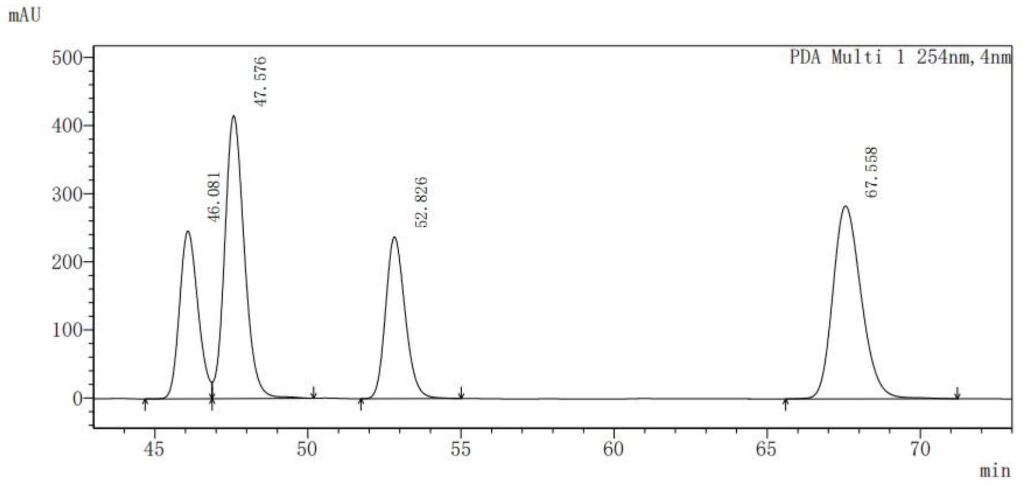

<Peak Results>

PDA Ch1 254nm

Index	Time/min	Height/mAU	Quantity/Area	Area %/%
1	46.081	246360	10337332	17.936
2	47.576	415328	18455775	32.022
3	52.826	237502	10542111	18.291
4	67.558	283201	18299741	31.751

<Chromatogram>

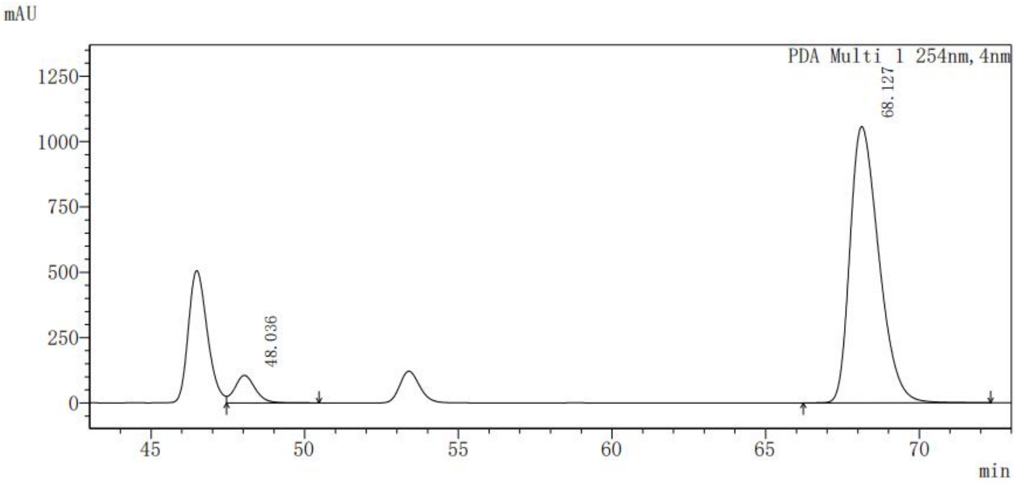

<Peak Results>

PDA Ch1 254nm

Index	Time/min	Height/mAU	Quantity/Area	Area %/%
1	48.036	105274	4829416	6.533
2	68.127	1057584	69092493	93.467

Supplementary Fig. 151. HPLC chromatograms of compound **4m.**

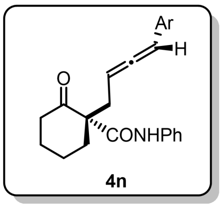

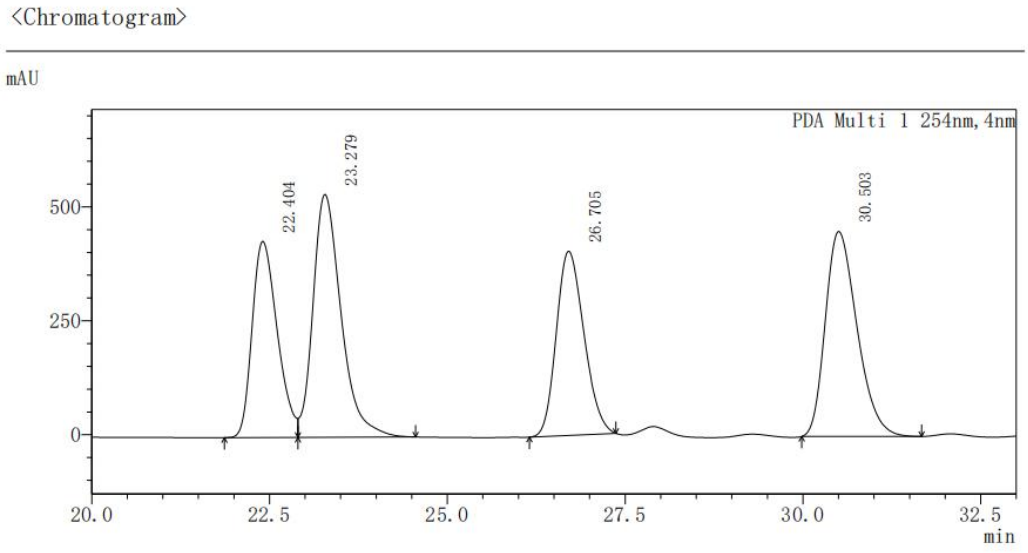

<Peak Results>

PDA Ch1 254nm

Index	Time/min	Height/mAU	Quantity/Area	Area %/%
1	22.404	430522	10577268	21.164
2	23.279	532719	14574860	29.163
3	26.705	404138	10925685	21.861
4	30.503	449777	13899213	27.811

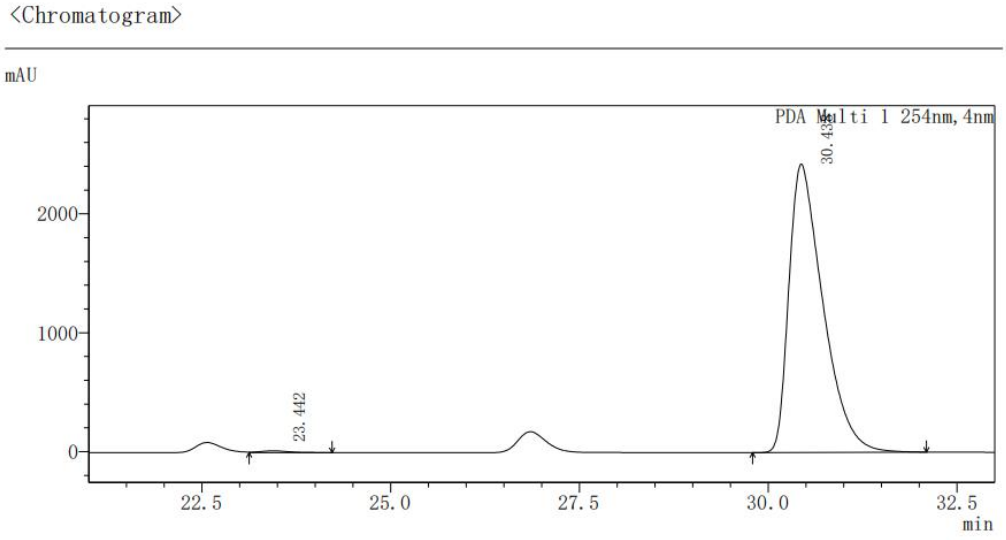

<Peak Results>

PDA Ch1 254nm

Index	Time/min	Height/mAU	Quantity/Area	Area %/%
1	23.442	14715	382342	0.500
2	30.438	2426987	76056114	99.500

Supplementary Fig. 152. HPLC chromatograms of compound **4n.**

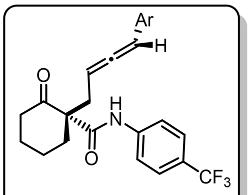

4o

<Chromatogram>

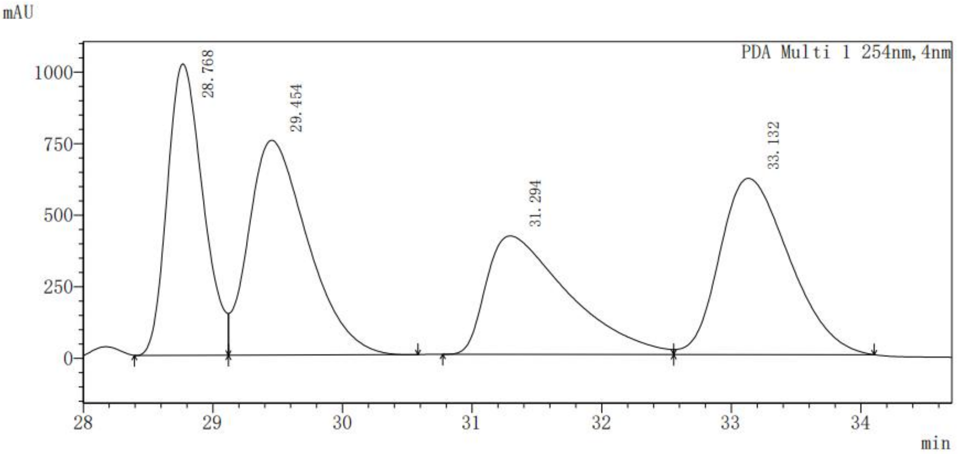

<Peak Results>

PDA Ch1 254nm

Index	Time/min	Height/mAU	Quantity/Area	Area %/%
1	28.768	1019069	19552483	23.120
2	29.454	750424	23786641	28.126
3	31.294	413704	18197222	21.517
4	33.132	615852	23034088	27.237

<Chromatogram>

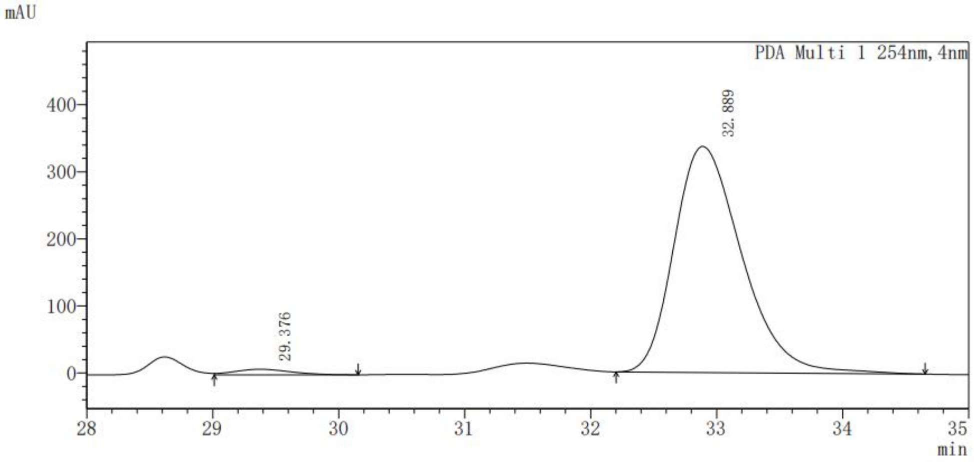

<Peak Results>

PDA Ch1 254nm

Index	Time/min	Height/mAU	Quantity/Area	Area %/%
1	29.376	8470	270567	2.101
2	32.889	337149	12606067	97.899

Supplementary Fig. 153. HPLC chromatograms of compound **4o.**

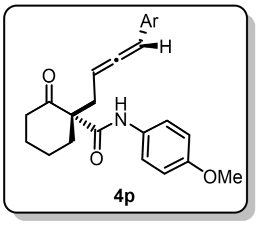

<Chromatogram>

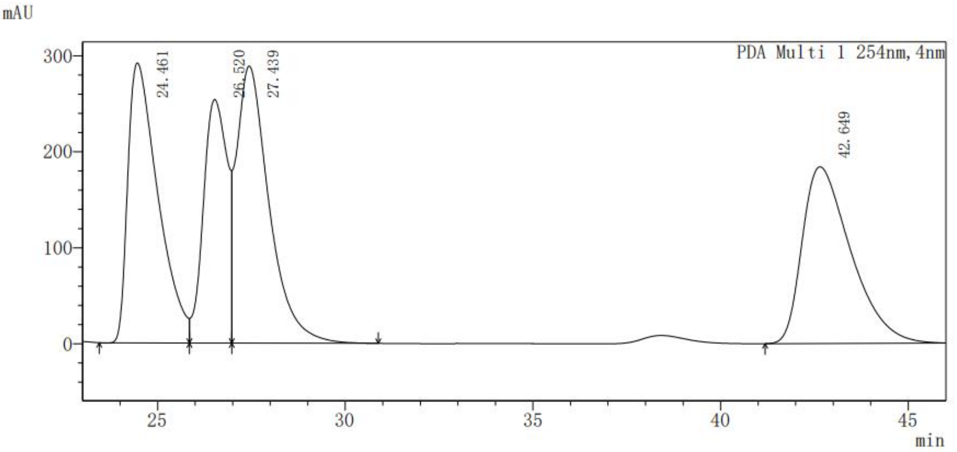

<Peak Results>

PDA Ch1 254nm

Index	Time/min	Height/mAU	Quantity/Area	Area %/%
1	24.461	291567	16311362	26.504
2	26.520	253712	11279010	18.327
3	27.439	288685	17383663	28.246
4	42.649	184035	16569788	26.924

<Chromatogram>

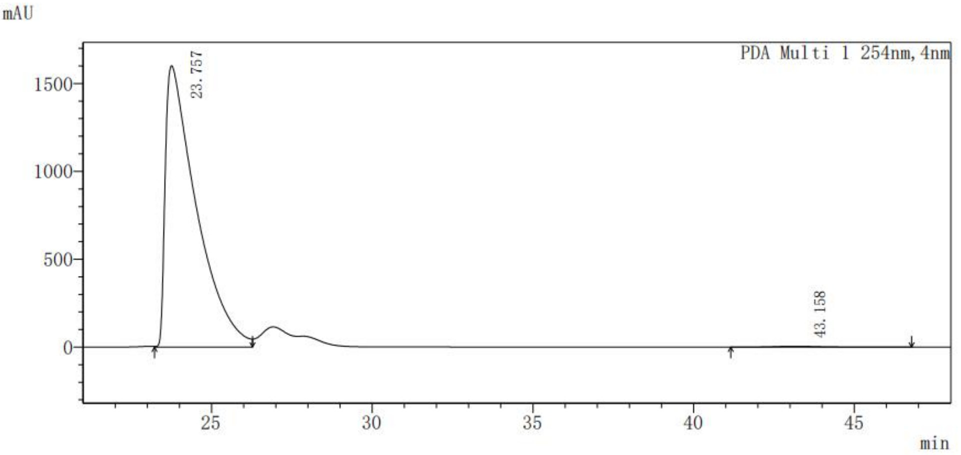

<Peak Results>

PDA Ch1 254nm

Index	Time/min	Height/mAU	Quantity/Area	Area %/%
1	23.757	1601091	107342390	99.625
2	43.158	4587	404336	0.375

Supplementary Fig. 154. HPLC chromatograms of compound **4p.**

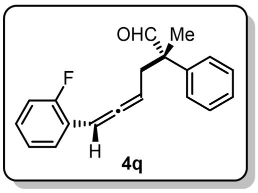

<Chromatogram>

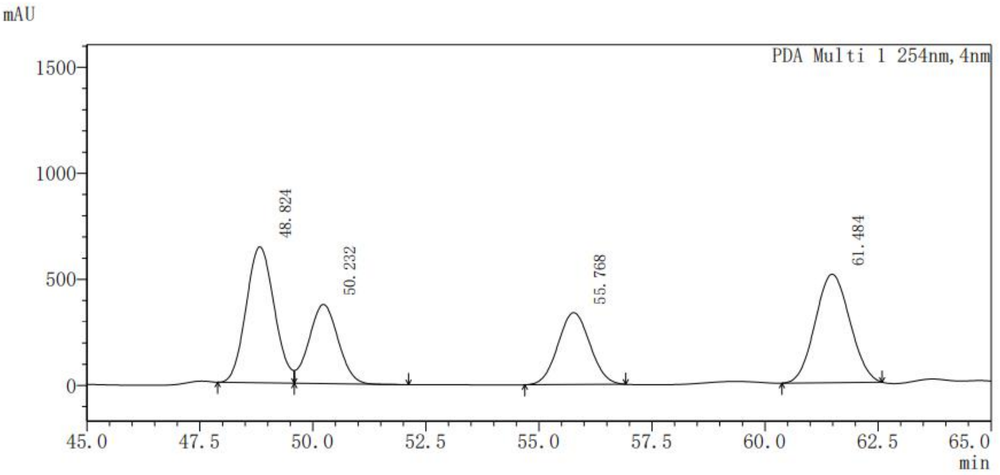

<Peak Results>

PDA Ch1 254nm

Index	Time/min	Height/mAU	Quantity/Area	Area %/%
1	48.824	641577	27849004	31.460
2	50.232	373242	16677612	18.840
3	55.768	339196	16693333	18.858
4	61.484	511012	27302770	30.843

<Chromatogram>

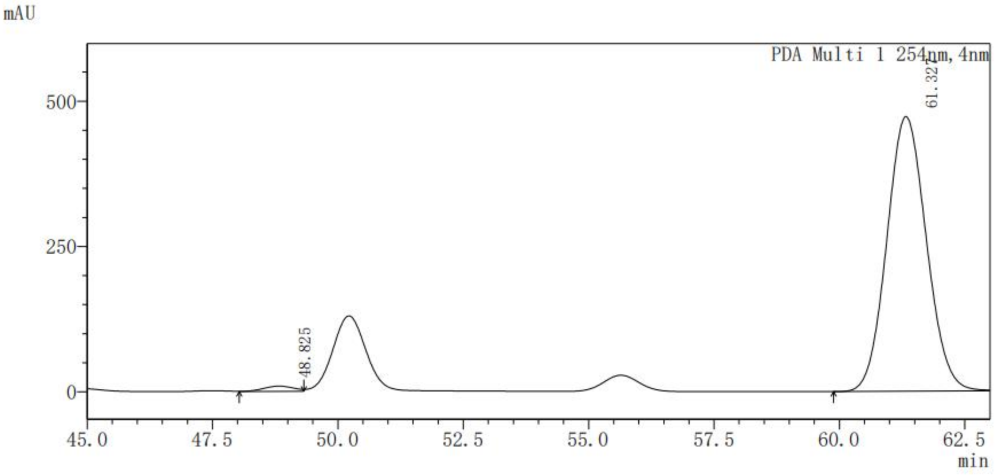

<Peak Results>

PDA Ch1 254nm

Index	Time/min	Height/mAU	Quantity/Area	Area %/%
1	48.825	9206	389059	1.476
2	61.327	472567	25968442	98.524

Supplementary Fig. 155. HPLC chromatograms of compound **4q.**

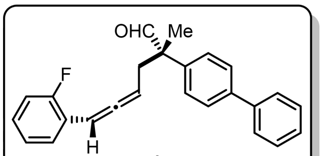

4r

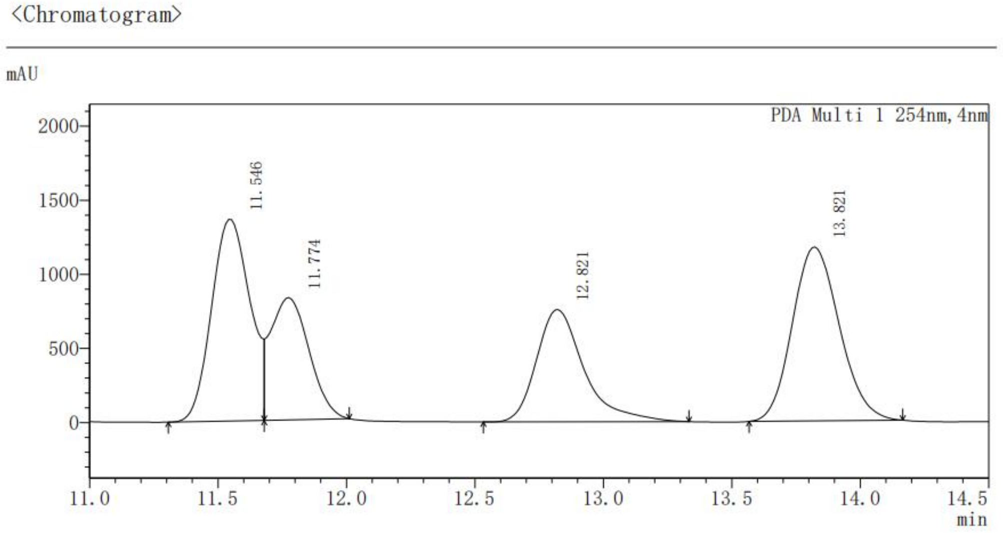

<Peak Results>

PDA Ch1 254nm

Index	Time/min	Height/mAU	Quantity/Area	Area %/%
1	11.546	1361016	14380541	30.266
2	11.774	825261	8429217	17.740
3	12.821	758589	9743126	20.506
4	13.821	1173790	14961403	31.488

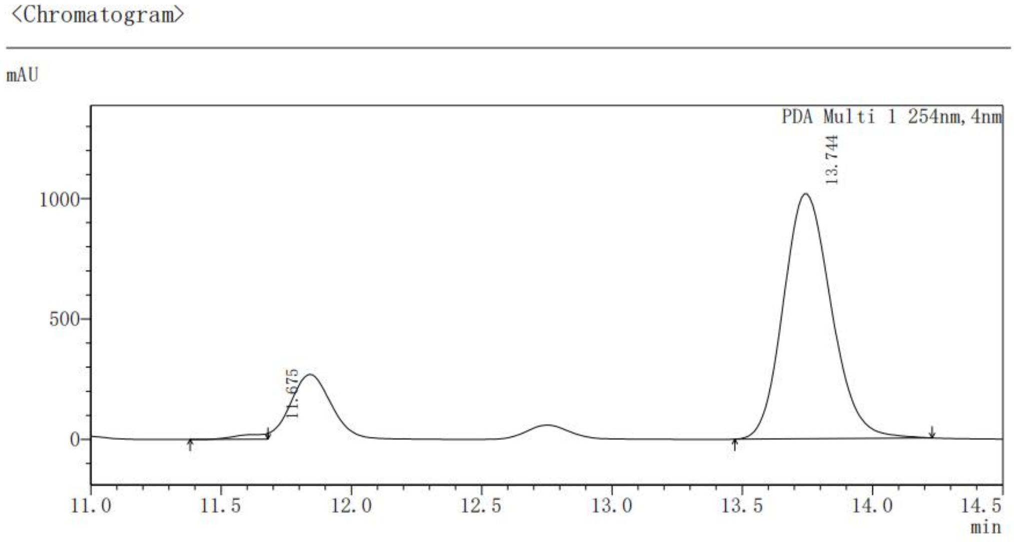

<Peak Results>

PDA Ch1 254nm

Index	Time/min	Height/mAU	Quantity/Area	Area %/%
1	11.675	21068	176307	1.337
2	13.744	1017369	13014074	98.663

Supplementary Fig. 156. HPLC chromatograms of compound **4r.**

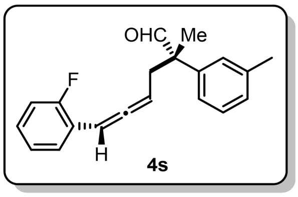

4s

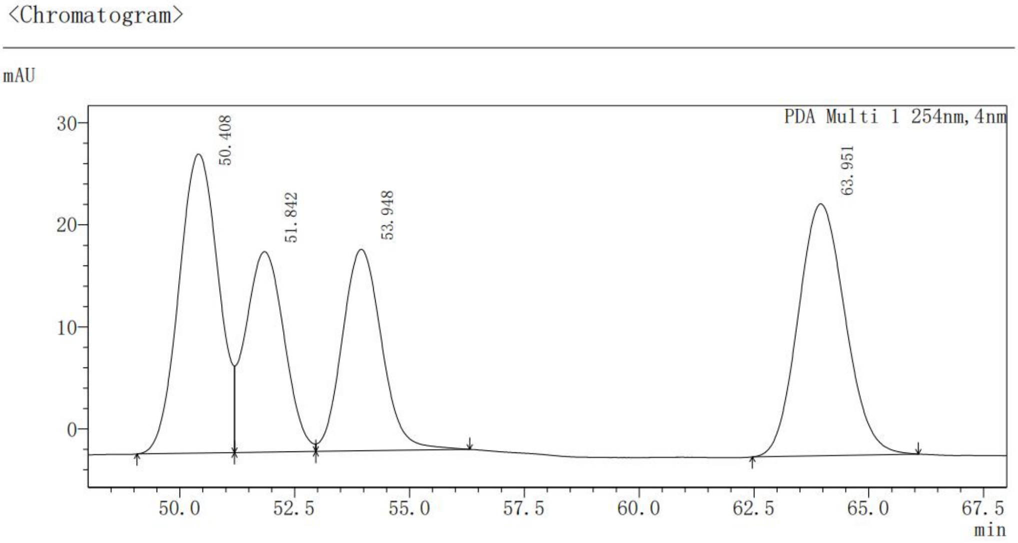

<Peak Results>

PDA Ch1 254nm

Index	Time/min	Height/mAU	Quantity/Area	Area %/%
1	50.408	29320	1753996	30.023
2	51.842	19656	1150659	19.696
3	53.948	19751	1178873	20.179
4	63.951	24695	1758652	30.103

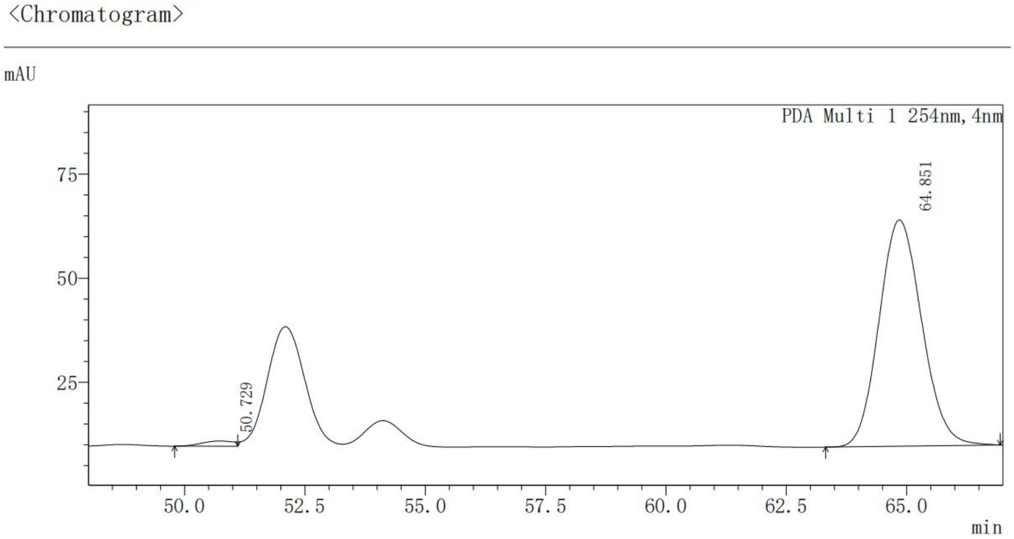

<Peak Results>

PDA Ch1 254nm

Index	Time/min	Height/mAU	Quantity/Area	Area %/%
1	50.729	1259	58107	1.698
2	64.851	54315	3363110	98.302

Supplementary Fig. 157. HPLC chromatograms of compound **4s.**

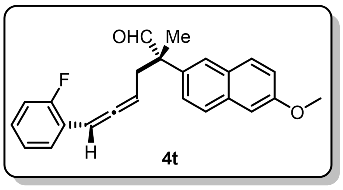

<Chromatogram>

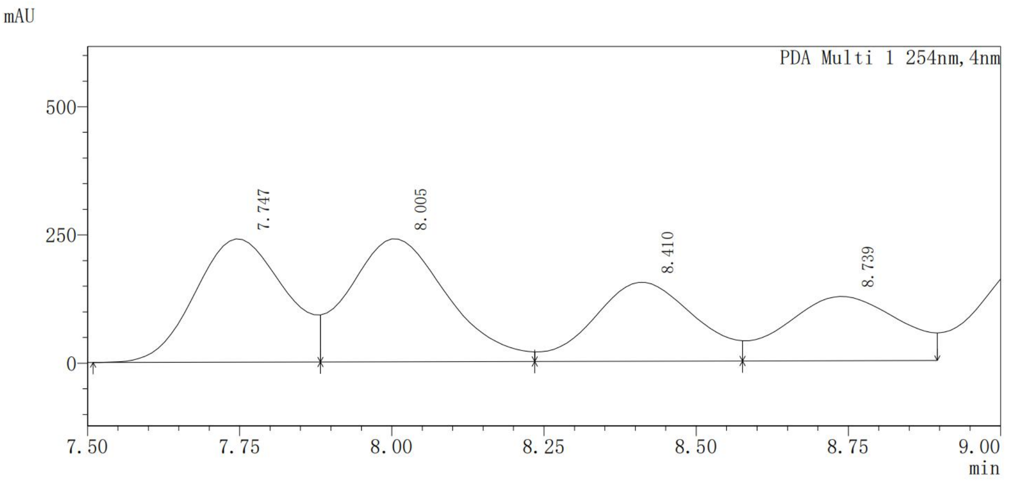

<Peak Results>

PDA Ch1 254nm

Index	Time/min	Height/mAU	Quantity/Area	Area %/%
1	7.747	240318	2503935	29.077
2	8.005	239462	2684868	31.178
3	8.410	153794	1776703	20.632
4	8.739	125365	1645913	19.113

<Chromatogram>

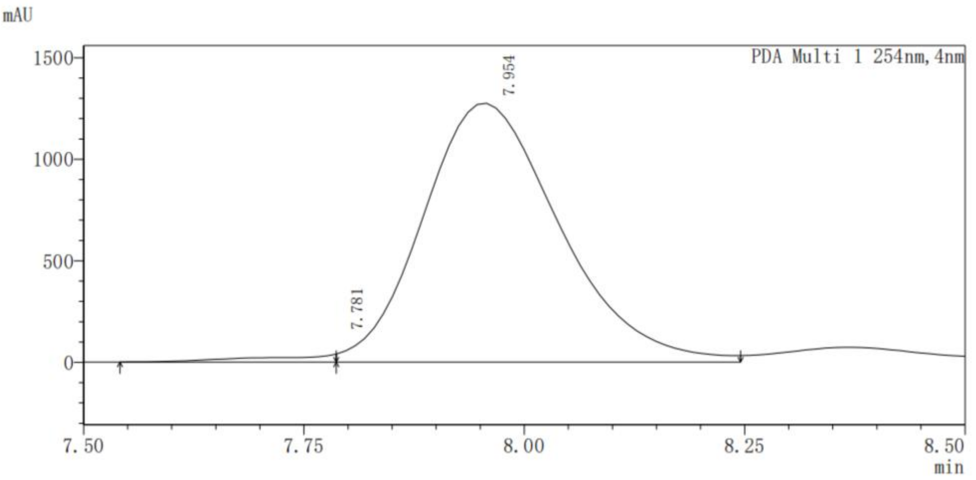

<Peak Results>

PDA Ch1 254nm

Index	Time/min	Height/mAU	Quantity/Area	Area %/%
1	7.781	29933	219228	1.570
2	7.954	1274336	13742766	98.430

Supplementary Fig. 158. HPLC chromatograms of compound **4t.**

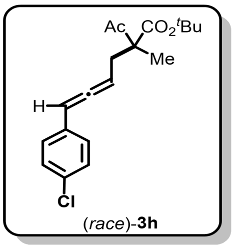

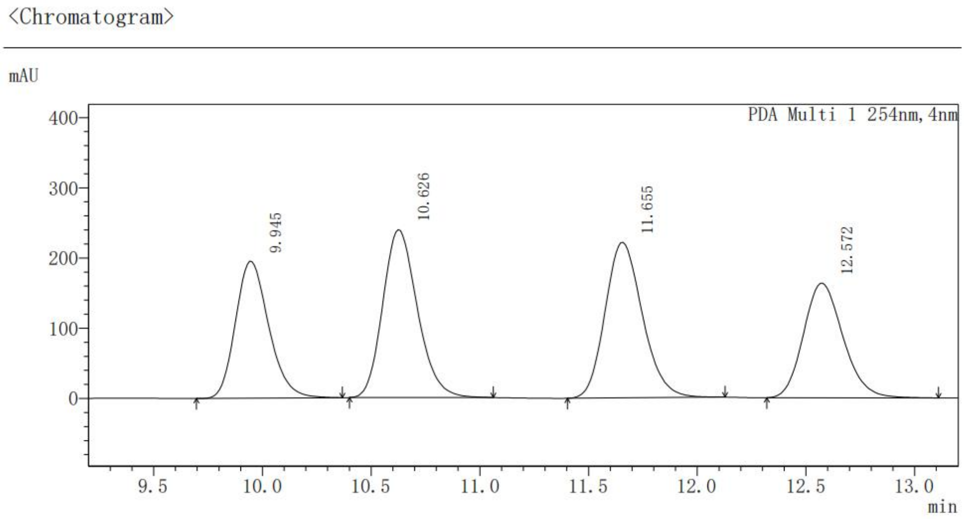

<Peak Results>

PDA Ch1 254nm

Index	Time/min	Height/mAU	Quantity/Area	Area %/%
1	9.945	195035	2077152	22.018
2	10.626	239114	2649189	28.082
3	11.655	221414	2644863	28.036
4	12.572	163205	2062588	21.864

Supplementary Fig. 159. HPLC chromatograms of compound (*race*)-**3h.**

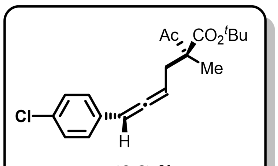

(*S*,*S*)-**3h**

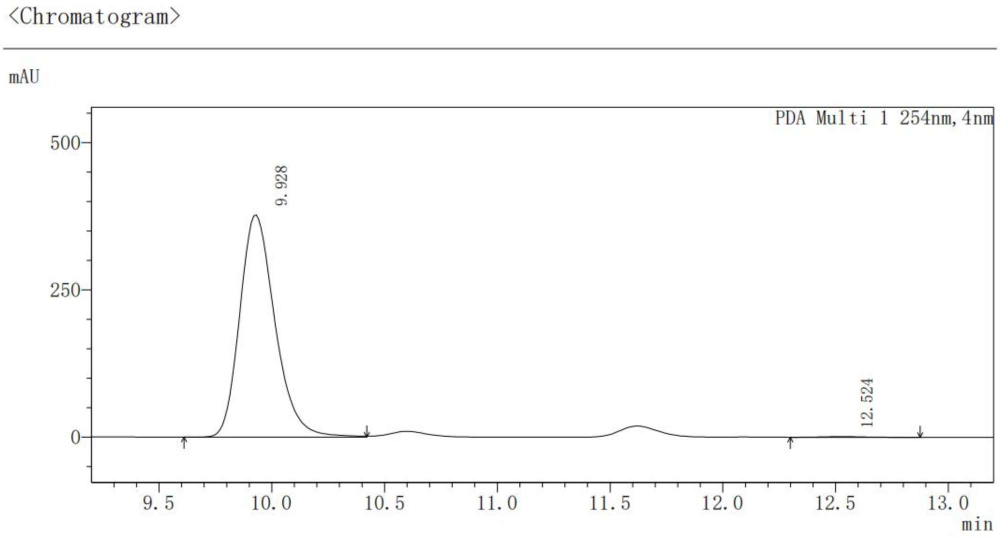

<Peak Results>

PDA Ch1 254nm

Index	Time/min	Height/mAU	Quantity/Area	Area %/%
1	9.928	377232	4106724	99.672
2	12.524	1118	13507	0.328

Supplementary Fig. 160. HPLC chromatograms of compound (*S*,*S*)-**3h.**

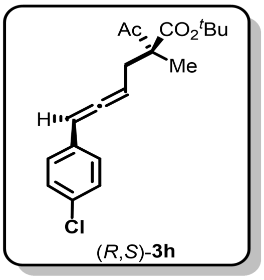

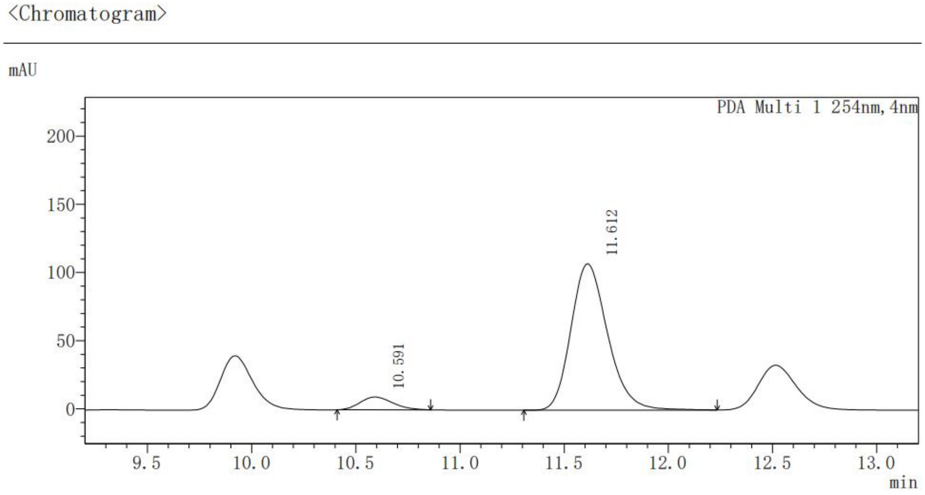

<Peak Results>

PDA Ch1 254nm

Index	Time/min	Height/mAU	Quantity/Area	Area %/%
1	10.591	9426	102041	7.178
2	11.612	107363	1319564	92.822

Supplementary Fig. 161. HPLC chromatograms of compound (*R,S*)-**3h.**

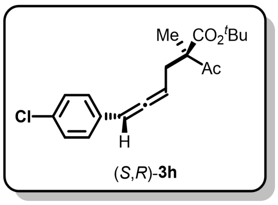

<Chromatogram>

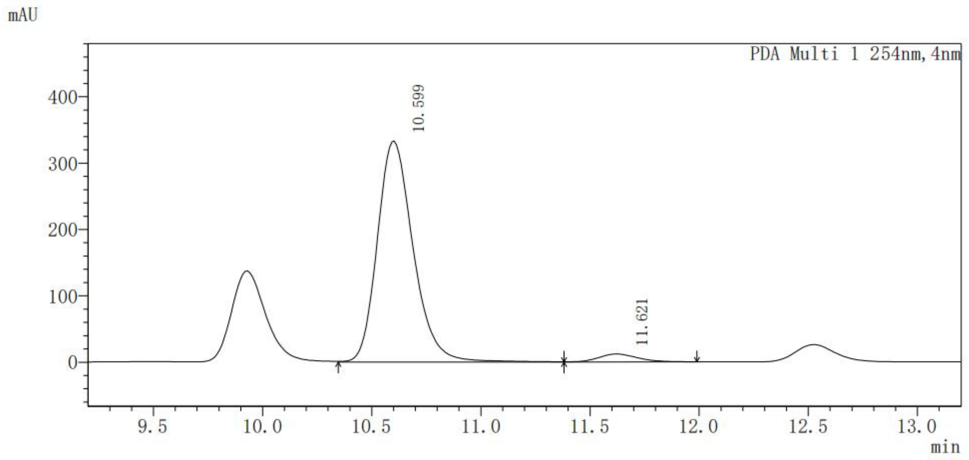

<Peak Results>

PDA Ch1 254nm

Index	Time/min	Height/mAU	Quantity/Area	Area %/%
1	10.599	333110	3799947	96.177
2	11.621	12070	151061	3.823

Supplementary Fig. 162. HPLC chromatograms of compound (*S*,*R*)-**3h.**

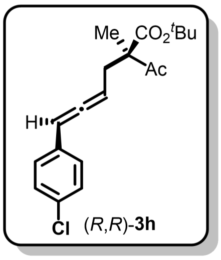

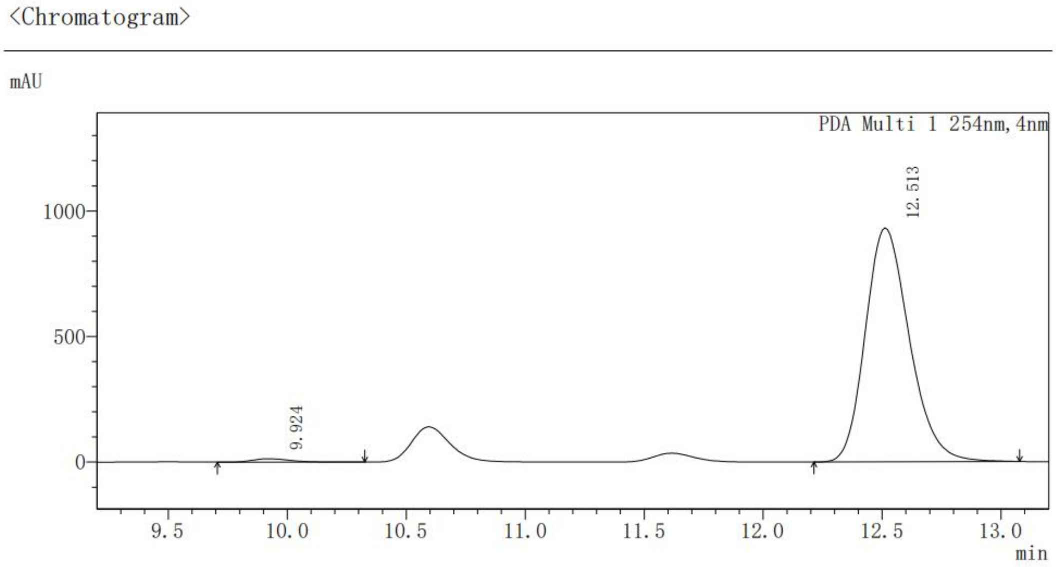

<Peak Results>

PDA Ch1 254nm

Index	Time/min	Height/mAU	Quantity/Area	Area %/%
1	9.924	13428	147181	1.213
2	12.513	931653	11987271	98.787

Supplementary Fig. 163. HPLC chromatograms of compound (*R,R*)-**3h.**

5. Supplementary References

[1] Zhou, H., Zhang, L., Xu, C.-M. & Luo, S.-Z. Chiral primary amine/palladium dual catalysis for asymmetric allylic alkylation of β-ketocarbonyl compounds with allylic alcohols. *Angew. Chem. Int. Ed.* **54**, 12645-12648 (2015).

[2] Hoffmann, S., Nicoletti, M. & List, B. Catalytic asymmetric reductive amination of aldehydes via dynamic kinetic resolution. *J. Am. Chem. Soc*. **128**, 13074 (2006).

[3] Yang, S.-Q., Wang, Y.-F., Zhao, W.-C., Lin, G.-Q. & He, Z.-T. Stereodivergent synthesis of tertiary fluoride-tethered allenes via copper and palladium dual catalysis. J. Am. Chem. Soc. **143**, 7285-7291 (2021).

[4] Li, Q.-Y., Fang, X.-X.; Pan, R., Yao, H.-Q. & Lin, A.-J. Palladium-catalyzed asymmetric sequential hydroamination of 1,3-enynes: enantioselective syntheses of chiral imidazolidinones. *J. Am. Chem. Soc.* **144**, 11364-11376 (2022).
